# Age- and sex-specific attributable impact of modifiable risk factors on atherosclerotic cardiovascular disease in Korea: a nationwide population-based cohort study

**DOI:** 10.1016/j.lanwpc.2026.101844

**Published:** 2026-04-03

**Authors:** Heesun Lee, Eungjae Kang, Tae-Min Rhee, Ju Hi Park, Jee Ye Kahng, Sangjun Lee, Soyeoun Kim, Su-Yeon Choi

**Affiliations:** a Department of Internal Medicine, Seoul National University College of Medicine, Seoul, Republic of Korea; b Division of Cardiology, Seoul National University Hospital Healthcare System Gangnam Center, Seoul, Republic of Korea; c Integrated Major in Innovative Medical Science, Seoul National University Graduate School, Seoul, Republic of Korea; d Department of Preventive Medicine, Seoul National University College of Medicine, Seoul, Republic of Korea; e Cancer Research Institute, Seoul National University, Seoul, Republic of Korea; f Biomedical Research Institute, Seoul National University Hospital, Seoul, Republic of Korea

**Keywords:** Atherosclerotic cardiovascular diseases, Modifiable risk factors, Population-attributable fraction, Primary prevention

## Abstract

**Background:**

While modifiable risk factors are central to the development of atherosclerotic cardiovascular disease (ASCVD), their relative contributions differ by age, sex, region, and period. However, contemporary evidence on these patterns in the large-scale Korean population remains scarce. We aimed to quantify the age- and sex-specific burden of major modifiable risk factors for ASCVD in a large nationwide cohort.

**Methods:**

This retrospective cohort study included 6,249,852 Korean adults without prior ASCVD who underwent the National Health Screening between 2009 and 2010. The primary outcome was incident ASCVD, defined as cardiovascular death, myocardial infarction, or ischemic stroke. Participants were followed for these outcomes through linkage to the Korean National Health Insurance Service claims database and Statistics Korea mortality data. Population-attributable fractions (PAFs) for risk factors—body mass index, systolic blood pressure (SBP), smoking, diabetes, and non-HDL-cholesterol—were estimated from multivariable-adjusted Cox proportional hazards models and stratified by age and sex.

**Findings:**

In this cohort (mean age 46.9 ± 13.7 years; 52.2% men), 279,093 ASCVD events occurred (incidence rate, 3.58/1000 person-years) during a median follow-up of 13.0 years. The five risk factors collectively explained 46.2% of the ASCVD burden, with the greatest contribution from SBP, followed by smoking, non-HDL-cholesterol, diabetes, and obesity. PAFs were higher in men (52.8%) than women (30.4%), and highest in men aged <50 years (71.5%), where SBP (48.6%) and smoking (29.4%) predominated. In women, SBP remained the leading contributor with less variation by age, whereas diabetes and atherogenic lipids contributed more prominently at older ages.

**Interpretation:**

These findings reveal distinct age- and sex-specific patterns in the burden of modifiable risk factors in contemporary Koreans. Tailored prevention strategies that reflect national epidemiological profiles and demographic risk patterns are needed to effectively reduce the ASCVD burden.

**Funding:**

None.

**Research in context**

**Evidence before this study**

Despite remarkable advances in the diagnosis and treatment of atherosclerotic cardiovascular disease (ASCVD) over recent decades, morbidity and mortality from ASCVD remain a significant global health burden. Accordingly, more precise assessment of risk factors and proactive prevention are essential to further reduce risk and optimize global cardiovascular health. We conducted a comprehensive search via PubMed up to April 2025 using combinations of terms such as “population attributable fraction”, “atherosclerotic cardiovascular disease”, “modifiable risk factors”, “age”, “sex”, and “East Asia” including “Korea”. Previous global cohort analyses–notably the 2023 *New Engl J Med* report by the Global Cardiovascular Risk Consortium, including over 1·5 million individuals from 112 cohorts across 34 countries–estimated that approximately half or slightly more of incident ASCVD burden is attributable to five modifiable risk factors (blood pressure [BP], lipids, smoking, diabetes, and obesity). Importantly, studies indicate that the distribution and contribution of each risk factor can vary by population. However, there have been gaps in stratified evidence from East Asia—especially for Korea. Most global studies include relatively limited East Asian data or do not provide detailed age- and sex-specific estimates for this region. In Korea, several smaller cohort studies (e.g., KoGES, NHIS subcohorts) have reported population-attributable fractions (PAFs) of individual risk factors for ASCVD, they have been limited by sample size, restricted age range, or insufficient demographic stratification. Therefore, before this study, the global importance of modifiable risk factors was well recognized, but evidence in East Asian settings was limited and lacked the granular stratification by age and sex needed to inform local policy.

**Added value of this study**

This study explicitly addresses the evidence gap by analyzing a nationwide cohort of over 6 million individuals in Korea–one of the largest population-based investigations of modifiable risk factors in an East Asian country to date. Our study provides comprehensive, age- and sex-specific PAFs of the major modifiable risk factors for ASCVD in a contemporary Korean population. The scale and detail of this dataset enabled us to identify unique patterns of demographic heterogeneity in the contribution of risk factors. For example, the total PAF for ASCVD in Korean women was 30.4%, markedly lower than that in Korean men (52.8%). This stands in contrast to the results of the Global Cardiovascular Risk Consortium, where the total PAF was higher in women (57.2%) than in men (52.6%). Notably, we found that the relative importance of each risk factor varied across age groups and between sexes within Korea. The relatively lower PAF in Korean women likely reflect distinctive behavioral and cultural characteristics, particularly a lower prevalence of obesity (27.7%) and a very low smoking rate (approximately 5%) due to social stigma. Furthermore, our study highlights a significantly higher ASCVD burden attributable to modifiable risk factors in men aged <50 years, with a total PAF of 71.5%. In this age group, systolic BP (48.6%) and smoking (29.4%) were the predominant contributors to preventable ASCVD burden. This suggests a greater susceptibility to metabolic insults in early adulthood. This observation supports evidence that early-life exposure to cardiovascular risk factors accelerates vascular ageing and long-term risk accumulation. By providing the robust quantification of how these risk factors drive ASCVD in large-scale Korean adults across diverse demographic strata, this study adds region-specific insights that complements global findings and inform targeted national prevention strategies.

**Implications of all the available evidence**

Considering both global and local evidence, it is clear that while a core set of modifiable risk factors underlies a substantial proportion of ASCVD worldwide, their attributable burden is not uniform across all populations. Consistent with the previous research, our findings underscore that the necessity for public health strategies to move beyond generic cardiovascular prevention and instead tailor interventions to the predominant risks within specific demographic groups and regions. In practical terms, this means prioritizing different interventions for different contexts–for example, aggressive BP control in populations or age groups where hypertension is the leading contributor or targeted smoking cessation efforts where smoking drives a disproportionate share of risk. Our demonstration of significant age- and sex-specific variation in the impact of risk factors, especially within an East Asian cohort, reinforces that “one-size-fits-all” approaches are suboptimal. Rather, demographically nuanced and regionally adapted prevention policies are needed to maximize reductions in ASCVD. By integrating Korea-specific insights with global evidence, health authorities can formulate more effective, equity-oriented prevention strategies–allocating resources and implementing interventions that address the most consequential risk factors for each subpopulation. Such an individualized approach is essential for improving global cardiovascular health and achieving further gains in ASCVD reduction, particularly as countries like Korea face an ageing population and evolving risk factor profiles.

## Introduction

Atherosclerotic cardiovascular disease (ASCVD) remains the leading cause of mortality and morbidity worldwide, causing approximately one-third of all deaths and imposing substantial medical and socioeconomic burdens.^1^^,^^2^ In response, there has been a paradigm shift from traditional treatment-centred approaches toward proactive strategies focused on early detection and prevention. Central to this shift is the precise identification and effective management of modifiable risk factors.^3^^,^^4^ In this context, the “Life's Simple 7” framework highlights the importance of both behavioral (e.g., smoking, physical activity, diet, and body weight) and clinical factors (e.g., hypertension, diabetes, and dyslipidemia) in promoting cardiovascular health.^5^ Indeed, prior large-scale studies have consistently shown that a limited number of modifiable risk factors account for the majority of ASCVD risk.^6^^,^^7^ The INTERHEART study attributed over 90% of acute myocardial infarction (MI) risk to nine key factors across 52 countries,^6^ while the PURE study demonstrated that metabolic, behavioral, and socioeconomic factors collectively explain more than 70% of global cardiovascular events.^7^ Most recently, the Global Cardiovascular Risk Consortium reported that five major risk factors—hypertension, diabetes, dyslipidemia, obesity, and smoking—accounted for approximately half of global ASCVD burden.^8^ Although the standardized risk assessment tools incorporating these risk factors are widely used in clinical practice, considerable heterogeneity in their distribution and impact has been observed across populations,^8^, ^9^, ^10^ and these models frequently overestimate ASCVD risk in East Asians as they were primarily derived from Western cohorts.^11^ Despite such recognized differences, few studies have comprehensively evaluated the age-, sex-, and region-specific prevalence of cardiovascular risk factors and their relative contributions to ASCVD, particularly using individual-level data from Korean populations.^8^ To address this gap, we aimed to characterize the age- and sex-specific distributions of modifiable risk factors and quantify their contributions to ASCVD in a large, nationwide, real-world cohort of over 6 million Korean adults free of baseline ASCVD. Furthermore, we sought to contextualize these findings in relation to global data across different geographic regions.

## Methods

### Data source, study design and population

We conducted a retrospective, population-based cohort study utilizing data from the Korean National Health Insurance Service (NHIS) database, which encompasses comprehensive and anonymized health information derived from South Korea's mandatory universal health insurance program. The NHIS database includes detailed records of inpatient and outpatient visits, diagnostic and procedural codes based on the International Classification of Diseases, 10th revision (ICD-10), prescriptions, and medical interventions.^12^ In addition, the NHIS collects data from standardized biennial National Health Screening examinations offered to all insured employees, regardless of age, and to individuals aged ≥40 years, including non-employees. Although not all eligible individuals undergo screening, the NHIS Health Screening cohort provides a large nationwide sample that is broadly representative of the Korean adult population.^13^

**Fig. 1:**
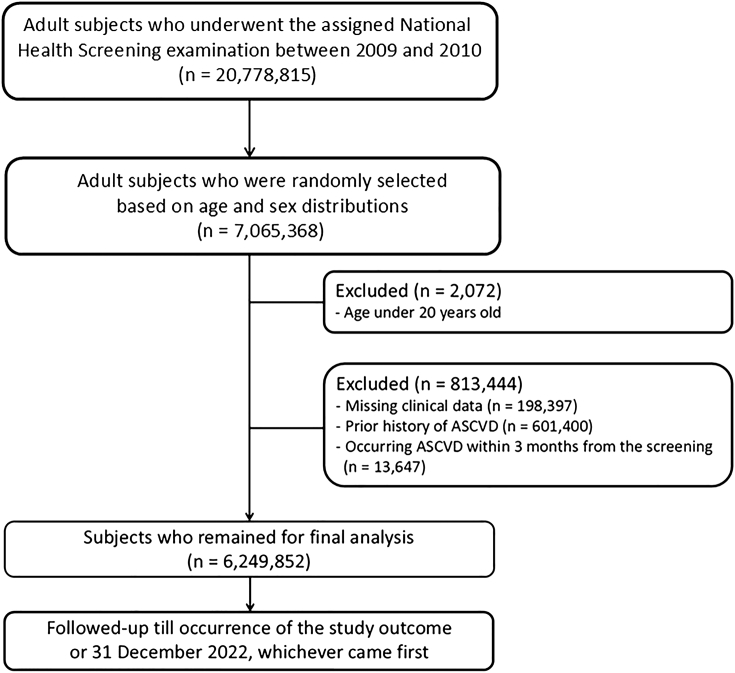
**Flowchart of the study design**. Approximately 6.3 million adults without prior ASCVD history who underwent the National Health Screening Examinations were enrolled in this cohort. They were followed up till the occurrence of incident ASCVD, defined as a composite of cardiovascular death, MI, and ischemic stroke, or 31 December 2022, whichever came first. ASCVD, atherosclerotic cardiovascular disease; MI, myocardial infarction.

The initial cohort consisted of 7,065,368 adults who participated in the National Health Screening between 1 January 2009, and 31 December 2010, representing a random sample of all participating Korean adults based on age and sex during the recruitment period (n = 20,778,815). This random sampling policy was implemented by the NHIS to ensure data confidentiality and to facilitate efficient management of the extremely large source population. From the initial cohort, we excluded individuals aged <20 years (n = 2072), those with missing data in any of the key variables used in the analysis, including body mass index (BMI), systolic blood pressure (SBP), smoking, diabetes, non-high-density lipoprotein cholesterol (non-HDL-c), and other relevant clinical or laboratory variables (n = 198,397), a prior history of ASCVD (n = 601,400), or outcome events occurring within 3 months of the index screening date (n = 13,647). The final analytic cohort included 6,249,852 individuals (Fig. 1).

### Definition of variables, including cardiovascular risk factors

Age and sex were derived from the resident registration number at birth. All measurements were performed as part of the National Health Screening examinations administered by the NHIS and regulated by the Ministry of Health and Welfare, Republic of Korea. Examinations were conducted exclusively in certified hospitals that undergo periodic quality assessments by the NHIS. Trained nurses conducted standardized anthropometric assessments, including BMI and waist circumference, and blood pressure measurement on the day of National Health Screening. Information on sociodemographic characteristics, comorbidities, medication use, anthropometric measurements, and laboratory results was obtained from the health examination records. Lifestyle factors—alcohol consumption, smoking status, and physical activity—were assessed using structured self-reported questionnaires. Alcohol intake was classified by weekly consumption as none (0 g/week), mild (1–209 g/week), or heavy (≥210 g/week), as previously described.^14^ Smoking status was categorized into 3 groups based on use within the recent year. Physical activity was categorized according to the World Health Organization (WHO)-recommended, duration-based thresholds as none (0 min/week), intermediate (1–149 min/week of moderate or 1–74 min/week of vigorous activity), and ideal (≥150 min/week of moderate or ≥75/week minutes of vigorous activity).^15^ Household income was dichotomized as the lowest quintile vs. all others. Laboratory evaluations included complete blood count, fasting glucose, lipid profile, basic liver function, and renal function from the overnight-fasting blood samples at NHIS-certified hospitals with quality control. Serum glucose, lipid profiles, and creatinine were measured using enzymatic methods traceable to national reference standards. Diabetes was defined as a self-reported diagnosis or fasting glucose ≥126 mg/dL. Hypertension was defined as a self-reported diagnosis, use of antihypertensive medications, or measured systolic/diastolic blood pressure (SBP/DBP) ≥140/90 mmHg. Dyslipidemia was defined by any of the following: use of lipid-lowering medications, total cholesterol ≥200 mg/dL, low-density lipoprotein cholesterol (LDL-c) ≥160 mg/dL, or HDL-c <40 mg/dL in men and <50 mg/dL in women. Chronic kidney disease was defined as an estimated glomerular filtration rate of <60 mL/min/1.73 m^2^ calculated using the CKD-EPI equation. These operational definitions were consistent with those used in previous NHIS-based studies.^16^

The major cardiovascular risk factors analyzed in this study included BMI, SBP, non-HDL-c, smoking, and diabetes.^8^ BMI was categorized according to WHO recommendations for Asian populations^17^ as follows: underweight (<18.5 kg/m^2^), normal (18.5–22.9 kg/m^2^), overweight (23.0–24.9 kg/m^2^), obesity class I (25.0–29.9 kg/m^2^), and obesity class II (≥30.0 kg/m^2^). SBP was divided into five categories at 20 mmHg increments: <100, 100–119, 120–139, 140–159, and ≥160 mmHg. Non-HDL-c was classified with 30 mg/dL intervals up to ≥220 mg/dL. Smoking status was categorized as non-, ex-, or current smoker. Diabetic status was classified into 3 categories according to American Diabetes Association^18^: normoglycemia, prediabetes, and diabetes.

All measurements followed uniform national standards to ensure data comparability and reproducibility across sites. Details of the collected variables are summarized in Supplementary Table S1 and Supplementary Figures S1 and S2.

### Study outcomes and follow-up

Primary outcome was a composite of major cardiovascular events, including cardiovascular death, MI, and ischemic stroke. Mortality data were obtained from the Statistics Korea database, with causes of death classified using the Korean Standard Classification of Diseases and Causes of Death, based on ICD-10 codes. Cardiovascular death was defined as a death with ICD-10 codes I00–I99, verified by official death certificates. MI was defined as a hospitalization with ICD-10 codes I21 or I22. Ischemic stroke was identified using ICD-10 codes I63 or I64 during a hospitalization. All clinical events occurring more than 3 months after the index date were ascertained and included in the analysis to minimize potential reverse causation. Participants were followed until the occurrence of the study outcome or 31 December 2022, whichever came first.

### Statistical analysis

Baseline characteristics were compared across study groups using one-way analysis of variance for continuous variables (mean ± standard deviation or median [interquartile range (IQR)]) and chi-squared tests for categorical variables (frequencies and percentages). Incidence rates of ASCVD events were calculated as the number of events per 1000 person-years and stratified by age and sex. Associations between major risk factors and outcomes were evaluated using multivariable Cox proportional hazards regression models and were reported as hazard ratios (HRs) with 95% confidence intervals (CIs). We assessed the proportional hazards assumption for each Cox model using scaled Schoenfeld residuals. For each outcome (total ASCVD, cardiovascular death, MI, and ischemic stroke), both covariate-specific and global tests of proportionality were performed, applying a two-sided α = 0.05 threshold. Ties were handled using Efron's method. Analyses were further stratified by age and sex. Population-attributable fractions (PAFs) of the five major risk factors were calculated using the formula: p_e_ × (adjusted HR−1)/(p_e_ × (adjusted HR−1)+1), where p_e_ denotes the age-standardized prevalence of each risk factor.^19^ Age standardization was based on the mid-year 2000 Korean population using the direct standardization method^20^ and 95% CIs for PAF were estimated using Monte Carlo simulations. For subgroup analyses, adjusted HRs and corresponding PAFs were estimated separately for men and women at age 50 to derive sex-specific estimates. As a sensitivity analysis, competing risks of non-cardiovascular death were accounted for using Fine–Gray subdistribution hazard models, with incident ASCVD as the event of interest.^21^ All statistical tests were two-sided, with *p* values < 0.05 considered statistically significant. All *p* values are nominal and were not adjusted for multiple comparisons. Analyses were performed using SAS version 9.4 (SAS Institute, Cary, NC, USA).

### Ethics approval

The study protocol was approved by the Institutional Review Board of Seoul National University Hospital (IRB No. E-2402-025-1507) and complied with the Declaration of Helsinki. The requirement for written informed consent was waived by the Institutional Review Board due to the retrospective nature of the study and the use of anonymized administrative data from the Korean NHIS.

### Role of the funding source

The study had no external funding.

## Results

### Baseline characteristics

Baseline characteristics of the cohort comprising 6,249,852 Korean adults (mean age, 46.9 years; 52.2% men), are summarized in Table 1. The mean BMI in the total population was 23.6 kg/m^2^, with 18.8% of abdominal obesity. The mean SBP was 121.9 mmHg, and the mean non-HDL-c was 140.2 mg/dL. The prevalence of diabetes was 7.9%. Marked sex-based differences in cardiovascular risk profiles were observed. Current smoking (44.5% vs. 3.6%) and heavy alcohol consumption (13.7% vs. 1.1%) were far more prevalent in men. Men also exhibited a higher mean BMI (24.1 vs. 23.1 kg/m^2^) and greater abdominal obesity (21.2% vs. 16.2%), whereas underweight was more common among women (2.2% vs. 5.6%) (all *p* < 0.001). Regular physical activity was reported by 17.9% of the participants, more frequent in men (19.9%) than women (15.8%) (*p* < 0.001). Men had higher SBP (124.5 vs. 119.2 mmHg) and DBP (77.9 vs. 73.9 mmHg), along with a higher prevalence of hypertension (23.9% vs. 20.6%) (all *p* < 0.001). Similarly, fasting glucose levels (98.9 vs. 94.6 mg/dL) and diabetes prevalence (9.4% vs. 6.3%) were higher in men (both *p* < 0.001). Non-HDL-c levels were 142.5 mg/dL in men and 137.6 mg/dL in women, respectively.

**Table 1:** Baseline characteristics of the study population.

	Total (n = 6,249,852)	Men (n = 3,261,795)	Women (n = 2,988,057)	*p*
Clinical parameters				
Age, years	46.9 ± 13.7	45.8 ± 13.5	48.2 ± 13.9	<0.001
Age groups, %				<0.001
<40	1,902,403 (30.4)	1,169,606 (35.9)	732,797 (24.5)	<0.001
40–49	1,717,847 (27.5)	854,320 (26.2)	863,527 (28.9)	
50–64	1,894,300 (30.3)	894,469 (27.4)	999,831 (33.5)	
≥65	735,302 (11.8)	343,400 (10.5)	391,902 (13.1)	
Men, %	3,261,795 (52.2)			<0.001
BMI, kg/m^2^	23.6 ± 3.7	24.1 ± 3.3	23.1 ± 4.0	<0.001
BMI groups, %				<0.001
<18.5	238,814 (3.8)	72,269 (2.2)	166,545 (5.6)	<0.001
18.5–22.9	2,492,083 (39.9)	1,092,707 (33.5)	1,399,376 (46.8)	
23.0–24.9	1,530,969 (24.5)	882,703 (27.1)	648,266 (21.7)	
25.0–29.9	1,773,812 (28.4)	1,095,910 (33.6)	677,902 (22.7)	
≥30.0	214,174 (3.4)	118,206 (3.6)	95,968 (3.2)	
WC, cm	79.9 ± 9.1	83.5 ± 7.8	75.9 ± 8.8	<0.001
Abdominal obesity, %	1,176,285 (18.8)	691,155 (21.2)	485,130 (16.2)	<0.001
Income, Q1, %	1,137,818 (18.21)	471,369 (14.5)	666,449 (22.3)	<0.001
Smoking, %				<0.001
Non	3,827,796 (61.3)	1,001,024 (30.7)	2, 826,772 (94.6)	<0.001
Ex	863,923 (13.8)	808,659 (24.8)	55, 264 (1.9)	
Current	1,558,133 (24.9)	1,452,112 (44.5)	106,021 (3.6)	
Drinking, %				<0.001
Non	3,242,290 (51.9)	1,033,343 (31.7)	2,208,947 (73.9)	<0.001
Mild	2,465,511 (39.5)	1,746,928 (53.6)	718,583 (24.1)	
Heavy	480,930 (7.7)	447,957 (13.7)	32,973 (1.1)	
Regular exercise, %	1,121,280 (17.9)	649,643 (19.9)	471,637 (15.8)	<0.001
Comorbidities, %				
Diabetes	494,389 (7.9)	306,595 (9.4)	187,794 (6.3)	<0.001
Hypertension	1,395,180 (22.3)	780,990 (23.9)	614,190 (20.6)	<0.001
Dyslipidemia	3,484,447 (55.8)	1,691,997 (51.9)	1,792,450 (60.0)	<0.001
CKD	259,567 (4.2)	131,453 (4.0)	128,114 (4.3)	<0.001
Vital signs				
Systolic BP, mmHg	121.9 ± 15.0	124.5 ± 14.1	119.2 ± 15.5	<0.001
Diastolic BP, mmHg	76.0 ± 10.1	77.9 ± 9.7	73.9 ± 10.0	<0.001
Laboratory parameters				
Fasting glucose	96.9 ± 23.0	98.9 ± 25.2	94.6 ± 20.0	<0.001
Total cholesterol	195.4 ± 36.8	194.8 ± 36.2	196.1 ± 37.5	<0.001
Triglycerides	107 (74–159)	124 (85–184)	91 (65–133)	<0.001
HDL-c	55.2 ± 13.8	52.3 ± 12.9	58.5 ± 13.9	<0.001
LDL-c	114.2 ± 36.2	112.9 ± 36.2	115.7 ± 36.1	<0.001
Non-HDL-c	140.2 ± 37.2	142.5 ± 36.6	137.6 ± 37.7	<0.001
eGFR, mL/min/1.73 m^2^	93.5 ± 22.5	94.3 ± 24.1	92.7 ± 20.4	<0.001

Values are presented as mean ± standard deviation, median (interquartile ranges) for triglycerides, or numbers (%). Measurement unit of laboratory findings are mg/dL BMI, body mass index; BP, blood pressure; CKD, chronic kidney disease; eGFR, estimated glomerular filtration rate; HDL-c, high-density lipoprotein cholesterol; LDL-c, low-density lipoprotein cholesterol; Q, quintile; WC, waist circumference.

Within sex, age-specific baseline characteristics are detailed in Supplementary Table S2. The distribution of risk factors varied significantly across age and sex. Abdominal obesity increased with age from 17.3% to 25.8% in men, and in women, the increase was more pronounced—from 5.9% to 34.5%—in parallel with a progressive rise in BMI. Smoking prevalence declined with age in men but remained consistently low in women. Non-HDL-c increased progressively with age in women but peaked in midlife in men. Diabetes and hypertension were more frequently observed in older individuals in both sexes.

### Incidence and the risk of ASCVD

Over a median follow-up of 13.0 years (IQR, 12.2–13.3 years)—incident ASCVD events, including cardiovascular death, MI, and ischemic stroke—occurred in 279,093 participants (4.5%), corresponding to an overall incidence rate of 3.58 per 1000 person-years. ASCVD incidence increased progressively with age, from 0.59 to 15.48 per 1000 person-years (Table 2). This trend persisted across sex and age strata. Across all age groups <65 years, the incidence of ASCVD was more than twice as high in men compared to women; however, in those aged ≥65 years, the sex gap was attenuated (17.91 vs. 13.55 per 1000 person-years).

**Table 2:** The incidence rate of ASCVD events according to age and sex.

	Total			Men			Women		
	N	ASCVD Events (%)	IR per 1000 person-years	N	ASCVD Events (%)	IR per 1000 person-years	N	ASCVD Events (%)	IR per 1000 person-years
Age groups	6,249,852	279,093 (4.5)	3.58	3,261,795	167,544 (5.1)	4.14	2,988,057	111,549 (3.7)	2.97
<40	1,902,403	14,545 (0.8)	0.59	1,169,606	11,738 (1.0)	0.78	732,797	2 807 (0.4)	0.30
40–49	1,717,847	37,909 (2.2)	1.73	854,320	26,772 (3.1)	2.47	863,527	11,137 (1.3)	1.01
50–64	1,894,300	102,383 (5.4)	4.35	894,469	65,287 (7.3)	5.98	999,831	37,096 (3.7)	2.94
≥65	735,302	124,256 (16.9)	15.48	343,400	63,747 (18.6)	17.91	391,902	60,509 (15.4)	13.55

ASCVD, atherosclerotic cardiovascular disease; IR, incidence rate; N, number.

**Fig. 2:**
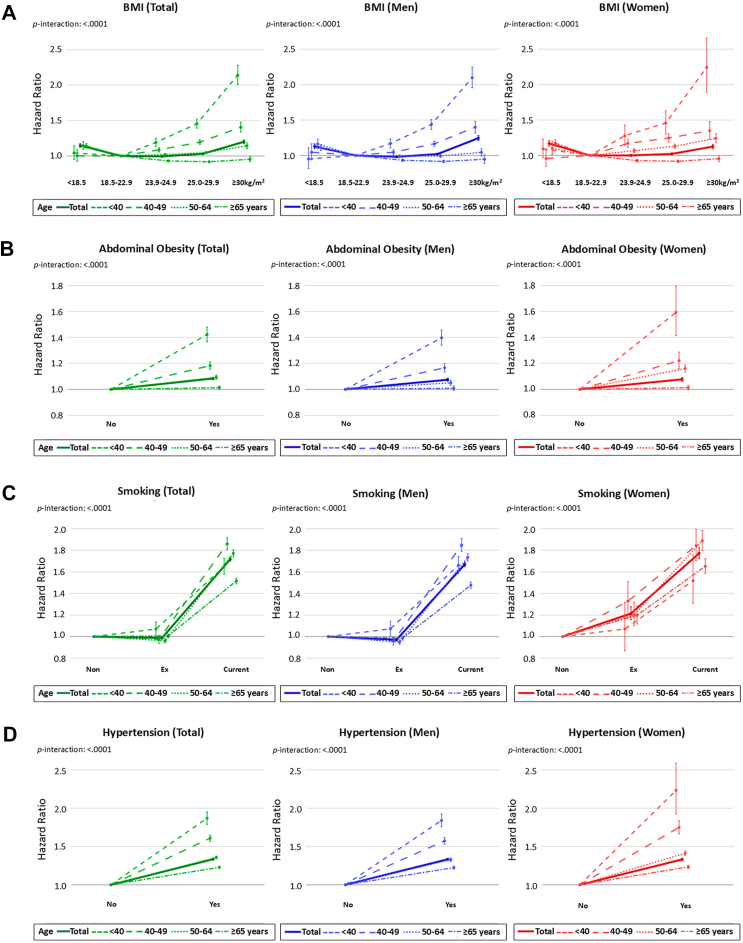

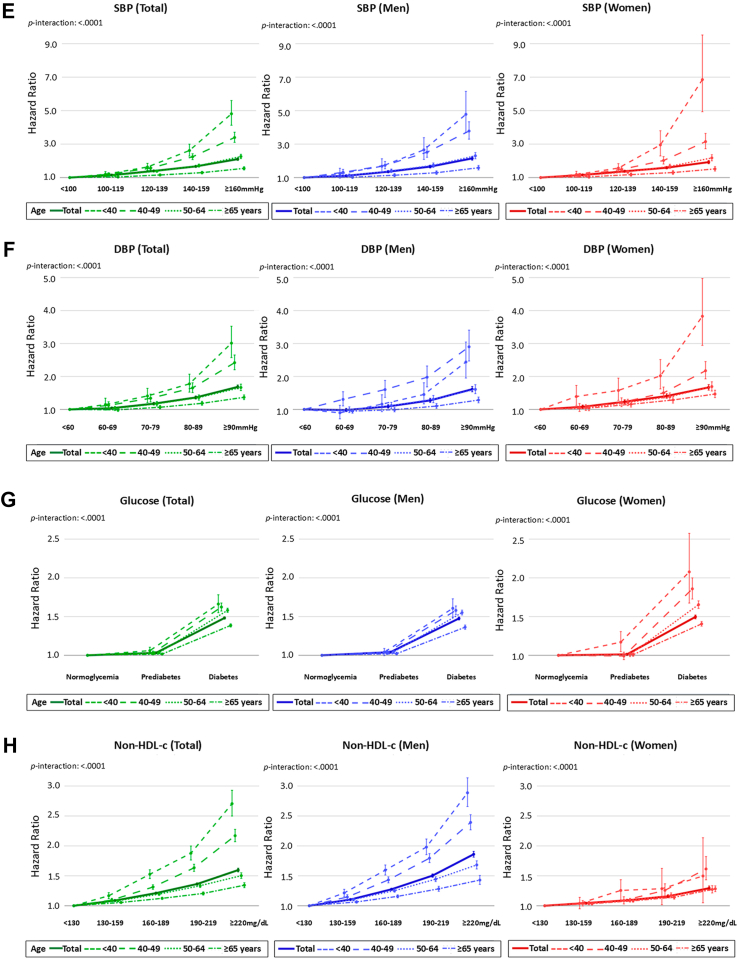
**Associations of individual cardiovascular risk factors with ASCVD risk, stratified by age and sex**. Adjusted HRs for incident ASCVD according to modifiable risk factors were presented for the total population (green), men (blue), and women (red): (A) BMI, (B) abdominal obesity, (C) smoking, (D) hypertension, (E) SBP, (F) DBP, (G) glucose levels, and (H) non-HDL-c levels. The contribution of each risk factor was further compared across different age groups. BMI, body mass index; DBP, diastolic blood pressure; HDL-c, high-density lipoprotein cholesterol; HR, hazard ratio; SBP, systolic blood pressure.

In the total population, a shallow U-shaped association between BMI and ASCVD risk was observed (Fig. 2A). Both underweight (BMI <18.5 kg/m^2^) and severe obesity (BMI ≥30.0 kg/m^2^) were associated with a 15–20% increased risk compared to normal BMI (18.5–23.0 kg/m^2^). This pattern was also observed in sex-stratified analyses. Age-stratified analyses revealed distinct patterns: in individuals aged <50 years, ASCVD risk increased steadily with BMI above 23.0 kg/m^2^, whereas underweight was not associated with excess risk, particularly with a steeper increment in younger age group. By contrast, in those aged ≥50 years, the highest risk was observed in the underweight group. Notably, in those aged ≥65 years, overweight (23.0–24.9 kg/m^2^) and obesity (≥25.0 kg/m^2^) appeared to be protective, with HRs <1.00. A similar age-dependent attenuation was observed for abdominal obesity (Fig. 2B). Current smoking exerted a strong association with ASCVD risk in all age and sex groups, while the impact of ex-smoking was evident only in women aged ≥40 years (Fig. 2C). Hypertension conferred a 34% increased risk (HR 1.34, 95% CI 1.33–1.35), with a dose-dependent manner observed for both SBP and DBP. The association was more strongly driven by SBP, showing steeper risk increases across SBP categories (Fig. 2D–**F**). Glucose dysregulation was another major contributor. Compared to normoglycemia, ASCVD risk was highest in those with diabetes (HR 1.48, 95% CI 1.47–1.50), followed by prediabetes (HR 1.03, 95% CI 1.02–1.04) (Fig. 2G). Non-HDL-c showed a stepwise association with ASCVD risk, peaking at ≥220 mg/dL (HR 1.60, 95% CI 1.57–1.63) (Fig. 2H). LDL-c and triglycerides exhibited similar trends, although the risk increase associated with LDL-c was more modest, with a flatter trajectory than other lipid parameters. These associations for BP, glucose and lipid parameters were consistent across sexes, with stronger effects in younger age groups. Interaction analyses indicated significant age-by-risk factor interactions (all *p* interaction <0.001). Detailed estimates for the associations shown in Fig. 2 across age and sex strata, are presented in Supplementary Table S3. Furthermore, the association between each risk factor and individual outcomes—cardiovascular death, MI, and ischemic stroke—are provided in Supplementary Tables S4–S6.

### Preventable ASCVD

**Fig. 3:**
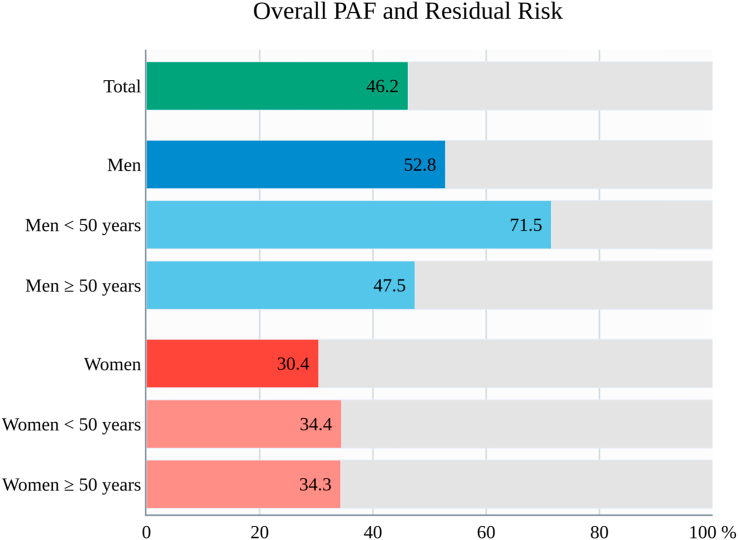
**Aggregated PAFs for ASCVD according to age and sex**. The overall PAF for ASCVD attributable to five major modifiable risk factors was 46.2% in the Korean population. Notably, the attributable burden varied significantly by age and sex groups, with the highest PAF observed in men younger than 50 years. PAF, population-attributable fraction, other abbreviations as Fig. 1.

The aggregated PAFs for five major modifiable risk factors—SBP, smoking, non-HDL-c, diabetes, and BMI—were calculated across age and sex groups (Fig. 3, Table 3). In the total population, these risk factors collectively accounted for a PAF of 46.2% (95% CI 43.2–49.1) of ASCVD events, indicating that nearly half of ASCVD burden could potentially be preventable through risk factor modification. Among individual risk factors, SBP contributed the most to ASCVD development. The remaining 53.8% (95% CI 50.9–56.8) represents residual risk not explained by these five risk factors, implying a potential role of other unmeasured or non-traditional determinants. Men had a higher aggregated PAF than women (52.8% [95% CI 48.8–56.6] vs. 30.4% [95% CI 25.6–35.0]). In men, the PAF was markedly greater in those aged <50 years (71.5%), compared to those ≥50 years (47.5%). In men aged <50 years, SBP alone explained 48.6% of the preventable burden, followed by smoking (29.4%), which had a far greater impact than observed in women. While a similar age-related trend was observed in women, the difference by age appeared less marked. SBP remained the leading contributor to ASCVD prevention in women, with comparable PAFs between younger (24.5%) and older (26.4%) age groups. The contribution of obesity and diabetes were modest, but obesity had a greater impact on younger age groups, while diabetes had a greater impact on middle-aged and older age groups. Detailed estimates of aggregated PAFs are provided in Supplementary Table S7. Results from the competing risk models were consistent with the main analyses (Supplementary Table S8). When PAFs were estimated separately for each cardiovascular outcome, distinct patterns were observed (Supplementary Tables S9–S11). Elevated SBP was the single largest contributor to the overall ASCVD burden. However, the relative contributions of individual risk factors differed by outcome type, showing an SBP-driven ischemic stroke pattern and an MI pattern jointly driven by atherogenic lipids, SBP, and smoking.

**Table 3:** PAFs of five major modifiable risk factors for ASCVD according to age and sex.

	Total	Men			Women		
		All	<50 years old	≥50 years old	All	<50 years old	≥50 years old
Aggregated PAF, % (95% CI)	46.2 (43.2–49.1)	52.8 (48.8–56.6)	71.5 (65.3–76.8)	47.5 (42.7–51.9)	30.4 (25.6–35.0)	34.4 (25.2–42.7)	34.3 (28.1–40.1)
Residual Risk, % (95% CI)	53.8 (50.9–56.8)	47.2 (43.4–51.2)	28.5 (23.2–34.7)	52.5 (48.1–57.3)	69.6 (65.0–74.4)	65.6 (57.3–74.8)	65.7 (59.9–71.9)
SBP, mmHg	27.7 (22.6–28.1)	27.4 (20.9–29.4)	48.6 (33.2–49.8)	26.4 (19.3–28.9)	23.3 (17.9–25.3)	24.5 (16.1–29.1)	26.4 (19.4–28.7)
Smoking	16.3 (16.0–16.7)	23.7 (23.2–24.2)	29.4 (28.2–30.6)	16.4 (15.9–16.9)	3.7 (3.3–4.0)	4.4 (3.4–5.3)	2.2 (2.0–2.4)
Non-HDL-c, mg/dL	9.0 (8.1–9.3)	12.8 (11.4–13.1)	21.0 (17.6–21.2)	10.5 (9.1–11.2)	3.5 (2.6–4.2)	3.0 (1.2–4.7)	6.2 (4.7–7.4)
Diabetes	3.5 (3.3–3.8)	4.5 (4.1–4.8)	3.1 (2.4–3.8)	7.8 (7.1–8.4)	2.4 (2.1–2.7)	1.6 (0.9–2.4)	5.7 (5.0–6.3)
BMI	2.1 (1.7–2.5)	1.4 (0.8–1.9)	11.2 (10.1–12.3)	0.0 (0.0–0.0)	2.2 (1.7–2.7)	7.1 (5.8–8.3)	0.1 (−0.6–0.8)

CI, confidence interval; PAF, population-attributable fraction; other abbreviation as Tables 1 and 2.

## Discussion

In this large, nationwide population-based cohort study of 6,249,852 adults without preexisting ASCVD, we identified the following key findings: 1) The burden and distribution of ASCVD risk factors varied substantially by age and sex; 2) five major modifiable risk factors jointly explained 46.6% of ASCVD events, with SBP contributing the most; 3) the aggregated PAFs of these risk factors were higher in men than in women, particularly in men aged <50 years (PAF 71.5%), where SBP and smoking were the dominant contributors to preventable ASCVD burden; 4) in women, SBP remained the most impactful risk factor across all ages, with less pronounced age-related differences, and other risk factors had comparatively limited preventive impact. Our results highlight the heterogeneity of preventable ASCVD risk and the need for targeted prevention strategies across demographic subgroups. Specifically, intensive control of SBP and smoking in younger men and consistent SBP management through the life course in women may yield the greatest preventive benefits.

Previous studies, including the Framingham Offspring^22^ and Global Cardiovascular Risk Consortium cohorts,^8^ have demonstrated marked differences in cardiovascular risk factor profiles and consequent ASCVD incidence by age, sex, geographic regions, and period.^6^, ^7^, ^8^^,^^22^, ^23^, ^24^ Although age has consistently been recognized as the strongest contributor to ASCVD risk and men are generally more susceptible to ASCVD than women,^22^ the relative contributions of individual risk factors vary considerably across populations.^8^ Furthermore, evidence from Asian data remain limited. Our study addresses this gap by providing contemporary, population-based data from a large, nationally representative Korean cohort, in which ASCVD patterns and risk factor contributions differ notably from those in the other global populations. In this study, the incidence rate of ASCVD was estimated at 4.14 per 1000 person-years in men and 2.97 in women, with less obese, lower diabetes prevalence, and higher men's smoking rates than Western population. These results were broadly consistent with the Asian data reported in the global cohort study.^8^ However, it was noticeable that our cohort had a mean age nearly 7 years younger than Asian cohorts included in the global study (46.9 vs. 54.0 years). This underscores the necessity of initiating prevention earlier in life, even within East Asian populations, particularly in Korea.

The age- and sex-stratified PAFs in our study provide further insights into the heterogeneity in ASCVD attribution. The aggregated PAF was higher in men (52.8%) than in women (30.4%), with the most striking difference observed in those aged <50 years (71.5% vs. 36.2%). Compared with prior global studies such as INTERHEART^6^ and PURE,^7^ the PAFs observed in our analysis were relatively smaller, largely driven by methodological and population-specific differences. Whereas INTERHEART and PURE included nine to ten risk factors across heterogeneous, often high-risk populations, our study focused on five conventional risk factors in the nationally representative Korean cohort. The smaller number of risk factors assessed, lower baseline risk, lack of ethnic diversity, and limited exposure variability likely contributed to smaller aggregated PAFs. Nonetheless, these population-specific estimates provide meaningful insight, as the relative contribution of individual risk factors to ASCVD differs across populations and ethnic groups. Because most traditional risk-prediction models using 5 key factors were developed in Western cohorts, they tend to overestimate ASCVD risk in East Asian populations.^11^ Our findings therefore emphasize the importance of ethnicity- and region-specific prevention strategies tailored to local epidemiology and risk distributions. Additionally, our findings align with global estimates for men from the Global Cardiovascular Risk Consortium, which reported an aggregated PAF of 52.6%, but contrast markedly with the female PAF of 57.2%.^8^ The relatively lower PAF in Korean women likely reflects distinctive behavioral and cultural characteristics. According to the recent report from the Korean Society for the Study of Obesity, the obesity rate in Korean women (27.7%) is merely half that of men (49.6%).^25^ Additionally, smoking among women remains socially stigmatized, with a low prevalence of around 5%,^26^ which helps explain why the PAF for smoking in women was only one-sixth of that in men. This prevailing risk profile pattern of Korean women may be partially responsible for the attenuated impact of major modifiable risk factors on ASCVD risk. Despite such favorable risk profile, Korean women may face increasing ASCVD risk due to emerging non-traditional determinants such as hypertensive disorders of pregnancy, gestational diabetes,^27^ and psychosocial stress,^28^ which have recently been emphasized as major considerations. These call for the need to expand current risk assessment frameworks to incorporate female-specific, culturally relevant, and psychosocial factors tailored to each national context. Among Korean men, especially those <50 years, the preventable ASCVD burden was markedly higher, driven primarily by SBP and smoking. Younger men showed a steeper risk increase across the spectrum of risk factor severity, suggesting greater susceptibility to metabolic insults in early adulthood. This supports previous evidence that early-life exposure to cardiovascular risk factors accelerates vascular ageing and long-term risk accumulation.^12^^,^^29^ Given the relatively younger age of our cohort, early-life interventions are likely to be critical for optimizing future cardiovascular health in Korea.

In terms of contribution of individual risk factors, our data corroborate the dominant role of hypertension in ASCVD risk. Hypertension is known to account for up to 13.5% of global deaths and is regarded as a single most influential factor for ASCVD.^30^ Current guidelines have shifted toward more intensive BP control to mitigate long-term ASCVD risk,^31^^,^^32^ as supported by the SPRINT trial showing benefit with SBP <120 mmHg.^33^ In line with this paradigm, our data reinforce that SBP control offers the greatest preventive potential, irrespective of sex. Notably, the PAF for SBP was remarkably high in young men aged <50 years (48.6%), indicating that hypertension in this group is a powerful yet under-recognized contributor to ASCVD burden. However, awareness and treatment of hypertension have been suboptimal in young Korean adults over the past decade,^34^ suggesting that a large proportion of preventable risk remains unaddressed. Bridging this gap through early detection, lifestyle modification, and timely initiation of pharmacologic therapy may yield substantial long-term benefits. Accordingly, to optimize cardiovascular health, it is imperative to implement targeted nation-level strategies encouraging young adults to recognize and treat hypertension properly. While we confirmed a robust and continuous association between non-HDL-c and ASCVD, consistent with previous studies,^35^ its attributable impact was modest. Interestingly, we observed divergent age-related patterns between sexes. In men, the PAF of non-HDL-c halved after the age of 50, whereas in women, it more than doubled after age 50, following minimal contribution at younger ages. This opposing trend may reflect differing age-related lipid trajectories: In men, lipid levels improve after the age of 60, but in women, dyslipidemia continues to rise with age, peaking at approximately 75% in their 70s.^36^ A more nuanced interpretation suggests that the relatively higher proportion of ischemic stroke among clinical events (58%) may partly explain the observed difference in PAFs between SBP and non-HDL-c across all age and sex groups. This pattern likely mirrors the epidemiologic characteristics of East Asia, where cerebrovascular events are more prevalent,^37^ as well as the distinctive risk profile of the Korean population, in which hypertension exerts a particularly strong influence.^38^ This interpretation was further supported by the outcome-specific analysis (Supplementary Tables S9–S11), where elevated SBP remained the predominant contributor to the total ASCVD burden, but its impact was especially pronounced for ischemic stroke. BMI and diabetes showed comparatively limited PAFs in this populations, suggesting potential differences in population-specific characteristics compared to prior reports.^8^ Of note, the association between BMI and ASCVD risk differed by age group: in younger and middle-aged adults, higher BMI was associated with increased ASCVD risk, whereas in adults aged ≥65 years, higher BMI (≥23.0 kg/m^2^) showed an inverse association with ASCVD risk, consistent with the prior reports.^39^ The inverse association in the elderly should be interpreted cautiously, as it may be influenced by reverse causation and selective survival. This age-dependent pattern may partly explain the modest overall contribution of BMI and diabetes to ASCVD in the Korean population. Nevertheless, in individuals aged <50 years, the PAFs of BMI were 11.2% in men and 7.1% in women, indicating that obesity remains a meaningful and preventable contributor to ASCVD burden in younger populations. These findings emphasize the importance of maintaining healthy body weight and metabolic control early in life, despite the attenuated associations observed in older age.

Taken together, we carry important implications for both clinical practice and public health. For clinicians, greater emphasis on systematic assessment and optimal control of SBP and smoking in younger men appears warranted, while risk evaluation in women should increasingly incorporate non-traditional factors. From a public health perspective, earlier interventions—through routine BP screening, smoking cessation initiatives, and workplace-based lifestyle programs—may offer substantial benefits in reducing the future ASCVD burden. Moreover, as over half of the total ASCVD risk remains unexplained by conventional factors, future studies should explore emerging determinants such as psychosocial stress, sleep, and environmental exposures to inform more comprehensive prevention strategies in Korea.

### Study limitations

Despite the important insights provided by this study, certain limitations warrant consideration. As an observational study, causal inferences are limited. Because all measurements were obtained at a single baseline examination, intra-individual biological variability or cumulative effect could not be fully accounted for. Nevertheless, this concern is mitigated by the rigorous standardization of the National Health Screening program, conducted in certified hospitals under strict quality-controls mandated by the NHIS. Despite the high participation rate, selection bias cannot be excluded because direct comparisons between participants and non-participants were not feasible. However, as the National Health Screening Program is provided free of charge, including for individuals covered by the Medical Aid program—Korea's government-funded health coverage for socioeconomically disadvantaged populations—socioeconomic barriers to participation may be attenuated. Non-participation is more common among individuals with severe baseline health conditions that limit engagement in preventive services, and as PAFs depend on the distribution of modifiable risk factors, the potential impact of selection bias on the interpretation and generalizability of our findings should be acknowledged. In addition, as participants with missing data (2.8%) were excluded, an additional source of selection bias cannot be entirely ruled out; however, given the small proportion of excluded participants, the potential impact on our findings is likely limited. Despite rigorous adjustments, residual confounding may persist, possibly explaining the large unexplained residual risk. ASCVD outcomes were ascertained through administrative claims, which may carry misclassification or underreporting bias. Treatment data were limited, in particular, regarding lipid-lowering therapies, which might have affected the observed clinical outcomes. This may explain the minor association between LDL-c levels and ASCVD risk. In addition, initiation of preventive therapies during follow-up could have attenuated subsequent ASCVD incidence, leading to an underestimation of the true PAFs for major risk factors. This potential limitation is inherent to PAF analyses based on baseline exposures, as similarly acknowledged in the Global Cardiovascular Risk Consortium study.^8^ Therefore, the PAFs in our study likely represent conservative estimates of the true impact of elevated SBP and non-HDL-c on ASCVD risk. Moreover, as PAFs were estimated using HRs derived from Cox proportional hazards models based on baseline exposures, these primarily reflect differences in instantaneous risk. When evaluated using cumulative incidence over longer time periods, PAFs may be attenuated, as modification of risk factors and initiation of preventive therapies may delay rather than entirely prevent ASCVD events. Lastly, generalizability may be restricted to the Korean population. However, our findings offer valuable insights given the paucity of recent large-scale cohort data in East Asia.

### Conclusions

In this nationwide cohort study, fewer than half of ASCVD events were attributable to major modifiable risk factors, with distinct age- and sex-specific variations. Our findings support the need for two-pronged preventive strategies in Korea: intensive control of SBP and smoking focused on young men, and sex-specific approaches in women that consider non-traditional and life-course factors. Such individualized yet population-wide approaches, informed by demographic heterogeneity, may yield the greatest reductions in ASCVD burden and optimize cardiovascular health at the national level.

## Contributors

All authors had final responsibility for the decision to submit the manuscript. Sangjun Lee and Soyeoun Kim had full access to the data and verified the underlying data. Authors’ contributions were as follows: Conception and design: H.L., T.M.R., and S.Y.C.; data acquisition: S.L. and S.K.; data analysis and interpretation: H.L., E.K., T.M.R., J.H.P., S.L., S.K., and S.Y.C.; statistical analysis: S.L. and S.K.; drafting and finalizing the paper: H.L. and E.K.; figures: H.L., S.L., and S.K.; critical revision of the paper for important intellectual content: T.M.R., J.H.P., J.Y.K., and S.Y.C.

## Data sharing statement

The data used in this study are owned by the Korean NHIS and cannot be shared publicly due to legal and institutional restrictions. By national policy, raw data cannot be moved from the NHIS secure analysis center, and analyses may only be performed on-site by pre-authorized researchers for approved topics within a designated time period. Only aggregate results may be extracted after review by the NHIS. Researchers interested in accessing the data may contact the corresponding author to discuss potential collaboration under the NHIS data use policies.

## Declaration of interests

The authors have declared no conflict of interest.
