# Supplementary material for: Age- and sex-specific attributable impact of modifiable risk factors on atherosclerotic cardiovascular disease in Korea: a nationwide population-based cohort study
Source: Lancet Reg Health West Pac. 2026 Apr 3;69:101844. doi: 10.1016/j.lanwpc.2026.101844 (PMC13085072; doi:10.1016/j.lanwpc.2026.101844)
Supplement: Supplementary Figure S1 [file mmc1.pdf]

<A front cover>

The guidance of a health screening program

Health Screening Program Information_English
Health Screening Program Information_Chinese
Health Screening Program Information_Japanese
Health Screening Program Information_Vietnamese
Health Screening Program Information_Philippines(Tagalog)
Health Screening Program Information_Thai
Health Screening Program Information_O‘zbektili
Health Screening Program Information_Mongolian
Health Screening Program Information_Cambodian
Health Screening Program Information_Nepali
Health Screening Program Information_Indonesian
Health Screening Program Information_Sinhala
Health Screening Program Information_Russian
Health Screening Program Information_Burmese

“We do little things to keep you happy; the National Health Insurance Service is with you.”

Ministry of Health & Welfare,
National Health Insurance Service

【The guidance of health screening program】

The National Health Insurance Service (NHIS) provides medical care assistance to its recipients and health insurance to its subscribers through health checkups.

The purpose of a health checkup is disease prevention or early detection to prevent its further advancement, and appropriate measures can be taken to maintain good health.

♠ **The general health screening** aims for the prevention and early detection of cardiovascular and cerebrovascular disease, such as hypertension and diabetes. The screening targets individuals above 20 years old and it shall be conducted once every 2 years. However, there are no age limits for those who are local members with a householder and working members and it shall be conducted once every year for those who are working members with nonoffice work experience.

♠ **An oral checkup** is conducted for the early detection of oral diseases, such as dental caries, periodontal diseases, and so on, for the subjects of the general health checkup.

♠ **The cancer screening** focuses on the six major cancers that have high occurrence rates that can be detected early through simple methods and are treatable. The cancer screening types and subjects include stomach and breast cancer for individuals over 40, colon cancer for individuals over 50, cervical cancer for females over 20, liver cancer for individuals over 40 with high risk, and lung cancer for individuals from 54 to 74 who smoked a pack a day for over 30 years. The screening intervals are once every two years for stomach, breast, cervical and lung cancer; once a year for colon cancer and twice a year for liver cancer.

♠ **The infant health screenings** include the required examinations for normal healthy growth, including growth and development assessments and infant care consultations reflecting health education. The qualifying candidates may obtain up to 10 screenings, for children under 6 years old at 4, 9, 18, 30, 42, 54, and 66 months of age, including a dental exam.

【The cost of health screening】

♠ The costs of general health screenings and the infants' health screening shall be borne fully by the corporation. Therefore, no extra cost shall be borne by the examinee during the examination.
♠ Regarding the cancer screening, 90% of the screening cost shall be borne by the corporation and the remaining 10% shall be borne by the examinee. However, regarding the cervical cancer and colon cancer screening, it shall be borne fully by the corporation.
♠ Regarding the screening cost of the national cancer screening subject (write on the screening table to the standard of insurance premium), it shall be borne by the country for 10% of the cost of burden for the examinee, so no cost shall be borne by the examinee.

【The procedure of health screening】

♠ Check health screening subject
Check the screening event and screening item by the screening confirmation dispatched (residence or working place) to the screening subject from the corporation.
♠ Appointment and visiting the health screening agency
You can schedule a health screening test in ANY AUTHORIZED hospital regardless of the area you are residing in. Please bring your ID and Medical Checkup Card on your appointment.
♠ Notification of the screening result
The screening result shall be mailed to the contact information of the examinee by the screening agency in the place where the screening took place, and a confirmation checkup shall be conducted on those suspected with disease based on the general health checkup results.

【Precautions during the health screening】

♠ You have to fast after 9:00 p.m. on the day before the health screening
- Should undergo fasting for at least 8 hours.
- The screening result may not be accurate if subject is not in a fasting state.
♠ Please avoid screening during menstruation (about 2–3 days before and after menstruation).
♠ The health screening can be received only with fixed numbers and in case of receiving in excess of the numbers, the screening cost shall be returned.

<Back side>

【9 tips for preventing cardiovascular and cerebrovascular disease】

<Reference: Ministry of Health & Welfare>

1_Discontinue smoking.
2_Reduce the consumption of alcohol to 1 or 2 glasses per day.
3_Maintain a healthy diet and eat plenty of vegetables and fish.
4_Try to exercise more than 30 minutes a day whenever possible.
5_Maintain proper weight. Regularly take waist measurements.
6_Find ways to reduce stress and enjoy life.
7_Measure blood pressure periodically and check blood sugar and cholesterol.
8_Reduce and cure hypertension, diabetes, and hyperlipidemia.
9_Note the symptoms of stroke and transmural myocardial infarction, and in the event of an emergency, seek immediate medical attention.

【The Web page of HNIS with foreign languages】

☞ **http://www.nhis.or.kr/english/**
☞ **http://www.nhis.or.kr/japanese/**
☞ **http://www.nhis.or.kr/chinese/**
☞ **http://www.nhis.or.kr/vietnamese/**

【English call center of NHIS】

☎ **033-811-2000**

【Sign language questionnaire and health checkup information】

Health iN homepage
