# Supplementary material for: Age- and sex-specific attributable impact of modifiable risk factors on atherosclerotic cardiovascular disease in Korea: a nationwide population-based cohort study
Source: Lancet Reg Health West Pac. 2026 Apr 3;69:101844. doi: 10.1016/j.lanwpc.2026.101844 (PMC13085072; doi:10.1016/j.lanwpc.2026.101844)
Supplement: Supplementary Figure S2 [file mmc2.pdf]

Health checkup questionnaire

※ Examinees must complete the questionnaire to receive the results of the cardiovascular disease risk assessment.

Last Name		Resident Reg. No..		Telephone	Home	
Given Name					Mobile phone	
Current address				E-mail		
				How to receive a health checkup report		□ Post □ E-mail □ Mobile

※ Please answer all the questions below.

Medical history (disease history, family history)

1. Have you ever been diagnosed by a doctor with any of the following diseases or are you currently taking any medication?

	Diagnosis		Medication therapy	
Brain stroke (paralysis)	Yes	No	Yes	No
Cardiac infarction/angina	Yes	No	Yes	No
High blood pressure	Yes	No	Yes	No
Diabetes	Yes	No	Yes	No
Dyslipidemia	Yes	No	Yes	No
Tuberculosis	Yes	No	Yes	No
Depression	Yes	No	Yes	No
Early Psychosis	Yes	No	Yes	No
Hepatitis C	Yes	No	Yes	No
Others (including cancer)	Yes	No	Yes	No

2. Has anyone in your family died from or gotten any of the following diseases?

Brain stroke (paralysis)	Yes	No
Cardiac infarction/angina	Yes	No
High blood pressure	Yes	No
Diabetes	Yes	No
Others (including cancer)	Yes	No

3. Are you a Hepatitis B virus antigen carrier?
① Yes ② No ③ No idea

Smoking

4. Have you ever smoked more than 5 packs of cigarettes (100 cigarettes) in your lifetime?
① No
② Yes. (☞ Go to Question 4-1)

4-1. Do you smoke cigarettes now?

① I do	A total of ____ years	An average of _______ cigarettes a day	
② I used to but not anymore	A total of ____ years	Used to smoke _______ cigarettes a day on average	____ years since I quitted

Drinking

※ In the past one year

5. How often do you have drinks containing alcohol? (Select one)
① (　　) times per week ② (　　) times per month
③ (　　) times per year
④ I don't drink alcohol.

5-1. How many drinks containing alcohol do you have on a typical day when you are drinking?
* Choose one among the glass, bottle, can, or cc (you can choose more than one for liquor types; choose a similar type for other liquor types that are not indicated)

Type of liquor	Glass	Bottle	Can	cc
Soju				
Beer				
Hard liquor				
Makgeolli (rice wine)				
Wine				

5-2. What is the largest amount of drinks containing alcohol that you have ever had in one day?
* Choose one among the glass, bottle, can, or cc (you can choose more than one for liquor types, choose a similar type for other liquor types that are not indicated)

Type of liquor	Glass	Bottle	Can	cc
Soju				
Beer				
Hard liquor				
Makgeolli (rice wine)				
Wine				

Exercising

6-1. How often do you do high intensity exercise (making you short of breath) per week?
(　　) days per week
* Examples of high intensity exercise> Running, aerobics, fast bicycling, construction labor, carrying items using stairs, etc.

6-2. How long do you do high intensity exercise (making you short of breath) per day?
(　　) hours (　　) minutes per day

7-1. How often do you do moderate intensity exercise (making you slightly short of breath) per week?
(　　) days per week
* Exclude exercise you have already written in Question 8
* Examples of moderate intensity exercise> Power walking, doubles tennis games, cycling at normal speed, carrying light items, cleaning, etc.

7-2. How long do you do moderate intensity exercise (making you slightly short of breath) per day?
(　　) hours (　　) minutes per day

8. How many days did you do weight training such as push-ups, sit-ups, dumbbell exercises, weight lifting, or horizontal bar exercise in the last one week?(　　) days per week

Additional health checkup questionnaires

Last Name		Resident Reg. No.	
Given Name			

※ Please fill out this questionnaire if it is applicable to you.

Functional assessment of elderly **(66, 70, and 80 years of age)**

1. Do you receive inoculations with influenza vaccine every year?
① Yes ② No

2. Have you received vaccinations against pneumonia?
① Yes ② No

3. The following questions are about your ability to perform activities of daily living
Please read and answer the questions below.

1) If someone sets the table for your meal, you can eat by yourself without any help.
① Yes ② No

2) Can you put on your clothes without any help?
① Yes ② No

3) Can you go to the toilet by yourself?
① Yes ② No

4) When you take a bath or a shower, can you wash by yourself?
① Yes ② No

5) Can you prepare your meals?
① Yes ② No

6) Can you go to places that are of walking distance, such as a store, clinic, neighbor, or any public offices, by yourself?
① Yes ② No

4. About fall injury: Have you fell down during the last 6 months?
① Yes ② No

5. Urinary function: Do you have any difficulty in urinating or in holding your urine?
① Yes ② No
