# Supplementary material for: Age- and sex-specific attributable impact of modifiable risk factors on atherosclerotic cardiovascular disease in Korea: a nationwide population-based cohort study
Source: Lancet Reg Health West Pac. 2026 Apr 3;69:101844. doi: 10.1016/j.lanwpc.2026.101844 (PMC13085072; doi:10.1016/j.lanwpc.2026.101844)
Supplement: Supplementary Tables [file mmc3.docx]

**Supplemental Tables**

**Supplementary Table 1.** Variables and Data Items Collected in the NHIS Health Screening Program

**Supplementary Table 2.** Baseline characteristics of the study population according to age subgroups

**Supplementary Table 3.** Associations of individual risk factors with ASCVD risk by age and sex

**Supplementary Table 4.** Associations of individual risk factors with CV death risk by age and sex

**Supplementary Table 5.** Associations of individual risk factors with MI risk by age and sex

**Supplementary Table 6.** Associations of individual risk factors with ischemic stroke risk by age and sex

**Supplementary Table 7.** Detailed estimates of aggregated PAFs

**Supplementary Table 8.** Sensitivity analysis using Fine–Gray competing risk models for ASCVD

**Supplementary Table 9.** PAFs of five major modifiable risk factors for CV death

**Supplementary Table 10.** PAFs of five major modifiable risk factors for MI

**Supplementary Table 11.** PAFs of five major modifiable risk factors for Ischemic stroke

**Supplementary Table 1. Variables and Data Items Collected in the NHIS Health Screening Program**

|  | **Code** | **Explanation** |
| --- | --- | --- |
| **The general health examination DB** | HCHK_YEAR | Year of examination |
|  | PERSON_ID | Personal identification number |
|  | YKIHO_GUBUN_CD | Code for the type of health screening institution |
|  | HEIGHT | Height |
|  | WEIGHT | Weight |
|  | WAIST | Waist circumference |
|  | BP_HIGH | Systolic blood pressure |
|  | BP_LWST | Diastolic blood pressure |
|  | BLDS | Fasting blood glucose |
|  | TOT_CHOLE | Total cholesterol |
|  | TRIGLYCERIDE | Triglyceride |
|  | HDL_CHOLE | HDL cholesterol |
|  | LDL_CHOLE | LDL cholesterol |
|  | HMG | Hemoglobin |
|  | OLIG_PROTE_CD | Urine protein |
|  | CREATININE | Serum creatinine |
|  | SGOT_AST | AST (SGOT) |
|  | SGPT_ALT | ALT (SGPT) |
|  | GAMMA_GTP | Gamma GTP |
|  | HCHK_APOP_PMH_YN | Personal history of stroke |
|  | HCHK_HDISE_PMH_YN | Personal history of cardiac disease (infarction/angina) |
|  | HCHK_HPRTS_PMH_YN | Personal history of hypertension |
|  | HCHK_DIABML_PMH_YN | Personal history of diabetes |
|  | HCHK_HPLPDM_PMH_YN | Personal history of dyslipidemia |
|  | HCHK_PHSS_PMH_YN | Personal history of tuberculosis |
|  | HCHK_ETCDSE_PMH_YN | Personal history of other diseases (including cancer) |
|  | MED_APOP_PMH_YN | Medication of stroke |
|  | MED_HDISE_PMH_YN | Medication of cardiac disease (infarction/angina) |
|  | MED_HPRTS_PMH_YN | Medication of hypertension |
|  | MED_HPRTS_PMH_YN | Medication of diabetes |
|  | MED_DIABML_PMH_YN | Medication of dyslipidemia |
|  | MED_HPLPDM_PMH_YN | Medication of tuberculosis |
|  | MED_HPRTS_PMH_YN | Medication of other diseases (including cancer) |
|  | FMLY_HPRTS_PATIEN_YN | Family history of hypertension |
|  | FMLY_APOP_PATIEN_YN | Family history of stroke |
|  | FMLY_HDISE_PATIEN_YN | Family history of cardiac disease |
|  | FMLY_DIABML_PATIEN_YN | Family history of diabetes |
|  | FMLY_CANCER_PATIEN_YN | Family history of other disease (including cancer) |
|  | SMK_STAT_TYPE_RSPS_CD | Smoking status |
|  | PAST_SMK_TERM_RSPS_CD | Years of smoking for past smokers |
|  | PAST_DSQTY_RSPS_CD | Number of cigarettes per day for past smokers |
|  | CUR_SMK_TERM_RSPS_CD | Years of smoking for current smokers |
|  | CUR_DSQTY_RSPS_CD | Number of cigarettes per day for current smokers |
|  | DRNK_HABIT_RSPS_CD | Frequency of alcohol consumption per week (Note: same variable name with that of 2002-20008 but different questions) |
|  | TM1_DRKQTY_RSPS_CD | Amount of alcohol intake per once (number of cups) |
|  | MOV20_WEK_FREQ_ID | Frequency of intensive exercise more than 20 minutes per week |
|  | MOV30_WEK_FREQ_ID | Frequency of moderate exercise more than 30 minutes per week |
|  | WLK30_WEK_FREQ_ID | Frequency of walking more than 30 minutes per week |

**Supplementary Table 2. Baseline characteristics of the study population according to age subgroups**

|  | | | Men (n=3,261,795) | | | | |  | Women (n=2,988,057) | | | | | |  |
| --- | --- | --- | --- | --- | --- | --- | --- | --- | --- | --- | --- | --- | --- | --- | --- |
| **Age groups** | <40  (n=1,169,606) | | | 40-49  (n=854,320) | 50-64  (n=894,469) | ≥65  (n=343,400) | *p* | | | <40  (n=732,797) | 40-49  (n=863,527) | 50-64  (n=999,831) | ≥65  (n=391,902) | *p* | |
| ***Clinical parameters*** | | | | | | | | | | | | | |  | |
| Age, years | | 31.9 ± 4.6 | | 44.2 ± 3.0 | 55.9 ± 4.3 | 70.9 ± 4.7 | <0.001 | | | 30.2 ± 5.1 | 44.1 ± 3.0 | 55.9 ± 4.4 | 71.2 ± 4.9 | <0.001 | |
| BMI, kg/m^2^ | | 24.1 ± 3.3 | | 24.4 ± 2.9 | 24.2 ± 2.8 | 23.4 ± 4.7 | <0.001 | | | 21.3 ± 3.2 | 23.1 ± 3.1 | 24.0 ± 3.9 | 24.1 ± 6.0 | <0.001 | |
| BMI groups, % | |  | |  |  |  |  | | |  |  |  |  |  | |
| <18.5 | | 28,778 (2.5) | | 12,816 (1.5) | 15,173 (1.7) | 15,502 (4.5) | <0.001 | | | 107,261 (14.6) | 29,043  (3.4) | 16,895  (1.7) | 13,346  (3.4) | <0.001 | |
| 18.5-22.9 | | 421,828 (36.1) | | 258,213 (30.2) | 275,189 (30.8) | 137,477 (40.0) |  |  |  | 456,301  (62.3) | 442,392 (51.2) | 371,542 (37.2) | 129,141 (33.0) |  |  |
| 23.0-24.9 | | 290,363 (24.8) | | 238,396 (27.9) | 261,460 (29.2) | 92,484  (26.9) |  |  |  | 85,658  (11.7) | 193,275 (22.4) | 269,396 (26.9) | 99,937  (25.5) |  |  |
| 25.0-29.9 | | 367,511 (31.4) | | 314,407 (36.8) | 321,096 (35.9) | 92,896  (27.1) |  |  |  | 67,924  (9.3) | 172,950 (20.0) | 303,745 (30.4) | 133,283 (34.0) |  |  |
| ≥30 | | 61,126 (5.2) | | 30,488 (3.6) | 21,551 (2.4) | 5,041 (1.5) |  |  |  | 15,653 (2.1) | 25,867 (3.0) | 38,253 (3.8) | 16,195 (4.1) |  |  |
| WC, cm | | 82.2 ± 8.2 | | 83.8 ± 7.4 | 84.7 ± 7.3 | 84.3 ± 8.1 | <0.001 | | | 70.6 ± 7.9 | 74.8 ± 7.8 | 78.5 ± 7.8 | 81.5 ± 8.3 | <0.001 | |
| Abdominal obesity, % | | 202,871 (17.3) | | 179,117 (21.0) | 220,610 (24.7) | 88,557  (25.8) | <0.001 | | | 43,127  (5.9) | 93,244  (10.8) | 213,577 (21.4) | 135,182 (34.5) | <0.001 | |
| Income, Q1, % | | 156,333 (13.4) | | 94,153  (11.0) | 155,252 (17.4) | 65,631  (19.1) | <0.001 | | | 180,255  (24.6) | 199,416 (23.1) | 218,190 (21.8) | 68,588  (17.5) | <0.001 | |
| Smoking, % | |  | |  |  |  |  | | |  |  |  |  |  | |
| Non | | 348,753 (29.8) | | 224,545 (26.3) | 280,077 (31.3) | 147,649 (43.0) | <0.001 | | | 671,770 (91.7) | 816,789 (94.6) | 960,054 (96.0) | 378,159 (96.5) | <0.001 | |
| Ex | | 184,671 (15.8) | | 228,519 (26.8) | 288,378 (32.2) | 107,091 (31.2) |  |  |  | 23,794 (3.3) | 15,479 (1.8) | 11,883 (1.2) | 4,108 (1.1) |  |  |
| Current | | 636,182 (54.4) | | 401,256 (47.0) | 326,014 (36.5) | 88,660  (25.8) |  |  |  | 37,233 (5.1) | 31,259 (3.6) | 27,894 (2.8) | 9,635 (2.5) |  |  |
| Drinking, % | |  | |  |  |  |  | | |  |  |  |  |  | |
| None | | 300,682 (25.7) | | 237,068 (27.8) | 319,732 (35.8) | 175,861 (51.2) | <0.001 | | | 408,760  (55.8) | 607,400 (70.3) | 830,949 (83.1) | 361,838 (92.3) | <0.001 | |
| Mild | | 705,788 (60.3) | | 474,251 (55.5) | 439,096 (49.1) | 127,793 (37.2) |  |  |  | 301,350  (41.1) | 238,527 (27.6) | 152,729 (15.3) | 25,977  (6.6) |  |  |
| Heavy | | 151,759 (13.0) | | 134,273 (15.7) | 125,813 (14.1) | 36,112  (10.5) |  |  |  | 15,307 (2.1) | 9,778 (1.1) | 7,040 (0.7) | 848 (0.2) |  |  |
| Exercise, % | | 185,725 (15.9) | | 163,302 (19.1) | 210,912 (23.6) | 89,704  (26.1) | <0.001 | | | 68,411  (9.3) | 141,448 (16.4) | 201,979 (20.2) | 59,799  (15.3) | <0.001 | |
| ***Comorbidities, %*** | | | | | | | | | | | | | | | |
| Diabetes | | 32,655  (2.8) | | 73,805  (8.6) | 136,383 (15.3) | 63,752  (18.6) | <0.001 | | | 6,981 (1.0) | 27,809 (3.2) | 87,397 (8.7) | 65,607 (16.7) | <0.001 | |
| Hypertension | | 127,945 (10.9) | | 178,473 (20.9) | 306,635 (34.28) | 167,937 (48.9) | <0.001 | | | 16,487  (2.3) | 92,169  (10.7) | 293,681 (29.4) | 211,853 (54.6) | <0.001 | |
| Dyslipidemia | | 355,523 (30.4) | | 344,189 (40.3) | 348,397 (39.0) | 114,386 (33.3) | <0.001 | | | 152,269 (20.8) | 280,444 (32.5) | 477,316 (47.7) | 221,220 (56.4) | <0.001 | |
| CKD | | 34,252 (2.9) | | 24,558 (2.9) | 35,865 (4.0) | 36,778 (10.7) | <0.001 | | | 17,894 (2.4) | 16,991 (2.0) | 41,284 (4.1) | 51,945 (13.3) | <0.001 | |
| ***Vital signs*** | | | | | | | | | | | | | | | |
| Systolic BP | | 122.2 ± 12.3 | | 123.5 ± 13.7 | 126.3 ± 14.9 | 129.9 ±16.1 | <0.001 | | | 111.3 ± 11.5 | 116.6 ± 14.0 | 122.9 ± 15.3 | 130.0 ± 16.3 | <0.001 | |
| Diastolic BP | | 76.6 ± 9.0 | | 78.3 ± 10.0 | 79.2 ± 10.0 | 78.3 ± 10.0 | <0.001 | | | 69.9 ± 8.5 | 73.0 ± 9.9 | 76.1 ± 10.0 | 78.0 ± 10.0 | <0.001 | |
| ***Laboratory parameters*** | | | | | | | | | | | | | | | |
| Fasting glucose, | | 92.6 ±18.1 | | 99.7 ± 25.9 | 104.4 ± 29.2 | 104.2 ± 27.8 | <0.001 | | | 88.2 ± 12.9 | 93.3 ± 17.7 | 97.8 ± 22.2 | 101.7 ± 25.3 | <0.001 | |
| Total cholesterol, | | 190.0 ± 35.0 | | 200.0 ± 36.4 | 197.8 ± 36.6 | 190.3 ± 36.3 | <0.001 | | | 178.5 ± 31.2 | 191.6 ± 33.9 | 208.7 ± 38.1 | 206.7 ± 38.9 | <0.001 | |
| TG | | 116 (79-176) | | 135 (92-119) | 128 (89-187) | 114 (82-163) | <0.001 | | | 70 (53-96) | 85 (62-119) | 107 (76-151) | 123 (90-170) | <0.001 | |
| HDL-c | | 52.7 ± 12.4 | | 51.9 ± 12.8 | 52.1 ± 13.2 | 52.2 ± 13.8 | <0.001 | | | 62.2 ± 13.9 | 58.9 ± 13.7 | 57.1 ± 13.7 | 54.2 ± 13.5 | <0.001 | |
| LDL-c | | 109.0 ± 34.8 | | 116.1 ± 36.7 | 115.4 ± 37.2 | 111.3 ± 35.9 | <0.001 | | | 99.9 ± 30.9 | 112.6 ± 33.5 | 126.6 ± 36.8 | 124.3 ± 37.2 | <0.001 | |
| Non-HDL-c | | 137.3 ± 36.0 | | 148.1 ± 36.7 | 145.8 ± 36.5 | 138.1 ± 35.8 | <0.001 | | | 116.3 ± 30.1 | 132.7 ± 33.4 | 151.6 ± 37.6 | 152.5 ± 38.1 | <0.001 | |
| eGFR | | 100.4 ± 25.3 | | 95.1 ± 23.4 | 90.2 ± 22.1 | 82.2 ± 20.3 | <.0001 | | | 102.8 ± 22.3 | 95.6 ± 18.1 | 88.2 ± 17.4 | 78.4 ± 16.9 | <0.001 | |

Values are presented as mean ± standard deviation, median (interquartile ranges) for triglycerides, or numbers (%). Measurement unit of laboratory findings are mg/dL BMI, body mass index; BP, blood pressure; CKD, chronic kidney disease; eGFR, estimated glomerular filtration rate; HDL-c, high-density lipoprotein cholesterol; LDL-c, low-density lipoprotein cholesterol; Q, quintile; WC, waist circumference.

**Supplementary Table 3. Associations of individual risk factors with ASCVD risk by age and sex**

1. **Total population**

|  | | **Total** | | | | | | | | | | | | | | | | | **<40 years old** | | | | | | | | | | | | | | | | | | | | | | | | | | | | | | | | | | | | **40-49 years old** | | | | | | | | | | | | | | | | | | | | | | | | | | | | | | | | |
| --- | --- | --- | --- | --- | --- | --- | --- | --- | --- | --- | --- | --- | --- | --- | --- | --- | --- | --- | --- | --- | --- | --- | --- | --- | --- | --- | --- | --- | --- | --- | --- | --- | --- | --- | --- | --- | --- | --- | --- | --- | --- | --- | --- | --- | --- | --- | --- | --- | --- | --- | --- | --- | --- | --- | --- | --- | --- | --- | --- | --- | --- | --- | --- | --- | --- | --- | --- | --- | --- | --- | --- | --- | --- | --- | --- | --- | --- | --- | --- | --- | --- | --- | --- | --- | --- | --- | --- |
|  | | **N** | | **Events** | | | | **IR** | | | **HR**  **(95% CI)** | | | ***p*** | | | | ***p*_trend_** | **N** | | | **Events** | | | | | | | | | | **IR** | | | | | **HR**  **(95% CI)** | | | | | ***p*** | | | | | | | | | | | ***p*_trend_** | | **N** | | | | | | | | **Events** | | | | | | **IR** | | | | | **HR**  **(95% CI)** | | | | | | ***p*** | | | | | | ***p*_trend_** | |
| **BMI, kg/m2** | | | | | | | | | | | | | | | | | | | | | | | | | | | | | | | | | | | | | | | | | | | | | | | | | | | | | | | | | | | | | | | | | | | | | | | | | | | | | | | | | | | | | | | |
| <18.5 | | 238,814 | | 8,368 | | | 2.85 | | | 1.15  (1.12–1.17) | | | | <0.001 | |  | | | 136,039 | | | 502 | | | | | | | | | | 0.29 | | | | | 1.04  (0.95–1.14) | | | | | 0.385 | | | | | <0.001 | | | | | | | | 41,859 | | | | | | | | 630 | | | | | | 1.19 | | | | | 1.00  (0.93–1.09) | | | | | | 0.956 | | | | | | <0.001 | |
| 18.5-22.9 | | 2,492,083 | | 94,017 | | | 3.02 | | | (ref) | | | | | | | |  | 878,129 | | | 4,296 | | | | | | | | | | 0.38 | | | | | (ref) | | | | | | | | | |  |  |  |  |  |  |  |  | 700,605 | | | | | | | | 11,705 | | | | | | 1.31 | | | | | (ref) | | | | | | | | | | | |  |  |
| 23.0-24.9 | | 1,530,969 | | 73,035 | | | 3.82 | | | 0.99  (0.98–1.00) | | | | 0.200 | | <0.001 | | | 376,021 | | | 3,021 | | | | | | | | | | 0.62 | | | | | 1.19  (1.14–1.25) | | | | | <0.001 | | | | |  |  |  |  |  |  |  |  | 431,671 | | | | | | | | 9,705 | | | | | | 1.76 | | | | | 1.09  (1.06–1.12) | | | | | | <0.001 | | | | | |  |  |
| 25.0-29.9 | | 1,773,812 | | 92,806 | | | 4.19 | | | 1.03  (1.02–1.04) | | | | <0.001 | |  | | | 435,435 | | | 5,191 | | | | | | | | | | 0.92 | | | | | 1.45  (1.39–1.52) | | | | | <0.001 | | | | |  |  |  |  |  |  |  |  | 487,357 | | | | | | | | 13,890 | | | | | | 2.24 | | | | | 1.20  (1.17–1.23) | | | | | | <0.001 | | | | | |  |  |
| ≥30.0 | | 214,174 | | 10,867 | | | 4.07 | | | 1.20  (1.18–1.23) | | | | <0.001 | |  | | | 76,779 | | | 1,535 | | | | | | | | | | 1.56 | | | | | 2.14  (2.01–2.28) | | | | | <0.001 | | | | |  |  |  |  |  |  |  |  | 56,355 | | | | | | | | 1,979 | | | | | | 2.79 | | | | | 1.41  (1.34–1.48) | | | | | | <0.001 | | | | | |  |  |
| **Smoking** | | | | | | | | | | | | | | | | | | | | | | | | | | | | | | | | | | | | | | | | | | | | | | | | | | | | | | | | | | | | | | | | | | | | | | | | | | | | | | | | | | | | | | | |
| Non | | 3,827,796 | | 156,650 | | | 3.27 | | | (ref) | | | | | | | |  | 1,020,523 | | | | | | | | 4,936 | | | | | 0.38 | | | | | (ref) | | | | | | | | | | <0.001 | | | | | | | | 1,041,334 | | | | | | 15,570 | | | | | | | 1.17 | | | | | (ref) | | | | | | | | | | |  | | | |
| Ex | | 863,923 | | 42,912 | | | 4.01 | | | 0.99  (0.98–1.00) | | | | 0.040 | | <0.001 | | | 208,465 | | | 1,704 | | | | | | | | | | 0.63 | | | | | 1.07  (1.01–1.14) | | | | | 0.032 | | | | |  |  |  |  |  |  |  |  | 243,998 | | | | | | | 5,361 | | | | | | 1.72 | | | | | 0.97  (0.94–1.01) | | | | | 0.157 | | | | | | <0.001 | | | |
| Current | | 1,558,133 | | 79,531 | | | 4.11 | | | 1.72  (1.70–1.74) | | | | <0.001 | |  | | | 673,415 | | | 7,905 | | | | | | | | | | 0.91 | | | | | 1.65  (1.58–1.73) | | | | | <0.001 | | | | |  |  |  |  |  |  |  |  | 432,515 | | | | | | | 16,978 | | | | | | 3.12 | | | | | 1.86  (1.81–1.92) | | | | | <0.001 | | | | | |  | | | |
| **Hypertension** | | | | | | | | | | | | | | | | | | | | | | | | | | | | | | | | | | | | | | | | | | | | | | | | | | | | | | | | | | | | | | | | | | | | | | | | | | | | | | | | | | | | | | | |
| Yes | | 1,395,180 | | 128,677 | | | 7.69 | | | 1.34  (1.33–1.35) | | | <0.001 | | | | | - | 144,432 | | 3,016 | | | | | | | | | 1.63 | | | | | | 1.87  (1.79–1.95) | | | | | | <0.001 | | | | | - | | | | | | | | 270,642 | | | | | 10,810 | | | | | | 3.17 | | | | | | | 1.61  (1.57–1.65) | | | | | <0.001 | | | | | | | - | | |
| **Diabetes** | | | | | | | | | | | | | | | | | | | | | | | | | | | | | | | | | | | | | | | | | | | | | | | | | | | | | | | | | | | | | | | | | | | | | | | | | | | | | | | | | | | | | | | |
| Normoglycemia | | 4,347,864 | | 152,067 | | | 2.78 | | | (ref) | | | | | | | |  | 1,561,963 | | | | | | 10,530 | | | | | | | | 0.52 | | | | | (ref) | | | | | | | | | <0.001 | | | | | | | | 1,216,685 | | | | 23,157 | | | | | | 1.49 | | | | | | | | (ref) | | | | | | | | | | | <0.001 | | | |
| Prediabetes | | 1,407,599 | | 72,948 | | | 4.18 | | | 1.03  (1.02–1.04) | | | <0.001 | | <0.001 | | | | 300,804 | | 3,079 | | | | | | | | | 0.79 | | | | | 1.06  (1.02–1.10) | | | | | | 0.006 | | | | | |  |  |  |  |  |  |  |  | 399,548 | | | | 9,727 | | | | | | 1.92 | | | | | | | | 1.03  (1.01–1.06) | | | 0.017 | | | | | | | |  |  |  |  |
| Diabetes | | 494,389 | | 54,078 | | | 9.33 | | | 1.48  (1.47–1.50) | | | <0.001 | |  | | | | 39,636 | | 936 | | | | | | | | | 1.85 | | | | | 1.66  (1.55–1.78) | | | | | | <0.001 | | | | | |  |  |  |  |  |  |  |  | 101,614 | | | | 5,025 | | | | | | 3.96 | | | | | | | | 1.62  (1.57–1.68) | | | <0.001 | | | | | | | |  |  |  |  |
| **Systolic BP, mmHg** | | | | | | | | | | | | | | | | | | | | | | | | | | | | | | | | | | | | | | | | | | | | | | | | | | | | | | | | | | | | | | | | | | | | | | | | | | | | | | | | | | | | | | | |
| <100 | | 221,402 | | 3,867 | | | 1.38 | | | (ref) | | | | | | | |  | 88,092 | | | | 268 | | | | | 0.24 | | | | | | | (ref) | | | | | | | | | | |  | | | | | | | | 74,010 | | | | 785 | | | | | | 0.83 | | | | | | | | (ref) | | | | | | | | | |  | | | | |  |
| 100-119 | | 2,371,006 | | 64,338 | | | 2.15 | | | 1.15  (1.11–1.19) | | | <0.001 | | | | |  | 879,732 | | | | 4,352 | | | | | 0.38 | | | | | | | 1.20  (1.06–1.36) | | | | 0.005 | | | | | | |  | | | | | | | | 721,388 | | | | 11,335 | | | | | | 1.23 | | | | | | | | 1.20  (1.11–1.29) | | | <0.001 | | | | | | |  | | | | |  |
| 120-139 | | 2,959,527 | | 144,089 | | | 3.91 | | | 1.40  (1.35–1.44) | | | <0.001 | | <0.001 | | | | 844,366 | | | | 7,831 | | | | | 0.72 | | | | | | | 1.63  (1.44–1.85) | | | | <0.001 | | | | | | | <0.001 | | | | | | | | 780,712 | | | | 19,572 | | | | | | 1.97 | | | | | | | | 1.57  (1.46–1.68) | | | <0.001 | | | | | | | <0.001 | | | | |  |
| 140-159 | | 568,074 | | 50,109 | | | 7.33 | | | 1.67  (1.61–1.72) | | | <0.001 | | | | |  | 77,539 | | | | 1,564 | | | | | 1.57 | | | | | | | 2.62  (2.29–3.00) | | | | <0.001 | | | | | | |  | | | | | | | | 118,115 | | | | 4,746 | | | | | | 3.19 | | | | | | | | 2.24  (2.08–2.42) | | | <0.001 | | | | | | |  | | | | |  |
| ≥160 | | 129,843 | | 16,690 | | | 11.10 | | | 2.10  (2.03–2.18) | | <0.001 | | | | |  | | 12,674 | | | | 530 | | | | | 3.31 | | | | | | 4.81  (4.13–5.60) | | | | | <0.001 | | | | | | |  | | | | | | | | | 23,622 | | | | 1,471 | | | | | | 5.04 | | | | | | | | 3.39  (3.11–3.71) | | | <0.001 | | | | | | | |  | | | |
| **Diastolic BP, mmHg** | | | | | | | | | | | | | | | | | | | | | | | | | | | | | | | | | | | | | | | | | | | | | | | | | | | | | | | | | | | | | | | | | | | | | | | | | | | | | | | | | | | | | | | |
| <60 | 150,910 | | 3,509 | | 1.85 | | | | (ref) | | | | | | | | | <0.001 | | 56,909 | | | | | | | 175 | | | | | 0.24 | | | | | (ref) | | | | | | | | | | | <0.001 | | | | | | | | 48,944 | | 507 | | | | | | | | | | 0.81 | | | | | | (ref) | | | | | | | | | | | | <0.001 | |
| 60-69 | 1,107,039 | | 30,479 | | 2.19 | | | | 1.04  (1.00–1.07) | | | | 0.043 | | | | |  |  | 400,546 | | | | | | | 1,623 | | | | | 0.31 | | | | | 1.15  (0.98–1.34) | | | | | | | | 0.087 | | |  |  |  |  |  |  |  |  | 335,298 | | 4,390 | | | | | | | | | | 1.03 | | | | | | 1.12  (1.03–1.23) | | | | | 0.013 | | | | | | |  |  |
| 70-79 | 2,268,533 | | 85,672 | | 3.01 | | | | 1.17  (1.14–1.21) | | | | <0.001 | | | | |  |  | 755,821 | | | | | | | 4,696 | | | | | 0.48 | | | | | 1.41  (1.21–1.64) | | | | | | | | <0.001 | | |  |  |  |  |  |  |  |  | 620,480 | | 11,522 | | | | | | | | | | 1.46 | | | | | | 1.34  (1.23–1.46) | | | | | <0.001 | | | | | | |  |  |
| 80-89 | 2,103,395 | | 109,430 | | 4.18 | | | | 1.36  (1.32–1.41) | | | | <0.001 | | | | |  |  | 587,863 | | | | | | | 5,796 | | | | | 0.76 | | | | | 1.78  (1.53–2.07) | | | | | | | | <0.001 | | |  |  |  |  |  |  |  |  | 553,425 | | 14,700 | | | | | | | | | | 2.09 | | | | | | 1.65  (1.51–1.81) | | | | | <0.001 | | | | | | |  |  |
| ≥90 | 619,975 | | 50,003 | | 6.65 | | | | 1.68  (1.63–1.74) | | | | <0.001 | | | | |  |  | 101,264 | | | | | | | 2,255 | | | | | 1.74 | | | | | 3.01  (2.57–3.52) | | | | | | | | <0.001 | | |  |  |  |  |  |  |  |  | 159,700 | | 6,790 | | | | | | | | | | 3.38 | | | | | | 2.42  (2.21–2.65) | | | | | <0.001 | | | | | | |  |  |
| **Non-HDL-c, mg/dL** | | | | | | | | | | | | | | | | | | | | | | | | | | | | | | | | | | | | | | | | | | | | | | | | | | | | | | | | | | | | | | | | | | | | | | | | | | | | | | | | | | | | | | | |
| <130 | 2,589,305 | | 87,470 | | 2.70 | | | | (ref) | | | | | | | | <0.001 | | | 1,041,985 | | | | | | 5,082 | | | | | | 0.38 | | | | | (ref) | | | | | | | | | | | | | | | <0.001 | | | | 700,724 | 11,318 | | | | | | | | | | | | | | 1.27 | | | (ref) | | | | | | | | | | | | <0.001 | |
| 130-159 | 1,913,101 | | 86,490 | | 3.62 | | | | 1.09  (1.08–1.10) | | | | <0.001 | | | |  |  |  | 510,962 | | | | | | 4,075 | | | | | | 0.62 | | | | | 1.17  (1.12–1.22) | | | | | | | <0.001 | | | | | | | |  |  |  |  | 549,841 | 11,345 | | | | | | | | | | | | | | 1.62 | | | 1.10  (1.07–1.12) | | | | | | | <0.001 | | | | |  |  |
| 160-189 | 1,150,763 | | 63,857 | | | 4.46 | | | 1.21  (1.20–1.22) | | | | <0.001 | | | |  |  |  | 242,787 | | | | | | 3,148 | | | | | | 1.00 | | | | | 1.53  (1.46–1.60) | | | | | | | <0.001 | | | | | | | |  |  |  |  | 314,844 | 8,803 | | | | | | | | | | | | | | 2.20 | | | 1.31  (1.28–1.35) | | | | | | | <0.001 | | | | |  |  |
| 190-219 | 436,771 | | 28,634 | | | 5.30 | | | 1.36  (1.35–1.38) | | | | <0.001 | | | |  |  |  | 79,766 | | | | | | 1,463 | | | | | | 1.42 | | | | | 1.88  (1.77–2.00) | | | | | | | <0.001 | | | | | | | |  |  |  |  | 112,950 | 4,307 | | | | | | | | | | | | | | 3.01 | | | 1.63  (1.58–1.69) | | | | | | | <0.001 | | | | |  |  |
| ≥220 | 159,912 | | 12,642 | | | 6.46 | | | 1.60  (1.57–1.63) | | | | <0.001 | | | |  |  |  | 26,903 | | | | | | 777 | | | | | | 2.26 | | | | | 2.71  (2.50–2.93) | | | | | | | <0.001 | | | | | | | |  |  |  |  | 39,488 | 2,136 | | | | | | | | | | | | | | 4.32 | | | 2.17  (2.07–2.28) | | | | | | | <0.001 | | | | |  |  |
| **LDL-c, mg/dL** | | | | | | | | | | | | | | | | | | | | | | | | | | | | | | | | | | | | | | | | | | | | | | | | | | | | | | | | | | | | | | | | | | | | | | | | | | | | | | | | | | | | | | | |
| <70 | 475,298 | | 20,831 | | | 3.54 | | | (ref) | | | | | | | | <0.001 | | | 190,321 | | | | | | 1,238 | | | | | 0.50 | | | | | | (ref) | | | | | | | | | | | | | | | <0.001 | | | | 116,751 | | 2,811 | | | | | | | | | | | | 1.90 | | | | (ref) | | | | | | | | | | | | <0.001 | |
| 70-99 | 1,703,244 | | 6,1922 | | | 2.90 | | | 0.95  (0.94–0.97) | | | | <0.001 | | | |  |  |  | 674,961 | | | | | | 3,794 | | | | | 0.44 | | | | | | 0.91  (0.85–0.97) | | | | | | 0.003 | | | | | | | | |  |  |  |  | 457,891 | | 8,216 | | | | | | | | | | | | 1.41 | | | | 0.89  (0.85–0.93) | | | | | | <0.001 | | | | | |  |  |
| 100-129 | 2,212,393 | | 93,222 | | | 3.37 | | | 1.01  (0.99–1.02) | | | | 0.504 | | | |  |  |  | 666,476 | | | | | | 4,873 | | | | | 0.57 | | | | | | 0.99  (0.93–1.05) | | | | | | 0.732 | | | | | | | | |  |  |  |  | 642,418 | | 12,757 | | | | | | | | | | | | 1.56 | | | | 0.96  (0.92–1.00) | | | | | | 0.027 | | | | | |  |  |
| 130-159 | 1,294,595 | | 66,785 | | | 4.14 | | | 1.09  (1.07–1.11) | | | | <0.001 | | | |  |  |  | 281,961 | | | | | | 3,085 | | | | | 0.85 | | | | | | 1.21  (1.13–1.29) | | | | | | <0.001 | | | | | | | | |  |  |  |  | 361,073 | | 9,114 | | | | | | | | | | | | 1.98 | | | | 1.12  (1.07–1.17) | | | | | | <0.001 | | | | | |  |  |
| ≥160 | 564,322 | | 36,333 | | | 5.21 | | | 1.25  (1.23–1.27) | | | | <0.001 | | | |  |  |  | 88,684 | | | | | | 1,555 | | | | | 1.36 | | | | | | 1.67  (1.55–1.81) | | | | | | <0.001 | | | | | | | | |  |  |  |  | 139,714 | | 5,011 | | | | | | | | | | | | 2.84 | | | | 1.48  (1.41–1.55) | | | | | | <0.001 | | | | | |  |  |
| **Triglycerides, mg/dL** | | | | | | | | | | | | | | | | | | | | | | | | | | | | | | | | | | | | | | | | | | | | | | | | | | | | | | | | | | | | | | | | | | | | | | | | | | | | | | | | | | | | | | | |
| <100 | 2,805,260 | | 87,490 | | | 2.48 | | | (ref) | | | | | | | | <0.001 | | | 1,025,571 | | | | | | 4,851 | | | | | 0.37 | | | | | | (ref) | | | | | | | | | | | | | | <0.001 | | | | | 793,014 | | 11,778 | | | | | | | | | | | 1.16 | | | | | (ref) | | | | | | | | | | | <0.001 | | |
| 100-149 | 1,669,525 | | 84,414 | | | 4.07 | | | 1.13  (1.12–1.14) | | | | <0.001 | | | |  |  |  | 428,981 | | | | | | 3,542 | | | | | 0.64 | | | | | | 1.18  (1.13–1.24) | | | | | <0.001 | | | | | | | | |  |  |  |  |  | 439,169 | | 9,825 | | | | | | | | | | | 1.76 | | | | | 1.15  (1.12–1.18) | | | | | | <0.001 | | | | |  |  |  |
| 150-199 | 844,984 | | 49,278 | | | 4.71 | | | 1.21  (1.19–1.22) | | | | <0.001 | | | |  |  |  | 205,143 | | | | | | 2,205 | | | | | 0.83 | | | | | | 1.25  (1.18–1.32) | | | | | <0.001 | | | | | | | | |  |  |  |  |  | 221,637 | | 6,446 | | | | | | | | | | | 2.29 | | | | | 1.28  (1.24–1.32) | | | | | | <0.001 | | | | |  |  |  |
| ≥200 | 930,083 | | 57,911 | | | 5.03 | | | 1.32  (1.31–1.34) | | | | <0.001 | | | |  |  |  | 242,708 | | | | | | 3,947 | | | | | 1.26 | | | | | | 1.53  (1.46–1.61) | | | | | <0.001 | | | | | | | | |  |  |  |  |  | 264,027 | | 9,860 | | | | | | | | | | | 2.95 | | | | | 1.41  (1.36–1.45) | | | | | | <0.001 | | | | |  |  |  |
| **Abdominal obesity** | | | | | | | | | | | | | | | | | | | | | | | | | | | | | | | | | | | | | | | | | | | | | | | | | | | | | | | | | | | | | | | | | | | | | | | | | | | | | | | | | | | | | | | |
| Yes | 1,176,285 | | 80,628 | | | 5.58 | | | 1.08  (1.07–1.09) | | | | <0.001 | | | | - | | | 245,998 | | | | 3,746 | | | | | 1.18 | | | | | | | | 1.42  (1.37–1.48) | | | <0.001 | | | | | | | | | | - | | | | | | 272,361 | | 8,867 | | | | | | | | | | 2.57 | | | | | | 1.18  (1.15–1.21) | | | | <0.001 | | | | | | - | | | |
| **Regular physical activity** | | | | | | | | | | | | | | | | | | | | | | | | | | | | | | | | | | | | | | | | | | | | | | | | | | | | | | | | | | | | | | | | | | | | | | | | | | | | | | | | | | | | | | | |
| Yes | 1,121,280 | | 52,362 | | | 3.74 | | | 1.13  (1.12–1.14) | | | | <0.001 | | | | - | | | 254,136 | | | 2,143 | | | | | 0.65 | | | | | | | | | 0.98  (0.93–1.02) | | 0.312 | | | | | | | | | | - | | | | | | | 304,750 | | 6,602 | | | | | | | | | 1.70 | | | | | | | 1.05  (1.02–1.08) | | | <0.001 | | | | | | - | | | | |

|  | | | | **50-64 years old** | | | | | | | | | | | | | | | | | | | | | | | | | | | | | | **≥65 years old** | | | | | | | | | | | | | | | | | | | | | | | | | | | | | | | | | | | |  | | | |
| --- | --- | --- | --- | --- | --- | --- | --- | --- | --- | --- | --- | --- | --- | --- | --- | --- | --- | --- | --- | --- | --- | --- | --- | --- | --- | --- | --- | --- | --- | --- | --- | --- | --- | --- | --- | --- | --- | --- | --- | --- | --- | --- | --- | --- | --- | --- | --- | --- | --- | --- | --- | --- | --- | --- | --- | --- | --- | --- | --- | --- | --- | --- | --- | --- | --- | --- | --- | --- | --- | --- | --- | --- | --- |
|  | | | | **N** | | **Events** | | | | | | | **IR** | | | | | | **HR**  **(95% CI)** | | | | | | | ***p*** | ***p*_trend_** | | | | | | **N** | | | | | | **Events** | | | | | | | | **IR** | | | **HR**  **(95% CI)** | | | | | | | ***p*** | | | | | ***p*_trend_** | | | | | | | | ***p*_interaction_** | | | |
| **BMI, kg/m2** | | | | | | | | | | | | | | | | | | | | | | | | | | | | | | | | | | | | | | | | | | | | | | | | | | | | | | | | | | | | | | | | | | | | | | | | | |
| <18.5 | | | | 32,068 | | 1,792 | | | | | | | 4.65 | | | | | | 1.15  (1.09–1.20) | | | | | | | <0.001 | | <0.001 | | | | | 28,848 | | | | | | 5,444 | | | | | | | | | 1.19 | | | 1.14  (1.11–1.18) | | | | | | <0.001 | | | | | <0.001 | | | | | | | | <0.001 | | | |
| 18.5-22.9 | | | | 646,731 | | 31,178 | | | | | | | 3.88 | | | | | | (ref) | | | | | | | | |  |  |  |  |  | 266,618 | | | | | | 46,838 | | | | | | | | | 1.31 | | | (ref) | | | | | | | | | | |  |  |  |  |  |  |  |  |  | | | |
| 23.0-24.9 | | | | 530,856 | | 28,601 | | | | | | | 4.33 | | | | | | 1.02  (1.00–1.04) | | | | | | | 0.020 | |  |  |  |  |  | 192,421 | | | | | | 31,708 | | | | | | | | | 1.76 | | | 0.93  (0.92–0.94) | | | | | | <0.001 | | | | |  |  |  |  |  |  |  |  |  | | | |
| 25.0-29.9 | | | | 624,841 | | 36,980 | | | | | | | 4.76 | | | | | | 1.05  (1.03–1.06) | | | | | | | <0.001 | |  |  |  |  |  | 226,179 | | | | | | 36,745 | | | | | | | | | 2.24 | | | 0.92  (0.91–0.93) | | | | | | <0.001 | | | | |  |  |  |  |  |  |  |  |  | | | |
| ≥30.0 | | | | 59,804 | | 3,832 | | | | | | | 5.18 | | | | | | 1.14  (1.11–1.18) | | | | | | | <0.001 | |  |  |  |  |  | 21,236 | | | | | | 3,521 | | | | | | | | | 2.79 | | | 0.95  (0.92–0.99) | | | | | | <0.001 | | | | |  |  |  |  |  |  |  |  |  | | | |
| **Smoking** | | | | | | | | | | | | | | | | | | | | | | | | | | | | | | | | | | | | | | | | | | | | | | | | | | | | | | | | | | | | | | | | | | | | | | | | | |
| Non | | | | 1,240,131 | 52,430 | | | | | | | | | | | 3.36 | | | (ref) | | | | | | | | | <0.001 | | | | | | 525,808 | 83,714 | | | | | | | | 14.22 | | | | | | | | | | | (ref) | | | | | | | | <0.001 | | | | | | | | <0.001 | | | |
| Ex | | | | 300,261 | 17,033 | | | | | | | | | | | 4.59 | | | 0.96  (0.94–0.98) | | <0.001 | | | | | | |  |  |  |  |  |  | 111,199 | 18,814 | | | | | | | | 16.00 | | | | | | | | | | 1.01  (0.99–1.02) | | | | 0.538 | | | | |  |  |  |  |  |  |  |  |  | | | |
| Current | | | | 353,908 | 32,920 | | | | | | | | | | | 7.77 | | | 1.77  (1.74–1.80) | | <0.001 | | | | | | |  |  |  |  |  |  | 98,295 | 21,728 | | | | | | | | 22.56 | | | | | | | | | | 1.52  (1.49–1.54) | | | | <0.001 | | | | |  |  |  |  |  |  |  |  |  | | | |
| **Hypertension** | | | | | | | | | | | | | | | | | | | | | | | | | | | | | | | | | | | | | | | | | | | | | | | | | | | | | | | | | | | | | | | | | | | | | | | | | |
| Yes | | | | 600,316 | | | 43,376 | | | | | | | | | 5.89 | | | 1.36  (1.34–1.38) | | <0.001 | | | | | | | - | | | | | | 379,790 | | | | 71,475 | | | | 17.42 | | | | | | | | | | 1.23  (1.21–1.24) | | | | | <0.001 | | | | | - | | | | | | | | <0.001 | | | |
| **Diabetes** | | | | | | | | | | | | | | | | | | | | | | | | | | | | | | | | | | | | | | | | | | | | | | | | | | | | | | | | | | | | | | | | | | | | | | | | | |
| Normoglycemia | | | | 1,161,480 | | | | 53,772 | | | | | | | | 3.70 | | (ref) | | | | | | | | | | | | | <0.001 | | | 407,736 | | | | 64,608 | | | 14.29 | | | | | | | | | | (ref) | | | | | | | | | |  | | | | | | | | | <0.001 | | | |
| Prediabetes | | | | 509,040 | | | 27,658 | | | | | | | | | 4.38 | | 1.03  (1.02–1.05) | | | | | <0.001 | | | | | | | |  |  |  | 198,207 | | | | 32,484 | | | 14.97 | | | | | | | | | | 1.02  (1.00–1.03) | | | | | | 0.022 | | | | | | | | <0.001 | | | | |  | | | |
| Diabetes | | | | 223,780 | | | 20,953 | | | | | | | | | 7.81 | | 1.58  (1.56–1.61) | | | | | <0.001 | | | | | | | |  |  |  | 129,359 | | | 27,164 | | | | 20.33 | | | | | | | | | | 1.39  (1.37–1.41) | | | | | | <0.001 | | | | | | | |  | | | | |  | | | |
| **Systolic BP, mmHg** | | | | | | | | | | | | | | | | | | | | | | | | | | | | | | | | | | | | | | | | | | | | | | | | | | | | | | | | | | | | | | | | | | | | | | | | | |
| <100 | | | | 50,473 | | | | | 1,609 | | | | | | | | 2.54 | | (ref) | | | | | | | | | | | |  | | | 8,827 | | 1,205 | | | | 12.76 | | | | | | | | | | (ref) | | | | | | | | | |  | | | | | | | | | | | <0.001 | | |
| 100-119 | | | | 609,874 | | | | | 25,148 | | | | | | | | 3.29 | | 1.14  (1.08–1.19) | | | | | <0.001 | | | | | | |  | | | 160,012 | | 23,503 | | | | 13.32 | | | | | | | | | | 1.04  (0.98–1.10) | | | | | | | 0.236 | | | | | | | | |  | | | | |  | | |
| 120-139 | | | | 953,152 | | | | | 53,429 | | | | | | | | 4.51 | | 1.38  (1.32–1.46) | | | | | <0.001 | | | | | | | <0.001 | | | 381,297 | | 63,257 | | | | 15.12 | | | | | | | | | | 1.16  (1.09–1.22) | | | | | | | <0.001 | | | | | | | | | <0.001 | | | | |  | | |
| 140-159 | | | | 229,942 | | | | | 17,100 | | | | | | | | 6.07 | | 1.71  (1.63–1.81) | | | | | <0.001 | | | | | | |  | | | 142,478 | | 26,699 | | | | 17.34 | | | | | | | | | | 1.30  (1.22–1.37) | | | | | | | <0.001 | | | | | | | | |  | | | | |  | | |
| ≥160 | | | | 50,859 | | | | | 5,097 | | | | | | | | 8.36 | | 2.26  (2.13–2.39) | | | | | <0.001 | | | | | |  | | | | 42,688 | | 9,592 | | | | 21.68 | | | | | | | | | | 1.55  (1.46–1.65) | | | | | | | <0.001 | | | | | | | | |  | | | | |  | | |
| **Diastolic BP, mmHg** | | | | | | | | | | | | | | | | | | | | | | | | | | | | | | | | | | | | | | | | | | | | | | | | | | | | | | | | | | | | | | | | | | | | | | | | | |
| <60 | 34,405 | | | | | | | | | 1,205 | | | | | | 2.79 | | | (ref) | | | | | | | | | | | | |  | | 10,652 | | | | 1,622 | | | | | | 14.28 | | | | | (ref) | | | | | | | |  | | | | | | | | | |  | | | | <0.001 | | |
| 60-69 | 278,331 | | | | | | | | | 10,704 | | | | | | 3.07 | | | 1.03  (0.97–1.09) | | | | | | 0.388 | | | | | | |  | | 92,864 | | | | 13,762 | | | | | | 13.50 | | | | | 0.99  (0.94–1.04) | | | | | | | | 0.682 | | | | | | | | | |  | | | |  | | |
| 70-79 | | 649,250 | | | | | | | | | | 31,187 | | | 3.85 | | | | | 1.17  (1.10–1.24) | | | | | <0.001 | | | | | | | <0.001 | | 242,982 | | | | 38,267 | | | | | | | | 14.32 | | | | | | | | | | 1.07  (1.01–1.12) | | | 0.012 | | | <0.001 | | | | | | |  | | | | |
| 80-89 | | 686,011 | | | | | | | | | | 40,745 | | | 4.79 | | | | | 1.36  (1.28–1.44) | | | | | <0.001 | | | | | | |  | | 276,096 | | | | 48,189 | | | | | | | | 15.98 | | | | | | | | | | 1.19  (1.13–1.25) | | | <0.001 | | |  | | | | | | |  | | | | |
| ≥90 | | 246,303 | | | | | | | | | | 18,542 | | | 6.15 | | | | | 1.67  (1.57–1.77) | | | | | <0.001 | | | | | | |  | | 112,708 | | | | 22,416 | | | | | | | | 18.58 | | | | | | | | | | 1.37  (1.30–1.44) | | | <0.001 | | |  | | | | | | |  | | | | |
| **Non-HDL-c, mg/dL** | | | | | | | | | | | | | | | | | | | | | | | | | | | | | | | | | | | | | | | | | | | | | | | | | | | | | | | | | | | | | | | | | | | | | | | | | |
| <130 | | | 591,275 | | | | | | | | 29,479 | | | 4.03 | | | | (ref) | | | | | | | | | | |  | | | | | 255,321 | | | 41,591 | | | | | | | | 15.25 | | | | | | | | | | (ref) | | | | | | | |  | | | | | <0.001 | | | | |  |
| 130-159 | | | 618,456 | | | | | | | | 32,089 | | | 4.16 | | | | 1.08  (1.07–1.10) | | | | <0.001 | | | | | | |  | | | | | 233,842 | | | 38,981 | | | | | | | | 15.14 | | | | | | | | | | 1.06  (1.04–1.07) | | | <0.001 | | | | |  | | | | |  | | | | |  |
| 160-189 | | | 435,088 | | | | | | | | 24,625 | | | 4.54 | | | | 1.19  (1.17–1.22) | | | | <0.001 | | | | | | | <0.001 | | | | | 158,044 | | | 27,281 | | | | | | | | 15.57 | | | | | | | | | | 1.12  (1.11–1.14) | | | <0.001 | | | | | <0.001 | | | | |  | | | | |  |
| 190-219 | | | 180,448 | | | | | | | | 11,328 | | | 5.06 | | | | 1.34  (1.31–1.37) | | | | <0.001 | | | | | | |  | | | | | 63,607 | | | 11,536 | | | | | | | | 16.38 | | | | | | | | | | 1.21  (1.18–1.23) | | | <0.001 | | | | |  | | | | |  | | | | |  |
| ≥220 | | | 69,033 | | | | | | | | 4,862 | | | 5.71 | | | | 1.50  (1.46–1.66) | | | | <0.001 | | | | | | |  | | | | | 24,488 | | | 4,867 | | | | | | | | 18.23 | | | | | | | | | | 1.34  (1.30–1.38) | | | <0.001 | | | | |  | | | | |  | | | | |  |
| **LDL-c, mg/dL** | | | | | | | | | | | | | | | | | | | | | | | | | | | | | | | | | | | | | | | | | | | | | | | | | | | | | | | | | | | | | | | | | | | | | | | | | |
| <70 | | | 114,139 | | | | | | | | 7,415 | | | 5.35 | | | | (ref) | | | | | | | | | | |  | | | | | 54,087 | | | 9,367 | | | | | | | | 16.71 | | | | | | | | | | (ref) | | | | | | | |  | | | | | <0.001 | | | | |  |
| 70-99 | | | 398,665 | | | | | | | | 21,297 | | | 4.31 | | | | 0.96  (0.94–0.99) | | | | 0.003 | | | | | | |  | | | | | 171,727 | | | 28,615 | | | | | | | | 15.51 | | | | | | | | | | 0.89  (0.85–0.93) | | | <0.001 | | | | |  | | | | |  | | | | |  |
| 100-129 | | | 653,239 | | | | | | | | 33,860 | | | 4.16 | | | | 1.01  (0.99–1.04) | | | | 0.301 | | | | | | | <0.001 | | | | | 250,260 | | | 41,732 | | | | | | | | 15.21 | | | | | | | | | | 0.96  (0.92–1.00) | | | 0.027 | | | | | <0.001 | | | | |  | | | | |  |
| 130-159 | | | 478,788 | | | | | | | | 25,451 | | | 4.26 | | | | 1.09  (1.06–1.12) | | | | <0.001 | | | | | | |  | | | | | 172,773 | | | 29,135 | | | | | | | | 15.20 | | | | | | | | | | 1.12  (1.07–1.17) | | | <0.001 | | | | |  | | | | |  | | | | |  |
| ≥160 | | | 249,469 | | | | | | | | 14,360 | | | 4.63 | | | | 1.23  (1.20–1.27) | | | | <0.001 | | | | | | |  | | | | | 86,455 | | | 15,407 | | | | | | | | 16.06 | | | | | | | | | | 1.48  (1.41–1.55) | | | <0.001 | | | | |  | | | | |  | | | | |  |
| **Triglycerides, mg/dL** | | | | | | | | | | | | | | | | | | | | | | | | | | | | | | | | | | | | | | | | | | | | | | | | | | | | | | | | | | | | | | | | | | | | | | | | | |
| <100 | | | 727,549 | | | | | | | | 31,039 | | | 3.41 | | | | (ref) | | | | | | | | | | | <0.001 | | | | | 259,126 | | | 39,822 | | | | | | | | 14.08 | | | | | | | | | | (ref) | | | | | | | | <0.001 | | | | | <0.001 | | | | |  |
| 100-149 | | | 561,527 | | | | | | | | 30,310 | | | 4.34 | | | | 1.12  (1.10–1.13) | | | | <0.001 | | | | | | |  |  |  |  |  | 239,848 | | | 40,737 | | | | | | | | 15.56 | | | | | | | | | | 1.09  (1.08–1.11) | | | <0.001 | | | | |  |  |  |  |  |  | | | | |  |
| 150-199 | | | 294,398 | | | | | | | | 18,449 | | | 5.06 | | | | 1.20  (1.18–1.22) | | | | <0.001 | | | | | | |  |  |  |  |  | 123,806 | | | 22,178 | | | | | | | | 16.38 | | | | | | | | | | 1.15  (1.13–1.17) | | | <0.001 | | | | |  |  |  |  |  |  | | | | |  |
| ≥200 | | | 310,826 | | | | | | | | 22,585 | | | 5.90 | | | | 1.27  (1.25–1.30) | | | | <0.001 | | | | | | |  |  |  |  |  | 112,522 | | | 21,519 | | | | | | | | 17.57 | | | | | | | | | | 1.22  (1.20–1.24) | | | <0.001 | | | | |  |  |  |  |  |  | | | | |  |
| **Abdominal obesity** | | | | | | | | | | | | | | | | | | | | | | | | | | | | | | | | | | | | | | | | | | | | | | | | | | | | | | | | | | | | | | | | | | | | | | | | | |
| Yes | | | 434,187 | | | | | | | | | 28,694 | | | 5.35 | | | 1.09  (1.08–1.11) | | | | <0.001 | | | | | | | - | | | | | 223,739 | | | 39,321 | | | | | | | | 15.91 | | | | | | | | | | 1.01  (1.00–1.03) | | | 0.044 | | | | | | - | | | | | <0.001 | | |  |  |
| **Regular physical activity** | | | | | | | | | | | | | | | | | | | | | | | | | | | | | | | | | | | | | | | | | | | | | | | | | | | | | | | | | | | | | | | | | | | | | | | | | |
| Yes | | | 412,891 | | | | | | | | | 21,253 | | | 4.13 | | | 1.11  (1.09–1.13) | | | | <0.001 | | | | | | | - | | | | | 149,503 | | | 22,364 | | | | | | | | 13.34 | | | | | | | | | | 1.16  (1.15–1.18) | | | <0.001 | | | | | - | | | | | <0.001 | | | |  |  |

1. **Men**

|  | **Total** | | | | | | | | | | | | | | | | | | | **<40 years old** | | | | | | | | | | | | | | | | | | | | | | | | | | | | | | | | | | | | | | | | | **40-49 years old** | | | | | | | | | | | | | | | | | | | | | |
| --- | --- | --- | --- | --- | --- | --- | --- | --- | --- | --- | --- | --- | --- | --- | --- | --- | --- | --- | --- | --- | --- | --- | --- | --- | --- | --- | --- | --- | --- | --- | --- | --- | --- | --- | --- | --- | --- | --- | --- | --- | --- | --- | --- | --- | --- | --- | --- | --- | --- | --- | --- | --- | --- | --- | --- | --- | --- | --- | --- | --- | --- | --- | --- | --- | --- | --- | --- | --- | --- | --- | --- | --- | --- | --- | --- | --- | --- | --- | --- | --- | --- | --- |
|  | **N** | **Events** | | | | **IR** | | | | **HR**  **(95% CI)** | ***p*** | | | | | ***p*_trend_** | | | | **N** | | | | | **Events** | | | | | | | | | | | **IR** | | | | | | | **HR**  **(95% CI)** | | | | | | ***p*** | | | | | | | | | ***p*_trend_** | | | **N** | | | | **Events** | | | | | **IR** | | | | **HR**  **(95% CI)** | | | ***p*** | | | | | ***p*_trend_** |
| **BMI, kg/m2** | | | | | | | | | | | | | | | | | | | | | | | | | | | | | | | | | | | | | | | | | | | | | | | | | | | | | | | | | | | | | | | | | | | | | | | | | | | | | | | | | | |
| <18.5 | 72,269 | 4,649 | | | | 5.55 | | | | 1.13  (1.10–1.16) | <0.001 | | | | |  | | | | 28,778 | | | | | 170 | | | | | | | | | | | 0.46 | | | | | | | 0.95  (0.81–1.11) | | | | | | | 0.528 | | | | | | | |  | | | 12,816 | | | | | | 351 | | | | 2.19 | | | | 1.05  (0.94–1.17) | | 0.410 | | | | |  |
| 18.5-22.9 | 109,2707 | 55,430 | | | | 4.11 | | | | (ref) | | |  | | | | | | | 421,828 | | | | | 2,817 | | | | | | | | | | | 0.51 | | | | | | | (ref) | | | | | | | | | | | | | | |  | | | 258,213 | | | | | | 6,905 | | | | 2.10 | | | | (ref) | | | | | | |  |
| 23.0-24.9 | 882,703 | 45,226 | | | | 4.12 | | | | 0.98  (0.97–0.99) | 0.002 | | | <0.001 | | | | | | 290,363 | | | | | 2,609 | | | | | | | | | | | 0.69 | | | | | | | 1.17  (1.11–1.23) | | | | | | | <0.001 | | | | | | | | <0.001 | | | 238,396 | | | | | | 7,008 | | | | 2.31 | | | | 1.05  (1.01–1.08) | | 0.009 | | | | | <0.001 |
| 25.0-29.9 | 109,5910 | 56,667 | | | | 4.15 | | | | 1.02  (1.01–1.03) | 0.003 | | |  | | | | | | 367,511 | | | | | 4,779 | | | | | | | | | | | 1.01 | | | | | | | 1.44  (1.37–1.51) | | | | | | | <0.001 | | | | | | | |  | | | 314,407 | | | | | | 11,061 | | | | 2.78 | | | | 1.17  (1.13–1.20) | | <0.001 | | | | |  |
| ≥30.0 | 118,206 | 5,572 | | | | 3.76 | | | | 1.25  (1.21–1.28) | <0.001 | | |  | | | | | | 61,126 | | | | | 1,363 | | | | | | | | | | | 1.74 | | | | | | | 2.10  (1.96–2.25) | | | | | | | <0.001 | | | | | | | |  | | | 30,488 | | | | | | 1,447 | | | | 3.79 | | | | 1.40  (1.32–1.48) | | <0.001 | | | | |  |
| **Smoking** | | | | | | | | | | | | | | | | | | | | | | | | | | | | | | | | | | | | | | | | | | | | | | | | | | | | | | | | | | | | | | | | | | | | | | | | | | | | | | | | | | |
| Non | 1,001,024 | 51,742 | | | | 4.17 | | | | (ref) | | |  | | | | | | | 348,753 | | | | | 2,426 | | | | | | | | | | | 0.54 | | | | | | | (ref) | | | | | | | | | | | | |  | | | | | 224,545 | | | | | | 5,399 | | | | 1.88 | | | | (ref) | | | | | | |  |
| Ex | 808,659 | 41,292 | | | | 4.13 | | | | 0.97  (0.96–0.98) | <0.001 | | | <0.001 | | | | | | 184,671 | | | | | 1,612 | | | | | | | | | | | 0.67 | | | | | | | 1.07  (1.00–1.14) | | | | | | | 0.037 | | | | | | | | <0.001 | | | 228,519 | | | | | | 5,121 | | | | 1.75 | | | | 0.96  (0.92–1.00) | | 0.029 | | | | | <0.001 |
| Current | 1,452,112 | 74,510 | | | | 4.13 | | | | 1.67  (1.65–1.69) | <0.001 | | |  | | | | | | 636,182 | | | | | 7,700 | | | | | | | | | | | 0.94 | | | | | | | 1.66  (1.59–1.74) | | | | | | | <0.001 | | | | | | | |  | | | 401,256 | | | | | | 16,252 | | | | 3.22 | | | | 1.85  (1.79–1.91) | | <0.001 | | | | |  |
| **Hypertension** | | | | | | | | | | | | | | | | | | | | | | | | | | | | | | | | | | | | | | | | | | | | | | | | | | | | | | | | | | | | | | | | | | | | | | | | | | | | | | | | | | |
| Yes | 780,990 | 73,721 | | | | 7.93 | | | | 1.33  (1.32–1.35) | <0.001 | | | | - | | | | | 127,945 | | | | | 2,800 | | | | | | | | | | 1.71 | | | | | | | 1.84  (1.76–1.93) | | | | | | | | <0.001 | | | | | | | | - | | | 178,473 | | | | | | 8,533 | | | | 3.81 | | | | 1.57  (1.53–1.62) | | <0.001 | | | | | - |
| **Diabetes** | | | | | | | | | | | | | | | | | | | | | | | | | | | | | | | | | | | | | | | | | | | | | | | | | | | | | | | | | | | | | | | | | | | | | | | | | | | | | | | | | | |
| Normoglycemia | 2,110,224 | 86,522 | | | | 3.27 | | | | (ref) | | | | |  | | | | | 910,342 | | | | | | 8,202 | | | | | | | | 0.70 | | | | | | | (ref) | | | | | | | | | | | | | | | | |  | | | 537,980 | | | | | | 14,984 | | | | | 2.19 | | | (ref) | | | | | | |  |
| Prediabetes | 844,976 | 47,005 | | | | 4.51 | | | | 1.04  (1.03–1.05) | <0.001 | | | | <0.001 | | | | | 226,609 | | | | | 2,693 | | | | | | | | | | 0.92 | | | | | 1.04  (1.00–1.09) | | | | | | | | | | 0.074 | | | | | | | | <0.001 | | | 242,535 | | | | | | 7,610 | | | | | 2.48 | | | 1.04  (1.01–1.07) | | 0.005 | | | | | <0.001 |
| Diabetes | 306,595 | 34,017 | | | | 9.54 | | | | 1.48  (1.46–1.50) | <0.001 | | | |  | | | | | 32,655 | | | | | 843 | | | | | | | | | | 2.02 | | | | | 1.61  (1.49–1.73) | | | | | | | | | | <0.001 | | | | | | | |  | | | 73,805 | | | | | | 4,178 | | | | | 4.55 | | | 1.58  (1.52–1.64) | | <0.001 | | | | |  |
| **Systolic BP, mmHg** | | | | | | | | | | | | | | | | | | | | | | | | | | | | | | | | | | | | | | | | | | | | | | | | | | | | | | | | | | | | | | | | | | | | | | | | | | | | | | | | | | |
| <100 | 45,444 | 1,687 | | | | 3.00 | | | | (ref) | | | | |  | | | | | 13,637 | | | | | | | 70 | | | | | | 0.40 | | | | | | | (ref) | | | | | | | | | | | | | | | | | |  | | | 13,963 | | | | | | 254 | | | | | 1.43 | | | (ref) | | | | | | |  |
| 100-119 | 1,030,001 | 36,414 | | | | 2.81 | | | | 1.11  (1.05–1.16) | <0.001 | | | |  | | | | | 419,475 | | | | | | | 2,840 | | | | | | 0.52 | | | | | | | 1.23  (0.97–1.55) | | | | | | | | | | 0.094 | | | | | | | |  | | | 286,055 | | | | | | 6,748 | | | | | 1.85 | | | 1.31  (1.16–1.49) | | <0.001 | | | | |  |
| 120-139 | 1,774,926 | 89,256 | | | | 4.05 | | | | 1.35  (1.29–1.42) | <0.001 | | | | <0.001 | | | | | 654,740 | | | | | | | 6,879 | | | | | | 0.81 | | | | | | | 1.68  (1.32–2.12) | | | | | | | | | | <0.001 | | | | | | | | <0.001 | | | 460,502 | | | | | | 14,758 | | | | | 2.53 | | | 1.73  (1.53–1.96) | | <0.001 | | | | | <0.001 |
| 140-159 | 337,114 | 30,266 | | | | 7.51 | | | | 1.66  (1.58–1.74) | <0.001 | | | |  | | | | | 70,354 | | | | | | | 1,466 | | | | | | 1.62 | | | | | | | 2.66  (2.09–3.38) | | | | | | | | | | <0.001 | | | | | | | |  | | | 78,310 | | | | | | 3,838 | | | | | 3.92 | | | 2.52  (2.21–2.86) | | <0.001 | | | | |  |
| ≥160 | 74,310 | 9,921 | | | | 11.69 | | | | 2.15  (2.04–2.26) | <0.001 | | | |  | | | | | 11,400 | | | | | | | 483 | | | | | | 3.35 | | | | | | 4.78  (3.71–6.15) | | | | | | | | | | | <0.001 | | | | | | | |  | | | 15,490 | | | | | | 1,174 | | | | | 6.20 | | | 3.78  (3.30–4.33) | | <0.001 | | | | |  |
| **Diastolic BP, mmHg** | | | | | | | | | | | | | | | | | | | | | | | | | | | | | | | | | | | | | | | | | | | | | | | | | | | | | | | | | | | | | | | | | | | | | | | | | | | | | | | | | | |
| <60 | 38,038 | 1,711 | | | 3.66 | | | | (ref) | | | | | | | |  | | | | 13,826 | | | | | | | 82 | | | | | | | 0.46 | | | | | | | (ref) | | | | | | | | | | | | | | |  | | 9,772 | | | | | | | 154 | | | | | | 1.24 | | | (ref) | | | | | | |  |
| 60-69 | 390,903 | 15,681 | | | 3.23 | | | | 0.98  (0.93–1.03) | | 0.452 | | | | | |  | | | | 152,428 | | | | | | | 861 | | | | | | | 0.44 | | | | | | | 0.90  (0.72–1.13) | | | | | | | 0.348 | | | | | | | |  | | 10,1778 | | | | | | | 2,107 | | | | | | 1.62 | | | 1.30  (1.11–1.54) | | 0.002 | | | | |  |
| 70-79 | 1,148,566 | 49,575 | | | 3.46 | | | | 1.09  (1.04–1.15) | | <0.001 | | | | | | <0.001 | | | | 455,400 | | | | | | | 3,593 | | | | | | | 0.61 | | | | | | | 1.16  (0.93–1.44) | | | | | | | 0.183 | | | | | | | | <0.001 | | 292,708 | | | | | | | 7,630 | | | | | | 2.05 | | | 1.60  (1.37–1.88) | <0.001 | | | <0.001 | | | |
| 80-89 | 1,291,556 | 68,555 | 4.27 | | | | 1.27  (1.21–1.34) | | | | <0.001 | |  | | | | | 458,424 | | | | 5,108 | | | | | | | 0.86 | | | | | | | | 1.45  (1.17–1.81) | | | | | | | <0.001 | | | | | | |  | | | | | | | | 340,411 | | | | | 11,333 | | | | 2.62 | | | | | 1.98  (1.69–2.32) | | | <0.001 | | |  | | | |
| ≥90 | 392,732 | 32,022 | 6.74 | | | | 1.61  (1.54–1.69) | | | | <0.001 | |  | | | | | 89,528 | | | | 2,094 | | | | | | | 1.83 | | | | | | | | 2.44  (1.95–3.05) | | | | | | | <0.001 | | | | | | |  | | | | | | | | 109,651 | | | | | 5,548 | | | | 4.05 | | | | | 2.90  (2.47–3.41) | | | <0.001 | | |  | | | |
| **Non-HDL-c, mg/dL** | | | | | | | | | | | | | | | | | | | | | | | | | | | | | | | | | | | | | | | | | | | | | | | | | | | | | | | | | | | | | | | | | | | | | | | | | | | | | | | | | | |
| <130 | 1,238,057 | 54,758 | 3.57 | | | | | (ref) | | | |  | | | | | | | 519,536 | | | | | 3,306 | | | | | | | | 0.49 | | | | | | (ref) | | | | | | | | | | | | | | | | |  | | | | | 270,462 | | 6,388 | | | | | | | | | | 1.86 | | (ref) | | | | | | |  | |
| 130-159 | 1,048,325 | 52,173 | 4.00 | | | | | 1.10  (1.09–1.12) | | | <0.001 |  | | | | | | | 359,018 | | | | | 3,422 | | | | | | | | 0.74 | | | | | | 1.21  (1.16–1.28) | | | | | | | | | | <0.001 | | | | | | |  | | | | | 283,301 | | 7,872 | | | | | | | | | | 2.18 | | 1.15  (1.11–1.19) | | | | <0.001 | | |  | |
| 160-189 | 651,023 | 37,535 | | 4.64 | | | | 1.28  (1.26–1.29) | | | <0.001 | <0.001 | | | | | | | 198,705 | | | | | 2,879 | | | | | | | | 1.12 | | | | | | 1.60  (1.51–1.68) | | | | | | | | | | <0.001 | | | | | | | <0.001 | | | | | 194,925 | | 7,022 | | | | | | | | | | 2.84 | | 1.43  (1.38–1.48) | | | | <0.001 | | | <0.001 | |
| 190-219 | 240,973 | 16,207 | | 5.44 | | | | 1.50  (1.48–1.53) | | | <0.001 |  | | | | | | | 69,114 | | | | | 1,386 | | | | | | | | 1.56 | | | | | | 1.98  (1.85–2.12) | | | | | | | | | | <0.001 | | | | | | |  | | | | | 77,377 | | 3,646 | | | | | | | | | | 3.74 | | 1.80  (1.72–1.87) | | | | <0.001 | | |  | |
| ≥220 | 83,417 | 6,871 | | 6.74 | | | | 1.86  (1.81–1.91) | | | <0.001 |  | | | | | | | 23,233 | | | | | 745 | | | | | | | | 2.51 | | | | | | 2.89  (2.66–3.14) | | | | | | | | | | <0.001 | | | | | | |  | | | | | 28,255 | | 1,844 | | | | | | | | | | 5.24 | | 2.39  (2.27–2.52) | | | | <0.001 | | |  | |
| **LDL-c, mg/dL** | | | | | | | | | | | | | | | | | | | | | | | | | | | | | | | | | | | | | | | | | | | | | | | | | | | | | | | | | | | | | | | | | | | | | | | | | | | | | | | | | | |
| <70 | 278,230 | 14,752 | | 4.34 | | | | (ref) | | | |  | | | | | | | 106,183 | | | | | 903 | | | | | | | 0.66 | | | | | | | (ref) | | | | | | | | | | | | | | | | | <0.001 | | | | | 63,863 | | | 2,051 | | | | | | | | 2.55 | | | (ref) | | | | |  | | | |
| 70-99 | 882,812 | 39,451 | | 3.60 | | | | 0.94  (0.93–0.96) | | | <0.001 |  | | | | | | | 366,243 | | | | | 2,746 | | | | | | | 0.58 | | | | | | | 0.94  (0.87–1.01) | | | | | | | | | 0.086 | | | | | | | |  |  |  |  |  | 205,636 | | | 5,223 | | | | | | | | 2.00 | | | 0.88  (0.84–0.93) | | <0.001 | | |  | | | |
| 100-129 | 1,165,890 | 56,661 | | 3.91 | | | | 1.01  (0.99–1.03) | | | 0.224 | <0.001 | | | | | | | 420,560 | | | | | 3,931 | | | | | | | 0.72 | | | | | | | 1.05  (0.97–1.13) | | | | | | | | | 0.227 | | | | | | | |  |  |  |  |  | 308,916 | | | 8,755 | | | | | | | | 2.23 | | | 1.00  (0.95–1.05) | | 0.887 | | | <0.001 | | | |
| 130-159 | 672,370 | 37,951 | | 4.55 | | | | 1.13  (1.11–1.16) | | | <0.001 |  | | | | | | | 206,711 | | | | | 2,721 | | | | | | | 1.02 | | | | | | | 1.30  (1.20–1.40) | | | | | | | | | <0.001 | | | | | | | |  |  |  |  |  | 194,553 | | | 6,756 | | | | | | | | 2.74 | | | 1.20  (1.14–1.26) | | <0.001 | | |  | | | |
| ≥160 | 262,493 | 18,729 | | 5.81 | | | | 1.40  (1.37–1.43) | | | <0.001 |  | | | | | | | 69,909 | | | | | 1,437 | | | | | | | 1.60 | | | | | | | 1.82  (1.67–1.98) | | | | | | | | | <0.001 | | | | | | | |  |  |  |  |  | 81,352 | | | 3,987 | | | | | | | | 3.90 | | | 1.64  (1.55–1.73) | | <0.001 | | |  | | | |
| **Triglycerides, mg/dL** | | | | | | | | | | | | | | | | | | | | | | | | | | | | | | | | | | | | | | | | | | | | | | | | | | | | | | | | | | | | | | | | | | | | | | | | | | | | | | | | | | |
| <100 | 1,128,507 | 48,721 | | 3.48 | | | | (ref) | | | | <0.001 | | | | | | | 459,303 | | | | | 2,964 | | | | | | | 0.50 | | | | | | | (ref) | | | | | | | | | | | | | | | | <0.001 | | | | | | 251,995 | | | 5,790 | | | | | | | 1.81 | | | | (ref) | | | | | <0.001 | | | |
| 100-149 | 919,711 | 48,929 | | 4.30 | | | | 1.13  (1.11–1.14) | | | <0.001 |  |  |  |  |  |  |  | 314,417 | | | | | 2,963 | | | | | | | 0.73 | | | | | | | 1.17  (1.11–1.23) | | | | | | | | <0.001 | | | | | | | |  |  |  |  |  |  | 237,294 | | | 6,983 | | | | | | | 2.32 | | | | 1.17  (1.13–1.21) | | <0.001 | | |  | | | |
| 150-199 | 535,691 | 30,409 | | 4.58 | | | | 1.22  (1.20–1.24) | | | <0.001 |  |  |  |  |  |  |  | 174,033 | | | | | 2,026 | | | | | | | 0.90 | | | | | | | 1.25  (1.18–1.32) | | | | | | | | <0.001 | | | | | | | |  |  |  |  |  |  | 152,028 | | | 5,273 | | | | | | | 2.74 | | | | 1.30  (1.25–1.35) | | <0.001 | | |  | | | |
| ≥200 | 677,886 | 39,485 | | 4.69 | | | | 1.33  (1.31–1.34) | | | <0.001 |  |  |  |  |  |  |  | 221,853 | | | | | 3,785 | | | | | | | 1.32 | | | | | | | 1.54  (1.46–1.62) | | | | | | | | <0.001 | | | | | | | |  |  |  |  |  |  | 213,003 | | | 8,726 | | | | | | | 3.24 | | | | 1.42  (1.37–1.47) | | <0.001 | | |  | | | |
| **Abdominal obesity** | | | | | | | | | | | | | | | | | | | | | | | | | | | | | | | | | | | | | | | | | | | | | | | | | | | | | | | | | | | | | | | | | | | | | | | | | | | | | | | | | | |
| Yes | 691,155 | 45,837 | | 5.40 | | | | 1.07  (1.06–1.09) | | | <0.001 | - | | | | | | | 202,871 | | | | 3,400 | | | | | | | 1.30 | | | | | | | | 1.40  (1.34–1.46) | | | | | | | <0.001 | | | | | | | | - | | | | | | | 179,117 | | | 7,107 | | | | | | 3.14 | | | | | 1.17  (1.13–1.20) | | <0.001 | | | | - | | |
| **Regular physical activity** | | | | | | | | | | | | | | | | | | | | | | | | | | | | | | | | | | | | | | | | | | | | | | | | | | | | | | | | | | | | | | | | | | | | | | | | | | | | | | | | | | |
| Yes | 649,643 | 35,989 | | 4.47 | | | | 1.11  (1.09–1.12) | | | <0.001 | - | | | | | | | 185,725 | | | 1,855 | | | | | | | 0.77 | | | | | | | | | 0.98  (0.93–1.03) | | | | | | 0.356 | | | | | | | | - | | | | | | | | 163,302 | | | 4,824 | | | | | 2.32 | | | | | | 1.04  (1.01–1.08) | | 0.007 | | | - | | | |

|  | | | | **50-64 years old** | | | | | | | | | | | | | | | | | | | | | | | | | | | | | | **≥65 years old** | | | | | | | | | | | | | | | | | | | | | | | | | |  | | | |
| --- | --- | --- | --- | --- | --- | --- | --- | --- | --- | --- | --- | --- | --- | --- | --- | --- | --- | --- | --- | --- | --- | --- | --- | --- | --- | --- | --- | --- | --- | --- | --- | --- | --- | --- | --- | --- | --- | --- | --- | --- | --- | --- | --- | --- | --- | --- | --- | --- | --- | --- | --- | --- | --- | --- | --- | --- | --- | --- | --- | --- | --- | --- | --- |
|  | | | | **N** | | **Events** | | | | | | | **IR** | | | | | | **HR**  **(95% CI)** | | | | | | | ***p*** | ***p*_trend_** | | | | | | **N** | | | | | | **Events** | | | | | | | | **IR** | | | **HR**  **(95% CI)** | | | ***p*** | ***p*_trend_** | | | | | | ***p*_interaction_** | | | |
| **BMI, kg/m2** | | | | | | | | | | | | | | | | | | | | | | | | | | | | | | | | | | | | | | | | | | | | | | | | | | | | | | | | | | | | | | | |
| <18.5 | | | | 15,173 | | 1,240 | | | | | | | 7.13 | | | | | | 1.16  (1.10–1.23) | | | | | | | <0.001 | |  | | | | | 15,502 | | | | | | 2,888 | | | | | | | | | 22.12 | | | 1.11  (1.07–1.16) | | <0.001 | | | |  | | | | <0.001 | | |
| 18.5-22.9 | | | | 275,189 | | 19,701 | | | | | | | 5.90 | | | | | | (ref) | | | | | | | | |  | | | | | 137,477 | | | | | | 26,007 | | | | | | | | | 18.80 | | | (ref) | | | | | |  | | | |  | | |
| 23.0-24.9 | | | | 261,460 | | 18,715 | | | | | | | 5.84 | | | | | | 0.99  (0.97–1.01) | | | | | | | 0.208 | | 0.138 | | | | | 92,484 | | | | | | 16,894 | | | | | | | | | 17.14 | | | 0.93  (0.91–0.95) | | <0.001 | | | | <0.001 | | | |  | | |
| 25.0-29.9 | | | | 321,096 | | 23,834 | | | | | | | 6.06 | | | | | | 0.99  (0.97–1.01) | | | | | | | 0.426 | |  | | | | | 92,896 | | | | | | 16,993 | | | | | | | | | 16.90 | | | 0.92  (0.90–0.93) | | <0.001 | | | |  | | | |  | | |
| ≥30.0 | | | | 21,551 | | 1,797 | | | | | | | 6.87 | | | | | | 1.04  (0.99–1.10) | | | | | | | 0.086 | |  | | | | | 5,041 | | | | | | 965 | | | | | | | | | 17.98 | | | 0.95  (0.89–1.01) | | 0.091 | | | |  | | | |  | | |
| **Smoking** | | | | | | | | | | | | | | | | | | | | | | | | | | | | | | | | | | | | | | | | | | | | | | | | | | | | | | | | | | | | | | | |
| Non | | | | 280,077 | 17,575 | | | | | | | | | | | 5.08 | | | (ref) | | | | | | | | | <0.001 | | | | | | 147,649 | 26,342 | | | | | | | | 16.87 | | | | | | (ref) | | | | | | | | <0.001 | | | | <0.001 | | |
| Ex | | | | 288,378 | 16,546 | | | | | | | | | | | 4.64 | | | 0.95  (0.93–0.97) | | <0.001 | | | | | | |  | | | | | | 107,091 | 18,013 | | | | | | | | 15.91 | | | | | | 0.99  (0.97–1.01) | | | | 0.138 | | | |  | | | |  | | |
| Current | | | | 326,014 | 31,166 | | | | | | | | | | | 8.01 | | | 1.77  (1.74–1.80) | | <0.001 | | | | | | |  | | | | | | 88,660 | 19,392 | | | | | | | | 22.41 | | | | | | 1.48  (1.45–1.51) | | | | <0.001 | | | |  | | | |  | | |
| **Hypertension** | | | | | | | | | | | | | | | | | | | | | | | | | | | | | | | | | | | | | | | | | | | | | | | | | | | | | | | | | | | | | | | |
| Yes | | | | 306,635 | | | 27,764 | | | | | | | | | 7.52 | | | 1.33  (1.31–1.35) | | <0.001 | | | | | | | - | | | | | | 167,937 | | | | 34,624 | | | | 20.08 | | | | | | | | | | 1.22  (1.21–1.24) | <0.001 | | | | - | | | | <0.001 | | |
| **Diabetes** | | | | | | | | | | | | | | | | | | | | | | | | | | | | | | | | | | | | | | | | | | | | | | | | | | | | | | | | | | | | | | | |
| Normoglycemia | | | | 481,175 | | | | 31,375 | | | | | | | | 5.30 | | (ref) | | | | | | | | | | | | | <0.001 | | | 180,727 | | | | 31,961 | | | 16.85 | | | | | | | | | | (ref) | | | | | |  | | | | <0.001 | | |
| Prediabetes | | | | 276,911 | | | 19,067 | | | | | | | | | 5.63 | | 1.04  (1.02–1.06) | | | | | <0.001 | | | | | | | |  | | | 98,921 | | | | 17,635 | | | 17.02 | | | | | | | | | | 1.02  (1.00–1.04) | | 0.031 | | | | <0.001 | | | |  | | |
| Diabetes | | | | 136,383 | | | 14,845 | | | | | | | | | 9.25 | | 1.55  (1.52–1.58) | | | | | <0.001 | | | | | | | |  | | | 63,752 | | | 14,151 | | | | 22.61 | | | | | | | | | | 1.36  (1.34–1.39) | | <0.001 | | | |  | | | |  | | |
| **Systolic BP, mmHg** | | | | | | | | | | | | | | | | | | | | | | | | | | | | | | | | | | | | | | | | | | | | | | | | | | | | | | | | | | | | | | | |
| <100 | | | | 13,762 | | | | | 735 | | | | | | | | 4.37 | | (ref) | | | | | | | | | | | |  | | | 4,082 | | 628 | | | | 15.72 | | | | | | | | | | (ref) | | | | | | |  | | | | <0.001 | | |
| 100-119 | | | | 249,867 | | | | | 14,590 | | | | | | | | 4.74 | | 1.13  (1.05–1.22) | | | | | <0.001 | | | | | | |  | | | 74,604 | | 12,236 | | | | 15.76 | | | | | | | | | | 1.03  (0.95–1.11) | | | 0.515 | | | |  | | | |  | | |
| 120-139 | | | | 480,542 | | | | | 35,109 | | | | | | | | 5.98 | | 1.40  (1.30–1.51) | | | | | <0.001 | | | | | | | <0.001 | | | 179,142 | | 32,510 | | | | 17.36 | | | | | | | | | | 1.14  (1.05–1.23) | | | 0.001 | | | | <0.001 | | | |  | | |
| 140-159 | | | | 122,277 | | | | | 11,382 | | | | | | | | 7.75 | | 1.74  (1.61–1.87) | | | | | <0.001 | | | | | | |  | | | 66,173 | | 13,580 | | | | 19.96 | | | | | | | | | | 1.29  (1.19–1.40) | | | <0.001 | | | |  | | | |  | | |
| ≥160 | | | | 28,021 | | | | | 3,471 | | | | | | | | 10.63 | | 2.30  (2.12–2.49) | | | | | <0.001 | | | | | |  | | | | 19,399 | | 4,793 | | | | 25.38 | | | | | | | | | | 1.59  (1.46–1.72) | | | <0.001 | | | |  | | | |  | | |
| **Diastolic BP, mmHg** | | | | | | | | | | | | | | | | | | | | | | | | | | | | | | | | | | | | | | | | | | | | | | | | | | | | | | | | | | | | | | | |
| <60 | 9,430 | | | | | | | | | 568 | | | | | | 4.95 | | | (ref) | | | | | | | | | | | | |  | | 5,010 | | | | 907 | | | | | | 18.46 | | | | | (ref) | | | | | | | |  | | | | <0.001 | | |
| 60-69 | 94,511 | | | | | | | | | 5,552 | | | | | | 4.79 | | | 0.99  (0.91–1.08) | | | | | | 0.388 | | | | | | |  | | 42,186 | | | | 7,161 | | | | | | 16.47 | | | | | 0.95  (0.89–1.02) | | | | 0.140 | | | |  | | | |  | | |
| 70-79 | | 287,757 | | | | | | | | | | 18,809 | | | 5.33 | | | | | 1.12  (1.03–1.21) | | | | | <0.001 | | | | | | | <0.001 | | 112,701 | | | | 19,543 | | | | | | | | 16.64 | | | 0.99  (0.93–1.06) | | | | 0.820 | | | | <0.001 | | | |  | | |
| 80-89 | | 362,238 | | | | | | | | | | 27,392 | | | 6.20 | | | | | 1.31  (1.20–1.42) | | | | | <0.001 | | | | | | |  | | 130,483 | | | | 24,722 | | | | | | | | 18.18 | | | 1.10  (1.03–1.18) | | | | 0.004 | | | |  | | | |  | | |
| ≥90 | | 140,533 | | | | | | | | | | 12,966 | | | 7.68 | | | | | 1.61  (1.48–1.75) | | | | | <0.001 | | | | | | |  | | 53,020 | | | | 11,414 | | | | | | | | 21.11 | | | 1.28  (1.20–1.37) | | | | <0.001 | | | |  | | | |  | | |
| **Non-HDL-c, mg/dL** | | | | | | | | | | | | | | | | | | | | | | | | | | | | | | | | | | | | | | | | | | | | | | | | | | | | | | | | | | | | | | | |
| <130 | | | 302,632 | | | | | | | | 19,719 | | | 5.37 | | | | (ref) | | | | | | | | | | |  | | | | | 145,427 | | | 25,345 | | | | | | | | 17.09 | | | | (ref) | | | | | |  | | | <0.001 | | | | |  |
| 130-159 | | | 296,185 | | | | | | | | 20,637 | | | 5.68 | | | | 1.10  (1.08–1.13) | | | | <0.001 | | | | | | |  | | | | | 109,821 | | | 20,242 | | | | | | | | 17.54 | | | | 1.06  (1.04–1.08) | | | | <0.001 | |  | | |  | | | | |  |
| 160-189 | | | 195,842 | | | | | | | | 15,401 | | | 6.43 | | | | 1.25  (1.23–1.28) | | | | <0.001 | | | | | | | <0.001 | | | | | 61,551 | | | 12,233 | | | | | | | | 18.90 | | | | 1.16  (1.13–1.18) | | | | <0.001 | | <0.001 | | |  | | | | |  |
| 190-219 | | | 74,309 | | | | | | | | 6,775 | | | 7.51 | | | | 1.44  (1.40–1.48) | | | | <0.001 | | | | | | |  | | | | | 20,173 | | | 4,400 | | | | | | | | 21.03 | | | | 1.28  (1.24–1.33) | | | | <0.001 | |  | | |  | | | | |  |
| ≥220 | | | 25,501 | | | | | | | | 2,755 | | | 9.02 | | | | 1.68  (1.62–1.75) | | | | <0.001 | | | | | | |  | | | | | 6,428 | | | 1,527 | | | | | | | | 23.67 | | | | 1.43  (1.36–1.51) | | | | <0.001 | |  | | |  | | | | |  |
| **LDL-c, mg/dL** | | | | | | | | | | | | | | | | | | | | | | | | | | | | | | | | | | | | | | | | | | | | | | | | | | | | | | | | | | | | | | | |
| <70 | | | 74,547 | | | | | | | | 5,696 | | | 6.40 | | | | (ref) | | | | | | | | | | |  | | | | | 33,637 | | | 6,102 | | | | | | | | 18.37 | | | | (ref) | | | | | |  | | | <0.001 | | | | |  |
| 70-99 | | | 216,649 | | | | | | | | 14,684 | | | 5.57 | | | | 0.95  (0.92–0.98) | | | | 0.002 | | | | | | |  | | | | | 94,284 | | | 16,798 | | | | | | | | 17.35 | | | | 0.97  (0.94–1.00) | | | | 0.023 | |  | | |  | | | | |  |
| 100-129 | | | 315,530 | | | | | | | | 21,881 | | | 5.65 | | | | 1.02  (0.99–1.05) | | | | 0.252 | | | | | | | <0.001 | | | | | 120,884 | | | 22,094 | | | | | | | | 17.45 | | | | 1.00  (0.97–1.03) | | | | 0.911 | | <0.001 | | |  | | | | |  |
| 130-159 | | | 202,483 | | | | | | | | 15,317 | | | 6.18 | | | | 1.13  (1.09–1.16) | | | | <0.001 | | | | | | |  | | | | | 68,623 | | | 13,157 | | | | | | | | 18.20 | | | | 1.06  (1.03–1.10) | | | | <0.001 | |  | | |  | | | | |  |
| ≥160 | | | 85,260 | | | | | | | | 7,709 | | | 7.46 | | | | 1.35  (1.30–1.40) | | | | <0.001 | | | | | | |  | | | | | 25,972 | | | 5,596 | | | | | | | | 20.83 | | | | 1.22  (1.17–1.26) | | | | <0.001 | |  | | |  | | | | |  |
| **Triglycerides, mg/dL** | | | | | | | | | | | | | | | | | | | | | | | | | | | | | | | | | | | | | | | | | | | | | | | | | | | | | | | | | | | | | | | |
| <100 | | | 283,754 | | | | | | | | 17,415 | | | 5.02 | | | | (ref) | | | | | | | | | | | <0.001 | | | | | 133,455 | | | 22,552 | | | | | | | | 16.31 | | | | (ref) | | | | | | <0.001 | | | <0.001 | | | | |  |
| 100-149 | | | 261,520 | | | | | | | | 18,901 | | | 5.92 | | | | 1.13  (1.11–1.15) | | | | <0.001 | | | | | | |  |  |  |  |  | 106,480 | | | 20,082 | | | | | | | | 18.24 | | | | 1.10  (1.08–1.13) | | | | <0.001 | |  |  |  |  | | | | |  |
| 150-199 | | | 156,514 | | | | | | | | 12,464 | | | 6.53 | | | | 1.22  (1.19–1.25) | | | | <0.001 | | | | | | |  |  |  |  |  | 53,116 | | | 10,646 | | | | | | | | 19.30 | | | | 1.17  (1.15–1.20) | | | | <0.001 | |  |  |  |  | | | | |  |
| ≥200 | | | 192,681 | | | | | | | | 16,507 | | | 7.05 | | | | 1.28  (1.25–1.30) | | | | <0.001 | | | | | | |  |  |  |  |  | 50,349 | | | 10,467 | | | | | | | | 20.00 | | | | 1.22  (1.20–1.25) | | | | <0.001 | |  |  |  |  | | | | |  |
| **Abdominal obesity** | | | | | | | | | | | | | | | | | | | | | | | | | | | | | | | | | | | | | | | | | | | | | | | | | | | | | | | | | | | | | | | |
| Yes | | | 220,610 | | | | | | | | | 18,008 | | | 6.72 | | | 1.05  (1.03–1.07) | | | | <0.001 | | | | | | | - | | | | | 88,557 | | | 17,322 | | | | | | | | 18.63 | | | | 1.01  (0.99–1.03) | | | | 0.324 | | | - | | | <0.001 | | |  |  |
| **Regular physical activity** | | | | | | | | | | | | | | | | | | | | | | | | | | | | | | | | | | | | | | | | | | | | | | | | | | | | | | | | | | | | | | | |
| Yes | | | 210,912 | | | | | | | | | 14,400 | | | 5.57 | | | 1.09  (1.07–1.11) | | | | <0.001 | | | | | | | - | | | | | 89,704 | | | 14,910 | | | | | | | | 15.36 | | | | 1.15  (1.13–1.18) | | | | <0.001 | | - | | | <0.001 | | | |  |  |

1. **Women**

|  | **Total** | | | | | | | | | | | | | | | | | | **<40 years old** | | | | | | | | | | | | | | | | | | | | | | | | | | | | | | | | **40-49 years old** | | | | | | | | | | | | | | | | | | | | | | | | | |
| --- | --- | --- | --- | --- | --- | --- | --- | --- | --- | --- | --- | --- | --- | --- | --- | --- | --- | --- | --- | --- | --- | --- | --- | --- | --- | --- | --- | --- | --- | --- | --- | --- | --- | --- | --- | --- | --- | --- | --- | --- | --- | --- | --- | --- | --- | --- | --- | --- | --- | --- | --- | --- | --- | --- | --- | --- | --- | --- | --- | --- | --- | --- | --- | --- | --- | --- | --- | --- | --- | --- | --- | --- | --- | --- | --- | --- |
|  | **N** | | **Events** | | | | **IR** | | **HR**  **(95% CI)** | ***p*** | | | | | | ***p*_trend_** | | | **N** | **Events** | | | | | | | | | | | | **IR** | | | | **HR**  **(95% CI)** | | | | | | | | ***p*** | | | | | ***p*_trend_** | | **N** | | | | **Events** | | | | **IR** | | | | | | **HR**  **(95% CI)** | | | | | ***p*** | | | | ***p*_trend_** | | |
| **BMI, kg/m2** | | | | | | | | | | | | | | | | | | | | | | | | | | | | | | | | | | | | | | | | | | | | | | | | | | | | | | | | | | | | | | | | | | | | | | | | | | | | |
| <18.5 | 166,545 | | 3,719 | | | | 1.78 | | 1.17  (1.13–1.21) | <0.001 | | | | | |  | | | 107,261 | 332 | | | | | | | | | | | | 0.24 | | | | 1.09  (0.97–1.23) | | | | | | | | 0.146 | | | |  | | | 29,043 | | | | 279 | | | | 0.76 | | | | | | 0.96  (0.85–1.08) | | | | | 0.479 | | | |  | | |
| 18.5-22.9 | | 1,399,376 | 38,587 | | | | 2.18 | | (ref) | | |  | | | | | | | 456,301 | 1,479 | | | | | | | | | | | | 0.25 | | | | (ref) | | | | | | | | | | | |  | | | 442,392 | | | | 4,800 | | | | 0.85 | | | | | | (ref) | | | | | | | | |  | | |
| 23.0-24.9 | | 648,266 | 27,809 | | | | 3.42 | | 1.00  (0.99–1.02) | 0.959 | | | | 0.009 | | | | | 85,658 | 412 | | | | | | | | | | | | 0.37 | | | | 1.28  (1.15–1.43) | | | | | | | | <0.001 | | | | <0.001 | | | 193,275 | | | | 2,697 | | | | 1.09 | | | | | | 1.17  (1.12–1.23) | | | | | 0.009 | | | | <0.001 | | |
| 25.0-29.9 | | 677,902 | 36,139 | | | | 4.27 | | 1.02  (1.01–1.05) | 0.003 | | | |  | | | | | 67,924 | 412 | | | | | | | | | | | | 0.47 | | | | 1.46  (1.30–1.63) | | | | | | | | <0.001 | | | |  | | | 172,950 | | | | 2,829 | | | | 1.28 | | | | | | 1.25  (1.19–1.31) | | | | | <0.001 | | | |  | | |
| ≥30.0 | | 95,968 | 5,295 | | | | 4.44 | | 1.13  (1.09–1.16) | <0.001 | | | |  | | | | | 15,653 | 172 | | | | | | | | | | | | 0.86 | | | | 2.24  (1.89–2.66) | | | | | | | | <0.001 | | | |  | | | 25,867 | | | | 532 | | | | 1.62 | | | | | | 1.35  (1.23–1.48) | | | | | <0.001 | | | |  | | |
| **Smoking** | | | | | | | | | | | | | | | | | | | | | | | | | | | | | | | | | | | | | | | | | | | | | | | | | | | | | | | | | | | | | | | | | | | | | | | | | | | | |
| Non | | 2,826,772 | 104,908 | | | | 2.95 | | (ref) | | | |  | | | | | | 671,770 | 2,510 | | | | | | | | | | | | 0.29 | | | | (ref) | | | | | | | | | | | |  | | | 816,789 | | | | 10,171 | | | | 0.97 | | | | | | (ref) | | | | | | | | |  | | |
| Ex | | 55,264 | 1,620 | | | | 2.32 | | 1.21  (1.16–1.28) | <0.001 | | | | <0.001 | | | | | 237,94 | 92 | | | | | | | | | | | | 0.30 | | | | 1.07  (0.87–1.32) | | | | | | | 0.529 | | | | | <0.001 | | | 15,479 | | | | 240 | | | | 1.22 | | | | | | 1.33  (1.17–1.51) | | | | | <0.001 | | | | <0.001 | | |
| Current | | 106,021 | 5,021 | | | | 3.82 | | 1.77  (1.72–1.83) | <0.001 | | | |  | | | | | 372,33 | 205 | | | | | | | | | | | | 0.43 | | | | 1.52  (1.31–1.76) | | | | | | | <0.001 | | | | |  | | | 31,259 | | | | 726 | | | | 1.84 | | | | | | 1.85  (1.71–2.00) | | | | | <0.001 | | | |  | | |
| **Hypertension** | | | | | | | | | | | | | | | | | | | | | | | | | | | | | | | | | | | | | | | | | | | | | | | | | | | | | | | | | | | | | | | | | | | | | | | | | | | | |
| Yes | | 614,190 | 54,956 | | | | 7.39 | | 1.33  (1.31–1.35) | <0.001 | | | | | - | | | | 16,487 | | | | | | 216 | | | | | | | 1.03 | | | | 2.23  (1.93–2.59) | | | | | | | <0.001 | | | | | - | | | 92,169 | | | | 2,277 | | | | 1.94 | | | | | | 1.75  (1.67–1.84) | | | | | <0.001 | | | | - | | |
| **Diabetes** | | | | | | | | | | | | | | | | | | | | | | | | | | | | | | | | | | | | | | | | | | | | | | | | | | | | | | | | | | | | | | | | | | | | | | | | | | | | |
| Normo-glycemia | | 2,237,640 | 65,545 | | | | 2.32 | | (ref) | | | | | |  | | | | 651,621 | | | | | | | 2,328 | | | | | | 0.28 | | | | (ref) | | | | | | | | | | | |  | | | 678,705 | | | | 8,173 | | | | 0.94 | | | | | | (ref) | | | | | | | | |  | | |
| Prediabetes | | 562,623 | 25,943 | | | | 3.69 | | 1.01  (1.00–1.03) | 0.101 | | | | | <0.001 | | | | 74,195 | | | | | | 386 | | | | | | | 0.40 | | | | 1.17  (1.05–1.31) | | | | | | | 0.005 | | | | | <0.001 | | | 157,013 | | | | 2,117 | | | | 1.06 | | | | | | 0.99  (0.95–1.04) | | | | | 0.730 | | | | <0.001 | | |
| Diabetes | | 187,794 | 20,061 | | | | 8.99 | | 1.50  (1.47–1.52) | <0.001 | | | | |  | | | | 6,981 | | | | | | 93 | | | | | | | 1.04 | | | | 2.08  (1.68–2.57) | | | | | | | <0.001 | | | | |  | | | 27,809 | | | | 847 | | | | 2.41 | | | | | | 1.86  (1.73–2.00) | | | | | <0.001 | | | |  | | |
| **Systolic BP, mmHg** | | | | | | | | | | | | | | | | | | | | | | | | | | | | | | | | | | | | | | | | | | | | | | | | | | | | | | | | | | | | | | | | | | | | | | | | | | | | |
| <100 | | 175,958 | 2,180 | | | | 0.97 | | (ref) | | | | | |  | | | | 74,455 | | | | | | | 198 | | | | | | 0.21 | | | | (ref) | | | | | | | | | | | |  | | | 60,047 | | | | 531 | | | | 0.69 | | | | | | (ref) | | | | | | | | |  | | |
| 100-119 | | 1,341,005 | 27,924 | | | | 1.64 | | 1.16  (1.11–1.21) | <0.001 | | | | |  | | | | 460,257 | | | | | | | 1,512 | | | | | | 0.26 | | | | 1.18  (1.02–1.37) | | | | | | | 0.026 | | | | |  | | | 435,333 | | | | 4,587 | | | | 0.82 | | | | | | 1.14  (1.04–1.25) | | | | | 0.004 | | | |  | | |
| 120-139 | | 1,184,601 | 54,833 | | | | 3.70 | | 1.38  (1.32–1.44) | <0.001 | | | | | <0.001 | | | | 189,626 | | | | | | | 952 | | | | | | 0.39 | | | | 1.57  (1.34–1.83) | | | | | | | <0.001 | | | | | <0.001 | | | 320,210 | | | | 4,814 | | | | 1.18 | | | | | | 1.49  (1.36–1.63) | | | | | <0.001 | | | | <0.001 | | |
| 140-159 | | 230,960 | 19,843 | | | | 7.08 | | 1.59  (1.52–1.66) | <0.001 | | | | |  | | | | 7,185 | | | | | | | 98 | | | | | | 1.07 | | | | 2.95  (2.30–3.80) | | | | | | | <0.001 | | | | |  | | | 39,805 | | | | 908 | | | | 1.80 | | | | | | 2.02  (1.81–2.25) | | | | | <0.001 | | | |  | | |
| ≥160 | | 55,533 | 6,769 | | | | 10.34 | | 1.92  (1.83–2.02) | <0.001 | | | | |  | | | | 1,274 | | | | | | | 47 | | | | | | 2.92 | | | | 6.58  (4.94–9.52) | | | | | | | <0.001 | | | | |  | | | 8,132 | | | | 297 | | | | 2.90 | | | | | | 3.15  (2.72–3.64) | | | | | <0.001 | | | |  | | |
| **Diastolic BP, mmHg** | | | | | | | | | | | | | | | | | | | | | | | | | | | | | | | | | | | | | | | | | | | | | | | | | | | | | | | | | | | | | | | | | | | | | | | | | | | | |
| <60 | | 112,872 | 1,798 | | | 1.26 | | | (ref) | | | | | | | |  | | 43,083 | | | | | | | 93 | | | | | | 0.17 | | | | (ref) | | | | | | | | | | | |  | | | 9,772 | | | | | 154 | | | | | | | 1.24 | | | | | | (ref) | | | | | |  | |
| 60-69 | | 716,136 | 14,798 | | | 1.63 | | | 1.08  (1.03–1.13) | 0.003 | | | | | | |  | | 248,118 | | | | | | | 762 | | | | | | 0.24 | | | | 1.39  (1.12–1.73) | | | | | | | 0.003 | | | | |  | | | 10,1778 | | | | | 2,107 | | | | | | | 1.62 | | | | | | 1.05  (0.94–1.18) | | | | 0.375 | |  | |
| 70-79 | | 1,119,967 | 36,097 | | | 2.55 | | | 1.23  (1.18–1.29) | <0.001 | | | | | | | <0.001 | | 300,421 | | | | | | | 1,103 | | | | | | 0.29 | | | | 1.58  (1.28–1.95) | | | | | | | 0.183 | | | | | <0.001 | | | 292,708 | | | | | 7,630 | | | | | | | 2.05 | | | | | | 1.21  (1.09–1.35) | | | | <0.001 | | <0.001 | |
| 80-89 | | 811,839 | 40,875 | 4.04 | | | | | 1.41  (1.34–1.48) | <0.001 | | | | | | |  | | 129,439 | | | | | | | 688 | | | | | | 0.41 | | | | 2.02  (1.62–2.51) | | | | | | | <0.001 | | | | |  | | | 340,411 | | | | | 11,333 | | | | | | | | 2.62 | | | | | 1.50  (1.35–1.68) | | | | <0.001 | |  | |
| ≥90 | | 227,243 | 17,981 | 6.48 | | | | | 1.67  (1.59–1.75) | <0.001 | | | | | | |  | | 11,736 | | | | | | | 161 | | | | | | 1.08 | | | | 3.83  (2.94–4.97) | | | | | | | <0.001 | | | | |  | | | 109,651 | | | | | 5,548 | | | | | | | | 4.05 | | | | | 2.17  (1.93–2.45) | | | | <0.001 | |  | |
| **Non-HDL-c, mg/dL** | | | | | | | | | | | | | | | | | | | | | | | | | | | | | | | | | | | | | | | | | | | | | | | | | | | | | | | | | | | | | | | | | | | | | | | | | | | | |
| <130 | 1,351,248 | | 32,712 | 1.91 | | | | (ref) | | | | |  | | | | | | 522,449 | | | | | 1,776 | | | | | | | 0.26 | | | | (ref) | | | | | | | | | | | |  | | | | 430,262 | 4,930 | | | | | | 0.90 | | | | | | | | | | (ref) | | | | | | | |  |
| 130-159 | 864,776 | | 34,317 | 3.16 | | | | 1.04  (1.03–1.06) | | <0.001 | | |  | | | | | | 151,944 | | | | | 653 | | | | | | | 0.33 | | | | 1.04  (0.95–1.14) | | | | | | | 0.397 | | | | |  | | | | 266,540 | 3,473 | | | | | | 1.02 | | | | | | | | | | 1.04  (1.00–1.09) | | | | 0.077 | | | |  |
| 160-189 | 499,740 | | 26,322 | | 4.23 | | | 1.09  (1.07–1.11) | | <0.001 | | | <0.001 | | | | | | 44,082 | | | | 269 | | | | | | | 0.48 | | | | 1.26  (1.10–1.44) | | | | | | | <0.001 | | | | <0.001 | | | | | | 119,919 | | 1,781 | | | | 1.16 | | | | | | | | | 1.09  (1.03–1.15) | | | | | 0.003 | | | | <0.001 | |
| 190-219 | 195,798 | | 12,427 | | 5.13 | | | 1.16  (1.13–1.18) | | <0.001 | | |  | | | | | | 10,652 | | | | 77 | | | | | | | 0.56 | | | | 1.29  (1.02–1.62) | | | | | | | 0.036 | | | |  | | | | | | 35,573 | | 661 | | | | 1.46 | | | | | | | | | 1.26  (1.16–1.37) | | | | | <0.001 | | | |  | |
| ≥220 | 76,495 | | 5,771 | | 6.15 | | | 1.29  (1.26–1.33) | | <0.001 | | |  | | | | | | 3,670 | | | | 32 | | | | | | | 0.68 | | | | 1.50  (1.05–2.14) | | | | | | | 0.026 | | | |  | | | | | | 11,233 | | 292 | | | | 2.05 | | | | | | | | | 1.62  (1.43–1.82) | | | | | <0.001 | | | |  | |
| **LDL-c, mg/dL** | | | | | | | | | | | | | | | | | | | | | | | | | | | | | | | | | | | | | | | | | | | | | | | | | | | | | | | | | | | | | | | | | | | | | | | | | | | | |
| <70 | 197,068 | | 6,079 | | 2.45 | | | (ref) | | | | |  | | | | | 84,138 | | | | 335 | | | | | | | 0.31 | | | | (ref) | | | | | | | | | | | | | | 0.945 | | | 52,888 | | | | 760 | | | | | | | | 1.13 | | | | | (ref) | | | | | | |  | | |
| 70-99 | 820,432 | | 22,471 | | 2.17 | | | 0.95  (0.92–0.98) | | <0.001 | | |  | | | | | 308,718 | | | | 1,048 | | | | | | | 0.26 | | | | 0.82  (0.73–0.93) | | | | | | | 0.002 | | | | | | |  |  |  | 252,255 | | | | 2,993 | | | | | | | | 0.93 | | | | | 0.87  (0.80–0.94) | | | <0.001 | | | |  | | |
| 100-129 | 1,046,503 | | 36,561 | | 2.78 | | | 0.96  (0.92–0.99) | | 0.006 | | | <0.001 | | | | | 245,916 | | | | 942 | | | | | | | 0.30 | | | | 0.83  (0.73–0.94) | | | | | | | 0.004 | | | | | | |  |  |  | 333,502 | | | | 4,002 | | | | | | | | 0.94 | | | | | 0.85  (0.78–0.92) | | | <0.001 | | | | 0.003 | | |
| 130-159 | 622,225 | | 28,834 | | 3.71 | | | 0.99  (0.97–1.02) | | 0.585 | | |  | | | | | 75,250 | | | | 364 | | | | | | | 0.38 | | | | 0.90  (0.78–1.05) | | | | | | | 0.183 | | | | | | |  |  |  | 166,520 | | | | 2,358 | | | | | | | | 1.11 | | | | | 0.93  (0.86–1.01) | | | 0.086 | | | |  | | |
| ≥160 | 301,829 | | 17,604 | | 4.70 | | | 1.06  (1.03–1.09) | | <0.001 | | |  | | | | | 18,775 | | | | 118 | | | | | | | 0.49 | | | | 1.02  (0.82–1.26) | | | | | | | 0.894 | | | | | | |  |  |  | 58,362 | | | | 1,024 | | | | | | | | 1.38 | | | | | 1.06  (0.97–1.17) | | | 0.225 | | | |  | | |
| **Triglycerides, mg/dL** | | | | | | | | | | | | | | | | | | | | | | | | | | | | | | | | | | | | | | | | | | | | | | | | | | | | | | | | | | | | | | | | | | | | | | | | | | | | |
| <100 | 1,676,753 | | 38,769 | | 1.82 | | | (ref) | | | <0.001 | | | | | | | 566,268 | | | | 1,887 | | | | | | | 0.26 | | | | (ref) | | | | | | | | | | | | | <0.001 | | | | 541,019 | | | | 5,988 | | | | | | | 0.87 | | | | | | (ref) | | | | | | | <0.001 | | |
| 100-149 | 749,814 | | 35,485 | | 3.79 | | | 1.10  (1.09–1.12) | | <0.001 |  |  |  |  |  |  |  | 114,564 | | | | 579 | | | | | | | 0.39 | | | | 1.23  (1.12–1.35) | | | | | | <0.001 | | | | | | |  |  |  |  | 201,875 | | | | 2,842 | | | | | | | 1.10 | | | | | | 1.12  (1.07–1.17) | | | <0.001 | | | |  |  |  |
| 150-199 | 309,293 | | 18,869 | | 4.93 | | | 1.15  (1.13–1.17) | | <0.001 |  |  |  |  |  |  |  | 31,110 | | | | 179 | | | | | | | 0.45 | | | | 1.19  (1.02–1.40) | | | | | | 0.031 | | | | | | |  |  |  |  | 69,609 | | | | 1,173 | | | | | | | 1.32 | | | | | | 1.21  (1.14–1.30) | | | <0.001 | | | |  |  |  |
| ≥200 | 252,197 | | 18,426 | | 5.94 | | | 1.27  (1.25–1.30) | | <0.001 |  |  |  |  |  |  |  | 20,855 | | | | 162 | | | | | | | 0.61 | | | | 1.40  (1.18–1.66) | | | | | | <0.001 | | | | | | |  |  |  |  | 51,024 | | | | 1,134 | | | | | | | 1.75 | | | | | | 1.45  (1.36–1.55) | | | <0.001 | | | |  |  |  |
| **Abdominal obesity** | | | | | | | | | | | | | | | | | | | | | | | | | | | | | | | | | | | | | | | | | | | | | | | | | | | | | | | | | | | | | | | | | | | | | | | | | | | | |
| Yes | 485,130 | | 34,791 | | 5.84 | | | 1.08  (1.06–1.09) | | <0.001 | - | | | | | | | 43,127 | | | 346 | | | | | | | 0.63 | | | | | 1.59  (1.41–1.80) | | | | | <0.001 | | | | | | | - | | | | | 93,244 | | | | 1,760 | | | | | | 1.48 | | | | | | | 1.22  (1.16–1.29) | | | <0.001 | | | | | - | |
| **Regular physical activity** | | | | | | | | | | | | | | | | | | | | | | | | | | | | | | | | | | | | | | | | | | | | | | | | | | | | | | | | | | | | | | | | | | | | | | | | | | | | |
| Yes | 471,637 | | 16,373 | | 2.75 | | | 1.15  (1.13–1.17) | | <0.001 | - | | | | | | | 68,411 | | 288 | | | | | | | 0.33 | | | | | | 0.97  (0.85–1.09) | | | | 0.356 | | | | | | | | - | | | | | 141,448 | | | | 1,778 | | | | | 0.98 | | | | | | | | 1.05  (1.00–1.11) | | | 0.041 | | | | - | | |

|  | | | | **50-64 years old** | | | | | | | | | | | | | | | | | | | | | | | | | | | | | | **≥65 years old** | | | | | | | | | | | | | | | | | | | | | | | | | |  | | |
| --- | --- | --- | --- | --- | --- | --- | --- | --- | --- | --- | --- | --- | --- | --- | --- | --- | --- | --- | --- | --- | --- | --- | --- | --- | --- | --- | --- | --- | --- | --- | --- | --- | --- | --- | --- | --- | --- | --- | --- | --- | --- | --- | --- | --- | --- | --- | --- | --- | --- | --- | --- | --- | --- | --- | --- | --- | --- | --- | --- | --- | --- | --- |
|  | | | | **N** | | **Events** | | | | | | | **IR** | | | | | | **HR**  **(95% CI)** | | | | | | | ***p*** | ***p*_trend_** | | | | | | **N** | | | | | | **Events** | | | | | | | | **IR** | | | **HR**  **(95% CI)** | | | ***p*** | ***p*_trend_** | | | | | | ***p*_interaction_** | | |
| **BMI, kg/m2** | | | | | | | | | | | | | | | | | | | | | | | | | | | | | | | | | | | | | | | | | | | | | | | | | | | | | | | | | | | | | | |
| <18.5 | | | | 16,895 | | 552 | | | | | | | 2.61 | | | | | | 1.10  (1.01–1.20) | | | | | | | 0.029 | |  | | | | | 13,346 | | | | | | 2,556 | | | | | | | | | 19.31 | | | 1.17  (1.13–1.22) | | <0.001 | | | |  | <0.001 | | | | |
| 18.5-22.9 | | | | 371,542 | | 11,477 | | | | | | | 2.44 | | | | | | (ref) | | | | | | | | |  | | | | | 129,141 | | | | | | 20,831 | | | | | | | | | 14.40 | | | (ref) | |  | | | |  |  | | | | |
| 23.0-24.9 | | | | 269,396 | | 9,886 | | | | | | | 2.90 | | | | | | 1.07  (1.04–1.10) | | | | | | | <0.001 | | <0.001 | | | | | 99,937 | | | | | | 14,814 | | | | | | | | | 12.83 | | | 0.93  (0.91–0.95) | | <0.001 | | | | <0.001 |  | | | | |
| 25.0-29.9 | | | | 303,745 | | 13,146 | | | | | | | 3.43 | | | | | | 1.13  (1.10–1.16) | | | | | | | <0.001 | |  | | | | | 133,283 | | | | | | 19,752 | | | | | | | | | 12.76 | | | 0.92  (0.90–0.94) | | <0.001 | | | |  |  | | | | |
| ≥30.0 | | | | 38,253 | | 2,035 | | | | | | | 4.26 | | | | | | 1.24  (1.18–1.30) | | | | | | | <0.001 | |  | | | | | 16,195 | | | | | | 2,556 | | | | | | | | | 13.75 | | | 0.96  (0.92–1.00) | | 0.032 | | | |  |  | | | | |
| **Smoking** | | | | | | | | | | | | | | | | | | | | | | | | | | | | | | | | | | | | | | | | | | | | | | | | | | | | | | | | | | | | | | |
| Non | | | | 960,054 | 34,855 | | | | | | | | | | | 2.87 | | | (ref) | | | | | | | | |  | | | | | | 378,159 | 57,372 | | | | | | | | 13.26 | | | | | | (ref) | | | | | | | |  | 0.005 | | | | |
| Ex | | | | 11,883 | 487 | | | | | | | | | | | 3.26 | | | 1.20  (1.10–1.32) | | <0.001 | | | | | | | <0.001 | | | | | | 4,108 | 801 | | | | | | | | 18.21 | | | | | | 1.20  (1.12–1.29) | | | | <0.001 | | | | <0.001 |  | | | | |
| Current | | | | 27,894 | 1,754 | | | | | | | | | | | 5.09 | | | 1.89  (1.80–1.99) | | <0.001 | | | | | | |  | | | | | | 9,635 | 2,336 | | | | | | | | 23.87 | | | | | | 1.65  (1.59–1.72) | | | | <0.001 | | | |  |  | | | | |
| **Hypertension** | | | | | | | | | | | | | | | | | | | | | | | | | | | | | | | | | | | | | | | | | | | | | | | | | | | | | | | | | | | | | | |
| Yes | | | | 293,681 | | | 15,612 | | | | | | | | | 4.25 | | | 1.41  (1.38–1.45) | | <0.001 | | | | | | | - | | | | | | 211,853 | | | | 36,851 | | | | 15.48 | | | | | | | | | | 1.23  (1.21–1.26) | <0.001 | | | | - | <0.001 | | | | |
| **Diabetes** | | | | | | | | | | | | | | | | | | | | | | | | | | | | | | | | | | | | | | | | | | | | | | | | | | | | | | | | | | | | | | |
| Normoglycemia | | | | 680,305 | | | | 22,397 | | | | | | | | 2.60 | | (ref) | | | | | | | | | | | | |  | | | 227,009 | | | | 32,647 | | | 12.45 | | | | | | | | | | (ref) | | | | | |  | <0.001 | | | | |
| Prediabetes | | | | 232,129 | | | 8,591 | | | | | | | | | 2.93 | | 1.01  (0.98–1.04) | | | | | <0.001 | | | | | | | | <0.001 | | | 99,286 | | | | 14,849 | | | 13.09 | | | | | | | | | | 1.01  (0.99–1.03) | | 0.441 | | | | <0.001 |  | | | | |
| Diabetes | | | | 87,397 | | | 6,108 | | | | | | | | | 5.66 | | 1.65  (1.61–1.70) | | | | | <0.001 | | | | | | | |  | | | 65,607 | | | 13,013 | | | | 18.32 | | | | | | | | | | 1.41  (1.38–1.44) | | <0.001 | | | |  |  | | | | |
| **Systolic BP, mmHg** | | | | | | | | | | | | | | | | | | | | | | | | | | | | | | | | | | | | | | | | | | | | | | | | | | | | | | | | | | | | | | |
| <100 | | | | 36,711 | | | | | 874 | | | | | | | | 1.88 | | (ref) | | | | | | | | | | | |  | | | 4,745 | | 577 | | | | 10.59 | | | | | | | | | | (ref) | | | | | | |  | <0.001 | | | | |
| 100-119 | | | | 360,007 | | | | | 10,558 | | | | | | | | 2.31 | | 1.13  (1.06–1.22) | | | | | <0.001 | | | | | | |  | | | 85,408 | | 11,267 | | | | 11.41 | | | | | | | | | | 1.05  (0.96–1.14) | | | 0.295 | | | |  |  | | | | |
| 120-139 | | | | 472,610 | | | | | 18,320 | | | | | | | | 3.07 | | 1.34  (1.25–1.44) | | | | | <0.001 | | | | | | | <0.001 | | | 202,155 | | 30,747 | | | | 13.30 | | | | | | | | | | 1.18  (1.08–1.28) | | | <0.001 | | | | <0.001 |  | | | | |
| 140-159 | | | | 107,665 | | | | | 5,718 | | | | | | | | 4.25 | | 1.67  (1.55–1.80) | | | | | <0.001 | | | | | | |  | | | 76,305 | | 13,119 | | | | 15.26 | | | | | | | | | | 1.30  (1.20–1.41) | | | <0.001 | | | |  |  | | | | |
| ≥160 | | | | 22,838 | | | | | 1,626 | | | | | | | | 5.75 | | 2.30  (2.12–2.49) | | | | | <0.001 | | | | | |  | | | | 23,289 | | 4,799 | | | | 18.92 | | | | | | | | | | 1.52  (1.39–1.66) | | | <0.001 | | | |  |  | | | | |
| **Diastolic BP, mmHg** | | | | | | | | | | | | | | | | | | | | | | | | | | | | | | | | | | | | | | | | | | | | | | | | | | | | | | | | | | | | | | |
| <60 | 24,975 | | | | | | | | | 637 | | | | | | 2.01 | | | (ref) | | | | | | | | | | | | |  | | 5,642 | | | | 715 | | | | | | 11.10 | | | | | (ref) | | | | | | | |  | <0.001 | | | | |
| 60-69 | 183,820 | | | | | | | | | 5,152 | | | | | | 2.21 | | | 1.05  (0.97–1.15) | | | | | | 0.211 | | | | | | |  | | 50,678 | | | | 6,601 | | | | | | 11.30 | | | | | 1.04  (0.96–1.12) | | | | 0.313 | | | |  |  | | | | |
| 70-79 | | 361,493 | | | | | | | | | | 12,378 | | | 2.71 | | | | | 1.21  (1.12–1.31) | | | | | <0.001 | | | | | | | <0.001 | | 130,281 | | | | 18,724 | | | | | | | | 12.50 | | | 1.16  (1.07–1.25) | | | | <0.001 | | | | <0.001 |  | | | | |
| 80-89 | | 323,773 | | | | | | | | | | 13,353 | | | 3.27 | | | | | 1.39  (1.28–1.50) | | | | | <0.001 | | | | | | |  | | 145,613 | | | | 23,467 | | | | | | | | 14.18 | | | 1.29  (1.19–1.39) | | | | <0.001 | | | |  |  | | | | |
| ≥90 | | 105,770 | | | | | | | | | | 5,576 | | | 4.21 | | | | | 1.69  (1.56–1.84) | | | | | <0.001 | | | | | | |  | | 59,688 | | | | 11,002 | | | | | | | | 16.53 | | | 1.47  (1.36–1.58) | | | | <0.001 | | | |  |  | | | | |
| **Non-HDL-c, mg/dL** | | | | | | | | | | | | | | | | | | | | | | | | | | | | | | | | | | | | | | | | | | | | | | | | | | | | | | | | | | | | | | |
| <130 | | | 288,643 | | | | | | | | 9,760 | | | 2.68 | | | | (ref) | | | | | | | | | | |  | | | | | 109,894 | | | 16,246 | | | | | | | | 13.06 | | | | (ref) | | | | | |  | | | <0.001 | | | |  |
| 130-159 | | | 322,271 | | | | | | | | 11,452 | | | 2.81 | | | | 1.04  (1.02–1.07) | | | | 0.002 | | | | | | |  | | | | | 124,021 | | | 18,739 | | | | | | | | 13.19 | | | | 1.04  (1.01–1.06) | | | | 0.001 | |  | | |  | | | |  |
| 160-189 | | | 239,246 | | | | | | | | 9,224 | | | 3.05 | | | | 1.09  (1.06–1.12) | | | | <0.001 | | | | | | | <0.001 | | | | | 96,493 | | | 15,048 | | | | | | | | 13.62 | | | | 1.08  (1.06–1.11) | | | | <0.001 | | <0.001 | | |  | | | |  |
| 190-219 | | | 106,139 | | | | | | | | 4,553 | | | 3.41 | | | | 1.17  (1.13–1.22) | | | | <0.001 | | | | | | |  | | | | | 43,434 | | | 7,136 | | | | | | | | 14.42 | | | | 1.14  (1.11–1.17) | | | | <0.001 | |  | | |  | | | |  |
| ≥220 | | | 43,532 | | | | | | | | 2,107 | | | 3.86 | | | | 1.27  (1.21–1.34) | | | | <0.001 | | | | | | |  | | | | | 18,060 | | | 3,340 | | | | | | | | 16.50 | | | | 1.28  (1.24–1.33) | | | | <0.001 | |  | | |  | | | |  |
| **LDL-c, mg/dL** | | | | | | | | | | | | | | | | | | | | | | | | | | | | | | | | | | | | | | | | | | | | | | | | | | | | | | | | | | | | | | |
| <70 | | | 39,592 | | | | | | | | 1,719 | | | 3.46 | | | | (ref) | | | | | | | | | | |  | | | | | 20,450 | | | 3,265 | | | | | | | | 14.30 | | | | (ref) | | | | | |  | | | <0.001 | | | |  |
| 70-99 | | | 182,016 | | | | | | | | 6,613 | | | 2.88 | | | | 0.95  (0.90–1.00) | | | | 0.060 | | | | | | |  | | | | | 77,443 | | | 11,817 | | | | | | | | 13.48 | | | | 0.98  (0.94–1.02) | | | | 0.301 | |  | | |  | | | |  |
| 100-129 | | | 337,709 | | | | | | | | 11,979 | | | 2.81 | | | | 0.97  (0.92–1.02) | | | | 0.245 | | | | | | | <0.001 | | | | | 129,376 | | | 19,638 | | | | | | | | 13.29 | | | | 1.00  (0.96–1.04) | | | | 0.874 | | <0.001 | | |  | | | |  |
| 130-159 | | | 276,305 | | | | | | | | 10,134 | | | 2.90 | | | | 0.99  (0.94–1.04) | | | | 0.626 | | | | | | |  | | | | | 104,150 | | | 15,978 | | | | | | | | 13.38 | | | | 1.03  (0.99–1.07) | | | | 0.191 | |  | | |  | | | |  |
| ≥160 | | | 164,209 | | | | | | | | 6,651 | | | 3.21 | | | | 1.06  (1.00–1.11) | | | | 0.049 | | | | | | |  | | | | | 60,483 | | | 9,811 | | | | | | | | 14.21 | | | | 1.10  (1.05–1.14) | | | | <0.001 | |  | | |  | | | |  |
| **Triglycerides, mg/dL** | | | | | | | | | | | | | | | | | | | | | | | | | | | | | | | | | | | | | | | | | | | | | | | | | | | | | | | | | | | | | | |
| <100 | | | 443,795 | | | | | | | | 13,624 | | | 2.42 | | | | (ref) | | | | | | | | | | | <0.001 | | | | | 125,671 | | | 17,270 | | | | | | | | 11.95 | | | | (ref) | | | | | | <0.001 | | | <0.001 | | | |  |
| 100-149 | | | 300,007 | | | | | | | | 11,409 | | | 3.01 | | | | 1.09  (1.07–1.12) | | | | <0.001 | | | | | | |  |  |  |  |  | 133,368 | | | 20,655 | | | | | | | | 13.61 | | | | 1.08  (1.06–1.10) | | | | <0.001 | |  |  |  |  | | | |  |
| 150-199 | | | 137,884 | | | | | | | | 5,985 | | | 3.45 | | | | 1.16  (1.13–1.20) | | | | <0.001 | | | | | | |  |  |  |  |  | 70,690 | | | 11,532 | | | | | | | | 14.37 | | | | 1.12  (1.09–1.14) | | | | <0.001 | |  |  |  |  | | | |  |
| ≥200 | | | 118,145 | | | | | | | | 6,078 | | | 4.10 | | | | 1.29  (1.25–1.34) | | | | <0.001 | | | | | | |  |  |  |  |  | 62,173 | | | 11,052 | | | | | | | | 15.76 | | | | 1.21  (1.18–1.24) | | | | <0.001 | |  |  |  |  | | | |  |
| **Abdominal obesity** | | | | | | | | | | | | | | | | | | | | | | | | | | | | | | | | | | | | | | | | | | | | | | | | | | | | | | | | | | | | | | |
| Yes | | | 213,577 | | | | | | | | | 10,686 | | | 3.99 | | | 1.16  (1.13–1.19) | | | | <0.001 | | | | | | | - | | | | | 135,182 | | | 21,999 | | | | | | | | 14.27 | | | | 1.01  (1.00–1.03) | | | | 0.127 | | | - | | | <0.001 | |  |  |
| **Regular physical activity** | | | | | | | | | | | | | | | | | | | | | | | | | | | | | | | | | | | | | | | | | | | | | | | | | | | | | | | | | | | | | | |
| Yes | | | 201,979 | | | | | | | | | 6,853 | | | 2.68 | | | 1.13  (1.10–1.16) | | | | <0.001 | | | | | | | - | | | | | 59,799 | | | 7,454 | | | | | | | | 10.56 | | | | 1.18  (1.15–1.21) | | | | <0.001 | | - | | | <0.001 | | |  |  |

HRs were estimated with adjustment for age, sex, BMI, smoking, alcohol, exercise, hypertension, diabetes, dyslipidemia, and eGFR, excluding the primary exposure variable under analysis. ASCVD, atherosclerotic cardiovascular disease; CI, confidence interval; HR, hazard ratio; IR, incidence rate; N, number; ref, reference; other abbreviations as **Supplementary Table 2**.

**Supplementary Table 4. Associations of individual risk factors with CV death risk by age and sex**

1. **Total population**

|  | | **Total** | | | | | | | | | | | | | | | | | **<40 years old** | | | | | | | | | | | | | | | | | | | | | | | | | | | | | | | | | | | **40-49 years old** | | | | | | | | | | | | | | | | | | | | | | | | | | | | | | | | | | | | | |
| --- | --- | --- | --- | --- | --- | --- | --- | --- | --- | --- | --- | --- | --- | --- | --- | --- | --- | --- | --- | --- | --- | --- | --- | --- | --- | --- | --- | --- | --- | --- | --- | --- | --- | --- | --- | --- | --- | --- | --- | --- | --- | --- | --- | --- | --- | --- | --- | --- | --- | --- | --- | --- | --- | --- | --- | --- | --- | --- | --- | --- | --- | --- | --- | --- | --- | --- | --- | --- | --- | --- | --- | --- | --- | --- | --- | --- | --- | --- | --- | --- | --- | --- | --- | --- | --- | --- | --- | --- | --- | --- | --- |
|  | | **N** | | **Events** | | | | **IR** | | | **HR**  **(95% CI)** | | | ***p*** | | | | ***p*_trend_** | **N** | | | **Events** | | | | | | | | | | **IR** | | | | | **HR**  **(95% CI)** | | | | | ***p*** | | | | | | | | | | ***p*_trend_** | | **N** | | | | | | | | **Events** | | | | | | | **IR** | | | | | | **HR**  **(95% CI)** | | | | | | | ***p*** | | | | | | | ***p*_trend_** | | |
| **BMI, kg/m2** | | | | | | | | | | | | | | | | | | | | | | | | | | | | | | | | | | | | | | | | | | | | | | | | | | | | | | | | | | | | | | | | | | | | | | | | | | | | | | | | | | | | | | | | | | | |
| <18.5 | | 238,814 | | 3,349 | | | 1.13 | | | 1.60  (1.55–1.66) | | | | <0.001 | | | |  | 136,039 | | | 64 | | | | | | | | | | 0.04 | | | | | 1.20  (0.92–1.56) | | | | | 0.171 | | | | <0.001 | | | | | | | | 41,859 | | | | | | | | 120 | | | | | | | 0.23 | | | | | | 1.57  (1.30–1.89) | | | | | | | 0.956 | | | | | | | 0.002 | | |
| 18.5-22.9 | | 2,492,083 | | 23,734 | | | 0.75 | | | (ref) | | | | | | | |  | 878,129 | | | 523 | | | | | | | | | | 0.05 | | | | | (ref) | | | | | | | | |  |  |  |  |  |  |  |  | 700,605 | | | | | | | | 1,467 | | | | | | | 0.16 | | | | | | (ref) | | | | | | | | | | | | | |  |  |  |
| 23.0-24.9 | | 1,530,969 | | 14,530 | | | 0.75 | | | 0.80  (0.78–0.82) | | | | <0.001 | | <0.001 | | | 376,021 | | | 379 | | | | | | | | | | 0.08 | | | | | 1.13  (0.99–1.30) | | | | | 0.075 | | | |  |  |  |  |  |  |  |  | 431,671 | | | | | | | | 1,002 | | | | | | | 0.18 | | | | | | 0.83  (0.76–0.90) | | | | | | | <0.001 | | | | | | |  |  |  |
| 25.0-29.9 | | 1,773,812 | | 17,060 | | | 0.76 | | | 0.78  (0.76–0.80) | | | | <0.001 | |  | | | 435,435 | | | 666 | | | | | | | | | | 0.12 | | | | | 1.35  (1.19–1.53) | | | | | <0.001 | | | |  |  |  |  |  |  |  |  | 487,357 | | | | | | | | 1,408 | | | | | | | 0.23 | | | | | | 0.84  (0.78–0.91) | | | | | | | <0.001 | | | | | | |  |  |  |
| ≥30.0 | | 214,174 | | 2,121 | | | 0.78 | | | 1.02  (0.97–1.06) | | | | 0.512 | |  | | | 76,779 | | | 214 | | | | | | | | | | 0.22 | | | | | 1.71  (2.03–2.41) | | | | | <0.001 | | | |  |  |  |  |  |  |  |  | 56,355 | | | | | | | | 265 | | | | | | | 0.37 | | | | | | 1.24  (1.09–1.42) | | | | | | | 0.002 | | | | | | |  |  |  |
| **Smoking** | | | | | | | | | | | | | | | | | | | | | | | | | | | | | | | | | | | | | | | | | | | | | | | | | | | | | | | | | | | | | | | | | | | | | | | | | | | | | | | | | | | | | | | | | | | |
| Non | | 3,827,796 | | 35,613 | | | 0.73 | | | (ref) | | | | | | | |  | 1,020,523 | | | | | | | | 523 | | | | | 0.04 | | | | | (ref) | | | | | | | | | <0.001 | | | | | | | | 1,041,334 | | | | | | 1,468 | | | | | | | 0.11 | | | | | | | (ref) | | | | | | | | | | | | |  | | | | |
| Ex | | 863,923 | | 8,985 | | | 0.83 | | | 0.97  (0.95–1.00) | | | | 0.043 | | <0.001 | | | 208,465 | | | 211 | | | | | | | | | | 0.08 | | | | | 1.10  (0.93–1.31) | | | | | 0.275 | | | |  |  |  |  |  |  |  |  | 243,998 | | | | | | | 578 | | | | | | | 0.18 | | | | | | 0.94  (0.84–1.05) | | | | | 0.292 | | | | | | | | <0.001 | | | | |
| Current | | 1,558,133 | | 16,196 | | | 0.82 | | | 1.89  (1.85–1.93) | | | | <0.001 | |  | | | 673,415 | | | 1,112 | | | | | | | | | | 0.13 | | | | | 1.91  (1.68–2.17) | | | | | <0.001 | | | |  |  |  |  |  |  |  |  | 432,515 | | | | | | | 2,216 | | | | | | | 0.40 | | | | | | 2.10  (1.92–2.29) | | | | | <0.001 | | | | | | | |  | | | | |
| **Hypertension** | | | | | | | | | | | | | | | | | | | | | | | | | | | | | | | | | | | | | | | | | | | | | | | | | | | | | | | | | | | | | | | | | | | | | | | | | | | | | | | | | | | | | | | | | | | |
| Yes | | 1,395,180 | | 33,223 | | | 1.93 | | | 1.49  (1.46–1.51) | | | <0.001 | | | | | - | 144,432 | | 487 | | | | | | | | | 0.26 | | | | | | 2.47  (2.21–2.77) | | | | | | <0.001 | | | | - | | | | | | | | 270,642 | | | | | 1,470 | | | | | | 0.42 | | | | | | | | | 2.09  (1.95–2.23) | | | | | <0.001 | | | | | | | | | - | | | |
| **Diabetes** | | | | | | | | | | | | | | | | | | | | | | | | | | | | | | | | | | | | | | | | | | | | | | | | | | | | | | | | | | | | | | | | | | | | | | | | | | | | | | | | | | | | | | | | | | | |
| Normo-glycemia | | 4,347,864 | | 31,787 | | | 0.57 | | | (ref) | | | | | | | |  | 1,561,963 | | | | | | 1,317 | | | | | | | | 0.07 | | | | | (ref) | | | | | | | | <0.001 | | | | | | | | 1,216,685 | | | | 2,451 | | | | | | 0.16 | | | | | | | | | | (ref) | | | | | | | | | | | | | <0.001 | | | | |
| Prediabetes | | 1,407,599 | | 15,851 | | | 0.89 | | | 1.03  (1.01–1.05) | | | 0.009 | | <0.001 | | | | 300,804 | | 400 | | | | | | | | | 0.10 | | | | | 1.04  (0.93–1.17) | | | | | | 0.502 | | | | |  |  |  |  |  |  |  |  | 399,548 | | | | 1,114 | | | | | | 0.22 | | | | | | | | | | 1.07  (1.00–1.16) | | | | 0.053 | | | | | | | | |  |  |  |  |  |
| Diabetes | | 494,389 | | 13,156 | | | 2.19 | | | 1.51  (1.48–1.54) | | | <0.001 | |  | | | | 39,636 | | 129 | | | | | | | | | 0.25 | | | | | 1.63  (1.35–1.96) | | | | | | <0.001 | | | | |  |  |  |  |  |  |  |  | 101,614 | | | | 697 | | | | | | 0.54 | | | | | | | | | | 1.92  (1.76–2.10) | | | | <0.001 | | | | | | | | |  |  |  |  |  |
| **Systolic BP, mmHg** | | | | | | | | | | | | | | | | | | | | | | | | | | | | | | | | | | | | | | | | | | | | | | | | | | | | | | | | | | | | | | | | | | | | | | | | | | | | | | | | | | | | | | | | | | | |
| <100 | | 221,402 | | 726 | | | 0.26 | | | (ref) | | | | | | | |  | 88,092 | | | | 15 | | | | | | 0.01 | | | | | | (ref) | | | | | | | | | |  | | | | | | | | 74,010 | | | | 79 | | | | | | 0.08 | | | | | | | | | | (ref) | | | | | | | | | | | |  | | | | | |  |
| 100-119 | | 2,371,006 | | 12,155 | | | 0.40 | | | 1.10  (1.02–1.19) | | | 0.014 | | | | |  | 879,732 | | | | 437 | | | | | | 0.04 | | | | | | 2.04  (1.21–3.42) | | | | | 0.007 | | | | |  | | | | | | | | 721,388 | | | | 1,060 | | | | | | 0.11 | | | | | | | | | | 1.11  (0.88–1.39) | | | | 0.388 | | | | | | | |  | | | | | |  |
| 120-139 | | 2,959,527 | | 30,396 | | | 0.81 | | | 1.35  (1.26–1.46) | | | <0.001 | | <0.001 | | | | 844,366 | | | | 1,047 | | | | | | 0.10 | | | | | | 3.61  (2.16–6.06) | | | | | <0.001 | | | | | <0.001 | | | | | | | | 780,712 | | | | 2,206 | | | | | | 0.22 | | | | | | | | | | 1.74  (1.38–2.18) | | | | <0.001 | | | | | | | | <0.001 | | | | | |  |
| 140-159 | | 568,074 | | 12,517 | | | 1.78 | | | 1.68  (1.56–1.81) | | | <0.001 | |  | | | | 77,539 | | | | 233 | | | | | | 0.23 | | | | | | 6.47  (3.80–11.01) | | | | | <0.001 | | | | |  | | | | | | | | 118,115 | | | | 653 | | | | | | 0.43 | | | | | | | | | | 3.01  (2.38–3.83) | | | | <0.001 | | | | | | | |  | | | | | |  |
| ≥160 | | 129,843 | | 5,000 | | | 3.19 | | | 2.31  (2.14–2.50) | | <0.001 | | |  | | | | 12,674 | | | | 114 | | | | | | 0.70 | | | | | 17.09  (9.86–29.64) | | | | | | <0.001 | | | | |  | | | | | | | | | 23,622 | | | | 264 | | | | | | 0.88 | | | | | | | | | | 5.79  (4.48–7.48) | | | | <0.001 | | | | | | | | |  | | | | |
| **Diastolic BP, mmHg** | | | | | | | | | | | | | | | | | | | | | | | | | | | | | | | | | | | | | | | | | | | | | | | | | | | | | | | | | | | | | | | | | | | | | | | | | | | | | | | | | | | | | | | | | | | |
| <60 | 150,910 | | 801 | | 0.42 | | | | (ref) | | | | | | | | | <0.001 | | 56,909 | | | | | | 15 | | | | | 0.02 | | | | | | (ref) | | | | | | | | | | <0.001 | | | | | | | | 48,944 | | 49 | | | | | | | | | | | 0.08 | | | | | | | | (ref) | | | | | | | | | | | | | <0.001 | | |
| 60-69 | 1,107,039 | | 6,395 | | 0.45 | | | | 0.98  (0.91–1.05) | | | | 0.499 | | | | |  |  | 400,546 | | | | | | 164 | | | | | 0.03 | | | | | | 1.31  (0.77–2.22) | | | | | | | 0.318 | | |  |  |  |  |  |  |  |  | 335,298 | | 391 | | | | | | | | | | | 0.09 | | | | | | | | 1.03  (0.76–1.38) | | | | | 0.859 | | | | | | | |  |  |  |
| 70-79 | 2,268,533 | | 17,765 | | 0.62 | | | | 1.08  (1.01–1.16) | | | | 0.028 | | | | |  |  | 755,821 | | | | | | 502 | | | | | 0.05 | | | | | | 1.64  (0.98–2.75) | | | | | | | 0.061 | | |  |  |  |  |  |  |  |  | 620,480 | | 1,162 | | | | | | | | | | | 0.15 | | | | | | | | 1.36  (1.02–1.82) | | | | | 0.035 | | | | | | | |  |  |  |
| 80-89 | 2,103,395 | | 23,757 | | 0.89 | | | | 1.30  (1.21–1.39) | | | | <0.001 | | | | |  |  | 587,863 | | | | | | 802 | | | | | 0.11 | | | | | | 2.63  (1.57–4.40) | | | | | | | <0.001 | | |  |  |  |  |  |  |  |  | 553,425 | | 1,691 | | | | | | | | | | | 0.24 | | | | | | | | 1.90  (1.42–2.53) | | | | | <0.001 | | | | | | | |  |  |  |
| ≥90 | 619,975 | | 12,076 | | 1.56 | | | | 1.69  (1.57–1.81) | | | | <0.001 | | | | |  |  | 101,264 | | | | | | 363 | | | | | 0.28 | | | | | | 5.13  (3.04–8.67) | | | | | | | <0.001 | | |  |  |  |  |  |  |  |  | 159,700 | | 969 | | | | | | | | | | | 0.48 | | | | | | | | 3.40  (2.55–4.55) | | | | | <0.001 | | | | | | | |  |  |  |
| **Non-HDL-c, mg/dL** | | | | | | | | | | | | | | | | | | | | | | | | | | | | | | | | | | | | | | | | | | | | | | | | | | | | | | | | | | | | | | | | | | | | | | | | | | | | | | | | | | | | | | | | | | | |
| <130 | 2,589,305 | | 21,444 | | 0.65 | | | | (ref) | | | | | | | | <0.001 | | | 1,041,985 | | | | | 681 | | | | | | 0.05 | | | | | | (ref) | | | | | | | | | | | | | | <0.001 | | | | 700,724 | 1,528 | | | | | | | | | | | | | | | | 0.17 | | | | (ref) | | | | | | | | | | | | | | <0.001 | |
| 130-159 | 1,913,101 | | 18,215 | | 0.75 | | | | 0.95  (0.94–0.97) | | | | <0.001 | | | |  |  |  | 510,962 | | | | | 502 | | | | | | 0.08 | | | | | | 1.01  (0.89–1.13) | | | | | | 0.914 | | | | | | | |  |  |  |  | 549,841 | 1,174 | | | | | | | | | | | | | | | | 0.17 | | | | 0.83  (0.77–0.90) | | | | | | | | <0.001 | | | | | |  |  |
| 160-189 | 1,150,763 | | 12,864 | | | 0.88 | | | 1.03  (1.00–1.05) | | | | 0.020 | | | |  |  |  | 242,787 | | | | | 395 | | | | | | 0.13 | | | | | | 1.28  (1.12–1.47) | | | | | | <0.001 | | | | | | | |  |  |  |  | 314,844 | 867 | | | | | | | | | | | | | | | | 0.21 | | | | 0.93  (0.85–1.01) | | | | | | | | 0.096 | | | | | |  |  |
| 190-219 | 436,771 | | 5,695 | | | 1.03 | | | 1.14  (1.11–1.18) | | | | <0.001 | | | |  |  |  | 79,766 | | | | | 175 | | | | | | 0.17 | | | | | | 1.46  (1.23–1.75) | | | | | | <0.001 | | | | | | | |  |  |  |  | 112,950 | 442 | | | | | | | | | | | | | | | | 0.31 | | | | 1.17  (1.05–1.31) | | | | | | | | 0.004 | | | | | |  |  |
| ≥220 | 159,912 | | 2,576 | | | 1.28 | | | 1.36  (1.30–1.42) | | | | <0.001 | | | |  |  |  | 26,903 | | | | | 93 | | | | | | 0.27 | | | | | | 2.05  (1.63–2.57) | | | | | | <0.001 | | | | | | | |  |  |  |  | 39,488 | 251 | | | | | | | | | | | | | | | | 0.50 | | | | 1.73  (1.51–1.98) | | | | | | | | <0.001 | | | | | |  |  |
| **LDL-c, mg/dL** | | | | | | | | | | | | | | | | | | | | | | | | | | | | | | | | | | | | | | | | | | | | | | | | | | | | | | | | | | | | | | | | | | | | | | | | | | | | | | | | | | | | | | | | | | | |
| <70 | 475,298 | | 5,299 | | | 0.89 | | | (ref) | | | | | | | | 0.059 | | | 190,321 | | | | | 191 | | | | | 0.08 | | | | | | | (ref) | | | | | | | | | | | | | | 0.002 | | | | 116,751 | | 475 | | | | | | | | | | | | | | 0.32 | | | | | (ref) | | | | | | | | | | | | | 0.926 | | |
| 70-99 | 1,703,244 | | 14,628 | | | 0.68 | | | 0.90  (0.87–0.92) | | | | <0.001 | | | |  |  |  | 674,961 | | | | | 495 | | | | | 0.06 | | | | | | | 0.80  (0.68–0.94) | | | | | 0.008 | | | | | | | | |  |  |  |  | 457,891 | | 1,032 | | | | | | | | | | | | | | 0.18 | | | | | 0.71  (0.64–0.80) | | | | | | | <0.001 | | | | | |  |  |  |
| 100-129 | 2,212,393 | | 19,950 | | | 0.71 | | | 0.87  (0.84–0.89) | | | | <0.001 | | | |  |  |  | 666,476 | | | | | 609 | | | | | 0.07 | | | | | | | 0.82  (0.70–0.97) | | | | | 0.020 | | | | | | | | |  |  |  |  | 642,418 | | 1,364 | | | | | | | | | | | | | | 0.17 | | | | | 0.67  (0.60–0.74) | | | | | | | <0.001 | | | | | |  |  |  |
| 130-159 | 1,294,595 | | 13,486 | | | 0.82 | | | 0.89  (0.86–0.92) | | | | <0.001 | | | |  |  |  | 281,961 | | | | | 358 | | | | | 0.10 | | | | | | | 0.91  (0.76–1.09) | | | | | 0.315 | | | | | | | | |  |  |  |  | 361,073 | | 877 | | | | | | | | | | | | | | 0.19 | | | | | 0.70  (0.63–0.79) | | | | | | | <0.001 | | | | | |  |  |  |
| ≥160 | 564,322 | | 7,431 | | | 1.04 | | | 1.02  (0.98–1.06) | | | | 0.334 | | | |  |  |  | 88,684 | | | | | 193 | | | | | 0.17 | | | | | | | 1.33  (1.08–1.62) | | | | | 0.007 | | | | | | | | |  |  |  |  | 139,714 | | 514 | | | | | | | | | | | | | | 0.29 | | | | | 0.98  (0.86–1.11) | | | | | | | 0.728 | | | | | |  |  |  |
| **Triglycerides, mg/dL** | | | | | | | | | | | | | | | | | | | | | | | | | | | | | | | | | | | | | | | | | | | | | | | | | | | | | | | | | | | | | | | | | | | | | | | | | | | | | | | | | | | | | | | | | | | |
| <100 | 2,805,260 | | 19,577 | | | 0.55 | | | (ref) | | | | | | | | <0.001 | | | 1,025,571 | | | | | 577 | | | | | 0.04 | | | | | | | (ref) | | | | | | | | | | | | | <0.001 | | | | | 793,014 | | 1,264 | | | | | | | | | | | | | 0.12 | | | | | | (ref) | | | | | | | | | | | | <0.001 | | | |
| 100-149 | 1,669,525 | | 19,264 | | | 0.91 | | | 1.10  (1.08–1.12) | | | | <0.001 | | | |  |  |  | 428,981 | | | | | 437 | | | | | 0.08 | | | | | | | 1.13  (1.00–1.29) | | | | 0.057 | | | | | | | | |  |  |  |  |  | 439,169 | | 1,128 | | | | | | | | | | | | | 0.20 | | | | | | 1.17  (1.08–1.27) | | | | | | <0.001 | | | | | |  |  |  |  |
| 150-199 | 844,984 | | 10,496 | | | 0.98 | | | 1.13  (1.10–1.15) | | | | <0.001 | | | |  |  |  | 205,143 | | | | | 306 | | | | | 0.12 | | | | | | | 1.30  (1.12–1.51) | | | | <0.001 | | | | | | | | |  |  |  |  |  | 221,637 | | 685 | | | | | | | | | | | | | 0.24 | | | | | | 1.16  (1.05–1.28) | | | | | | 0.003 | | | | | |  |  |  |  |
| ≥200 | 930,083 | | 11,457 | | | 0.97 | | | 1.23  (1.20–1.26) | | | | <0.001 | | | |  |  |  | 242,708 | | | | | 526 | | | | | 0.17 | | | | | | | 1.48  (1.29–1.69) | | | | <0.001 | | | | | | | | |  |  |  |  |  | 264,027 | | 1,185 | | | | | | | | | | | | | 0.35 | | | | | | 1.38  (1.26–1.50) | | | | | | <0.001 | | | | | |  |  |  |  |
| **Abdominal obesity** | | | | | | | | | | | | | | | | | | | | | | | | | | | | | | | | | | | | | | | | | | | | | | | | | | | | | | | | | | | | | | | | | | | | | | | | | | | | | | | | | | | | | | | | | | | |
| Yes | 1,176,285 | | 17,584 | | | 1.19 | | | 0.97  (0.96–0.99) | | | | 0.003 | | | | - | | | 245,998 | | | | 502 | | | | | 0.16 | | | | | | | | 1.38  (1.24–1.54) | | | <0.001 | | | | | | | | | - | | | | | | 272,361 | | 1,011 | | | | | | | | | | | 0.29 | | | | | | | | 1.10  (1.02–1.18) | | | | 0.016 | | | | | | | - | | | | |
| **Regular physical activity** | | | | | | | | | | | | | | | | | | | | | | | | | | | | | | | | | | | | | | | | | | | | | | | | | | | | | | | | | | | | | | | | | | | | | | | | | | | | | | | | | | | | | | | | | | | |
| Yes | 1,121,280 | | 10,364 | | | 0.73 | | | 1.23  (1.21–1.26) | | | | <0.001 | | | | - | | | 254,136 | | | 277 | | | | | 0.08 | | | | | | | | | 0.97  (0.85–1.10) | | 0.648 | | | | | | | | | - | | | | | | | 304,750 | | 720 | | | | | | | | | 0.18 | | | | | | | | | | 1.08  (1.00–1.17) | | | 0.055 | | | | | | | - | | | | | |

|  | | | | **50-64 years old** | | | | | | | | | | | | | | | | | | | | | | | | | | | | | | **≥65 years old** | | | | | | | | | | | | | | | | | | | | | | | | | | | | | | | | | | | |  | | | |
| --- | --- | --- | --- | --- | --- | --- | --- | --- | --- | --- | --- | --- | --- | --- | --- | --- | --- | --- | --- | --- | --- | --- | --- | --- | --- | --- | --- | --- | --- | --- | --- | --- | --- | --- | --- | --- | --- | --- | --- | --- | --- | --- | --- | --- | --- | --- | --- | --- | --- | --- | --- | --- | --- | --- | --- | --- | --- | --- | --- | --- | --- | --- | --- | --- | --- | --- | --- | --- | --- | --- | --- | --- | --- |
|  | | | | **N** | | **Events** | | | | | | | **IR** | | | | | | **HR**  **(95% CI)** | | | | | | | ***p*** | ***p*_trend_** | | | | | | **N** | | | | | | **Events** | | | | | | | | **IR** | | | **HR**  **(95% CI)** | | | | | | | ***p*** | | | | | ***p*_trend_** | | | | | | | | ***p*_interaction_** | | | |
| **BMI, kg/m2** | | | | | | | | | | | | | | | | | | | | | | | | | | | | | | | | | | | | | | | | | | | | | | | | | | | | | | | | | | | | | | | | | | | | | | | | | |
| <18.5 | | | | 32,068 | | 462 | | | | | | | 1.18 | | | | | | 1.92  (1.74–2.11) | | | | | | | <0.001 | | <0.001 | | | | | 28,848 | | | | | | 2,703 | | | | | | | | | 9.83 | | | 1.48  (1.42–1.54) | | | | | | <0.001 | | | | | <0.001 | | | | | | | | <0.001 | | | |
| 18.5-22.9 | | | | 646,731 | | 4,727 | | | | | | | 0.58 | | | | | | (ref) | | | | | | | | |  |  |  |  |  | 266,618 | | | | | | 17,017 | | | | | | | | | 5.71 | | | (ref) | | | | | | | | | | |  |  |  |  |  |  |  |  |  | | | |
| 23.0-24.9 | | | | 530,856 | | 3,583 | | | | | | | 0.53 | | | | | | 0.82  (0.78–0.85) | | | | | | | <0.001 | |  |  |  |  |  | 192,421 | | | | | | 9,566 | | | | | | | | | 4.24 | | | 0.80  (0.78–0.82) | | | | | | <0.001 | | | | |  |  |  |  |  |  |  |  |  | | | |
| 25.0-29.9 | | | | 624,841 | | 4,546 | | | | | | | 0.57 | | | | | | 0.80  (0.77–0.84) | | | | | | | <0.001 | |  |  |  |  |  | 226,179 | | | | | | 10,440 | | | | | | | | | 3.88 | | | 0.75  (0.74–0.77) | | | | | | <0.001 | | | | |  |  |  |  |  |  |  |  |  | | | |
| ≥30.0 | | | | 59,804 | | 549 | | | | | | | 0.73 | | | | | | 1.02  (0.93–1.11) | | | | | | | 0.754 | |  |  |  |  |  | 21,236 | | | | | | 1,093 | | | | | | | | | 4.32 | | | 0.86  (0.81–0.92) | | | | | | <0.001 | | | | |  |  |  |  |  |  |  |  |  | | | |
| **Smoking** | | | | | | | | | | | | | | | | | | | | | | | | | | | | | | | | | | | | | | | | | | | | | | | | | | | | | | | | | | | | | | | | | | | | | | | | | |
| Non | | | | 1,240,131 | 6,284 | | | | | | | | | | | 0.40 | | | (ref) | | | | | | | | | <0.001 | | | | | | 525,808 | 27,338 | | | | | | | | 4.42 | | | | | | | | | | | (ref) | | | | | | | | <0.001 | | | | | | | | <0.001 | | | |
| Ex | | | | 300,261 | 2,194 | | | | | | | | | | | 0.58 | | | 0.94  (0.88–0.99) | | 0.020 | | | | | | |  |  |  |  |  |  | 111,199 | 6,002 | | | | | | | | 4.85 | | | | | | | | | | 1.01  (0.98–1.05) | | | | 0.443 | | | | |  |  |  |  |  |  |  |  |  | | | |
| Current | | | | 353,908 | 5,389 | | | | | | | | | | | 1.23 | | | 2.14  (2.04–2.25) | | <0.001 | | | | | | |  |  |  |  |  |  | 98,295 | 7,479 | | | | | | | | 7.22 | | | | | | | | | | 1.66  (1.61–1.71) | | | | <0.001 | | | | |  |  |  |  |  |  |  |  |  | | | |
| **Hypertension** | | | | | | | | | | | | | | | | | | | | | | | | | | | | | | | | | | | | | | | | | | | | | | | | | | | | | | | | | | | | | | | | | | | | | | | | | |
| Yes | | | | 600,316 | | | 6,651 | | | | | | | | | 0.88 | | | 1.67  (1.62–1.73) | | <0.001 | | | | | | | - | | | | | | 379,790 | | | | 24,615 | | | | 5.66 | | | | | | | | | | 1.35  (1.33–1.38) | | | | | <0.001 | | | | | - | | | | | | | | <0.001 | | | |
| **Diabetes** | | | | | | | | | | | | | | | | | | | | | | | | | | | | | | | | | | | | | | | | | | | | | | | | | | | | | | | | | | | | | | | | | | | | | | | | | |
| Normoglycemia | | | | 1,161,480 | | | | 6,871 | | | | | | | | 0.46 | | (ref) | | | | | | | | | | | | | <0.001 | | | 407,736 | | | | 21,148 | | | 4.46 | | | | | | | | | | (ref) | | | | | | | | | |  | | | | | | | | | <0.001 | | | |
| Prediabetes | | | | 509,040 | | | 3,659 | | | | | | | | | 0.57 | | 1.04  (1.00–1.09) | | | | | 0.049 | | | | | | | |  |  |  | 198,207 | | | | 10,678 | | | 4.68 | | | | | | | | | | 1.03  (1.00–1.05) | | | | | | 0.039 | | | | | | | | <0.001 | | | | |  | | | |
| Diabetes | | | | 223,780 | | | 3,337 | | | | | | | | | 1.20 | | 1.79  (1.72–1.87) | | | | | <0.001 | | | | | | | |  |  |  | 129,359 | | | 8,993 | | | | 6.29 | | | | | | | | | | 1.44  (1.41–1.48) | | | | | | <0.001 | | | | | | | |  | | | | |  | | | |
| **Systolic BP, mmHg** | | | | | | | | | | | | | | | | | | | | | | | | | | | | | | | | | | | | | | | | | | | | | | | | | | | | | | | | | | | | | | | | | | | | | | | | | |
| <100 | | | | 50,473 | | | | | 204 | | | | | | | | 0.32 | | (ref) | | | | | | | | | | | |  | | | 8,827 | | 428 | | | | 4.35 | | | | | | | | | | (ref) | | | | | | | | | |  | | | | | | | | | | | <0.001 | | |
| 100-119 | | | | 609,874 | | | | | 2,985 | | | | | | | | 0.38 | | 1.09  (0.95–1.26) | | | | | 0.238 | | | | | | |  | | | 160,012 | | 7,673 | | | | 4.17 | | | | | | | | | | 0.96  (0.87–1.06) | | | | | | | 0.454 | | | | | | | | |  | | | | |  | | |
| 120-139 | | | | 953,152 | | | | | 7,038 | | | | | | | | 0.58 | | 1.47  (1.28–1.69) | | | | | <0.001 | | | | | | | <0.001 | | | 381,297 | | 20,105 | | | | 4.56 | | | | | | | | | | 1.05  (0.95–1.15) | | | | | | | 0.346 | | | | | | | | | <0.001 | | | | |  | | |
| 140-159 | | | | 229,942 | | | | | 2,638 | | | | | | | | 0.91 | | 2.09  (1.81–2.41) | | | | | <0.001 | | | | | | |  | | | 142,478 | | 8,993 | | | | 5.51 | | | | | | | | | | 1.22  (1.11–1.35) | | | | | | | <0.001 | | | | | | | | |  | | | | |  | | |
| ≥160 | | | | 50,859 | | | | | 1,002 | | | | | | | | 1.58 | | 1.94  (1.66–2.28) | | | | | <0.001 | | | | | |  | | | | 42,688 | | 3,620 | | | | 7.61 | | | | | | | | | | 1.56  (1.41–1.72) | | | | | | | <0.001 | | | | | | | | |  | | | | |  | | |
| **Diastolic BP, mmHg** | | | | | | | | | | | | | | | | | | | | | | | | | | | | | | | | | | | | | | | | | | | | | | | | | | | | | | | | | | | | | | | | | | | | | | | | | |
| <60 | 34,405 | | | | | | | | | 164 | | | | | | 0.38 | | | (ref) | | | | | | | | | | | | |  | | 10,652 | | | | 573 | | | | | | 4.83 | | | | | (ref) | | | | | | | |  | | | | | | | | | |  | | | | <0.001 | | |
| 60-69 | 278,331 | | | | | | | | | 1,317 | | | | | | 0.37 | | | 0.94  (0.80–1.11) | | | | | | 0.460 | | | | | | |  | | 92,864 | | | | 4,523 | | | | | | 4.25 | | | | | 0.96  (0.88–1.05) | | | | | | | | 0.398 | | | | | | | | | |  | | | |  | | |
| 70-79 | | 649,250 | | | | | | | | | | 3,840 | | | 0.47 | | | | | 1.07  (0.92–1.25) | | | | | 0.390 | | | | | | | <0.001 | | 242,982 | | | | 12,261 | | | | | | | | 4.37 | | | | | | | | | | 1.03  (0.94–1.12) | | | 0.559 | | | <0.001 | | | | | | |  | | | | |
| 80-89 | | 686,011 | | | | | | | | | | 5,603 | | | 0.64 | | | | | 1.39  (1.19–1.62) | | | | | <0.001 | | | | | | |  | | 276,096 | | | | 15,661 | | | | | | | | 4.92 | | | | | | | | | | 1.15  (1.06–1.25) | | | 0.001 | | |  | | | | | | |  | | | | |
| ≥90 | | 246,303 | | | | | | | | | | 2,943 | | | 0.95 | | | | | 1.94  (1.66–2.28) | | | | | <0.001 | | | | | | |  | | 112,708 | | | | 7,801 | | | | | | | | 6.07 | | | | | | | | | | 1.40  (1.29–1.52) | | | <0.001 | | |  | | | | | | |  | | | | |
| **Non-HDL-c, mg/dL** | | | | | | | | | | | | | | | | | | | | | | | | | | | | | | | | | | | | | | | | | | | | | | | | | | | | | | | | | | | | | | | | | | | | | | | | | |
| <130 | | | 591,275 | | | | | | | | 4,612 | | | 0.62 | | | | (ref) | | | | | | | | | | |  | | | | | 255,321 | | | 14,623 | | | | | | | | 5.12 | | | | | | | | | | (ref) | | | | | | | |  | | | | | <0.001 | | | | |  |
| 130-159 | | | 618,456 | | | | | | | | 4,110 | | | 0.52 | | | | 0.93  (0.89–0.97) | | | | <0.001 | | | | | | |  | | | | | 233,842 | | | 12,429 | | | | | | | | 4.58 | | | | | | | | | | 0.97  (0.94–0.99) | | | 0.006 | | | | |  | | | | |  | | | | |  |
| 160-189 | | | 435,088 | | | | | | | | 2,983 | | | 0.54 | | | | 0.99  (0.94–1.03) | | | | 0.579 | | | | | | | <0.001 | | | | | 158,044 | | | 8,619 | | | | | | | | 4.65 | | | | | | | | | | 1.03  (1.00–1.06) | | | 0.036 | | | | | <0.001 | | | | |  | | | | |  |
| 190-219 | | | 180,448 | | | | | | | | 1,453 | | | 0.63 | | | | 1.17  (1.11–1.25) | | | | <0.001 | | | | | | |  | | | | | 63,607 | | | 3,625 | | | | | | | | 4.85 | | | | | | | | | | 1.10  (1.06–1.14) | | | <0.001 | | | | |  | | | | |  | | | | |  |
| ≥220 | | | 69,033 | | | | | | | | 709 | | | 0.81 | | | | 1.50  (1.39–1.63) | | | | <0.001 | | | | | | |  | | | | | 24,488 | | | 1,523 | | | | | | | | 5.33 | | | | | | | | | | 1.20  (1.14–1.27) | | | <0.001 | | | | |  | | | | |  | | | | |  |
| **LDL-c, mg/dL** | | | | | | | | | | | | | | | | | | | | | | | | | | | | | | | | | | | | | | | | | | | | | | | | | | | | | | | | | | | | | | | | | | | | | | | | | |
| <70 | | | 114,139 | | | | | | | | 1,302 | | | 0.92 | | | | (ref) | | | | | | | | | | |  | | | | | 54,087 | | | 3,331 | | | | | | | | 5.65 | | | | | | | | | | (ref) | | | | | | | |  | | | | | <0.001 | | | | |  |
| 70-99 | | | 398,665 | | | | | | | | 3,295 | | | 0.66 | | | | 0.90  (0.85–0.96) | | | | 0.002 | | | | | | |  | | | | | 171,727 | | | 9,806 | | | | | | | | 5.06 | | | | | | | | | | 0.92  (0.88–0.96) | | | <0.001 | | | | |  | | | | |  | | | | |  |
| 100-129 | | | 653,239 | | | | | | | | 4,401 | | | 0.53 | | | | 0.83  (0.78–0.89) | | | | <0.001 | | | | | | | 0.709 | | | | | 250,260 | | | 13,576 | | | | | | | | 4.70 | | | | | | | | | | 0.90  (0.87–0.94) | | | <0.001 | | | | | <0.001 | | | | |  | | | | |  |
| 130-159 | | | 478,788 | | | | | | | | 3,022 | | | 0.50 | | | | 0.84  (0.78–0.90) | | | | <0.001 | | | | | | |  | | | | | 172,773 | | | 9,229 | | | | | | | | 4.56 | | | | | | | | | | 0.93  (0.89–0.97) | | | <0.001 | | | | |  | | | | |  | | | | |  |
| ≥160 | | | 249,469 | | | | | | | | 1,847 | | | 0.58 | | | | 1.05  (0.97–1.13) | | | | 0.207 | | | | | | |  | | | | | 86,455 | | | 4,877 | | | | | | | | 4.79 | | | | | | | | | | 1.01  (0.97–1.06) | | | 0.558 | | | | |  | | | | |  | | | | |  |
| **Triglycerides, mg/dL** | | | | | | | | | | | | | | | | | | | | | | | | | | | | | | | | | | | | | | | | | | | | | | | | | | | | | | | | | | | | | | | | | | | | | | | | | |
| <100 | | | 727,549 | | | | | | | | 4,200 | | | 0.46 | | | | (ref) | | | | | | | | | | | <0.001 | | | | | 259,126 | | | 13,536 | | | | | | | | 4.58 | | | | | | | | | | (ref) | | | | | | | | <0.001 | | | | | <0.001 | | | | |  |
| 100-149 | | | 561,527 | | | | | | | | 4,075 | | | 0.57 | | | | 1.09  (1.04–1.14) | | | | <0.001 | | | | | | |  |  |  |  |  | 239,848 | | | 13,624 | | | | | | | | 4.94 | | | | | | | | | | 1.08  (1.05–1.10) | | | <0.001 | | | | |  |  |  |  |  |  | | | | |  |
| 150-199 | | | 294,398 | | | | | | | | 2,447 | | | 0.66 | | | | 1.14  (1.08–1.20) | | | | <0.001 | | | | | | |  |  |  |  |  | 123,806 | | | 7,058 | | | | | | | | 4.92 | | | | | | | | | | 1.08  (1.05–1.12) | | | <0.001 | | | | |  |  |  |  |  |  | | | | |  |
| ≥200 | | | 310,826 | | | | | | | | 3,145 | | | 0.80 | | | | 1.24  (1.18–1.30) | | | | <0.001 | | | | | | |  |  |  |  |  | 112,522 | | | 6,601 | | | | | | | | 5.06 | | | | | | | | | | 1.13  (1.10–1.17) | | | <0.001 | | | | |  |  |  |  |  |  | | | | |  |
| **Abdominal obesity** | | | | | | | | | | | | | | | | | | | | | | | | | | | | | | | | | | | | | | | | | | | | | | | | | | | | | | | | | | | | | | | | | | | | | | | | | |
| Yes | | | 434,187 | | | | | | | | | 3,880 | | | 0.71 | | | 1.03  (0.99–1.07) | | | | 0.199 | | | | | | | - | | | | | 223,739 | | | 12,191 | | | | | | | | 4.66 | | | | | | | | | | 0.93  (0.91–0.95) | | | <0.001 | | | | | | - | | | | | <0.001 | | |  |  |
| **Regular physical activity** | | | | | | | | | | | | | | | | | | | | | | | | | | | | | | | | | | | | | | | | | | | | | | | | | | | | | | | | | | | | | | | | | | | | | | | | | |
| Yes | | | 412,891 | | | | | | | | | 2,833 | | | 0.54 | | | 1.14  (1.09–1.19) | | | | <0.001 | | | | | | | - | | | | | 149,503 | | | 6,534 | | | | | | | | 3.72 | | | | | | | | | | 1.23  (1.20–1.26) | | | <0.001 | | | | | - | | | | | <0.001 | | | |  |  |

1. **Men**

|  | **Total** | | | | | | | | | | | | | | | | | | **<40 years old** | | | | | | | | | | | | | | | | | | | | | | | | | | | | | | | | | | | | | | | | **40-49 years old** | | | | | | | | | | | | | | | | | | | | |
| --- | --- | --- | --- | --- | --- | --- | --- | --- | --- | --- | --- | --- | --- | --- | --- | --- | --- | --- | --- | --- | --- | --- | --- | --- | --- | --- | --- | --- | --- | --- | --- | --- | --- | --- | --- | --- | --- | --- | --- | --- | --- | --- | --- | --- | --- | --- | --- | --- | --- | --- | --- | --- | --- | --- | --- | --- | --- | --- | --- | --- | --- | --- | --- | --- | --- | --- | --- | --- | --- | --- | --- | --- | --- | --- | --- | --- | --- | --- | --- |
|  | **N** | **Events** | | | | **IR** | | | | **HR**  **(95% CI)** | ***p*** | | | | ***p*_trend_** | | | | **N** | | | | | | **Events** | | | | | | | | | | | **IR** | | | | | | **HR**  **(95% CI)** | | | | | | ***p*** | | | | | | | | | ***p*_trend_** | | **N** | | | | **Events** | | | | | | **IR** | | | | **HR**  **(95% CI)** | | ***p*** | | | | ***p*_trend_** |
| **BMI, kg/m2** | | | | | | | | | | | | | | | | | | | | | | | | | | | | | | | | | | | | | | | | | | | | | | | | | | | | | | | | | | | | | | | | | | | | | | | | | | | | | | | |
| <18.5 | 72,269 | 1,862 | | | | 2.19 | | | | 1.60  (1.52–1.68) | <0.001 | | | |  | | | | 28,778 | | | | | | 38 | | | | | | | | | | | 0.10 | | | | | | 1.49  (1.07–2.08) | | | | | | | 0.019 | | | | | | | |  | | 12,816 | | | | | | 85 | | | | 0.53 | | | | 1.70  (1.37–2.13) | | <0.001 | | | |  |
| 18.5-22.9 | 109,2707 | 14,154 | | | | 1.03 | | | | (ref) | | |  | | | | | | 421,828 | | | | | | 403 | | | | | | | | | | | 0.07 | | | | | | (ref) | | | | | | | | | | | | | | |  | | 258,213 | | | | | | 1,035 | | | | 0.31 | | | | (ref) | | | | | |  |
| 23.0-24.9 | 882,703 | 8,709 | | | | 0.78 | | | | 0.78  (0.76–0.80) | <0.001 | | | <0.001 | | | | | 290,363 | | | | | | 329 | | | | | | | | | | | 0.09 | | | | | | 1.02  (0.88–1.18) | | | | | | | 0.786 | | | | | | | | <0.001 | | 238,396 | | | | | | 799 | | | | 0.26 | | | | 0.78  (0.71–0.86) | | 0.009 | | | | <0.001 |
| 25.0-29.9 | 109,5910 | 9,792 | | | | 0.70 | | | | 0.76  (0.74–0.78) | <0.001 | | |  | | | | | 367,511 | | | | | | 611 | | | | | | | | | | | 0.13 | | | | | | 1.23  (1.08–1.41) | | | | | | | 0.002 | | | | | | | |  | | 314,407 | | | | | | 1,169 | | | | 0.29 | | | | 0.78  (0.72–0.86) | | <0.001 | | | |  |
| ≥30.0 | 118,206 | 1,002 | | | | 0.67 | | | | 1.10  (1.03–1.17) | <0.001 | | |  | | | | | 61,126 | | | | | | 196 | | | | | | | | | | | 0.25 | | | | | | 1.89  (1.57–2.27) | | | | | | | <0.001 | | | | | | | |  | | 30,488 | | | | | | 207 | | | | 0.53 | | | | 1.20  (1.03–1.40) | | 0.021 | | | |  |
| **Smoking** | | | | | | | | | | | | | | | | | | | | | | | | | | | | | | | | | | | | | | | | | | | | | | | | | | | | | | | | | | | | | | | | | | | | | | | | | | | | | | | |
| Non | 1,001,024 | 12,117 | | | | 0.96 | | | | (ref) | | |  | | | | | | 348,753 | | | | | | 308 | | | | | | | | | | | 0.07 | | | | | | (ref) | | | | | | | | | | | | |  | | | | 224,545 | | | | | | 625 | | | | 0.22 | | | | (ref) | | | | | |  |
| Ex | 808,659 | 8,581 | | | | 0.84 | | | | 0.93  (0.90–0.95) | <0.001 | | | <0.001 | | | | | 184,671 | | | | | | 198 | | | | | | | | | | | 0.08 | | | | | | 1.03  (0.86–1.23) | | | | | | | 0.772 | | | | | | | | <0.001 | | 228,519 | | | | | | 558 | | | | 0.19 | | | | 0.89  (0.79–1.00) | | 0.051 | | | | <0.001 |
| Current | 1,452,112 | 14,821 | | | | 0.81 | | | | 1.73  (1.68–1.77) | <0.001 | | |  | | | | | 636,182 | | | | | | 1,071 | | | | | | | | | | | 0.13 | | | | | | 1.77  (1.55–2.01) | | | | | | | <0.001 | | | | | | | |  | | 401,256 | | | | | | 2,112 | | | | 0.41 | | | | 1.97  (1.80–2.17) | | <0.001 | | | |  |
| **Hypertension** | | | | | | | | | | | | | | | | | | | | | | | | | | | | | | | | | | | | | | | | | | | | | | | | | | | | | | | | | | | | | | | | | | | | | | | | | | | | | | | |
| Yes | 780,990 | 18,041 | | | | 1.88 | | | | 1.47  (1.44–1.51) | <0.001 | | | | - | | | | 127,945 | | | | | | 449 | | | | | | | | | | 0.27 | | | | | | | 2.40  (2.14–2.70) | | | | | | | <0.001 | | | | | | | | - | | 178,473 | | | | | | 1192 | | | | 0.52 | | | | 1.94  (1.80–2.10) | | <0.001 | | | | - |
| **Diabetes** | | | | | | | | | | | | | | | | | | | | | | | | | | | | | | | | | | | | | | | | | | | | | | | | | | | | | | | | | | | | | | | | | | | | | | | | | | | | | | | |
| Normo-glycemia | 2,110,224 | 17,924 | | | | 0.67 | | | | (ref) | | | | |  | | | | 910,342 | | | | | | | 1,104 | | | | | | | | 0.09 | | | | | | | (ref) | | | | | | | | | | | | | | | |  | | 537,980 | | | | | | 1,755 | | | | | 0.25 | | | (ref) | | | | | |  |
| Prediabetes | 844,976 | 9,672 | | | | 0.91 | | | | 1.02  (0.99–1.04) | 0.211 | | | | <0.001 | | | | 226,609 | | | | | | 355 | | | | | | | | | | 0.12 | | | | | 1.01  (0.89–1.14) | | | | | | | | | 0.901 | | | | | | | | <0.001 | | 242,535 | | | | | | 942 | | | | | 0.30 | | | 1.11  (1.02–1.20) | | 0.011 | | | | <0.001 |
| Diabetes | 306,595 | 7,923 | | | | 2.14 | | | | 1.51  (1.47–1.55) | <0.001 | | | |  | | | | 32,655 | | | | | | 118 | | | | | | | | | | 0.28 | | | | | 1.58  (1.30–1.93) | | | | | | | | | <0.001 | | | | | | | |  | | 73,805 | | | | | | 598 | | | | | 0.64 | | | 1.88  (1.71–2.07) | | <0.001 | | | |  |
| **Systolic BP, mmHg** | | | | | | | | | | | | | | | | | | | | | | | | | | | | | | | | | | | | | | | | | | | | | | | | | | | | | | | | | | | | | | | | | | | | | | | | | | | | | | | |
| <100 | 45,444 | 409 | | | | 0.72 | | | | (ref) | | | | |  | | | | 13,637 | | | | | | | | 7 | | | | | | 0.04 | | | | | | | (ref) | | | | | | | | | | | | | | | | |  | | 13,963 | | | | | | 38 | | | | | 0.21 | | | (ref) | | | | | |  |
| 100-119 | 1,030,001 | 7,052 | | | | 0.54 | | | | 0.97  (0.88–1.08) | 0.609 | | | |  | | | | 419,475 | | | | | | | | 336 | | | | | | 0.06 | | | | | | | 1.52  (0.72–3.22) | | | | | | | | | 0.272 | | | | | | | |  | | 286,055 | | | | | | 747 | | | | | 0.20 | | | 1.05  (0.76–1.46) | | 0.760 | | | |  |
| 120-139 | 1,774,926 | 18,020 | | | | 0.80 | | | | 1.20  (1.09–1.33) | <0.001 | | | | <0.001 | | | | 654,740 | | | | | | | | 910 | | | | | | 0.11 | | | | | | | 2.40  (1.14–5.06) | | | | | | | | | 0.021 | | | | | | | | <0.001 | | 460,502 | | | | | | 1,746 | | | | | 0.30 | | | 1.54  (1.12–2.13) | | 0.009 | | | | <0.001 |
| 140-159 | 337,114 | 7,158 | | | | 1.72 | | | | 1.54  (1.39–1.70) | <0.001 | | | |  | | | | 70,354 | | | | | | | | 218 | | | | | | 0.24 | | | | | | | 4.36  (2.05–9.29) | | | | | | | | | <0.001 | | | | | | | |  | | 78,310 | | | | | | 546 | | | | | 0.55 | | | 2.71  (1.94–3.78) | | <0.001 | | | |  |
| ≥160 | 74,310 | 2,880 | | | | 3.24 | | | | 2.25  (2.03–2.50) | <0.001 | | | |  | | | | 11,400 | | | | | | | | 106 | | | | | | 0.73 | | | | | | 11.51  (5.33–24.86) | | | | | | | | | | <0.001 | | | | | | | |  | | 15,490 | | | | | | 218 | | | | | 1.12 | | | 5.15  (3.64–7.29) | | <0.001 | | | |  |
| **Diastolic BP, mmHg** | | | | | | | | | | | | | | | | | | | | | | | | | | | | | | | | | | | | | | | | | | | | | | | | | | | | | | | | | | | | | | | | | | | | | | | | | | | | | | | |
| <60 | 38,038 | 481 | | | 1.02 | | | | (ref) | | | | | | |  | | | | 13,826 | | | | | | | | 11 | | | | | | | 0.06 | | | | | | | (ref) | | | | | | | | | | | | | |  | | 9,772 | | | | | | 29 | | | | | | 0.23 | | | (ref) | | | | | |  |
| 60-69 | 390,903 | 3,567 | | | 0.72 | | | | 0.87  (0.79–0.96) | | 0.004 | | | | |  | | | | 152,428 | | | | | | | | 111 | | | | | | | 0.06 | | | | | | | 0.88  (0.47–1.63) | | | | | | 0.680 | | | | | | | |  | | 10,1778 | | | | | | 234 | | | | | | 0.18 | | | 0.79  (0.54–1.17) | | 0.238 | | | |  |
| 70-79 | 1,148,566 | 10,151 | | | 0.70 | | | | 0.92  (0.84–1.01) | | 0.068 | | | | | <0.001 | | | | 455,400 | | | | | | | | 419 | | | | | | | 0.07 | | | | | | | 1.04  (0.57–1.90) | | | | | | 0.891 | | | | | | | | <0.001 | | 292,708 | | | | | | 860 | | | | | | 0.23 | | | 1.02  (0.70–1.48) | 0.925 | | | <0.001 | | |
| 80-89 | 1,291,556 | 14,100 | 0.86 | | | | 1.10  (1.00–1.20) | | | | 0.046 | |  | | | | 458,424 | | | | 702 | | | | | | | | 0.12 | | | | | | | | 1.57  (0.86–2.84) | | | | | | 0.141 | | | | | | |  | | | | | | | | 340,411 | | | | 1,363 | | | | 0.31 | | | | | 1.37  (0.95–1.98) | | | 0.096 | | |  | | |
| ≥90 | 392,732 | 7220 | 1.48 | | | | 1.47  (1.34–1.61) | | | | <0.001 | |  | | | | 89,528 | | | | 334 | | | | | | | | 0.29 | | | | | | | | 3.08  (1.68–5.63) | | | | | | <0.001 | | | | | | |  | | | | | | | | 109,651 | | | | 809 | | | | 0.58 | | | | | 2.44  (1.63–3.54) | | | <0.001 | | |  | | |
| **Non-HDL-c, mg/dL** | | | | | | | | | | | | | | | | | | | | | | | | | | | | | | | | | | | | | | | | | | | | | | | | | | | | | | | | | | | | | | | | | | | | | | | | | | | | | | | |
| <130 | 1,238,057 | 13,857 | 0.89 | | | | | (ref) | | | |  | | | | | | 519,536 | | | | | | 514 | | | | | | | | 0.08 | | | | | | (ref) | | | | | | | | | | | | | | | |  | | | | 270,462 | | 1,048 | | | | | | | | | 0.30 | | | (ref) | | | | | |  | |
| 130-159 | 1,048,325 | 10,654 | 0.80 | | | | | 0.95  (0.93–0.98) | | | <0.001 |  | | | | | | 359,018 | | | | | | 437 | | | | | | | | 0.09 | | | | | | 1.02  (0.89–1.16) | | | | | | | | | 0.806 | | | | | | |  | | | | 283,301 | | 902 | | | | | | | | | 0.25 | | | 0.84  (0.77–0.92) | | | <0.001 | | |  | |
| 160-189 | 651,023 | 6,881 | | 0.83 | | | | 1.03  (1.00–1.06) | | | 0.038 | <0.001 | | | | | | 198,705 | | | | | | 371 | | | | | | | | 0.14 | | | | | | 1.34  (1.16–1.54) | | | | | | | | | <0.001 | | | | | | | <0.001 | | | | 194,925 | | 739 | | | | | | | | | 0.29 | | | 0.97  (0.88–1.07) | | | 0.523 | | | <0.001 | |
| 190-219 | 240,973 | 2,888 | | 0.95 | | | | 1.22  (1.17–1.27) | | | <0.001 |  | | | | | | 69,114 | | | | | | 168 | | | | | | | | 0.19 | | | | | | 1.54  (1.28–1.85) | | | | | | | | | <0.001 | | | | | | |  | | | | 77,377 | | 384 | | | | | | | | | 0.39 | | | 1.21  (1.07–1.36) | | | 0.002 | | |  | |
| ≥220 | 83,417 | 1,239 | | 1.18 | | | | 1.56  (1.47–1.65) | | | <0.001 |  | | | | | | 23,233 | | | | | | 87 | | | | | | | | 0.29 | | | | | | 2.11  (1.66–2.67) | | | | | | | | | <0.001 | | | | | | |  | | | | 28,255 | | 222 | | | | | | | | | 0.61 | | | 1.80  (1.55–2.08) | | | <0.001 | | |  | |
| **LDL-c, mg/dL** | | | | | | | | | | | | | | | | | | | | | | | | | | | | | | | | | | | | | | | | | | | | | | | | | | | | | | | | | | | | | | | | | | | | | | | | | | | | | | | |
| <70 | 278,230 | 3,772 | | 1.09 | | | | (ref) | | | |  | | | | | | 106,183 | | | | | | 147 | | | | | | | 0.11 | | | | | | | (ref) | | | | | | | | | | | | | | | | <0.001 | | | | 63,863 | | | 391 | | | | | | | | 0.48 | | | (ref) | | | |  | | | |
| 70-99 | 882,812 | 9,380 | | 0.84 | | | | 0.89  (0.85–0.92) | | | <0.001 |  | | | | | | 366,243 | | | | | | 393 | | | | | | | 0.08 | | | | | | | 0.85  (0.70–1.02) | | | | | | | | 0.086 | | | | | | | |  |  |  |  | 205,636 | | | 742 | | | | | | | | 0.28 | | | 0.69  (0.61–0.77) | | <0.001 | |  | | | |
| 100-129 | 1,165,890 | 11,786 | | 0.80 | | | | 0.85  (0.82–0.89) | | | <0.001 | 0.013 | | | | | | 420,560 | | | | | | 530 | | | | | | | 0.10 | | | | | | | 0.91  (0.76–1.20) | | | | | | | | 0.320 | | | | | | | |  |  |  |  | 308,916 | | | 1,028 | | | | | | | | 0.26 | | | 0.66  (0.58–0.74) | | 0.887 | | 0.332 | | | |
| 130-159 | 672,370 | 7,094 | | 0.83 | | | | 0.88  (0.85–0.92) | | | <0.001 |  | | | | | | 206,711 | | | | | | 328 | | | | | | | 0.12 | | | | | | | 1.02  (0.84–1.24) | | | | | | | | 0.873 | | | | | | | |  |  |  |  | 194,553 | | | 699 | | | | | | | | 0.28 | | | 0.70  (0.62–0.80) | | <0.001 | |  | | | |
| ≥160 | 262,493 | 3,487 | | 1.05 | | | | 1.10  (1.05–1.15) | | | <0.001 |  | | | | | | 69,909 | | | | | | 179 | | | | | | | 0.20 | | | | | | | 1.47  (1.18–1.83) | | | | | | | | <0.001 | | | | | | | |  |  |  |  | 81,352 | | | 435 | | | | | | | | 0.42 | | | 1.02  (0.89–1.17) | | 0.801 | |  | | | |
| **Triglycerides, mg/dL** | | | | | | | | | | | | | | | | | | | | | | | | | | | | | | | | | | | | | | | | | | | | | | | | | | | | | | | | | | | | | | | | | | | | | | | | | | | | | | | |
| <100 | 1,128,507 | 11,627 | | 0.82 | | | | (ref) | | | | <0.001 | | | | | | 459,303 | | | | | | 416 | | | | | | | 0.07 | | | | | | | (ref) | | | | | | | | | | | | | | | <0.001 | | | | | 251,995 | | | 773 | | | | | | | 0.24 | | | | (ref) | | | | <0.001 | | | |
| 100-149 | 919,711 | 10,729 | | 0.93 | | | | 1.09  (1.06–1.12) | | | <0.001 |  |  |  |  |  |  | 314,417 | | | | | | 373 | | | | | | | 0.09 | | | | | | | 1.07  (0.93–1.23) | | | | | | | 0.365 | | | | | | | |  |  |  |  |  | 237,294 | | | 865 | | | | | | | 0.28 | | | | 1.12  (1.02–1.24) | | 0.022 | |  | | | |
| 150-199 | 535,691 | 5,970 | | 0.88 | | | | 1.12  (1.08–1.15) | | | <0.001 |  |  |  |  |  |  | 174,033 | | | | | | 284 | | | | | | | 0.13 | | | | | | | 1.27  (1.09–1.49) | | | | | | | 0.003 | | | | | | | |  |  |  |  |  | 152,028 | | | 584 | | | | | | | 0.30 | | | | 1.13  (1.01–1.26) | | 0.034 | |  | | | |
| ≥200 | 677,886 | 7,193 | | 0.84 | | | | 1.22  (1.18–1.26) | | | <0.001 |  |  |  |  |  |  | 221,853 | | | | | | 504 | | | | | | | 0.18 | | | | | | | 1.46  (1.27–1.69) | | | | | | | <0.001 | | | | | | | |  |  |  |  |  | 213,003 | | | 1,073 | | | | | | | 0.39 | | | | 1.35  (1.22–1.49) | | <0.001 | |  | | | |
| **Abdominal obesity** | | | | | | | | | | | | | | | | | | | | | | | | | | | | | | | | | | | | | | | | | | | | | | | | | | | | | | | | | | | | | | | | | | | | | | | | | | | | | | | |
| Yes | 691,155 | 9,254 | | 1.07 | | | | 0.97  (0.94–0.99) | | | 0.004 | - | | | | | | 202,871 | | | | | 458 | | | | | | | 0.17 | | | | | | | | 1.34  (1.19–1.50) | | | | | | <0.001 | | | | | | | | - | | | | | | 179,117 | | | 853 | | | | | | 0.37 | | | | | 1.09  (1.00–1.18) | | 0.040 | | | - | | |
| **Regular physical activity** | | | | | | | | | | | | | | | | | | | | | | | | | | | | | | | | | | | | | | | | | | | | | | | | | | | | | | | | | | | | | | | | | | | | | | | | | | | | | | | |
| Yes | 649,643 | 7,430 | | 0.91 | | | | 1.18  (1.15–1.21) | | | <0.001 | - | | | | | | 185,725 | | | | 246 | | | | | | | 0.10 | | | | | | | | | 0.98  (0.86–1.12) | | | | | 0.782 | | | | | | | | - | | | | | | | 163,302 | | | 586 | | | | | 0.28 | | | | | | 1.04  (0.94–1.14) | | 0.350 | | - | | | |

|  | | | | **50-64 years old** | | | | | | | | | | | | | | | | | | | | | | | | | | | | | | | **≥65 years old** | | | | | | | | | | | | | | | | | | | | | | | | | |  | | | |
| --- | --- | --- | --- | --- | --- | --- | --- | --- | --- | --- | --- | --- | --- | --- | --- | --- | --- | --- | --- | --- | --- | --- | --- | --- | --- | --- | --- | --- | --- | --- | --- | --- | --- | --- | --- | --- | --- | --- | --- | --- | --- | --- | --- | --- | --- | --- | --- | --- | --- | --- | --- | --- | --- | --- | --- | --- | --- | --- | --- | --- | --- | --- | --- | --- |
|  | | | | **N** | | **Events** | | | | | | | **IR** | | | | | | **HR**  **(95% CI)** | | | | | | | ***p*** | | ***p*_trend_** | | | | | | **N** | | | | | | **Events** | | | | | | | | **IR** | | | **HR**  **(95% CI)** | | | ***p*** | ***p*_trend_** | | | | | | ***p*_interaction_** | | | |
| **BMI, kg/m2** | | | | | | | | | | | | | | | | | | | | | | | | | | | | | | | | | | | | | | | | | | | | | | | | | | | | | | | | | | | | | | | | |
| <18.5 | | | | 15,173 | | 346 | | | | | | | 1.94 | | | | | | 1.87  (1.68–2.09) | | | | | | | <0.001 | | |  | | | | | 15,502 | | | | | | 1,393 | | | | | | | | | 10.19 | | | 1.48  (1.40–1.57) | | <0.001 | | | |  | | | | <0.001 | | |
| 18.5-22.9 | | | | 275,189 | | 3,399 | | | | | | | 0.99 | | | | | | (ref) | | | | | | | | | |  | | | | | 137,477 | | | | | | 9,317 | | | | | | | | | 6.37 | | | (ref) | | | | | |  | | | |  | | |
| 23.0-24.9 | | | | 261,460 | | 2,504 | | | | | | | 0.76 | | | | | | 0.76  (0.72–0.80) | | | | | | | <0.001 | | | <0.001 | | | | | 92,484 | | | | | | 5,077 | | | | | | | | | 4.86 | | | 0.80  (0.77–0.82) | | <0.001 | | | | <0.001 | | | |  | | |
| 25.0-29.9 | | | | 321,096 | | 3,127 | | | | | | | 0.77 | | | | | | 0.74  (0.70–0.77) | | | | | | | <0.001 | | |  | | | | | 92,896 | | | | | | 4,885 | | | | | | | | | 4.58 | | | 0.76  (0.73–0.78) | | <0.001 | | | |  | | | |  | | |
| ≥30.0 | | | | 21,551 | | 290 | | | | | | | 1.08 | | | | | | 0.92  (0.81–1.04) | | | | | | | 0.166 | | |  | | | | | 5,041 | | | | | | 309 | | | | | | | | | 5.42 | | | 0.88  (0.79–0.99) | | 0.030 | | | |  | | | |  | | |
| **Smoking** | | | | | | | | | | | | | | | | | | | | | | | | | | | | | | | | | | | | | | | | | | | | | | | | | | | | | | | | | | | | | | | | |
| Non | | | | 280,077 | 2,424 | | | | | | | | | | | 0.69 | | | (ref) | | | | | | | | | | <0.001 | | | | | | 147,649 | 8,760 | | | | | | | | 5.32 | | | | | | (ref) | | | | | | | | <0.001 | | | | <0.001 | | |
| Ex | | | | 288,378 | 2,129 | | | | | | | | | | | 0.59 | | | 0.90  (0.85–0.95) | | | <0.001 | | | | | | |  | | | | | | 107,091 | 5,696 | | | | | | | | 4.78 | | | | | | 0.98  (0.95–1.01) | | | | 0.192 | | | |  | | | |  | | |
| Current | | | | 326,014 | 5,113 | | | | | | | | | | | 1.27 | | | 2.02  (1.92–2.13) | | | <0.001 | | | | | | |  | | | | | | 88,660 | 6,525 | | | | | | | | 7.02 | | | | | | 1.57  (1.52–1.62) | | | | <0.001 | | | |  | | | |  | | |
| **Hypertension** | | | | | | | | | | | | | | | | | | | | | | | | | | | | | | | | | | | | | | | | | | | | | | | | | | | | | | | | | | | | | | | | |
| Yes | | | | 306,635 | | | 4,565 | | | | | | | | | 1.20 | | | 1.60  (1.54–1.67) | | | <0.001 | | | | | | | - | | | | | | 167,937 | | | | 11,835 | | | | 6.44 | | | | | | | | | | 1.34  (1.30–1.37) | <0.001 | | | | - | | | | <0.001 | | |
| **Diabetes** | | | | | | | | | | | | | | | | | | | | | | | | | | | | | | | | | | | | | | | | | | | | | | | | | | | | | | | | | | | | | | | | |
| Normoglycemia | | | | 481,175 | | | | 4,438 | | | | | | | | 0.73 | | (ref) | | | | | | | | | | | | | | <0.001 | | | 180,727 | | | | 10,627 | | | 5.31 | | | | | | | | | | (ref) | | | | | |  | | | | <0.001 | | |
| Prediabetes | | | | 276,911 | | | 2,696 | | | | | | | | | 0.78 | | 1.05  (1.00–1.10) | | | | | | 0.044 | | | | | | | |  | | | 98,921 | | | | 5,679 | | | 5.19 | | | | | | | | | | 1.01  (0.98–1.05) | | 0.434 | | | | <0.001 | | | |  | | |
| Diabetes | | | | 136,383 | | | 2,532 | | | | | | | | | 1.51 | | 1.80  (1.71–1.89) | | | | | | <0.001 | | | | | | | |  | | | 63,752 | | | 4,675 | | | | 6.96 | | | | | | | | | | 1.42  (1.38–1.48) | | <0.001 | | | |  | | | |  | | |
| **Systolic BP, mmHg** | | | | | | | | | | | | | | | | | | | | | | | | | | | | | | | | | | | | | | | | | | | | | | | | | | | | | | | | | | | | | | | | |
| <100 | | | | 13,762 | | | | | 122 | | | | | | | | 0.71 | | (ref) | | | | | | | | | | | | |  | | | 4,082 | | 242 | | | | 5.78 | | | | | | | | | | (ref) | | | | | | |  | | | | <0.001 | | |
| 100-119 | | | | 249,867 | | | | | 1,920 | | | | | | | | 0.61 | | 0.97  (0.81–1.17) | | | | | | 0.771 | | | | | | |  | | | 74,604 | | 4,049 | | | | 4.97 | | | | | | | | | | 0.92  (0.81–1.05) | | | 0.211 | | | |  | | | |  | | |
| 120-139 | | | | 480,542 | | | | | 5,004 | | | | | | | | 0.83 | | 1.34  (1.12–1.61) | | | | | | 0.002 | | | | | | | <0.001 | | | 179,142 | | 10,360 | | | | 5.23 | | | | | | | | | | 0.99  (0.87–1.13) | | | 0.915 | | | | <0.001 | | | |  | | |
| 140-159 | | | | 122,277 | | | | | 1,886 | | | | | | | | 1.24 | | 1.90  (1.58–2.28) | | | | | | <0.001 | | | | | | |  | | | 66,173 | | 4,508 | | | | 6.21 | | | | | | | | | | 1.16  (1.02–1.32) | | | 0.025 | | | |  | | | |  | | |
| ≥160 | | | | 28,021 | | | | | 734 | | | | | | | | 2.14 | | 3.09  (2.55–3.75) | | | | | | <0.001 | | | | | |  | | | | 19,399 | | 1,822 | | | | 8.90 | | | | | | | | | | 1.58  (1.38–1.80) | | | <0.001 | | | |  | | | |  | | |
| **Diastolic BP, mmHg** | | | | | | | | | | | | | | | | | | | | | | | | | | | | | | | | | | | | | | | | | | | | | | | | | | | | | | | | | | | | | | | | |
| <60 | 9,430 | | | | | | | | | 95 | | | | | | 0.81 | | | | | (ref) | | | | | | | | | | | |  | | 5,010 | | | | 346 | | | | | | 6.69 | | | | | (ref) | | | | | | | |  | | | | <0.001 | | |
| 60-69 | 94,511 | | | | | | | | | 788 | | | | | | 0.67 | | | | | 0.88  (0.71–1.08) | | | | | | 0.223 | | | | | |  | | 42,186 | | | | 2,434 | | | | | | 5.32 | | | | | 0.90  (0.80–1.00) | | | | 0.057 | | | |  | | | |  | | |
| 70-79 | | 287,757 | | | | | | | | | | 2,592 | | | 0.72 | | | | | | 0.99  (0.81–1.22) | | | | | | 0.952 | | | | | | <0.001 | | 112,701 | | | | 6,280 | | | | | | | | 5.07 | | | 0.91  (0.82–1.01) | | | | 0.088 | | | | <0.001 | | | |  | | |
| 80-89 | | 362,238 | | | | | | | | | | 4,036 | | | 0.89 | | | | | 1.27  (1.03–1.56) | | | | | | | 0.023 | | | | | |  | | 130,483 | | | | 7,999 | | | | | | | | 5.55 | | | 1.02  (0.92–1.14) | | | | 0.688 | | | |  | | | |  | | |
| ≥90 | | 140,533 | | | | | | | | | | 2,155 | | | 1.23 | | | | | 1.76  (1.43–2.16) | | | | | | | <0.001 | | | | | |  | | 53,020 | | | | 3,922 | | | | | | | | 6.77 | | | 1.26  (1.13–1.41) | | | | <0.001 | | | |  | | | |  | | |
| **Non-HDL-c, mg/dL** | | | | | | | | | | | | | | | | | | | | | | | | | | | | | | | | | | | | | | | | | | | | | | | | | | | | | | | | | | | | | | | | |
| <130 | | | 302,632 | | | | | | | | 3,410 | | | 0.91 | | | | (ref) | | | | | | | | | | | |  | | | | | 145,427 | | | 8,885 | | | | | | | | 5.70 | | | | (ref) | | | | | |  | | | <0.001 | | | | |  |
| 130-159 | | | 296,185 | | | | | | | | 2,858 | | | 0.77 | | | | 0.93  (0.88–0.98) | | | | | 0.003 | | | | | | |  | | | | | 6,457 | | | 5.28 | | | | | | | | 5.32 | | | | 0.99  (0.96–1.02) | | | | 0.512 | |  | | |  | | | | |  |
| 160-189 | | | 195,842 | | | | | | | | 1,998 | | | 0.81 | | | | 1.00  (0.95–1.06) | | | | | 0.987 | | | | | | | <0.001 | | | | | 3,773 | | | 5.46 | | | | | | | | 5.07 | | | | 1.05  (1.01–1.09) | | | | 0.016 | | <0.001 | | |  | | | | |  |
| 190-219 | | | 74,309 | | | | | | | | 951 | | | 1.02 | | | | 1.24  (1.15–1.33) | | | | | <0.001 | | | | | | |  | | | | | 1,385 | | | 6.15 | | | | | | | | 5.55 | | | | 1.18  (1.12–1.25) | | | | <0.001 | |  | | |  | | | | |  |
| ≥220 | | | 25,501 | | | | | | | | 449 | | | 1.41 | | | | 1.65  (1.50–1.83) | | | | | <0.001 | | | | | | |  | | | | | 481 | | | 6.86 | | | | | | | | 6.77 | | | | 1.32  (1.20–1.45) | | | | <0.001 | |  | | |  | | | | |  |
| **LDL-c, mg/dL** | | | | | | | | | | | | | | | | | | | | | | | | | | | | | | | | | | | | | | | | | | | | | | | | | | | | | | | | | | | | | | | | |
| <70 | | | 74,547 | | | | | | | | 1,080 | | | 1.18 | | | | (ref) | | | | | | | | | | | |  | | | | | 33,637 | | | 2,154 | | | | | | | | 6.14 | | | | (ref) | | | | | |  | | | <0.001 | | | | |  |
| 70-99 | | | 216,649 | | | | | | | | 2,442 | | | 0.90 | | | | 0.87  (0.81–0.94) | | | | | <0.001 | | | | | | |  | | | | | 94,284 | | | 5,803 | | | | | | | | 5.69 | | | | 0.93  (0.88–0.97) | | | | 0.002 | |  | | |  | | | | |  |
| 100-129 | | | 315,530 | | | | | | | | 3,068 | | | 0.77 | | | | 0.81  (0.76–0.87) | | | | | <0.001 | | | | | | | 0.224 | | | | | 120,884 | | | 7,160 | | | | | | | | 5.34 | | | | 0.91  (0.86–0.95) | | | | <0.001 | | 0.140 | | |  | | | | |  |
| 130-159 | | | 202,483 | | | | | | | | 1,963 | | | 0.77 | | | | 0.83  (0.77–0.90) | | | | | <0.001 | | | | | | |  | | | | | 68,623 | | | 4,104 | | | | | | | | 5.34 | | | | 0.93  (0.89–0.98) | | | | 0.010 | |  | | |  | | | | |  |
| ≥160 | | | 85,260 | | | | | | | | 1,113 | | | 1.04 | | | | 1.12  (1.03–1.22) | | | | | 0.008 | | | | | | |  | | | | | 25,972 | | | 1,760 | | | | | | | | 6.09 | | | | 1.07  (1.01–1.14) | | | | 0.036 | |  | | |  | | | | |  |
| **Triglycerides, mg/dL** | | | | | | | | | | | | | | | | | | | | | | | | | | | | | | | | | | | | | | | | | | | | | | | | | | | | | | | | | | | | | | | | |
| <100 | | | 283,754 | | | | | | | | 2,675 | | | 0.76 | | | | (ref) | | | | | | | | | | | | <0.001 | | | | | 133,455 | | | 7,763 | | | | | | | | 5.34 | | | | (ref) | | | | | | <0.001 | | | <0.001 | | | | |  |
| 100-149 | | | 261,520 | | | | | | | | 2,788 | | | 0.85 | | | | 1.10  (1.05–1.16) | | | | | <0.001 | | | | | | |  |  |  |  |  | 106,480 | | | 6,703 | | | | | | | | 5.74 | | | | 1.10  (1.06–1.13) | | | | <0.001 | |  |  |  |  | | | | |  |
| 150-199 | | | 156,514 | | | | | | | | 1,775 | | | 0.90 | | | | 1.16  (1.09–1.23) | | | | | <0.001 | | | | | | |  |  |  |  |  | 53,116 | | | 3,327 | | | | | | | | 5.65 | | | | 1.11  (1.06–1.16) | | | | <0.001 | |  |  |  |  | | | | |  |
| ≥200 | | | 192,681 | | | | | | | | 2,428 | | | 1.00 | | | | 1.24  (1.17–1.31) | | | | | <0.001 | | | | | | |  |  |  |  |  | 50,349 | | | 3,188 | | | | | | | | 5.69 | | | | 1.16  (1.11–1.21) | | | | <0.001 | |  |  |  |  | | | | |  |
| **Abdominal obesity** | | | | | | | | | | | | | | | | | | | | | | | | | | | | | | | | | | | | | | | | | | | | | | | | | | | | | | | | | | | | | | | | |
| Yes | | | 220,610 | | | | | | | | | 2,569 | | | 0.93 | | | 0.96  (0.91–1.00) | | | | | 0.065 | | | | | | | - | | | | | 88,557 | | | 5,374 | | | | | | | | 5.42 | | | | 0.93  (0.90–0.96) | | | | <0.001 | | | - | | | <0.001 | | |  |  |
| **Regular physical activity** | | | | | | | | | | | | | | | | | | | | | | | | | | | | | | | | | | | | | | | | | | | | | | | | | | | | | | | | | | | | | | | | |
| Yes | | | 210,912 | | | | | | | | | 2,096 | | | 0.79 | | | 1.11  (1.05–1.16) | | | | | <0.001 | | | | | | | - | | | | | 89,704 | | | 4,502 | | | | | | | | 4.41 | | | | 1.21  (1.17–1.25) | | | | <0.001 | | - | | | <0.001 | | | |  |  |

1. **Women**

|  | **Total** | | | | | | | | | | | | | | | | | | **<40 years old** | | | | | | | | | | | | | | | | | | | | | | | | | | | | | | | | **40-49 years old** | | | | | | | | | | | | | | | | | | | | | | | | | |
| --- | --- | --- | --- | --- | --- | --- | --- | --- | --- | --- | --- | --- | --- | --- | --- | --- | --- | --- | --- | --- | --- | --- | --- | --- | --- | --- | --- | --- | --- | --- | --- | --- | --- | --- | --- | --- | --- | --- | --- | --- | --- | --- | --- | --- | --- | --- | --- | --- | --- | --- | --- | --- | --- | --- | --- | --- | --- | --- | --- | --- | --- | --- | --- | --- | --- | --- | --- | --- | --- | --- | --- | --- | --- | --- | --- | --- |
|  | **N** | | **Events** | | | | **IR** | | **HR**  **(95% CI)** | ***p*** | | | | | | ***p*_trend_** | | | **N** | **Events** | | | | | | | | | | | | **IR** | | | | **HR**  **(95% CI)** | | | | | | | | ***p*** | | | | | ***p*_trend_** | | **N** | | | | **Events** | | | | **IR** | | | | | | **HR**  **(95% CI)** | | | | | ***p*** | | | | ***p*_trend_** | | |
| **BMI, kg/m2** | | | | | | | | | | | | | | | | | | | | | | | | | | | | | | | | | | | | | | | | | | | | | | | | | | | | | | | | | | | | | | | | | | | | | | | | | | | | |
| <18.5 | 166,545 | | 1,487 | | | | 0.71 | | 1.55  (1.47–1.64) | <0.001 | | | | | |  | | | 107,261 | 26 | | | | | | | | | | | | 0.02 | | | | 1.08  (0.71–1.66) | | | | | | | | 0.721 | | | |  | | | 29,043 | | | | 35 | | | | 0.09 | | | | | | 1.30  (0.92–1.83) | | | | | 0.140 | | | |  | | |
| 18.5-22.9 | | 1,399,376 | 9,580 | | | | 0.54 | | (ref) | | |  | | | | | | | 456,301 | 120 | | | | | | | | | | | | 0.02 | | | | (ref) | | | | | | | | | | | |  | | | 442,392 | | | | 432 | | | | 0.08 | | | | | | (ref) | | | | | | | | |  | | |
| 23.0-24.9 | | 648,266 | 5,821 | | | | 0.71 | | 0.82  (0.79–0.84) | <0.001 | | | | <0.001 | | | | | 85,658 | 50 | | | | | | | | | | | | 0.05 | | | | 1.82  (1.30–2.54) | | | | | | | | <0.001 | | | | <0.001 | | | 193,275 | | | | 203 | | | | 0.08 | | | | | | 0.95  (0.81–1.13) | | | | | 0.585 | | | | 0.441 | | |
| 25.0-29.9 | | 677,902 | 7,268 | | | | 0.84 | | 0.78  (0.76–0.81) | <0.001 | | | |  | | | | | 67,924 | 55 | | | | | | | | | | | | 0.06 | | | | 2.13  (1.53–2.98) | | | | | | | | <0.001 | | | |  | | | 172,950 | | | | 239 | | | | 0.11 | | | | | | 1.06  (0.90–1.25) | | | | | 0.489 | | | |  | | |
| ≥30.0 | | 95,968 | 1,119 | | | | 0.92 | | 0.93  (0.87–0.99) | 0.016 | | | |  | | | | | 15,653 | 18 | | | | | | | | | | | | 0.09 | | | | 2.20  (1.29–3.76) | | | | | | | | 0.004 | | | |  | | | 25,867 | | | | 58 | | | | 0.18 | | | | | | 1.27  (0.95–1.70) | | | | | 0.102 | | | |  | | |
| **Smoking** | | | | | | | | | | | | | | | | | | | | | | | | | | | | | | | | | | | | | | | | | | | | | | | | | | | | | | | | | | | | | | | | | | | | | | | | | | | | |
| Non | | 2,826,772 | 23,496 | | | | 0.65 | | (ref) | | | |  | | | | | | 671,770 | 215 | | | | | | | | | | | | 0.02 | | | | (ref) | | | | | | | | | | | |  | | | 816,789 | | | | 843 | | | | 0.08 | | | | | | (ref) | | | | | | | | |  | | |
| Ex | | 55,264 | 404 | | | | 0.57 | | 1.33  (1.20–1.46) | <0.001 | | | | <0.001 | | | | | 237,94 | 13 | | | | | | | | | | | | 0.04 | | | | 1.65  (0.94–2.91) | | | | | | | 0.081 | | | | | <0.001 | | | 15,479 | | | | 20 | | | | 0.10 | | | | | | 1.24  (0.80–1.94) | | | | | 0.339 | | | | <0.001 | | |
| Current | | 106,021 | 1,375 | | | | 1.03 | | 2.11  (2.00–2.23) | <0.001 | | | |  | | | | | 372,33 | 41 | | | | | | | | | | | | 0.09 | | | | 3.25  (2.28–4.64) | | | | | | | <0.001 | | | | |  | | | 31,259 | | | | 104 | | | | 0.26 | | | | | | 2.80  (2.25–3.49) | | | | | <0.001 | | | |  | | |
| **Hypertension** | | | | | | | | | | | | | | | | | | | | | | | | | | | | | | | | | | | | | | | | | | | | | | | | | | | | | | | | | | | | | | | | | | | | | | | | | | | | |
| Yes | | 614,190 | 15,182 | | | | 1.98 | | 1.48  (1.44–1.52) | <0.001 | | | | | - | | | | 16,487 | | | | | | 38 | | | | | | | 0.18 | | | | 3.86  (2.66–5.59) | | | | | | | <0.001 | | | | | - | | | 92,169 | | | | 278 | | | | 0.24 | | | | | | 2.86  (2.46–3.32) | | | | | <0.001 | | | | - | | |
| **Diabetes** | | | | | | | | | | | | | | | | | | | | | | | | | | | | | | | | | | | | | | | | | | | | | | | | | | | | | | | | | | | | | | | | | | | | | | | | | | | | |
| Normo-glycemia | | 2,237,640 | 13,863 | | | | 0.49 | | (ref) | | | | | |  | | | | 651,621 | | | | | | | 213 | | | | | | 0.03 | | | | (ref) | | | | | | | | | | | |  | | | 678,705 | | | | 696 | | | | 0.08 | | | | | | (ref) | | | | | | | | |  | | |
| Prediabetes | | 562,623 | 6,179 | | | | 0.87 | | 1.02  (0.99–1.06) | 0.126 | | | | | <0.001 | | | | 74,195 | | | | | | 45 | | | | | | | 0.05 | | | | 1.33  (0.96–1.84) | | | | | | | 0.092 | | | | | <0.001 | | | 157,013 | | | | 172 | | | | 0.09 | | | | | | 0.91  (0.77–1.08) | | | | | 0.263 | | | | <0.001 | | |
| Diabetes | | 187,794 | 5,233 | | | | 2.27 | | 1.49  (1.45–1.54) | <0.001 | | | | |  | | | | 6,981 | | | | | | 11 | | | | | | | 0.12 | | | | 2.11  (1.13–3.97) | | | | | | | 0.020 | | | | |  | | | 27,809 | | | | 99 | | | | 0.28 | | | | | | 2.27  (1.82–2.84) | | | | | <0.001 | | | |  | | |
| **Systolic BP, mmHg** | | | | | | | | | | | | | | | | | | | | | | | | | | | | | | | | | | | | | | | | | | | | | | | | | | | | | | | | | | | | | | | | | | | | | | | | | | | | |
| <100 | | 175,958 | 317 | | | | 0.14 | | (ref) | | | | | |  | | | | 74,455 | | | | | | | 8 | | | | | | 0.01 | | | | (ref) | | | | | | | | | | | |  | | | 60,047 | | | | 41 | | | | 0.05 | | | | | | (ref) | | | | | | | | |  | | |
| 100-119 | | 1,341,005 | 5,103 | | | | 0.30 | | 1.16  (1.04–1.30) | 0.010 | | | | |  | | | | 460,257 | | | | | | | 101 | | | | | | 0.02 | | | | 1.91  (0.93–3.92) | | | | | | | 0.080 | | | | |  | | | 435,333 | | | | 313 | | | | 0.06 | | | | | | 1.07  (0.77–1.48) | | | | | 0.695 | | | |  | | |
| 120-139 | | 1,184,601 | 12,376 | | | | 0.82 | | 1.38  (1.23–1.54) | <0.001 | | | | | <0.001 | | | | 189,626 | | | | | | | 137 | | | | | | 0.06 | | | | 5.21  (2.54–10.69) | | | | | | | <0.001 | | | | | <0.001 | | | 320,210 | | | | 460 | | | | 0.11 | | | | | | 2.04  (1.48–2.82) | | | | | <0.001 | | | | <0.001 | | |
| 140-159 | | 230,960 | 5,359 | | | | 1.86 | | 1.66  (1.48–1.86) | <0.001 | | | | |  | | | | 7,185 | | | | | | | 15 | | | | | | 0.16 | | | | 10.00  (4.25–24.10) | | | | | | | <0.001 | | | | |  | | | 39,805 | | | | 107 | | | | 0.21 | | | | | | 3.47  (2.40–5.02) | | | | | <0.001 | | | |  | | |
| ≥160 | | 55,533 | 2,120 | | | | 3.11 | | 2.08  (1.84–2.34) | <0.001 | | | | |  | | | | 1,274 | | | | | | | 8 | | | | | | 0.49 | | | | 25.35  (9.26–69.36) | | | | | | | <0.001 | | | | |  | | | 8,132 | | | | 46 | | | | 0.44 | | | | | | 6.09  (4.54–10.75) | | | | | <0.001 | | | |  | | |
| **Diastolic BP, mmHg** | | | | | | | | | | | | | | | | | | | | | | | | | | | | | | | | | | | | | | | | | | | | | | | | | | | | | | | | | | | | | | | | | | | | | | | | | | | | |
| <60 | | 112,872 | 320 | | | 0.22 | | | (ref) | | | | | | | |  | | 43,083 | | | | | | | 4 | | | | | | 0.01 | | | | (ref) | | | | | | | | | | | |  | | | 9,772 | | | | | 20 | | | | | | | 0.04 | | | | | | (ref) | | | | | |  | |
| 60-69 | | 716,136 | 2,828 | | | 0.31 | | | 1.09  (0.97–1.23) | 0.137 | | | | | | |  | | 248,118 | | | | | | | 53 | | | | | | 0.02 | | | | 2.21  (0.80–6.11) | | | | | | | 0.125 | | | | |  | | | 10,1778 | | | | | 157 | | | | | | | 0.05 | | | | | | 1.32  (0.83–2.10) | | | | 0.248 | |  | |
| 70-79 | | 1,119,967 | 7,614 | | | 0.53 | | | 1.25  (1.11–1.39) | <0.001 | | | | | | | <0.001 | | 300,421 | | | | | | | 83 | | | | | | 0.02 | | | | 2.63  (0.97–7.19) | | | | | | | 0.059 | | | | | <0.001 | | | 292,708 | | | | | 302 | | | | | | | 0.07 | | | | | | 1.76  (1.12–2.77) | | | | 0.015 | | <0.001 | |
| 80-89 | | 811,839 | 9,657 | 0.94 | | | | | 1.46  (1.31–1.64) | <0.001 | | | | | | |  | | 129,439 | | | | | | | 100 | | | | | | 0.06 | | | | 6.28  (2.30–17.10) | | | | | | | <0.001 | | | | |  | | | 340,411 | | | | | 328 | | | | | | | | 0.12 | | | | | 2.80  (1.78–4.40) | | | | <0.001 | |  | |
| ≥90 | | 227,243 | 4,856 | 1.71 | | | | | 1.83  (1.63–2.05) | <0.001 | | | | | | |  | | 11,736 | | | | | | | 29 | | | | | | 0.19 | | | | 14.00  (4.86–40.35) | | | | | | | <0.001 | | | | |  | | | 109,651 | | | | | 160 | | | | | | | | 0.25 | | | | | 5.37  (3.35–8.59) | | | | <0.001 | |  | |
| **Non-HDL-c, mg/dL** | | | | | | | | | | | | | | | | | | | | | | | | | | | | | | | | | | | | | | | | | | | | | | | | | | | | | | | | | | | | | | | | | | | | | | | | | | | | |
| <130 | 1,351,248 | | 7,587 | 0.44 | | | | (ref) | | | | |  | | | | | | 522,449 | | | | | 167 | | | | | | | 0.02 | | | | (ref) | | | | | | | | | | | |  | | | | 430,262 | 480 | | | | | | 0.09 | | | | | | | | | | (ref) | | | | | | | |  |
| 130-159 | 864,776 | | 7,561 | 0.69 | | | | 0.91  (0.89–0.94) | | <0.001 | | |  | | | | | | 151,944 | | | | | 65 | | | | | | | 0.03 | | | | 1.00  (0.74–1.34) | | | | | | | 0.976 | | | | |  | | | | 266,540 | 272 | | | | | | 0.08 | | | | | | | | | | 0.85  (0.73–0.99) | | | | 0.033 | | | |  |
| 160-189 | 499,740 | | 5,983 | | 0.94 | | | 0.96  (0.93–1.00) | | 0.025 | | | 0.012 | | | | | | 44,082 | | | | 24 | | | | | | | 0.04 | | | | 0.99  (0.64–1.54) | | | | | | | 0.967 | | | | 0.435 | | | | | | 119,919 | | 128 | | | | 0.08 | | | | | | | | | 0.81  (0.66–0.98) | | | | | 0.035 | | | | 0.971 | |
| 190-219 | 195,798 | | 2,807 | | 1.13 | | | 1.00  (0.96–1.04) | | 0.997 | | |  | | | | | | 10,652 | | | | 7 | | | | | | | 0.05 | | | | 0.99  (0.46–2.15) | | | | | | | 0.983 | | | |  | | | | | | 35,573 | | 58 | | | | 0.13 | | | | | | | | | 1.10  (0.84–1.46) | | | | | 0.484 | | | |  | |
| ≥220 | 76,495 | | 1,337 | | 1.39 | | | 1.13  (1.06–1.20) | | <0.001 | | |  | | | | | | 3,670 | | | | 6 | | | | | | | 0.13 | | | | 2.35  (1.01–5.43) | | | | | | | 0.047 | | | |  | | | | | | 11,233 | | 29 | | | | 0.20 | | | | | | | | | 1.52  (1.04–2.23) | | | | | 0.031 | | | |  | |
| **LDL-c, mg/dL** | | | | | | | | | | | | | | | | | | | | | | | | | | | | | | | | | | | | | | | | | | | | | | | | | | | | | | | | | | | | | | | | | | | | | | | | | | | | |
| <70 | 197,068 | | 1,527 | | 0.61 | | | (ref) | | | | |  | | | | | 84,138 | | | | 44 | | | | | | | 0.04 | | | | (ref) | | | | | | | | | | | | | | 0.030 | | | 52,888 | | | | 84 | | | | | | | | 0.12 | | | | | (ref) | | | | | | |  | | |
| 70-99 | 820,432 | | 5,248 | | 0.50 | | | 0.91  (0.86–0.96) | | <0.001 | | |  | | | | | 308,718 | | | | 102 | | | | | | | 0.03 | | | | 0.63  (0.44–0.90) | | | | | | | 0.010 | | | | | | |  |  |  | 252,255 | | | | 290 | | | | | | | | 0.09 | | | | | 0.82  (0.64–1.05) | | | 0.115 | | | |  | | |
| 100-129 | 1,046,503 | | 8,164 | | 0.61 | | | 0.86  (0.82–0.91) | | 0.006 | | | 0.091 | | | | | 245,916 | | | | 79 | | | | | | | 0.02 | | | | 0.52  (0.36–0.75) | | | | | | | <0.001 | | | | | | |  |  |  | 333,502 | | | | 336 | | | | | | | | 0.08 | | | | | 0.71  (0.56–0.91) | | | 0.006 | | | | 0.043 | | |
| 130-159 | 622,225 | | 6,392 | | 0.81 | | | 0.87  (0.82–0.92) | | 0.585 | | |  | | | | | 75,250 | | | | 30 | | | | | | | 0.03 | | | | 0.51  (0.32–0.82) | | | | | | | 0.006 | | | | | | |  |  |  | 166,520 | | | | 178 | | | | | | | | 0.08 | | | | | 0.71  (0.54–0.92) | | | 0.009 | | | |  | | |
| ≥160 | 301,829 | | 3,944 | | 1.03 | | | 0.93  (0.87–0.98) | | 0.011 | | |  | | | | | 18,775 | | | | 14 | | | | | | | 0.06 | | | | 0.79  (0.42–1.46) | | | | | | | 0.443 | | | | | | |  |  |  | 58,362 | | | | 79 | | | | | | | | 0.11 | | | | | 0.81  (0.59–1.10) | | | 0.182 | | | |  | | |
| **Triglycerides, mg/dL** | | | | | | | | | | | | | | | | | | | | | | | | | | | | | | | | | | | | | | | | | | | | | | | | | | | | | | | | | | | | | | | | | | | | | | | | | | | | |
| <100 | 1,676,753 | | 7,950 | | 0.37 | | | (ref) | | | <0.001 | | | | | | | 566,268 | | | | 161 | | | | | | | 0.02 | | | | (ref) | | | | | | | | | | | | | 0.007 | | | | 541,019 | | | | 491 | | | | | | | 0.07 | | | | | | (ref) | | | | | | | <0.001 | | |
| 100-149 | 749,814 | | 8,535 | | 0.90 | | | 1.06  (1.03–1.10) | | <0.001 |  |  |  |  |  |  |  | 114,564 | | | | 64 | | | | | | | 0.04 | | | | 1.39  (1.03–1.88) | | | | | | 0.031 | | | | | | |  |  |  |  | 201,875 | | | | 263 | | | | | | | 0.10 | | | | | | 1.24  (1.06–1.44) | | | 0.007 | | | |  |  |  |
| 150-199 | 309,293 | | 4,526 | | 1.16 | | | 1.06  (1.02–1.10) | | 0.002 |  |  |  |  |  |  |  | 31,110 | | | | 22 | | | | | | | 0.05 | | | | 1.41  (0.89–2.24) | | | | | | 0.147 | | | | | | |  |  |  |  | 69,609 | | | | 101 | | | | | | | 0.11 | | | | | | 1.20  (0.96–1.50) | | | 0.103 | | | |  |  |  |
| ≥200 | 252,197 | | 4,264 | | 1.34 | | | 1.13  (1.09–1.17) | | <0.001 |  |  |  |  |  |  |  | 20,855 | | | | 22 | | | | | | | 0.08 | | | | 1.72  (1.07–2.78) | | | | | | 0.027 | | | | | | |  |  |  |  | 51,024 | | | | 112 | | | | | | | 0.17 | | | | | | 1.55  (1.24–1.93) | | | <0.001 | | | |  |  |  |
| **Abdominal obesity** | | | | | | | | | | | | | | | | | | | | | | | | | | | | | | | | | | | | | | | | | | | | | | | | | | | | | | | | | | | | | | | | | | | | | | | | | | | | |
| Yes | 485,130 | | 8,330 | | 1.36 | | | 0.96  (0.93–0.98) | | 0.002 | - | | | | | | | 43,127 | | | 44 | | | | | | | 0.08 | | | | | 1.82  (1.28–2.59) | | | | | <0.001 | | | | | | | - | | | | | 93,244 | | | | 158 | | | | | | 0.13 | | | | | | | 1.10  (0.92–1.31) | | | 0.322 | | | | | - | |
| **Regular physical activity** | | | | | | | | | | | | | | | | | | | | | | | | | | | | | | | | | | | | | | | | | | | | | | | | | | | | | | | | | | | | | | | | | | | | | | | | | | | | |
| Yes | 471,637 | | 2,934 | | 0.49 | | | 1.27  (1.23–1.32) | | <0.001 | - | | | | | | | 68,411 | | 31 | | | | | | | 0.04 | | | | | | 0.90  (0.62–1.31) | | | | 0.572 | | | | | | | | - | | | | | 141,448 | | | | 134 | | | | | 0.07 | | | | | | | | 1.23  (1.03–1.48) | | | 0.026 | | | | - | | |

|  | | | | **50-64 years old** | | | | | | | | | | | | | | | | | | | | | | | | | | | | | | **≥65 years old** | | | | | | | | | | | | | | | | | | | | | | | | | |  | | |
| --- | --- | --- | --- | --- | --- | --- | --- | --- | --- | --- | --- | --- | --- | --- | --- | --- | --- | --- | --- | --- | --- | --- | --- | --- | --- | --- | --- | --- | --- | --- | --- | --- | --- | --- | --- | --- | --- | --- | --- | --- | --- | --- | --- | --- | --- | --- | --- | --- | --- | --- | --- | --- | --- | --- | --- | --- | --- | --- | --- | --- | --- | --- |
|  | | | | **N** | | **Events** | | | | | | | **IR** | | | | | | **HR**  **(95% CI)** | | | | | | | ***p*** | ***p*_trend_** | | | | | | **N** | | | | | | **Events** | | | | | | | | **IR** | | | **HR**  **(95% CI)** | | | ***p*** | ***p*_trend_** | | | | | | ***p*_interaction_** | | |
| **BMI, kg/m2** | | | | | | | | | | | | | | | | | | | | | | | | | | | | | | | | | | | | | | | | | | | | | | | | | | | | | | | | | | | | | | |
| <18.5 | | | | 16,895 | | 116 | | | | | | | 0.54 | | | | | | 2.00  (1.65–2.41) | | | | | | | <0.001 | |  | | | | | 13,346 | | | | | | 1,310 | | | | | | | | | 9.46 | | | 1.46  (1.38–1.55) | | <0.001 | | | |  | <0.001 | | | | |
| 18.5-22.9 | | | | 371,542 | | 1,328 | | | | | | | 0.28 | | | | | | (ref) | | | | | | | | |  | | | | | 129,141 | | | | | | 7,700 | | | | | | | | | 5.08 | | | (ref) | |  | | | |  |  | | | | |
| 23.0-24.9 | | | | 269,396 | | 1,079 | | | | | | | 0.31 | | | | | | 0.96  (0.89–1.04) | | | | | | | 0.320 | | <0.001 | | | | | 99,937 | | | | | | 4,489 | | | | | | | | | 3.71 | | | 0.79  (0.77–0.82) | | <0.001 | | | | <0.001 |  | | | | |
| 25.0-29.9 | | | | 303,745 | | 1,419 | | | | | | | 0.36 | | | | | | 0.95  (0.88–1.03) | | | | | | | 0.207 | |  | | | | | 133,283 | | | | | | 5,555 | | | | | | | | | 3.42 | | | 0.75  (0.73–0.78) | | <0.001 | | | |  |  | | | | |
| ≥30.0 | | | | 38,253 | | 259 | | | | | | | 0.53 | | | | | | 1.16  (1.01–1.33) | | | | | | | 0.033 | |  | | | | | 16,195 | | | | | | 784 | | | | | | | | | 4.00 | | | 0.85  (0.79–0.92) | | <0.001 | | | |  |  | | | | |
| **Smoking** | | | | | | | | | | | | | | | | | | | | | | | | | | | | | | | | | | | | | | | | | | | | | | | | | | | | | | | | | | | | | | |
| Non | | | | 960,054 | 3,860 | | | | | | | | | | | 0.31 | | | (ref) | | | | | | | | |  | | | | | | 378,159 | 18,578 | | | | | | | | 4.10 | | | | | | (ref) | | | | | | | |  | 0.005 | | | | |
| Ex | | | | 11,883 | 65 | | | | | | | | | | | 0.43 | | | 1.50  (1.17–1.92) | | 0.001 | | | | | | | <0.001 | | | | | | 4,108 | 306 | | | | | | | | 6.56 | | | | | | 1.26  (1.12–1.41) | | | | <0.001 | | | | <0.001 |  | | | | |
| Current | | | | 27,894 | 276 | | | | | | | | | | | 0.78 | | | 2.70  (2.38–3.07) | | <0.001 | | | | | | |  | | | | | | 9,635 | 954 | | | | | | | | 9.06 | | | | | | 1.88  (1.76–2.00) | | | | <0.001 | | | |  |  | | | | |
| **Hypertension** | | | | | | | | | | | | | | | | | | | | | | | | | | | | | | | | | | | | | | | | | | | | | | | | | | | | | | | | | | | | | | |
| Yes | | | | 293,681 | | | 2,086 | | | | | | | | | 0.56 | | | 1.84  (1.73–1.97) | | <0.001 | | | | | | | - | | | | | | 211,853 | | | | 12,780 | | | | 5.09 | | | | | | | | | | 1.37  (1.33–1.41) | <0.001 | | | | - | <0.001 | | | | |
| **Diabetes** | | | | | | | | | | | | | | | | | | | | | | | | | | | | | | | | | | | | | | | | | | | | | | | | | | | | | | | | | | | | | | |
| Normoglycemia | | | | 680,305 | | | | 2,433 | | | | | | | | 0.28 | | (ref) | | | | | | | | | | | | |  | | | 227,009 | | | | 10,521 | | | 3.84 | | | | | | | | | | (ref) | | | | | |  | <0.001 | | | | |
| Prediabetes | | | | 232,129 | | | 963 | | | | | | | | | 0.32 | | 1.01  (0.94–1.09) | | | | | 0.837 | | | | | | | | <0.001 | | | 99,286 | | | | 4,999 | | | 4.21 | | | | | | | | | | 1.03  (1.00–1.07) | | 0.067 | | | | <0.001 |  | | | | |
| Diabetes | | | | 87,397 | | | 805 | | | | | | | | | 0.73 | | 1.78  (1.64–1.94) | | | | | <0.001 | | | | | | | |  | | | 65,607 | | | 4,318 | | | | 5.70 | | | | | | | | | | 1.45  (1.40–1.50) | | <0.001 | | | |  |  | | | | |
| **Systolic BP, mmHg** | | | | | | | | | | | | | | | | | | | | | | | | | | | | | | | | | | | | | | | | | | | | | | | | | | | | | | | | | | | | | | |
| <100 | | | | 36,711 | | | | | 82 | | | | | | | | 0.17 | | (ref) | | | | | | | | | | | |  | | | 4,745 | | 186 | | | | 3.30 | | | | | | | | | | (ref) | | | | | | |  | <0.001 | | | | |
| 100-119 | | | | 360,007 | | | | | 1,065 | | | | | | | | 0.23 | | 1.26  (1.00–1.58) | | | | | 0.046 | | | | | | |  | | | 85,408 | | 3,624 | | | | 3.53 | | | | | | | | | | 1.02  (0.88–1.18) | | | 0.785 | | | |  |  | | | | |
| 120-139 | | | | 472,610 | | | | | 2,034 | | | | | | | | 0.34 | | 1.62  (1.30–2.03) | | | | | <0.001 | | | | | | | <0.001 | | | 202,155 | | 9,745 | | | | 4.02 | | | | | | | | | | 1.12  (0.97–1.30) | | | 0.127 | | | | <0.001 |  | | | | |
| 140-159 | | | | 107,665 | | | | | 752 | | | | | | | | 0.55 | | 2.33  (1.85–2.94) | | | | | <0.001 | | | | | | |  | | | 76,305 | | 4,485 | | | | 4.94 | | | | | | | | | | 1.30  (1.12–1.51) | | | <0.001 | | | |  |  | | | | |
| ≥160 | | | | 22,838 | | | | | 268 | | | | | | | | 0.92 | | 3.71  (2.89–4.76) | | | | | <0.001 | | | | | |  | | | | 23,289 | | 1,798 | | | | 6.64 | | | | | | | | | | 1.56  (1.34–1.82) | | | <0.001 | | | |  |  | | | | |
| **Diastolic BP, mmHg** | | | | | | | | | | | | | | | | | | | | | | | | | | | | | | | | | | | | | | | | | | | | | | | | | | | | | | | | | | | | | | |
| <60 | 24,975 | | | | | | | | | 69 | | | | | | 0.22 | | | (ref) | | | | | | | | | | | | |  | | 5,642 | | | | 227 | | | | | | 3.40 | | | | | (ref) | | | | | | | |  | <0.001 | | | | |
| 60-69 | 183,820 | | | | | | | | | 529 | | | | | | 0.22 | | | 1.02  (0.79–1.31) | | | | | | 0.905 | | | | | | |  | | 50,678 | | | | 2,089 | | | | | | 3.44 | | | | | 1.06  (0.93–1.22) | | | | 0.400 | | | |  |  | | | | |
| 70-79 | | 361,493 | | | | | | | | | | 1,248 | | | 0.27 | | | | | 1.15  (0.90–1.46) | | | | | 0.271 | | | | | | | <0.001 | | 130,281 | | | | 5,981 | | | | | | | | 3.82 | | | 1.19  (1.04–1.35) | | | | 0.012 | | | | <0.001 |  | | | | |
| 80-89 | | 323,773 | | | | | | | | | | 1,567 | | | 0.38 | | | | | 1.53  (1.20–1.94) | | | | | <0.001 | | | | | | |  | | 145,613 | | | | 7,662 | | | | | | | | 4.40 | | | 1.33  (1.16–1.51) | | | | <0.001 | | | |  |  | | | | |
| ≥90 | | 105,770 | | | | | | | | | | 788 | | | 0.58 | | | | | 2.22  (1.73–2.84) | | | | | <0.001 | | | | | | |  | | 59,688 | | | | 3,879 | | | | | | | | 5.50 | | | 1.59  (1.36–1.82) | | | | <0.001 | | | |  |  | | | | |
| **Non-HDL-c, mg/dL** | | | | | | | | | | | | | | | | | | | | | | | | | | | | | | | | | | | | | | | | | | | | | | | | | | | | | | | | | | | | | | |
| <130 | | | 288,643 | | | | | | | | 1,202 | | | 0.33 | | | | (ref) | | | | | | | | | | |  | | | | | 109,894 | | | 5,738 | | | | | | | | 4.42 | | | | (ref) | | | | | |  | | | <0.001 | | | |  |
| 130-159 | | | 322,271 | | | | | | | | 1,252 | | | 0.30 | | | | 0.94  (0.82–1.02) | | | | 0.131 | | | | | | |  | | | | | 124,021 | | | 5,972 | | | | | | | | 4.01 | | | | 0.93  (0.89–0.96) | | | | <0.001 | |  | | |  | | | |  |
| 160-189 | | | 239,246 | | | | | | | | 985 | | | 0.32 | | | | 0.96  (0.78–1.04) | | | | 0.342 | | | | | | | <0.001 | | | | | 96,493 | | | 4,846 | | | | | | | | 4.17 | | | | 0.99  (0.96–1.03) | | | | 0.718 | | <0.001 | | |  | | | |  |
| 190-219 | | | 106,139 | | | | | | | | 502 | | | 0.37 | | | | 1.05  (0.95–1.17) | | | | 0.358 | | | | | | |  | | | | | 43,434 | | | 2,240 | | | | | | | | 4.29 | | | | 1.02  (0.97–1.07) | | | | 0.458 | |  | | |  | | | |  |
| ≥220 | | | 43,532 | | | | | | | | 260 | | | 0.47 | | | | 1.26  (1.10–1.44) | | | | <0.001 | | | | | | |  | | | | | 18,060 | | | 1,042 | | | | | | | | 4.83 | | | | 1.12  (1.05–1.20) | | | | <0.001 | |  | | |  | | | |  |
| **LDL-c, mg/dL** | | | | | | | | | | | | | | | | | | | | | | | | | | | | | | | | | | | | | | | | | | | | | | | | | | | | | | | | | | | | | | |
| <70 | | | 39,592 | | | | | | | | 222 | | | 0.44 | | | | (ref) | | | | | | | | | | |  | | | | | 20,450 | | | 1,177 | | | | | | | | 4.92 | | | | (ref) | | | | | |  | | | <0.001 | | | |  |
| 70-99 | | | 182,016 | | | | | | | | 853 | | | 0.37 | | | | 1.00  (0.86–1.16) | | | | 0.974 | | | | | | |  | | | | | 77,443 | | | 4,003 | | | | | | | | 4.37 | | | | 0.91  (0.85–0.97) | | | | 0.003 | |  | | |  | | | |  |
| 100-129 | | | 337,709 | | | | | | | | 1,333 | | | 0.31 | | | | 0.90  (0.78–1.04) | | | | 0.162 | | | | | | | 0.156 | | | | | 129,376 | | | 6,416 | | | | | | | | 4.15 | | | | 0.89  (0.84–0.95) | | | | <0.001 | | 0.325 | | |  | | | |  |
| 130-159 | | | 276,305 | | | | | | | | 1,059 | | | 0.30 | | | | 0.87  (0.75–1.00) | | | | 0.057 | | | | | | |  | | | | | 104,150 | | | 5,125 | | | | | | | | 4.09 | | | | 0.91  (0.86–0.97) | | | | 0.004 | |  | | |  | | | |  |
| ≥160 | | | 164,209 | | | | | | | | 734 | | | 0.35 | | | | 0.97  (0.84–1.13) | | | | 0.720 | | | | | | |  | | | | | 60,483 | | | 3,117 | | | | | | | | 4.28 | | | | 0.97  (0.91–1.04) | | | | 0.355 | |  | | |  | | | |  |
| **Triglycerides, mg/dL** | | | | | | | | | | | | | | | | | | | | | | | | | | | | | | | | | | | | | | | | | | | | | | | | | | | | | | | | | | | | | | |
| <100 | | | 443,795 | | | | | | | | 1,525 | | | 0.27 | | | | (ref) | | | | | | | | | | | <0.001 | | | | | 125,671 | | | 5,773 | | | | | | | | 3.84 | | | | (ref) | | | | | | <0.001 | | | <0.001 | | | |  |
| 100-149 | | | 300,007 | | | | | | | | 1,287 | | | 0.34 | | | | 1.06  (0.98–1.14) | | | | 0.125 | | | | | | |  |  |  |  |  | 133,368 | | | 6,921 | | | | | | | | 4.35 | | | | 1.05  (1.01–1.09) | | | | 0.006 | |  |  |  |  | | | |  |
| 150-199 | | | 137,884 | | | | | | | | 672 | | | 0.38 | | | | 1.10  (1.00–1.20) | | | | 0.053 | | | | | | |  |  |  |  |  | 70,690 | | | 3,731 | | | | | | | | 4.42 | | | | 1.04  (1.00–1.09) | | | | 0.048 | |  |  |  |  | | | |  |
| ≥200 | | | 118,145 | | | | | | | | 717 | | | 0.47 | | | | 1.25  (1.14–1.37) | | | | <0.001 | | | | | | |  |  |  |  |  | 62,173 | | | 3,413 | | | | | | | | 4.59 | | | | 1.09  (1.04–1.13) | | | | <0.001 | |  |  |  |  | | | |  |
| **Abdominal obesity** | | | | | | | | | | | | | | | | | | | | | | | | | | | | | | | | | | | | | | | | | | | | | | | | | | | | | | | | | | | | | | |
| Yes | | | 213,577 | | | | | | | | | 1,311 | | | 0.48 | | | 1.17  (1.09–1.25) | | | | <0.001 | | | | | | | - | | | | | 135,182 | | | 6,817 | | | | | | | | 4.20 | | | | 0.92  (0.90–0.95) | | | | <0.001 | | | - | | | <0.001 | |  |  |
| **Regular physical activity** | | | | | | | | | | | | | | | | | | | | | | | | | | | | | | | | | | | | | | | | | | | | | | | | | | | | | | | | | | | | | | |
| Yes | | | 201,979 | | | | | | | | | 737 | | | 0.28 | | | 1.20  (1.11–1.30) | | | | <0.001 | | | | | | | - | | | | | 59,799 | | | 2,032 | | | | | | | | 2.77 | | | | 1.26  (1.20–1.32) | | | | <0.001 | | - | | | <0.001 | | |  |  |

HRs were estimated with adjustment for age, sex, BMI, smoking, alcohol, exercise, hypertension, diabetes, dyslipidemia, and eGFR, excluding the primary exposure variable under analysis. CV, cardiovascular; other abbreviations as **Supplementary Table 2** and **3**.

**Supplementary Table 5. Associations of individual risk factors with MI risk by age and sex**

1. **Total population**

|  | | **Total** | | | | | | | | | | | | | | | | | **<40 years old** | | | | | | | | | | | | | | | | | | | | | | | | | | | | | | | | | | | **40-49 years old** | | | | | | | | | | | | | | | | | | | | | | | | | | | | | | | | | | | | | |
| --- | --- | --- | --- | --- | --- | --- | --- | --- | --- | --- | --- | --- | --- | --- | --- | --- | --- | --- | --- | --- | --- | --- | --- | --- | --- | --- | --- | --- | --- | --- | --- | --- | --- | --- | --- | --- | --- | --- | --- | --- | --- | --- | --- | --- | --- | --- | --- | --- | --- | --- | --- | --- | --- | --- | --- | --- | --- | --- | --- | --- | --- | --- | --- | --- | --- | --- | --- | --- | --- | --- | --- | --- | --- | --- | --- | --- | --- | --- | --- | --- | --- | --- | --- | --- | --- | --- | --- | --- | --- | --- | --- |
|  | | **N** | | **Events** | | | | **IR** | | | **HR**  **(95% CI)** | | | ***p*** | | | | ***p*_trend_** | **N** | | | **Events** | | | | | | | | | | **IR** | | | | | **HR**  **(95% CI)** | | | | | ***p*** | | | | | | | | | | ***p*_trend_** | | **N** | | | | | | | | **Events** | | | | | | | **IR** | | | | | | **HR**  **(95% CI)** | | | | | | | ***p*** | | | | | | | ***p*_trend_** | | |
| **BMI, kg/m2** | | | | | | | | | | | | | | | | | | | | | | | | | | | | | | | | | | | | | | | | | | | | | | | | | | | | | | | | | | | | | | | | | | | | | | | | | | | | | | | | | | | | | | | | | | | |
| <18.5 | | 238,814 | | 1,527 | | | 0.52 | | | 1.00  (0.95–1.05) | | | | 0.988 | | | |  | 136,039 | | | 139 | | | | | | | | | | 0.08 | | | | | 0.98  (0.82–1.16) | | | | | 0.776 | | | | <0.001 | | | | | | | | 41,859 | | | | | | | | 155 | | | | | | | 0.29 | | | | | | 0.87  (0.74–1.02) | | | | | | | 0.082 | | | | | | | <0.001 | | |
| 18.5-22.9 | | 2,492,083 | | 21,062 | | | 0.67 | | | (ref) | | | | | | | |  | 878,129 | | | 1,468 | | | | | | | | | | 0.13 | | | | | (ref) | | | | | | | | |  |  |  |  |  |  |  |  | 700,605 | | | | | | | | 3,436 | | | | | | | 0.38 | | | | | | (ref) | | | | | | | | | | | | | |  |  |  |
| 23.0-24.9 | | 1,530,969 | | 18,529 | | | 0.96 | | | 1.09  (1.07–1.11) | | | | <0.001 | | <0.001 | | | 376,021 | | | 1,163 | | | | | | | | | | 0.24 | | | | | 1.19  (1.10–1.29) | | | | | <0.001 | | | |  |  |  |  |  |  |  |  | 431,671 | | | | | | | | 3,319 | | | | | | | 0.60 | | | | | | 1.16  (1.10–1.21) | | | | | | | <0.001 | | | | | | |  |  |  |
| 25.0-29.9 | | 1,773,812 | | 24,842 | | | 1.11 | | | 1.18  (1.15–1.20) | | | | <0.001 | |  | | | 435,435 | | | 2,260 | | | | | | | | | | 0.40 | | | | | 1.58  (1.47–1.69) | | | | | <0.001 | | | |  |  |  |  |  |  |  |  | 487,357 | | | | | | | | 5,252 | | | | | | | 0.84 | | | | | | 1.36  (1.30–1.42) | | | | | | | <0.001 | | | | | | |  |  |  |
| ≥30.0 | | 214,174 | | 2,895 | | | 1.07 | | | 1.34  (1.29–1.40) | | | | <0.001 | |  | | | 76,779 | | | 644 | | | | | | | | | | 0.65 | | | | | 2.23  (2.02–2.47) | | | | | <0.001 | | | |  |  |  |  |  |  |  |  | 56,355 | | | | | | | | 719 | | | | | | | 1.00 | | | | | | 1.61  (1.48–1.75) | | | | | | | <0.001 | | | | | | |  |  |  |
| **Smoking** | | | | | | | | | | | | | | | | | | | | | | | | | | | | | | | | | | | | | | | | | | | | | | | | | | | | | | | | | | | | | | | | | | | | | | | | | | | | | | | | | | | | | | | | | | | |
| Non | | 3,827,796 | | 32,062 | | | 0.66 | | | (ref) | | | | | | | |  | 1,020,523 | | | | | | | | 1,533 | | | | | 0.12 | | | | | (ref) | | | | | | | | | <0.001 | | | | | | | | 1,041,334 | | | | | | 4,089 | | | | | | | 0.31 | | | | | | | (ref) | | | | | | | | | | | | |  | | | | |
| Ex | | 863,923 | | 11,782 | | | 1.09 | | | 1.10  (1.07–1.13) | | | | 0.043 | | <0.001 | | | 208,465 | | | 653 | | | | | | | | | | 0.24 | | | | | 1.14  (1.03–1.26) | | | | | 0.009 | | | |  |  |  |  |  |  |  |  | 243,998 | | | | | | | 2,060 | | | | | | | 0.66 | | | | | | 1.10  (1.04–1.17) | | | | | 0.002 | | | | | | | | <0.001 | | | | |
| Current | | 1,558,133 | | 25,011 | | | 1.28 | | | 2.02  (1.98–2.07) | | | | <0.001 | |  | | | 673,415 | | | 3,488 | | | | | | | | | | 0.40 | | | | | 1.99  (1.86–2.14) | | | | | <0.001 | | | |  |  |  |  |  |  |  |  | 432,515 | | | | | | | 6,732 | | | | | | | 1.23 | | | | | | 2.18  (2.07–2.29) | | | | | <0.001 | | | | | | | |  | | | | |
| **Hypertension** | | | | | | | | | | | | | | | | | | | | | | | | | | | | | | | | | | | | | | | | | | | | | | | | | | | | | | | | | | | | | | | | | | | | | | | | | | | | | | | | | | | | | | | | | | | |
| Yes | | 1,395,180 | | 28,453 | | | 1.66 | | | 1.25  (1.23–1.27) | | | <0.001 | | | | | - | 144,432 | | 1,164 | | | | | | | | | 0.63 | | | | | | 1.65  (1.54–1.76) | | | | | | <0.001 | | | | - | | | | | | | | 270,642 | | | | | 3,526 | | | | | | 1.02 | | | | | | | | | 1.40  (1.34–1.46) | | | | | <0.001 | | | | | | | | | - | | | |
| **Diabetes** | | | | | | | | | | | | | | | | | | | | | | | | | | | | | | | | | | | | | | | | | | | | | | | | | | | | | | | | | | | | | | | | | | | | | | | | | | | | | | | | | | | | | | | | | | | |
| Normo-  glycemia | | 4,347,864 | | 37,295 | | | 0.68 | | | (ref) | | | | | | | |  | 1,561,963 | | | | | | 4,054 | | | | | | | | 0.20 | | | | | (ref) | | | | | | | | <0.001 | | | | | | | | 1,216,685 | | | | 7,755 | | | | | | 0.50 | | | | | | | | | | (ref) | | | | | | | | | | | | | <0.001 | | | | |
| Prediabetes | | 1,407,599 | | 18,013 | | | 1.02 | | | 1.05  (1.03–1.07) | | | <0.001 | | <0.001 | | | | 300,804 | | 1,240 | | | | | | | | | 0.32 | | | | | 1.04  (0.98–1.11) | | | | | | 0.191 | | | | |  |  |  |  |  |  |  |  | 399,548 | | | | 3,419 | | | | | | 0.67 | | | | | | | | | | 1.01  (0.97–1.05) | | | | 0.670 | | | | | | | | |  |  |  |  |  |
| Diabetes | | 494,389 | | 13,547 | | | 2.28 | | | 1.57  (1.54–1.60) | | | <0.001 | |  | | | | 39,636 | | 380 | | | | | | | | | 0.75 | | | | | 1.57  (1.41–1.75) | | | | | | <0.001 | | | | |  |  |  |  |  |  |  |  | 101,614 | | | | 1,707 | | | | | | 1.33 | | | | | | | | | | 1.45  (1.37–1.53) | | | | <0.001 | | | | | | | | |  |  |  |  |  |
| **Systolic BP, mmHg** | | | | | | | | | | | | | | | | | | | | | | | | | | | | | | | | | | | | | | | | | | | | | | | | | | | | | | | | | | | | | | | | | | | | | | | | | | | | | | | | | | | | | | | | | | | |
| <100 | | 221,402 | | 968 | | | 0.34 | | | (ref) | | | | | | | |  | 88,092 | | | | 92 | | | | | | 0.08 | | | | | | (ref) | | | | | | | | | |  | | | | | | | | 74,010 | | | | 224 | | | | | | 0.24 | | | | | | | | | | (ref) | | | | | | | | | | | |  | | | | | |  |
| 100-119 | | 2,371,006 | | 17,055 | | | 0.57 | | | 1.14  (1.07–1.22) | | | <0.001 | | | | |  | 879,732 | | | | 1,636 | | | | | | 0.14 | | | | | | 1.02  (0.83–1.27) | | | | | 0.839 | | | | |  | | | | | | | | 721,388 | | | | 3,874 | | | | | | 0.42 | | | | | | | | | | 1.20  (1.05–1.38) | | | | 0.008 | | | | | | | |  | | | | | |  |
| 120-139 | | 2,959,527 | | 36,460 | | | 0.98 | | | 1.35  (1.26–1.44) | | | <0.001 | | <0.001 | | | | 844,366 | | | | 3,168 | | | | | | 0.29 | | | | | | 1.30  (1.05–1.61) | | | | | 0.016 | | | | | <0.001 | | | | | | | | 780,712 | | | | 6,870 | | | | | | 0.69 | | | | | | | | | | 1.46  (1.27–1.66) | | | | <0.001 | | | | | | | | <0.001 | | | | | |  |
| 140-159 | | 568,074 | | 11,163 | | | 1.60 | | | 1.53  (1.43–1.64) | | | <0.001 | |  | | | | 77,539 | | | | 612 | | | | | | 0.61 | | | | | | 1.90  (1.51–2.38) | | | | | <0.001 | | | | |  | | | | | | | | 118,115 | | | | 1,551 | | | | | | 1.03 | | | | | | | | | | 1.88  (1.63–2.17) | | | | <0.001 | | | | | | | |  | | | | | |  |
| ≥160 | | 129,843 | | 3,209 | | | 2.06 | | | 1.73  (1.61–1.87) | | <0.001 | | |  | | | | 12,674 | | | | 166 | | | | | | 1.03 | | | | | 2.75  (2.12–3.58) | | | | | | <0.001 | | | | |  | | | | | | | | | 23,622 | | | | 362 | | | | | | 1.22 | | | | | | | | | | 2.14  (1.80–2.53) | | | | <0.001 | | | | | | | | |  | | | | |
| **Diastolic BP, mmHg** | | | | | | | | | | | | | | | | | | | | | | | | | | | | | | | | | | | | | | | | | | | | | | | | | | | | | | | | | | | | | | | | | | | | | | | | | | | | | | | | | | | | | | | | | | | |
| <60 | 150,910 | | 832 | | 0.44 | | | | (ref) | | | | | | | | | <0.001 | | 56,909 | | | | | | 65 | | | | | 0.09 | | | | | | (ref) | | | | | | | | | | <0.001 | | | | | | | | 48,944 | | 141 | | | | | | | | | | | 0.23 | | | | | | | | (ref) | | | | | | | | | | | | | <0.001 | | |
| 60-69 | 1,107,039 | | 7,582 | | 0.54 | | | | 1.06  (0.98–1.13) | | | | 0.144 | | | | |  |  | 400,546 | | | | | | 559 | | | | | 0.11 | | | | | | 0.96  (0.74–1.24) | | | | | | | 0.728 | | |  |  |  |  |  |  |  |  | 335,298 | | 1,411 | | | | | | | | | | | 0.33 | | | | | | | | 1.18  (0.99–1.40) | | | | | 0.063 | | | | | | | |  |  |  |
| 70-79 | 2,268,533 | | 21,806 | | 0.76 | | | | 1.18  (1.11–1.27) | | | | <0.001 | | | | |  |  | 755,821 | | | | | | 1,844 | | | | | 0.19 | | | | | | 1.18  (0.92–1.52) | | | | | | | 0.184 | | |  |  |  |  |  |  |  |  | 620,480 | | 3,951 | | | | | | | | | | | 0.50 | | | | | | | | 1.34  (1.13–1.59) | | | | | <0.001 | | | | | | | |  |  |  |
| 80-89 | 2,103,395 | | 27,339 | | 1.03 | | | | 1.33  (1.24–1.42) | | | | <0.001 | | | | |  |  | 587,863 | | | | | | 2,353 | | | | | 0.31 | | | | | | 1.43  (1.11–1.83) | | | | | | | 0.005 | | |  |  |  |  |  |  |  |  | 553,425 | | 5,236 | | | | | | | | | | | 0.74 | | | | | | | | 1.61  (1.36–1.90) | | | | | <0.001 | | | | | | | |  |  |  |
| ≥90 | 619,975 | | 11,296 | | 1.47 | | | | 1.52  (1.42–1.63) | | | | <0.001 | | | | |  |  | 101,264 | | | | | | 853 | | | | | 0.66 | | | | | | 2.15  (1.66–2.78) | | | | | | | <0.001 | | |  |  |  |  |  |  |  |  | 159,700 | | 2,142 | | | | | | | | | | | 1.06 | | | | | | | | 2.02  (1.70–2.40) | | | | | <0.001 | | | | | | | |  |  |  |
| **Non-HDL-c, mg/dL** | | | | | | | | | | | | | | | | | | | | | | | | | | | | | | | | | | | | | | | | | | | | | | | | | | | | | | | | | | | | | | | | | | | | | | | | | | | | | | | | | | | | | | | | | | | |
| <130 | 2,589,305 | | 17,946 | | 0.55 | | | | (ref) | | | | | | | | <0.001 | | | 1,041,985 | | | | | 1,539 | | | | | | 0.11 | | | | | | (ref) | | | | | | | | | | | | | | <0.001 | | | | 700,724 | 2,867 | | | | | | | | | | | | | | | | 0.32 | | | | (ref) | | | | | | | | | | | | | | <0.001 | |
| 130-159 | 1,913,101 | | 20,646 | | 0.85 | | | | 1.26  (1.24–1.29) | | | | <0.001 | | | |  |  |  | 510,962 | | | | | 1,477 | | | | | | 0.22 | | | | | | 1.33  (1.23–1.43) | | | | | | 0.914 | | | | | | | |  |  |  |  | 549,841 | 3,609 | | | | | | | | | | | | | | | | 0.51 | | | | 1.29  (1.22–1.35) | | | | | | | | <0.001 | | | | | |  |  |
| 160-189 | 1,150,763 | | 17,263 | | | 1.19 | | | 1.59  (1.56–1.63) | | | | 0.020 | | | |  |  |  | 242,787 | | | | | 1,404 | | | | | | 0.45 | | | | | | 2.10  (1.94–2.27) | | | | | | <0.001 | | | | | | | |  |  |  |  | 314,844 | 3,377 | | | | | | | | | | | | | | | | 0.84 | | | | 1.78  (1.69–1.87) | | | | | | | | 0.096 | | | | | |  |  |
| 190-219 | 436,771 | | 8,769 | | | 1.60 | | | 2.04  (1.99–2.10) | | | | <0.001 | | | |  |  |  | 79,766 | | | | | 804 | | | | | | 0.78 | | | | | | 3.17  (2.89–3.47) | | | | | | <0.001 | | | | | | | |  |  |  |  | 112,950 | 1,966 | | | | | | | | | | | | | | | | 1.37 | | | | 2.58  (2.43–2.73) | | | | | | | | 0.004 | | | | | |  |  |
| ≥220 | 159,912 | | 4,231 | | | 2.12 | | | 2.64  (2.55–2.73) | | | | <0.001 | | | |  |  |  | 26,903 | | | | | 450 | | | | | | 1.30 | | | | | | 4.84  (4.33–5.41) | | | | | | <0.001 | | | | | | | |  |  |  |  | 39,488 | 1,062 | | | | | | | | | | | | | | | | 2.13 | | | | 3.70  (3.44–3.98) | | | | | | | | <0.001 | | | | | |  |  |
| **LDL-c, mg/dL** | | | | | | | | | | | | | | | | | | | | | | | | | | | | | | | | | | | | | | | | | | | | | | | | | | | | | | | | | | | | | | | | | | | | | | | | | | | | | | | | | | | | | | | | | | | |
| <70 | 475,298 | | 4,370 | | | 0.74 | | | (ref) | | | | | | | | 0.059 | | | 190,321 | | | | | 404 | | | | | 0.16 | | | | | | | (ref) | | | | | | | | | | | | | | <0.001 | | | | 116,751 | | 737 | | | | | | | | | | | | | | 0.50 | | | | | (ref) | | | | | | | | | | | | | <0.001 | | |
| 70-99 | 1,703,244 | | 13,483 | | | 0.63 | | | 1.00  (0.97–1.03) | | | | 0.919 | | | |  |  |  | 674,961 | | | | | 1,253 | | | | | 0.14 | | | | | | | 0.92  (0.83–1.03) | | | | | 0.166 | | | | | | | | |  |  |  |  | 457,891 | | 2,289 | | | | | | | | | | | | | | 0.39 | | | | | 0.97  (0.89–1.05) | | | | | | | 0.467 | | | | | |  |  |  |
| 100-129 | 2,212,393 | | 22,326 | | | 0.80 | | | 1.17  (1.13–1.21) | | | | <0.001 | | | |  |  |  | 666,476 | | | | | 1,790 | | | | | 0.21 | | | | | | | 1.09  (0.97–1.21) | | | | | 0.142 | | | | | | | | |  |  |  |  | 642,418 | | 4,151 | | | | | | | | | | | | | | 0.50 | | | | | 1.18  (1.09–0.74) | | | | | | | <0.001 | | | | | |  |  |  |
| 130-159 | 1,294,595 | | 17,706 | | | 1.09 | | | 1.43  (1.39–1.48) | | | | <0.001 | | | |  |  |  | 281,961 | | | | | 1,372 | | | | | 0.38 | | | | | | | 1.56  (1.39–1.74) | | | | | <0.001 | | | | | | | | |  |  |  |  | 361,073 | | 3,385 | | | | | | | | | | | | | | 0.73 | | | | | 1.53  (1.42–1.66) | | | | | | | <0.001 | | | | | |  |  |  |
| ≥160 | 564,322 | | 10,970 | | | 1.55 | | | 1.93  (1.87–2.00) | | | | <0.001 | | | |  |  |  | 88,684 | | | | | 855 | | | | | 0.75 | | | | | | | 2.62  (2.32–2.96) | | | | | <0.001 | | | | | | | | |  |  |  |  | 139,714 | | 2,319 | | | | | | | | | | | | | | 1.30 | | | | | 2.46  (2.27–2.68) | | | | | | | <0.001 | | | | | |  |  |  |
| **Triglycerides, mg/dL** | | | | | | | | | | | | | | | | | | | | | | | | | | | | | | | | | | | | | | | | | | | | | | | | | | | | | | | | | | | | | | | | | | | | | | | | | | | | | | | | | | | | | | | | | | | |
| <100 | 2,805,260 | | 19,056 | | | 0.54 | | | (ref) | | | | | | | | <0.001 | | | 1,025,571 | | | | | 1,538 | | | | | 0.12 | | | | | | | (ref) | | | | | | | | | | | | | <0.001 | | | | | 793,014 | | 3,226 | | | | | | | | | | | | | 0.32 | | | | | | (ref) | | | | | | | | | | | | <0.001 | | | |
| 100-149 | 1,669,525 | | 20,265 | | | 0.97 | | | 1.24  (1.21–1.26) | | | | <0.001 | | | |  |  |  | 428,981 | | | | | 1,366 | | | | | 0.25 | | | | | | | 1.30  (1.20–1.40) | | | | <0.001 | | | | | | | | |  |  |  |  |  | 439,169 | | 3,264 | | | | | | | | | | | | | 0.58 | | | | | | 1.24  (1.18–1.30) | | | | | | <0.001 | | | | | |  |  |  |  |
| 150-199 | 844,984 | | 12,844 | | | 1.21 | | | 1.39  (1.36–1.43) | | | | <0.001 | | | |  |  |  | 205,143 | | | | | 923 | | | | | 0.35 | | | | | | | 1.45  (1.33–1.58) | | | | <0.001 | | | | | | | | |  |  |  |  |  | 221,637 | | 2,452 | | | | | | | | | | | | | 0.87 | | | | | | 1.49  (1.41–1.57) | | | | | | <0.001 | | | | | |  |  |  |  |
| ≥200 | 930,083 | | 16,690 | | | 1.43 | | | 1.59  (1.56–1.62) | | | | <0.001 | | | |  |  |  | 242,708 | | | | | 1,847 | | | | | 0.59 | | | | | | | 1.97  (1.82–2.13) | | | | <0.001 | | | | | | | | |  |  |  |  |  | 264,027 | | 3,939 | | | | | | | | | | | | | 1.17 | | | | | | 1.68  (1.60–1.77) | | | | | | <0.001 | | | | | |  |  |  |  |
| **Abdominal obesity** | | | | | | | | | | | | | | | | | | | | | | | | | | | | | | | | | | | | | | | | | | | | | | | | | | | | | | | | | | | | | | | | | | | | | | | | | | | | | | | | | | | | | | | | | | | |
| Yes | 1,176,285 | | 19,950 | | | 1.36 | | | 1.14  (1.12–1.16) | | | | <0.001 | | | | - | | | 245,998 | | | | 1,609 | | | | | 0.51 | | | | | | | | 1.47  (1.38–1.56) | | | <0.001 | | | | | | | | | - | | | | | | 272,361 | | 3,302 | | | | | | | | | | | 0.95 | | | | | | | | 1.24  (1.19–1.29) | | | | <0.001 | | | | | | | - | | | | |
| **Regular physical activity** | | | | | | | | | | | | | | | | | | | | | | | | | | | | | | | | | | | | | | | | | | | | | | | | | | | | | | | | | | | | | | | | | | | | | | | | | | | | | | | | | | | | | | | | | | | |
| Yes | 1,121,280 | | 13,292 | | | 0.94 | | | 1.09  (1.07–1.11) | | | | <0.001 | | | | - | | | 254,136 | | | 855 | | | | | 0.26 | | | | | | | | | 0.97  (0.90–1.04) | | 0.361 | | | | | | | | | - | | | | | | | 304,750 | | 2,236 | | | | | | | | | 0.57 | | | | | | | | | | 1.05  (1.00–1.10) | | | 0.049 | | | | | | | - | | | | | |

|  | | | | **50-64 years old** | | | | | | | | | | | | | | | | | | | | | | | | | | | | | | **≥65 years old** | | | | | | | | | | | | | | | | | | | | | | | | | | | | | | | | | | | |  | | | |
| --- | --- | --- | --- | --- | --- | --- | --- | --- | --- | --- | --- | --- | --- | --- | --- | --- | --- | --- | --- | --- | --- | --- | --- | --- | --- | --- | --- | --- | --- | --- | --- | --- | --- | --- | --- | --- | --- | --- | --- | --- | --- | --- | --- | --- | --- | --- | --- | --- | --- | --- | --- | --- | --- | --- | --- | --- | --- | --- | --- | --- | --- | --- | --- | --- | --- | --- | --- | --- | --- | --- | --- | --- | --- |
|  | | | | **N** | | **Events** | | | | | | | **IR** | | | | | | **HR**  **(95% CI)** | | | | | | | ***p*** | ***p*_trend_** | | | | | | **N** | | | | | | **Events** | | | | | | | | **IR** | | | **HR**  **(95% CI)** | | | | | | | ***p*** | | | | | ***p*_trend_** | | | | | | | | ***p*_interaction_** | | | |
| **BMI, kg/m2** | | | | | | | | | | | | | | | | | | | | | | | | | | | | | | | | | | | | | | | | | | | | | | | | | | | | | | | | | | | | | | | | | | | | | | | | | |
| <18.5 | | | | 32,068 | | 392 | | | | | | | 1.00 | | | | | | 0.99  (0.89–1.09) | | | | | | | 0.767 | | <0.001 | | | | | 28,848 | | | | | | 841 | | | | | | | | | 3.08 | | | 1.05  (0.98–1.13) | | | | | | 0.204 | | | | | <0.001 | | | | | | | | <0.001 | | | |
| 18.5-22.9 | | | | 646,731 | | 7,845 | | | | | | | 0.96 | | | | | | (ref) | | | | | | | | |  |  |  |  |  | 266,618 | | | | | | 8,313 | | | | | | | | | 2.82 | | | (ref) | | | | | | | | | | |  |  |  |  |  |  |  |  |  | | | |
| 23.0-24.9 | | | | 530,856 | | 7,988 | | | | | | | 1.19 | | | | | | 1.11  (1.08–1.15) | | | | | | | <0.001 | |  |  |  |  |  | 192,421 | | | | | | 6,059 | | | | | | | | | 2.72 | | | 0.98  (0.94–1.01) | | | | | | 0.161 | | | | |  |  |  |  |  |  |  |  |  | | | |
| 25.0-29.9 | | | | 624,841 | | 10,481 | | | | | | | 1.33 | | | | | | 1.16  (1.12–1.19) | | | | | | | <0.001 | |  |  |  |  |  | 226,179 | | | | | | 6,849 | | | | | | | | | 2.57 | | | 0.94  (0.91–0.97) | | | | | | <0.001 | | | | |  |  |  |  |  |  |  |  |  | | | |
| ≥30.0 | | | | 59,804 | | 953 | | | | | | | 1.27 | | | | | | 1.19  (1.11–1.27) | | | | | | | <0.001 | |  |  |  |  |  | 21,236 | | | | | | 579 | | | | | | | | | 2.31 | | | 0.88  (0.80–0.95) | | | | | | 0.002 | | | | |  |  |  |  |  |  |  |  |  | | | |
| **Smoking** | | | | | | | | | | | | | | | | | | | | | | | | | | | | | | | | | | | | | | | | | | | | | | | | | | | | | | | | | | | | | | | | | | | | | | | | | |
| Non | | | | 1,240,131 | 12,172 | | | | | | | | | | | 0.77 | | | (ref) | | | | | | | | | <0.001 | | | | | | 525,808 | 14,268 | | | | | | | | 2.33 | | | | | | | | | | | (ref) | | | | | | | | <0.001 | | | | | | | | <0.001 | | | |
| Ex | | | | 300,261 | 5,259 | | | | | | | | | | | 1.40 | | | 1.05  (1.01–1.09) | | 0.011 | | | | | | |  |  |  |  |  |  | 111,199 | 3,810 | | | | | | | | 3.11 | | | | | | | | | | 1.12  (1.07–1.16) | | | | <0.001 | | | | |  |  |  |  |  |  |  |  |  | | | |
| Current | | | | 353,908 | 10,228 | | | | | | | | | | | 2.36 | | | 1.96  (1.90–2.03) | | <0.001 | | | | | | |  |  |  |  |  |  | 98,295 | 4,563 | | | | | | | | 4.49 | | | | | | | | | | 1.74  (1.67–1.81) | | | | <0.001 | | | | |  |  |  |  |  |  |  |  |  | | | |
| **Hypertension** | | | | | | | | | | | | | | | | | | | | | | | | | | | | | | | | | | | | | | | | | | | | | | | | | | | | | | | | | | | | | | | | | | | | | | | | | |
| Yes | | | | 600,316 | | | 11,096 | | | | | | | | | 1.48 | | | 1.24  (1.21–1.27) | | <0.001 | | | | | | | - | | | | | | 379,790 | | | | 12,667 | | | | 2.95 | | | | | | | | | | 1.15  (1.12–1.18) | | | | | <0.001 | | | | | - | | | | | | | | <0.001 | | | |
| **Diabetes** | | | | | | | | | | | | | | | | | | | | | | | | | | | | | | | | | | | | | | | | | | | | | | | | | | | | | | | | | | | | | | | | | | | | | | | | | |
| Normoglycemia | | | | 1,161,480 | | | | 14,233 | | | | | | | | 0.97 | | (ref) | | | | | | | | | | | | | <0.001 | | | 407,736 | | | | 11,253 | | | 2.40 | | | | | | | | | | (ref) | | | | | | | | | |  | | | | | | | | | <0.001 | | | |
| Prediabetes | | | | 509,040 | | | 7,478 | | | | | | | | | 1.17 | | 1.03  (1.00–1.06) | | | | | 0.058 | | | | | | | |  |  |  | 198,207 | | | | 5,876 | | | 2.60 | | | | | | | | | | 1.05  (1.02–1.09) | | | | | | 0.001 | | | | | | | | <0.001 | | | | |  | | | |
| Diabetes | | | | 223,780 | | | 5,948 | | | | | | | | | 2.16 | | 1.60  (1.55–1.65) | | | | | <0.001 | | | | | | | |  |  |  | 129,359 | | | 5,512 | | | | 3.91 | | | | | | | | | | 1.56  (1.51–1.61) | | | | | | <0.001 | | | | | | | |  | | | | |  | | | |
| **Systolic BP, mmHg** | | | | | | | | | | | | | | | | | | | | | | | | | | | | | | | | | | | | | | | | | | | | | | | | | | | | | | | | | | | | | | | | | | | | | | | | | |
| <100 | | | | 50,473 | | | | | 413 | | | | | | | | 0.65 | | (ref) | | | | | | | | | | | |  | | | 8,827 | | 239 | | | | 2.46 | | | | | | | | | | (ref) | | | | | | | | | |  | | | | | | | | | | | <0.001 | | |
| 100-119 | | | | 609,874 | | | | | 7,188 | | | | | | | | 0.93 | | 1.18  (1.07–1.31) | | | | | 0.001 | | | | | | |  | | | 160,012 | | 4,357 | | | | 2.39 | | | | | | | | | | 0.97  (0.85–1.11) | | | | | | | 0.662 | | | | | | | | |  | | | | |  | | |
| 120-139 | | | | 953,152 | | | | | 14,702 | | | | | | | | 1.22 | | 1.35  (1.22–1.49) | | | | | <0.001 | | | | | | | <0.001 | | | 381,297 | | 11,720 | | | | 2.69 | | | | | | | | | | 1.08  (0.95–1.23) | | | | | | | 0.236 | | | | | | | | | <0.001 | | | | |  | | |
| 140-159 | | | | 229,942 | | | | | 4,249 | | | | | | | | 1.48 | | 1.52  (1.37–1.69) | | | | | <0.001 | | | | | | |  | | | 142,478 | | 4,751 | | | | 2.94 | | | | | | | | | | 1.17  (1.03–1.33) | | | | | | | 0.020 | | | | | | | | |  | | | | |  | | |
| ≥160 | | | | 50,859 | | | | | 1,107 | | | | | | | | 1.76 | | 1.75  (1.56–1.96) | | | | | <0.001 | | | | | |  | | | | 42,688 | | 1,574 | | | | 3.35 | | | | | | | | | | 1.30  (1.14–1.50) | | | | | | | <0.001 | | | | | | | | |  | | | | |  | | |
| **Diastolic BP, mmHg** | | | | | | | | | | | | | | | | | | | | | | | | | | | | | | | | | | | | | | | | | | | | | | | | | | | | | | | | | | | | | | | | | | | | | | | | | |
| <60 | 34,405 | | | | | | | | | 314 | | | | | | 0.72 | | | (ref) | | | | | | | | | | | | |  | | 10,652 | | | | 312 | | | | | | 2.66 | | | | | (ref) | | | | | | | |  | | | | | | | | | |  | | | | <0.001 | | |
| 60-69 | 278,331 | | | | | | | | | 2,917 | | | | | | 0.83 | | | 1.04  (0.92–1.17) | | | | | | 0.539 | | | | | | |  | | 92,864 | | | | 2,695 | | | | | | 2.56 | | | | | 1.01  (0.89–1.13) | | | | | | | | 0.929 | | | | | | | | | |  | | | |  | | |
| 70-79 | | 649,250 | | | | | | | | | | 8,785 | | | 1.07 | | | | | 1.17  (1.05–1.31) | | | | | 0.005 | | | | | | | <0.001 | | 242,982 | | | | 7,226 | | | | | | | | 2.60 | | | | | | | | | | 1.04  (0.93–1.17) | | | 0.456 | | | <0.001 | | | | | | |  | | | | |
| 80-89 | | 686,011 | | | | | | | | | | 11,093 | | | 1.28 | | | | | 1.28  (1.15–1.44) | | | | | <0.001 | | | | | | |  | | 276,096 | | | | 8,657 | | | | | | | | 2.75 | | | | | | | | | | 1.10  (0.98–1.23) | | | 0.093 | | |  | | | | | | |  | | | | |
| ≥90 | | 246,303 | | | | | | | | | | 4,550 | | | 1.48 | | | | | 1.41  (1.26–1.58) | | | | | <0.001 | | | | | | |  | | 112,708 | | | | 3,751 | | | | | | | | 2.95 | | | | | | | | | | 1.18  (1.06–1.33) | | | 0.004 | | |  | | | | | | |  | | | | |
| **Non-HDL-c, mg/dL** | | | | | | | | | | | | | | | | | | | | | | | | | | | | | | | | | | | | | | | | | | | | | | | | | | | | | | | | | | | | | | | | | | | | | | | | | |
| <130 | | | 591,275 | | | | | | | | 6,772 | | | 0.91 | | | | (ref) | | | | | | | | | | |  | | | | | 255,321 | | | 6,768 | | | | | | | | 2.39 | | | | | | | | | | (ref) | | | | | | | |  | | | | | <0.001 | | | | |  |
| 130-159 | | | 618,456 | | | | | | | | 8,496 | | | 1.09 | | | | 1.24  (1.20–1.28) | | | | <0.001 | | | | | | |  | | | | | 233,842 | | | 7,064 | | | | | | | | 2.63 | | | | | | | | | | 1.18  (1.14–1.22) | | | <0.001 | | | | |  | | | | |  | | | | |  |
| 160-189 | | | 435,088 | | | | | | | | 7,163 | | | 1.30 | | | | 1.50  (1.45–1.55) | | | | <0.001 | | | | | | | <0.001 | | | | | 158,044 | | | 5,319 | | | | | | | | 2.91 | | | | | | | | | | 1.36  (1.31–1.41) | | | <0.001 | | | | | <0.001 | | | | |  | | | | |  |
| 190-219 | | | 180,448 | | | | | | | | 3,604 | | | 1.58 | | | | 1.86  (1.78–1.93) | | | | <0.001 | | | | | | |  | | | | | 63,607 | | | 2,395 | | | | | | | | 3.25 | | | | | | | | | | 1.56  (1.49–1.64) | | | <0.001 | | | | |  | | | | |  | | | | |  |
| ≥220 | | | 69,033 | | | | | | | | 1,624 | | | 1.87 | | | | 2.22  (2.10–2.35) | | | | <0.001 | | | | | | |  | | | | | 24,488 | | | 1,095 | | | | | | | | 3.90 | | | | | | | | | | 1.89  (1.78–2.02) | | | <0.001 | | | | |  | | | | |  | | | | |  |
| **LDL-c, mg/dL** | | | | | | | | | | | | | | | | | | | | | | | | | | | | | | | | | | | | | | | | | | | | | | | | | | | | | | | | | | | | | | | | | | | | | | | | | |
| <70 | | | 114,139 | | | | | | | | 1,710 | | | 1.21 | | | | (ref) | | | | | | | | | | |  | | | | | 54,087 | | | 1,519 | | | | | | | | 2.60 | | | | | | | | | | (ref) | | | | | | | |  | | | | | <0.001 | | | | |  |
| 70-99 | | | 398,665 | | | | | | | | 5,116 | | | 1.02 | | | | 1.01  (0.96–1.07) | | | | 0.713 | | | | | | |  | | | | | 171,727 | | | 4,825 | | | | | | | | 2.51 | | | | | | | | | | 1.03  (0.97–1.09) | | | 0.322 | | | | |  | | | | |  | | | | |  |
| 100-129 | | | 653,239 | | | | | | | | 8,949 | | | 1.08 | | | | 1.18  (1.12–1.24) | | | | <0.001 | | | | | | | <0.001 | | | | | 250,260 | | | 7,436 | | | | | | | | 2.60 | | | | | | | | | | 1.13  (1.07–1.19) | | | <0.001 | | | | | <0.001 | | | | |  | | | | |  |
| 130-159 | | | 478,788 | | | | | | | | 7,365 | | | 1.22 | | | | 1.41  (1.33–1.48) | | | | <0.001 | | | | | | |  | | | | | 172,773 | | | 5,584 | | | | | | | | 2.79 | | | | | | | | | | 1.28  (1.21–1.35) | | | <0.001 | | | | |  | | | | |  | | | | |  |
| ≥160 | | | 249,469 | | | | | | | | 4,519 | | | 1.44 | | | | 1.79  (1.69–1.89) | | | | <0.001 | | | | | | |  | | | | | 86,455 | | | 3,277 | | | | | | | | 3.27 | | | | | | | | | | 1.57  (1.47–1.67) | | | <0.001 | | | | |  | | | | |  | | | | |  |
| **Triglycerides, mg/dL** | | | | | | | | | | | | | | | | | | | | | | | | | | | | | | | | | | | | | | | | | | | | | | | | | | | | | | | | | | | | | | | | | | | | | | | | | |
| <100 | | | 727,549 | | | | | | | | 7,571 | | | 0.82 | | | | (ref) | | | | | | | | | | | <0.001 | | | | | 259,126 | | | 6,721 | | | | | | | | 2.29 | | | | | | | | | | (ref) | | | | | | | | <0.001 | | | | | <0.001 | | | | |  |
| 100-149 | | | 561,527 | | | | | | | | 8,182 | | | 1.16 | | | | 1.20  (1.16–1.24) | | | | <0.001 | | | | | | |  |  |  |  |  | 239,848 | | | 7,453 | | | | | | | | 2.73 | | | | | | | | | | 1.19  (1.15–1.23) | | | <0.001 | | | | |  |  |  |  |  |  | | | | |  |
| 150-199 | | | 294,398 | | | | | | | | 5,200 | | | 1.40 | | | | 1.31  (1.26–1.36) | | | | <0.001 | | | | | | |  |  |  |  |  | 123,806 | | | 4,269 | | | | | | | | 3.01 | | | | | | | | | | 1.30  (1.25–1.35) | | | <0.001 | | | | |  |  |  |  |  |  | | | | |  |
| ≥200 | | | 310,826 | | | | | | | | 6,706 | | | 1.72 | | | | 1.43  (1.39–1.48) | | | | <0.001 | | | | | | |  |  |  |  |  | 112,522 | | | 4,198 | | | | | | | | 3.26 | | | | | | | | | | 1.38  (1.33–1.44) | | | <0.001 | | | | |  |  |  |  |  |  | | | | |  |
| **Abdominal obesity** | | | | | | | | | | | | | | | | | | | | | | | | | | | | | | | | | | | | | | | | | | | | | | | | | | | | | | | | | | | | | | | | | | | | | | | | | |
| Yes | | | 434,187 | | | | | | | | | 7,841 | | | 1.44 | | | 1.11  (1.08–1.14) | | | | <0.001 | | | | | | | - | | | | | 223,739 | | | 7,198 | | | | | | | | 2.78 | | | | | | | | | | 1.01  (0.98–1.04) | | | 0.399 | | | | | | - | | | | | <0.001 | | |  |  |
| **Regular physical activity** | | | | | | | | | | | | | | | | | | | | | | | | | | | | | | | | | | | | | | | | | | | | | | | | | | | | | | | | | | | | | | | | | | | | | | | | | |
| Yes | | | 412,891 | | | | | | | | | 5,863 | | | 1.13 | | | 1.07  (1.04–1.10) | | | | <0.001 | | | | | | | - | | | | | 149,503 | | | 4,338 | | | | | | | | 2.50 | | | | | | | | | | 1.13  (1.09–1.17) | | | <0.001 | | | | | - | | | | | <0.001 | | | |  |  |

1. **Men**

|  | **Total** | | | | | | | | | | | | | | | | | | | | | **<40 years old** | | | | | | | | | | | | | | | | | | | | | | | | | | | | | | | | | | | | | | | | **40-49 years old** | | | | | | | | | | | | | | | | | | | | | | |
| --- | --- | --- | --- | --- | --- | --- | --- | --- | --- | --- | --- | --- | --- | --- | --- | --- | --- | --- | --- | --- | --- | --- | --- | --- | --- | --- | --- | --- | --- | --- | --- | --- | --- | --- | --- | --- | --- | --- | --- | --- | --- | --- | --- | --- | --- | --- | --- | --- | --- | --- | --- | --- | --- | --- | --- | --- | --- | --- | --- | --- | --- | --- | --- | --- | --- | --- | --- | --- | --- | --- | --- | --- | --- | --- | --- | --- | --- | --- | --- | --- | --- | --- | --- | --- |
|  | | **N** | **Events** | | | | **IR** | | | | **HR**  **(95% CI)** | ***p*** | | | | | | ***p*_trend_** | | | | **N** | | | | | | **Events** | | | | | | | | | | | **IR** | | | | | | **HR**  **(95% CI)** | | | | | | ***p*** | | | | | | | | | ***p*_trend_** | | **N** | | | | **Events** | | | | | | **IR** | | | | | **HR**  **(95% CI)** | | | ***p*** | | | | ***p*_trend_** |
| **BMI, kg/m2** | | | | | | | | | | | | | | | | | | | | | | | | | | | | | | | | | | | | | | | | | | | | | | | | | | | | | | | | | | | | | | | | | | | | | | | | | | | | | | | | | | | | |
| <18.5 | | 72,269 | 933 | | | | 1.10 | | | | 0.96  (0.90–1.03) | 0.282 | | | | | |  | | | | 28,778 | | | | | | 54 | | | | | | | | | | | 0.14 | | | | | | 0.78  (0.59–1.03) | | | | | | | 0.075 | | | | | | | |  | | 12,816 | | | | | | 94 | | | | | 0.58 | | | | | 0.81  (0.66–1.00) | | 0.044 | | | |  |
| 18.5-22.9 | | 109,2707 | 14,073 | | | | 1.03 | | | | (ref) | | | |  | | | | | | | 421,828 | | | | | | 1,098 | | | | | | | | | | | 0.20 | | | | | | (ref) | | | | | | | | | | | | | | |  | | 258,213 | | | | | | 2,405 | | | | | 0.73 | | | | | (ref) | | | | | |  |
| 23.0-24.9 | | 882,703 | 13,410 | | | | 1.21 | | | | 1.11  (1.08–1.14) | <0.001 | | | | <0.001 | | | | | | 290,363 | | | | | | 1,053 | | | | | | | | | | | 0.28 | | | | | | 1.18  (1.08–1.28) | | | | | | | <0.001 | | | | | | | | <0.001 | | 238,396 | | | | | | 2,771 | | | | | 0.91 | | | | | 1.17  (1.11–1.24) | | <0.001 | | | | <0.001 |
| 25.0-29.9 | | 109,5910 | 18,118 | | | | 1.31 | | | | 1.20  (1.17–1.23) | <0.001 | | | |  | | | | | | 367,511 | | | | | | 2,179 | | | | | | | | | | | 0.46 | | | | | | 1.59  (1.47–1.72) | | | | | | | <0.001 | | | | | | | |  | | 314,407 | | | | | | 4,659 | | | | | 1.16 | | | | | 1.38  (1.32–1.46) | | <0.001 | | | |  |
| ≥30.0 | | 118,206 | 1,922 | | | | 1.29 | | | | 1.41  (1.35–1.48) | <0.001 | | | |  | | | | | | 61,126 | | | | | | 605 | | | | | | | | | | | 0.77 | | | | | | 2.22  (2.00–2.47) | | | | | | | <0.001 | | | | | | | |  | | 30,488 | | | | | | 609 | | | | | 1.58 | | | | | 1.67  (1.52–1.82) | | <0.001 | | | |  |
| **Smoking** | | | | | | | | | | | | | | | | | | | | | | | | | | | | | | | | | | | | | | | | | | | | | | | | | | | | | | | | | | | | | | | | | | | | | | | | | | | | | | | | | | | | |
| Non | | 1,001,024 | 13,032 | | | | 1.04 | | | | (ref) | | | |  | | | | | | | 348,753 | | | | | | 926 | | | | | | | | | | | 0.20 | | | | | | (ref) | | | | | | | | | | | | |  | | | | 224,545 | | | | | | 1,975 | | | | | 0.69 | | | | | (ref) | | | | | |  |
| Ex | | 808,659 | 11,465 | | | | 1.13 | | | | 1.09  (1.07–1.12) | <0.001 | | | | <0.001 | | | | | | 184,671 | | | | | | 628 | | | | | | | | | | | 0.26 | | | | | | 1.14  (1.03–1.26) | | | | | | | 0.014 | | | | | | | | <0.001 | | 228,519 | | | | | | 1,994 | | | | | 0.68 | | | | | 1.09  (1.02–1.16) | | 0.010 | | | | <0.001 |
| Current | | 1,452,112 | 23,959 | | | | 1.31 | | | | 1.97  (1.93–2.01) | <0.001 | | | |  | | | | | | 636,182 | | | | | | 3,435 | | | | | | | | | | | 0.42 | | | | | | 2.01  (1.87–2.17) | | | | | | | <0.001 | | | | | | | |  | | 401,256 | | | | | | 6,569 | | | | | 1.29 | | | | | 2.18  (2.07–2.30) | | <0.001 | | | |  |
| **Hypertension** | | | | | | | | | | | | | | | | | | | | | | | | | | | | | | | | | | | | | | | | | | | | | | | | | | | | | | | | | | | | | | | | | | | | | | | | | | | | | | | | | | | | |
| Yes | | 780,990 | 18,909 | | | | 1.99 | | | | 1.23  (1.20–1.25) | <0.001 | | | | | - | | | | | 127,945 | | | | | | 1,133 | | | | | | | | | | 0.69 | | | | | | | 1.65  (1.54–1.77) | | | | | | | <0.001 | | | | | | | | - | | 178,473 | | | | | | 3,100 | | | | | 1.37 | | | | | 1.39  (1.33–1.45) | | <0.001 | | | | - |
| **Diabetes** | | | | | | | | | | | | | | | | | | | | | | | | | | | | | | | | | | | | | | | | | | | | | | | | | | | | | | | | | | | | | | | | | | | | | | | | | | | | | | | | | | | | |
| Normo-glycemia | | 2,110,224 | 25,563 | | | | 0.96 | | | | (ref) | | | | | |  | | | | | 910,342 | | | | | | | 3,461 | | | | | | | | 0.29 | | | | | | | (ref) | | | | | | | | | | | | | | | |  | | 537,980 | | | | | | 6,039 | | | | | | 0.88 | | | | (ref) | | | | | |  |
| Prediabetes | | 844,976 | 13,468 | | | | 1.28 | | | | 1.06  (1.03–1.08) | <0.001 | | | | | <0.001 | | | | | 226,609 | | | | | | 1,164 | | | | | | | | | | 0.40 | | | | | 1.05  (0.98–1.12) | | | | | | | | | 0.152 | | | | | | | | <0.001 | | 242,535 | | | | | | 2,984 | | | | | | 0.97 | | | | 1.01  (0.97–1.06) | | 0.604 | | | | <0.001 |
| Diabetes | | 306,595 | 9,425 | | | | 2.58 | | | | 1.49  (1.45–1.53) | <0.001 | | | | |  | | | | | 32,655 | | | | | | 364 | | | | | | | | | | 0.87 | | | | | 1.55  (1.39–1.74) | | | | | | | | | <0.001 | | | | | | | |  | | 73,805 | | | | | | 1,515 | | | | | | 1.63 | | | | 1.38  (1.31–1.47) | | <0.001 | | | |  |
| **Systolic BP, mmHg** | | | | | | | | | | | | | | | | | | | | | | | | | | | | | | | | | | | | | | | | | | | | | | | | | | | | | | | | | | | | | | | | | | | | | | | | | | | | | | | | | | | | |
| <100 | | 45,444 | 519 | | | | 0.92 | | | | (ref) | | | | | |  | | | | | 13,637 | | | | | | | | 37 | | | | | | 0.21 | | | | | | | (ref) | | | | | | | | | | | | | | | | |  | | 13,963 | | | | | | 101 | | | | | | 0.57 | | | | (ref) | | | | | |  |
| 100-119 | | 1,030,001 | 11,528 | | | | 0.88 | | | | 1.08  (0.99–1.18) | 0.076 | | | | |  | | | | | 419,475 | | | | | | | | 1,238 | | | | | | 0.23 | | | | | | | 0.97  (0.70–1.35) | | | | | | | | | 0.869 | | | | | | | |  | | 286,055 | | | | | | 2,835 | | | | | | 0.77 | | | | 1.35  (1.10–1.64) | | 0.003 | | | |  |
| 120-139 | | 1,774,926 | 26,512 | | | | 1.19 | | | | 1.27  (1.17–1.39) | <0.001 | | | | | <0.001 | | | | | 654,740 | | | | | | | | 2,959 | | | | | | 0.35 | | | | | | | 1.27  (0.91–1.75) | | | | | | | | | 0.156 | | | | | | | | <0.001 | | 460,502 | | | | | | 5,902 | | | | | | 1.00 | | | | 1.66  (1.76–2.64) | | <0.001 | | | | <0.001 |
| 140-159 | | 337,114 | 7,792 | | | | 1.89 | | | | 1.47  (1.34–1.60) | <0.001 | | | | |  | | | | | 70,354 | | | | | | | | 594 | | | | | | 0.66 | | | | | | | 1.82  (1.30–2.54) | | | | | | | | | <0.001 | | | | | | | |  | | 78,310 | | | | | | 1,381 | | | | | | 1.39 | | | | 2.16  (1.76–2.64) | | <0.001 | | | |  |
| ≥160 | | 74,310 | 2,105 | | | | 2.40 | | | | 1.63  (1.48–1.79) | <0.001 | | | | |  | | | | | 11,400 | | | | | | | | 161 | | | | | | 1.11 | | | | | | 2.64  (1.84–3.79) | | | | | | | | | | <0.001 | | | | | | | |  | | 15,490 | | | | | | 319 | | | | | | 1.65 | | | | 2.43  (1.94–3.05) | | <0.001 | | | |  |
| **Diastolic BP, mmHg** | | | | | | | | | | | | | | | | | | | | | | | | | | | | | | | | | | | | | | | | | | | | | | | | | | | | | | | | | | | | | | | | | | | | | | | | | | | | | | | | | | | | |
| <60 | | 38,038 | 464 | | | 0.99 | | | | (ref) | | | | | | | | |  | | | | 13,826 | | | | | | | | 36 | | | | | | | 0.20 | | | | | | | (ref) | | | | | | | | | | | | | |  | | 9,772 | | | | | | 61 | | | | | | | 0.49 | | | | (ref) | | | | | |  |
| 60-69 | | 390,903 | 4,596 | | | 0.94 | | | | 1.02  (0.93–1.12) | | 0.699 | | | | | | |  | | | | 152,428 | | | | | | | | 355 | | | | | | | 0.18 | | | | | | | 0.83  (0.59–1.17) | | | | | | 0.279 | | | | | | | |  | | 10,1778 | | | | | | 866 | | | | | | | 0.67 | | | | 1.34  (1.03–1.73) | | 0.029 | | | |  |
| 70-79 | | 1,148,566 | 14,974 | | | 1.04 | | | | 1.14  (1.04–1.25) | | 0.005 | | | | | | | <0.001 | | | | 455,400 | | | | | | | | 1,570 | | | | | | | 0.27 | | | | | | | 1.11  (0.80–1.54) | | | | | | 0.543 | | | | | | | | <0.001 | | 292,708 | | | | | | 3,127 | | | | | | | 0.84 | | | | 1.62  (1.26–2.09) | <0.001 | | <0.001 | | | |
| 80-89 | 1,291,556 | | 20,094 | 1.24 | | | | 1.29  (1.17–1.41) | | | | <0.001 | | |  | | | | | 458,424 | | | | 2,194 | | | | | | | | 0.37 | | | | | | | | 1.33  (0.96–1.85) | | | | | | 0.090 | | | | | | |  | | | | | | | | 340,411 | | | | 4,566 | | | | 1.05 | | | | | | 1.95  (1.51–2.51) | | | | <0.001 | |  | | | |
| ≥90 | 392,732 | | 8,328 | 1.72 | | | | 1.49  (1.36–1.64) | | | | <0.001 | | |  | | | | | 89,528 | | | | 834 | | | | | | | | 0.72 | | | | | | | | 2.01  (1.44–2.82) | | | | | | <0.001 | | | | | | |  | | | | | | | | 109,651 | | | | 1,918 | | | | 1.38 | | | | | | 2.44  (1.89–3.15) | | | | <0.001 | |  | | | |
| **Non-HDL-c, mg/dL** | | | | | | | | | | | | | | | | | | | | | | | | | | | | | | | | | | | | | | | | | | | | | | | | | | | | | | | | | | | | | | | | | | | | | | | | | | | | | | | | | | | | |
| <130 | 1,238,057 | | 12,326 | 0.80 | | | | | (ref) | | | | |  | | | | | | | 519,536 | | | | | | 1,091 | | | | | | | | 0.16 | | | | | | (ref) | | | | | | | | | | | | | | | |  | | | | 270,462 | | 1,844 | | | | | | | | | | 0.53 | | | (ref) | | | | | | |  | |
| 130-159 | 1,048,325 | | 14,711 | 1.12 | | | | | 1.32  (1.29–1.35) | | | | <0.001 |  | | | | | | | 359,018 | | | | | | 1,333 | | | | | | | | 0.29 | | | | | | 1.44  (1.33–1.56) | | | | | | | | | <0.001 | | | | | | |  | | | | 283,301 | | 2,867 | | | | | | | | | | 0.79 | | | 1.41  (1.33–1.50) | | | | <0.001 | | |  | |
| 160-189 | 651,023 | | 12,264 | | 1.50 | | | | 1.71  (1.67–1.76) | | | | <0.001 | <0.001 | | | | | | | 198,705 | | | | | | 1,335 | | | | | | | | 0.52 | | | | | | 2.26  (2.08–2.46) | | | | | | | | | <0.001 | | | | | | | <0.001 | | | | 194,925 | | 3,022 | | | | | | | | | | 1.21 | | | 2.06  (1.94–2.18) | | | | <0.001 | | | <0.001 | |
| 190-219 | 240,973 | | 6,233 | | 2.07 | | | | 2.31  (2.24–2.38) | | | | <0.001 |  | | | | | | | 69,114 | | | | | | 787 | | | | | | | | 0.88 | | | | | | 3.45  (3.13–3.80) | | | | | | | | | <0.001 | | | | | | |  | | | | 77,377 | | 1,816 | | | | | | | | | | 1.85 | | | 2.96  (2.77–3.17) | | | | <0.001 | | |  | |
| ≥220 | 83,417 | | 2,922 | | 2.82 | | | | 3.08  (2.96–3.21) | | | | <0.001 |  | | | | | | | 23,233 | | | | | | 443 | | | | | | | | 1.49 | | | | | | 5.28  (4.70–5.94) | | | | | | | | | <0.001 | | | | | | |  | | | | 28,255 | | 989 | | | | | | | | | | 2.78 | | | 4.24  (3.91–4.58) | | | | <0.001 | | |  | |
| **LDL-c, mg/dL** | | | | | | | | | | | | | | | | | | | | | | | | | | | | | | | | | | | | | | | | | | | | | | | | | | | | | | | | | | | | | | | | | | | | | | | | | | | | | | | | | | | | |
| <70 | 278,230 | | 3,319 | | 0.96 | | | | (ref) | | | | |  | | | | | | | 106,183 | | | | | | 314 | | | | | | | 0.23 | | | | | | | (ref) | | | | | | | | | | | | | | | | <0.001 | | | | 63,863 | | | 573 | | | | | | | | 0.71 | | | | (ref) | | | | |  | | | |
| 70-99 | 882,812 | | 9,561 | | 0.86 | | | | 0.99  (0.95–1.03) | | | | 0.608 |  | | | | | | | 366,243 | | | | | | 1,007 | | | | | | | 0.21 | | | | | | | 0.98  (0.86–1.11) | | | | | | | | 0.759 | | | | | | | |  |  |  |  | 205,636 | | | 1,670 | | | | | | | | 0.64 | | | | 0.98  (0.89–1.08) | | | 0.719 | |  | | | |
| 100-129 | 1,165,890 | | 15,969 | | 1.09 | | | | 1.21  (1.16–1.25) | | | | <0.001 | <0.001 | | | | | | | 420,560 | | | | | | 1,565 | | | | | | | 0.29 | | | | | | | 1.18  (1.04–1.33) | | | | | | | | 0.008 | | | | | | | |  |  |  |  | 308,916 | | | 3,307 | | | | | | | | 0.84 | | | | 1.28  (1.17–1.40) | | | <0.001 | | <0.001 | | | |
| 130-159 | 672,370 | | 12,320 | | 1.46 | | | | 1.52  (1.47–1.58) | | | | <0.001 |  | | | | | | | 206,711 | | | | | | 1,275 | | | | | | | 0.48 | | | | | | | 1.70  (1.50–1.93) | | | | | | | | <0.001 | | | | | | | |  |  |  |  | 194,553 | | | 2,910 | | | | | | | | 1.17 | | | | 1.73  (1.58–1.89) | | | <0.001 | |  | | | |
| ≥160 | 262,493 | | 7,287 | | 2.23 | | | | 2.20  (2.11–2.30) | | | | <0.001 |  | | | | | | | 69,909 | | | | | | 828 | | | | | | | 0.92 | | | | | | | 2.91  (2.56–3.32) | | | | | | | | <0.001 | | | | | | | |  |  |  |  | 81,352 | | | 2,078 | | | | | | | | 2.02 | | | | 2.81  (2.56–3.09) | | | <0.001 | |  | | | |
| **Triglycerides, mg/dL** | | | | | | | | | | | | | | | | | | | | | | | | | | | | | | | | | | | | | | | | | | | | | | | | | | | | | | | | | | | | | | | | | | | | | | | | | | | | | | | | | | | | |
| <100 | 1,128,507 | | 12,207 | | 0.86 | | | | (ref) | | | | | <0.001 | | | | | | | 459,303 | | | | | | 1,071 | | | | | | | 0.18 | | | | | | | (ref) | | | | | | | | | | | | | | | <0.001 | | | | | 251,995 | | | 1,971 | | | | | | | 0.61 | | | | | (ref) | | | | | <0.001 | | | |
| 100-149 | 919,711 | | 13,920 | | 1.21 | | | | 1.24  (1.21–1.27) | | | | <0.001 |  |  |  |  |  |  |  | 314,417 | | | | | | 1,229 | | | | | | | 0.30 | | | | | | | 1.32  (1.22–1.44) | | | | | | | <0.001 | | | | | | | |  |  |  |  |  | 237,294 | | | 2,684 | | | | | | | 0.89 | | | | | 1.30  (1.22–1.38) | | | <0.001 | |  | | | |
| 150-199 | 535,691 | | 9,348 | | 1.39 | | | | 1.40  (1.37–1.44) | | | | <0.001 |  |  |  |  |  |  |  | 174,033 | | | | | | 882 | | | | | | | 0.39 | | | | | | | 1.48  (1.35–1.62) | | | | | | | <0.001 | | | | | | | |  |  |  |  |  | 152,028 | | | 2,190 | | | | | | | 1.13 | | | | | 1.55  (1.45–1.65) | | | <0.001 | |  | | | |
| ≥200 | 677,886 | | 12,981 | | 1.52 | | | | 1.57  (1.53–1.62) | | | | <0.001 |  |  |  |  |  |  |  | 221,853 | | | | | | 1,807 | | | | | | | 0.63 | | | | | | | 2.00  (1.84–2.17) | | | | | | | <0.001 | | | | | | | |  |  |  |  |  | 213,003 | | | 3,693 | | | | | | | 1.36 | | | | | 1.75  (1.65–1.85) | | | <0.001 | |  | | | |
| **Abdominal obesity** | | | | | | | | | | | | | | | | | | | | | | | | | | | | | | | | | | | | | | | | | | | | | | | | | | | | | | | | | | | | | | | | | | | | | | | | | | | | | | | | | | | | |
| Yes | 691,155 | | 13,643 | | 1.59 | | | | 1.13  (1.11–1.16) | | | | <0.001 | - | | | | | | | 202,871 | | | | | 1,536 | | | | | | | 0.59 | | | | | | | | 1.46  (1.37–1.55) | | | | | | <0.001 | | | | | | | | - | | | | | | 179,117 | | | 2,937 | | | | | | 1.29 | | | | | | 1.23  (1.18–1.29) | | | <0.001 | | | - | | |
| **Regular physical activity** | | | | | | | | | | | | | | | | | | | | | | | | | | | | | | | | | | | | | | | | | | | | | | | | | | | | | | | | | | | | | | | | | | | | | | | | | | | | | | | | | | | | |
| Yes | 649,643 | | 10,133 | | 1.24 | | | | 1.07  (1.05–1.09) | | | | <0.001 | - | | | | | | | 185,725 | | | | 789 | | | | | | | 0.33 | | | | | | | | | 0.96  (0.89–1.04) | | | | | 0.279 | | | | | | | | - | | | | | | | 163,302 | | | 1,865 | | | | | 0.89 | | | | | | | 1.04  (0.99–1.10) | | | 0.096 | | - | | | |

|  | | | | **50-64 years old** | | | | | | | | | | | | | | | | | | | | | | | | | | | | | | **≥65 years old** | | | | | | | | | | | | | | | | | | | | | | | | |  | | | |
| --- | --- | --- | --- | --- | --- | --- | --- | --- | --- | --- | --- | --- | --- | --- | --- | --- | --- | --- | --- | --- | --- | --- | --- | --- | --- | --- | --- | --- | --- | --- | --- | --- | --- | --- | --- | --- | --- | --- | --- | --- | --- | --- | --- | --- | --- | --- | --- | --- | --- | --- | --- | --- | --- | --- | --- | --- | --- | --- | --- | --- | --- | --- |
|  | | | | **N** | | **Events** | | | | | | | **IR** | | | | | | **HR**  **(95% CI)** | | | | | | ***p*** | | ***p*_trend_** | | | | | | **N** | | | | | | **Events** | | | | | | | **IR** | | | **HR**  **(95% CI)** | | | ***p*** | ***p*_trend_** | | | | | | ***p*_interaction_** | | | |
| **BMI, kg/m2** | | | | | | | | | | | | | | | | | | | | | | | | | | | | | | | | | | | | | | | | | | | | | | | | | | | | | | | | | | | | | | |
| <18.5 | | | | 15,173 | | 289 | | | | | | | 1.63 | | | | | | 0.96  (0.85–1.08) | | | | | | 0.461 | | |  | | | | | 15,502 | | | | | | 496 | | | | | | | | 3.66 | | | 1.04  (0.95–1.14) | | 0.447 | | | |  | | | | <0.001 | | |
| 18.5-22.9 | | | | 275,189 | | 5,612 | | | | | | | 1.65 | | | | | | (ref) | | | | | | | | |  | | | | | 137,477 | | | | | | 4,958 | | | | | | | | 3.43 | | | (ref) | | | | | |  | | | |  | | |
| 23.0-24.9 | | | | 261,460 | | 6,014 | | | | | | | 1.85 | | | | | | 1.11  (1.07–1.15) | | | | | | <0.001 | | | <0.001 | | | | | 92,484 | | | | | | 3,572 | | | | | | | | 3.47 | | | 1.01  (0.96–1.05) | | 0.746 | | | | <0.001 | | | |  | | |
| 25.0-29.9 | | | | 321,096 | | 7,768 | | | | | | | 1.94 | | | | | | 1.13  (1.09–1.17) | | | | | | <0.001 | | |  | | | | | 92,896 | | | | | | 3,512 | | | | | | | | 3.34 | | | 0.95  (0.91–1.00) | | 0.041 | | | |  | | | |  | | |
| ≥30.0 | | | | 21,551 | | 544 | | | | | | | 2.04 | | | | | | 1.11  (1.01–1.21) | | | | | | 0.025 | | |  | | | | | 5,041 | | | | | | 164 | | | | | | | | 2.91 | | | 0.80  (0.68–0.93) | | 0.004 | | | |  | | | |  | | |
| **Smoking** | | | | | | | | | | | | | | | | | | | | | | | | | | | | | | | | | | | | | | | | | | | | | | | | | | | | | | | | | | | | | | |
| Non | | | | 280,077 | 5,238 | | | | | | | | | | | 1.49 | | | (ref) | | | | | | | | | <0.001 | | | | | | 147,649 | 4,893 | | | | | | | | 3.00 | | | | | (ref) | | | | | | | | <0.001 | | | | <0.001 | | |
| Ex | | | | 288,378 | 5,161 | | | | | | | | | | | 1.43 | | | 1.04  (1.00–1.08) | | 0.054 | | | | | | |  | | | | | | 107,091 | 3,682 | | | | | | | | 3.13 | | | | | 1.10  (1.05–1.15) | | | | <0.001 | | | |  | | | |  | | |
| Current | | | | 326,014 | 9,828 | | | | | | | | | | | 2.47 | | | 1.92  (1.85–1.99) | | <0.001 | | | | | | |  | | | | | | 88,660 | 4,127 | | | | | | | | 4.52 | | | | | 1.70  (1.63–1.77) | | | | <0.001 | | | |  | | | |  | | |
| **Hypertension** | | | | | | | | | | | | | | | | | | | | | | | | | | | | | | | | | | | | | | | | | | | | | | | | | | | | | | | | | | | | | | |
| Yes | | | | 306,635 | | | 7,976 | | | | | | | | | 2.11 | | | 1.20  (1.16–1.23) | | <0.001 | | | | | | | - | | | | | | 167,937 | | | | 6,700 | | | | 3.70 | | | | | | | | | 1.14  (1.10–1.18) | <0.001 | | | | - | | | | <0.001 | | |
| **Diabetes** | | | | | | | | | | | | | | | | | | | | | | | | | | | | | | | | | | | | | | | | | | | | | | | | | | | | | | | | | | | | | | |
| Normoglycemia | | | | 481,175 | | | | 9,920 | | | | | | | | 1.65 | | (ref) | | | | | | | | | | | | | <0.001 | | | 180,727 | | | | 6,143 | | | 3.11 | | | | | | | | | (ref) | | | | | |  | | | | <0.001 | | |
| Prediabetes | | | | 276,911 | | | 5,800 | | | | | | | | | 1.68 | | 1.02  (0.99–1.06) | | | | | 0.217 | | | | | | | |  | | | 98,921 | | | | 3,520 | | | 3.26 | | | | | | | | | 1.07  (1.03–1.11) | | 0.002 | | | | <0.001 | | | |  | | |
| Diabetes | | | | 136,383 | | | 4,507 | | | | | | | | | 2.73 | | 1.50  (1.44–1.55) | | | | | <0.001 | | | | | | | |  | | | 63,752 | | | 3,039 | | | | 4.60 | | | | | | | | | 1.47  (1.41–1.54) | | <0.001 | | | |  | | | |  | | |
| **Systolic BP, mmHg** | | | | | | | | | | | | | | | | | | | | | | | | | | | | | | | | | | | | | | | | | | | | | | | | | | | | | | | | | | | | | | |
| <100 | | | | 13,762 | | | | | 239 | | | | | | | | 1.41 | | (ref) | | | | | | | | | | | |  | | | 4,082 | | 142 | | | | 3.44 | | | | | | | | | (ref) | | | | | | |  | | | | <0.001 | | |
| 100-119 | | | | 249,867 | | | | | 4,989 | | | | | | | | 1.60 | | 1.17  (1.03–1.33) | | | | | 0.018 | | | | | | |  | | | 74,604 | | 2,466 | | | | 3.06 | | | | | | | | | 0.92  (0.78–1.09) | | | 0.335 | | | |  | | | |  | | |
| 120-139 | | | | 480,542 | | | | | 11,033 | | | | | | | | 1.85 | | 1.34  (1.18–1.53) | | | | | <0.001 | | | | | | | <0.001 | | | 179,142 | | 6,618 | | | | 3.38 | | | | | | | | | 1.03  (0.87–1.22) | | | 0.734 | | | | <0.001 | | | |  | | |
| 140-159 | | | | 122,277 | | | | | 3,173 | | | | | | | | 2.11 | | 1.51  (1.32–1.72) | | | | | <0.001 | | | | | | |  | | | 66,173 | | 2,644 | | | | 3.69 | | | | | | | | | 1.12  (0.95–1.33) | | | 0.181 | | | |  | | | |  | | |
| ≥160 | | | | 28,021 | | | | | 793 | | | | | | | | 2.34 | | 1.65  (1.43–1.91) | | | | | <0.001 | | | | | |  | | | | 19,399 | | 832 | | | | 4.13 | | | | | | | | | 1.24  (1.04–1.48) | | | 0.019 | | | |  | | | |  | | |
| **Diastolic BP, mmHg** | | | | | | | | | | | | | | | | | | | | | | | | | | | | | | | | | | | | | | | | | | | | | | | | | | | | | | | | | | | | | | |
| <60 | 9,430 | | | | | | | | | 190 | | | | | | 1.63 | | | | (ref) | | | | | | | | | | | |  | | 5,010 | | | | 177 | | | | | | 3.47 | | | | (ref) | | | | | | | |  | | | | <0.001 | | |
| 60-69 | 94,511 | | | | | | | | | 1,849 | | | | | | 1.58 | | | | 0.98  (0.84–1.14) | | | | | | 0.763 | | | | | |  | | 42,186 | | | | 1,526 | | | | | | 3.38 | | | | 1.03  (0.88–1.20) | | | | 0.727 | | | |  | | | |  | | |
| 70-79 | | 287,757 | | | | | | | | | | 6,244 | | | 1.74 | | | | | 1.10  (0.95–1.27) | | | | | | 0.193 | | | | | | <0.001 | | 112,701 | | | | 4,033 | | | | | | | 3.30 | | | 1.04  (0.90–1.21) | | | | 0.593 | | | | <0.001 | | | |  | | |
| 80-89 | | 362,238 | | | | | | | | | | 8,412 | | | 1.87 | | | | | 1.19  (1.03–1.38) | | | | | | 0.017 | | | | | |  | | 130,483 | | | | 4,922 | | | | | | | 3.46 | | | 1.12  (0.96–1.30) | | | | 0.156 | | | |  | | | |  | | |
| ≥90 | | 140,533 | | | | | | | | | | 3,532 | | | 2.04 | | | | | 1.31  (1.13–1.52) | | | | | | <0.001 | | | | | |  | | 53,020 | | | | 2,044 | | | | | | | 3.58 | | | 1.17  (1.00–1.36) | | | | 0.048 | | | |  | | | |  | | |
| **Non-HDL-c, mg/dL** | | | | | | | | | | | | | | | | | | | | | | | | | | | | | | | | | | | | | | | | | | | | | | | | | | | | | | | | | | | | | | |
| <130 | | | 302,632 | | | | | | | | 4,964 | | | 1.33 | | | | (ref) | | | | | | | | | | |  | | | | | 145,427 | | | 4,427 | | | | | | | 2.87 | | | | (ref) | | | | | |  | | | <0.001 | | | | |  |
| 130-159 | | | 296,185 | | | | | | | | 6,337 | | | 1.71 | | | | 1.30  (1.26–1.35) | | | | <0.001 | | | | | | |  | | | | | 6,457 | | | 4,174 | | | | | | | 3.46 | | | | 1.22  (1.17–1.27) | | | | <0.001 | |  | | |  | | | | |  |
| 160-189 | | | 195,842 | | | | | | | | 5,242 | | | 2.15 | | | | 1.60  (1.54–1.67) | | | | <0.001 | | | | | | | <0.001 | | | | | 3,773 | | | 2,665 | | | | | | | 3.92 | | | | 1.37  (1.31–1.44) | | | | <0.001 | | <0.001 | | |  | | | | |  |
| 190-219 | | | 74,309 | | | | | | | | 2,593 | | | 2.82 | | | | 2.05  (1.95–2.15) | | | | <0.001 | | | | | | |  | | | | | 1,385 | | | 1,037 | | | | | | | 4.69 | | | | 1.61  (1.50–1.72) | | | | <0.001 | |  | | |  | | | | |  |
| ≥220 | | | 25,501 | | | | | | | | 1,091 | | | 3.49 | | | | 2.45  (2.29–2.61) | | | | <0.001 | | | | | | |  | | | | | 481 | | | 399 | | | | | | | 5.84 | | | | 1.95  (1.76–2.16) | | | | <0.001 | |  | | |  | | | | |  |
| **LDL-c, mg/dL** | | | | | | | | | | | | | | | | | | | | | | | | | | | | | | | | | | | | | | | | | | | | | | | | | | | | | | | | | | | | | | |
| <70 | | | 74,547 | | | | | | | | 1,390 | | | 1.53 | | | | (ref) | | | | | | | | | | |  | | | | | 33,637 | | | 1,042 | | | | | | | 3.00 | | | | (ref) | | | | | |  | | | <0.001 | | | | |  |
| 70-99 | | | 216,649 | | | | | | | | 3,864 | | | 1.44 | | | | 1.00  (0.94–1.06) | | | | 0.896 | | | | | | |  | | | | | 94,284 | | | 3,020 | | | | | | | 2.99 | | | | 1.01  (0.94–1.09) | | | | 0.726 | |  | | |  | | | | |  |
| 100-129 | | | 315,530 | | | | | | | | 6,678 | | | 1.70 | | | | 1.20  (1.14–1.28) | | | | <0.001 | | | | | | | <0.001 | | | | | 120,884 | | | 4,419 | | | | | | | 3.34 | | | | 1.15  (1.07–1.23) | | | | <0.001 | | <0.001 | | |  | | | | |  |
| 130-159 | | | 202,483 | | | | | | | | 5,261 | | | 2.08 | | | | 1.47  (1.38–1.56) | | | | <0.001 | | | | | | |  | | | | | 68,623 | | | 2,874 | | | | | | | 3.80 | | | | 1.30  (1.21–1.40) | | | | <0.001 | |  | | |  | | | | |  |
| ≥160 | | | 85,260 | | | | | | | | 3,034 | | | 2.88 | | | | 1.98  (1.85–2.11) | | | | <0.001 | | | | | | |  | | | | | 25,972 | | | 1,347 | | | | | | | 4.75 | | | | 1.60  (1.48–1.74) | | | | <0.001 | |  | | |  | | | | |  |
| **Triglycerides, mg/dL** | | | | | | | | | | | | | | | | | | | | | | | | | | | | | | | | | | | | | | | | | | | | | | | | | | | | | | | | | | | | | | |
| <100 | | | 283,754 | | | | | | | | 5,002 | | | 1.42 | | | | (ref) | | | | | | | | | | | <0.001 | | | | | 133,455 | | | 4,163 | | | | | | | 2.90 | | | | (ref) | | | | | | <0.001 | | | <0.001 | | | | |  |
| 100-149 | | | 261,520 | | | | | | | | 5,921 | | | 1.82 | | | | 1.21  (1.17–1.26) | | | | <0.001 | | | | | | |  |  |  |  |  | 106,480 | | | 4,086 | | | | | | | 3.55 | | | | 1.19  (1.14–1.24) | | | | <0.001 | |  |  |  |  | | | | |  |
| 150-199 | | | 156,514 | | | | | | | | 3,990 | | | 2.05 | | | | 1.33  (1.27–1.39) | | | | <0.001 | | | | | | |  |  |  |  |  | 53,116 | | | 2,286 | | | | | | | 3.94 | | | | 1.31  (1.24–1.38) | | | | <0.001 | |  |  |  |  | | | | |  |
| ≥200 | | | 192,681 | | | | | | | | 5,314 | | | 2.22 | | | | 1.41  (1.35–1.47) | | | | <0.001 | | | | | | |  |  |  |  |  | 50,349 | | | 2,167 | | | | | | | 3.93 | | | | 1.31  (1.24–1.38) | | | | <0.001 | |  |  |  |  | | | | |  |
| **Abdominal obesity** | | | | | | | | | | | | | | | | | | | | | | | | | | | | | | | | | | | | | | | | | | | | | | | | | | | | | | | | | | | | | | |
| Yes | | | 220,610 | | | | | | | | | 5,633 | | | 2.06 | | | 1.07  (1.04–1.11) | | | | <0.001 | | | | | | | - | | | | | 88,557 | | | 3,537 | | | | | | | 3.62 | | | | 1.02  (0.98–1.06) | | | | 0.349 | | | - | | | <0.001 | | |  |  |
| **Regular physical activity** | | | | | | | | | | | | | | | | | | | | | | | | | | | | | | | | | | | | | | | | | | | | | | | | | | | | | | | | | | | | | | |
| Yes | | | 210,912 | | | | | | | | | 4,435 | | | 1.69 | | | 1.07  (1.04–1.10) | | | | <0.001 | | | | | | | - | | | | | 89,704 | | | 3,044 | | | | | | | 3.01 | | | | 1.12  (1.08–1.17) | | | | <0.001 | | - | | | <0.001 | | | |  |  |

1. **Women**

|  | **Total** | | | | | | | | | | | | | | | | | | **<40 years old** | | | | | | | | | | | | | | | | | | | | | | | | | | | | | | | | **40-49 years old** | | | | | | | | | | | | | | | | | | | | | | | | | |
| --- | --- | --- | --- | --- | --- | --- | --- | --- | --- | --- | --- | --- | --- | --- | --- | --- | --- | --- | --- | --- | --- | --- | --- | --- | --- | --- | --- | --- | --- | --- | --- | --- | --- | --- | --- | --- | --- | --- | --- | --- | --- | --- | --- | --- | --- | --- | --- | --- | --- | --- | --- | --- | --- | --- | --- | --- | --- | --- | --- | --- | --- | --- | --- | --- | --- | --- | --- | --- | --- | --- | --- | --- | --- | --- | --- | --- |
|  | **N** | | **Events** | | | | **IR** | | **HR**  **(95% CI)** | ***p*** | | | | | | ***p*_trend_** | | | **N** | **Events** | | | | | | | | | | | | **IR** | | | | **HR**  **(95% CI)** | | | | | | | | ***p*** | | | | | ***p*_trend_** | | **N** | | | | **Events** | | | | **IR** | | | | | | **HR**  **(95% CI)** | | | | | ***p*** | | | | ***p*_trend_** | | |
| **BMI, kg/m2** | | | | | | | | | | | | | | | | | | | | | | | | | | | | | | | | | | | | | | | | | | | | | | | | | | | | | | | | | | | | | | | | | | | | | | | | | | | | |
| <18.5 | 166,545 | | 594 | | | | 0.28 | | 1.07  (0.98–1.16) | 0.120 | | | | | |  | | | 107,261 | 85 | | | | | | | | | | | | 0.06 | | | | 1.08  (0.85–1.37) | | | | | | | | 0.513 | | | |  | | | 29,043 | | | | 61 | | | | 0.16 | | | | | | 0.97  (0.75–1.26) | | | | | 0.830 | | | |  | | |
| 18.5-22.9 | | 1,399,376 | 6,989 | | | | 0.39 | | (ref) | | |  | | | | | | | 456,301 | 370 | | | | | | | | | | | | 0.06 | | | | (ref) | | | | | | | | | | | |  | | | 442,392 | | | | 1,031 | | | | 0.18 | | | | | | (ref) | | | | | | | | |  | | |
| 23.0-24.9 | | 648,266 | 5,119 | | | | 0.62 | | 1.03  (0.99–1.06) | 0.174 | | | | <0.001 | | | | | 85,658 | 110 | | | | | | | | | | | | 0.10 | | | | 1.42  (1.14–1.76) | | | | | | | | 0.001 | | | | <0.001 | | | 193,275 | | | | 548 | | | | 0.22 | | | | | | 1.11  (1.00–1.23) | | | | | 0.050 | | | | <0.001 | | |
| 25.0-29.9 | | 677,902 | 6,724 | | | | 0.78 | | 1.06  (1.02–1.10) | 0.001 | | | |  | | | | | 67,924 | 81 | | | | | | | | | | | | 0.09 | | | | 1.24  (0.97–1.59) | | | | | | | | 0.086 | | | |  | | | 172,950 | | | | 593 | | | | 0.27 | | | | | | 1.23  (1.10–1.36) | | | | | <0.001 | | | |  | | |
| ≥30.0 | | 95,968 | 973 | | | | 0.80 | | 1.11  (1.04–1.19) | 0.002 | | | |  | | | | | 15,653 | 39 | | | | | | | | | | | | 0.19 | | | | 2.44  (1.72–3.46) | | | | | | | | <0.001 | | | |  | | | 25,867 | | | | 110 | | | | 0.33 | | | | | | 1.33  (1.08–1.62) | | | | | 0.007 | | | |  | | |
| **Smoking** | | | | | | | | | | | | | | | | | | | | | | | | | | | | | | | | | | | | | | | | | | | | | | | | | | | | | | | | | | | | | | | | | | | | | | | | | | | | |
| Non | | 2,826,772 | 19,030 | | | | 0.53 | | (ref) | | | |  | | | | | | 671,770 | 607 | | | | | | | | | | | | 0.07 | | | | (ref) | | | | | | | | | | | |  | | | 816,789 | | | | 2,114 | | | | 0.20 | | | | | | (ref) | | | | | | | | |  | | |
| Ex | | 55,264 | 317 | | | | 0.45 | | 1.31  (1.17–1.46) | <0.001 | | | | <0.001 | | | | | 237,94 | 25 | | | | | | | | | | | | 0.08 | | | | 1.22  (0.82–1.83) | | | | | | | 0.333 | | | | | <0.001 | | | 15,479 | | | | 66 | | | | 0.33 | | | | | | 1.74  (1.36–2.22) | | | | | <0.001 | | | | <0.001 | | |
| Current | | 106,021 | 1,052 | | | | 0.79 | | 2.04  (1.91–2.17) | <0.001 | | | |  | | | | | 372,33 | 53 | | | | | | | | | | | | 0.11 | | | | 1.66  (1.24–2.23) | | | | | | | 0.001 | | | | |  | | | 31,259 | | | | 163 | | | | 0.41 | | | | | | 1.98  (1.68–2.34) | | | | | <0.001 | | | |  | | |
| **Hypertension** | | | | | | | | | | | | | | | | | | | | | | | | | | | | | | | | | | | | | | | | | | | | | | | | | | | | | | | | | | | | | | | | | | | | | | | | | | | | |
| Yes | | 614,190 | 9,544 | | | | 1.25 | | 1.27  (1.23–1.31) | <0.001 | | | | | - | | | | 16,487 | | | | | | 31 | | | | | | | 0.15 | | | | 1.36  (0.93–1.98) | | | | | | | 0.109 | | | | | - | | | 92,169 | | | | 426 | | | | 0.36 | | | | | | 1.49  (1.33–1.66) | | | | | <0.001 | | | | - | | |
| **Diabetes** | | | | | | | | | | | | | | | | | | | | | | | | | | | | | | | | | | | | | | | | | | | | | | | | | | | | | | | | | | | | | | | | | | | | | | | | | | | | |
| Normo-glycemia | | 2,237,640 | 11,732 | | | | 0.41 | | (ref) | | | | | |  | | | | 651,621 | | | | | | | 593 | | | | | | 0.07 | | | | (ref) | | | | | | | | | | | |  | | | 678,705 | | | | 1,716 | | | | 0.20 | | | | | | (ref) | | | | | | | | |  | | |
| Prediabetes | | 562,623 | 4,545 | | | | 0.64 | | 1.02  (0.99–1.06) | 0.182 | | | | | <0.001 | | | | 74,195 | | | | | | 76 | | | | | | | 0.08 | | | | 0.95  (0.74–1.21) | | | | | | | 0.661 | | | | | <0.001 | | | 157,013 | | | | 435 | | | | 0.22 | | | | | | 0.98  (0.88–1.09) | | | | | 0.736 | | | | <0.001 | | |
| Diabetes | | 187,794 | 4,122 | | | | 1.80 | | 1.80  (1.74–1.87) | <0.001 | | | | |  | | | | 6,981 | | | | | | 16 | | | | | | | 0.18 | | | | 1.60  (0.96–2.66) | | | | | | | 0.071 | | | | |  | | | 27,809 | | | | 192 | | | | 0.54 | | | | | | 2.06  (1.76–2.40) | | | | | <0.001 | | | |  | | |
| **Systolic BP, mmHg** | | | | | | | | | | | | | | | | | | | | | | | | | | | | | | | | | | | | | | | | | | | | | | | | | | | | | | | | | | | | | | | | | | | | | | | | | | | | |
| <100 | | 175,958 | 449 | | | | 0.20 | | (ref) | | | | | |  | | | | 74,455 | | | | | | | 55 | | | | | | 0.06 | | | | (ref) | | | | | | | | | | | |  | | | 60,047 | | | | 123 | | | | 0.16 | | | | | | (ref) | | | | | | | | |  | | |
| 100-119 | | 1,341,005 | 5,527 | | | | 0.32 | | 1.15  (1.05–1.27) | 0.004 | | | | |  | | | | 460,257 | | | | | | | 398 | | | | | | 0.07 | | | | 1.13  (0.85–1.50) | | | | | | | 0.398 | | | | |  | | | 435,333 | | | | 1,039 | | | | 0.19 | | | | | | 1.12  (0.93–1.35) | | | | | 0.252 | | | |  | | |
| 120-139 | | 1,184,601 | 9,948 | | | | 0.66 | | 1.31  (1.19–1.44) | <0.001 | | | | | <0.001 | | | | 189,626 | | | | | | | 209 | | | | | | 0.09 | | | | 1.28  (0.95–1.73) | | | | | | | 0.111 | | | | | 0.004 | | | 320,210 | | | | 968 | | | | 0.24 | | | | | | 1.28  (1.06–1.55) | | | | | 0.010 | | | | <0.001 | | |
| 140-159 | | 230,960 | 3,371 | | | | 1.18 | | 1.45  (1.31–1.61) | <0.001 | | | | |  | | | | 7,185 | | | | | | | 18 | | | | | | 0.20 | | | | 2.10  (1.21–3.64) | | | | | | | 0.008 | | | | |  | | | 39,805 | | | | 170 | | | | 0.33 | | | | | | 1.60  (1.26–2.03) | | | | | <0.001 | | | |  | | |
| ≥160 | | 55,533 | 1,104 | | | | 1.63 | | 1.70  (1.52–1.91) | <0.001 | | | | |  | | | | 1,274 | | | | | | | 5 | | | | | | 0.31 | | | | 2.88  (1.14–7.31) | | | | | | | 0.026 | | | | |  | | | 8,132 | | | | 43 | | | | 0.41 | | | | | | 1.90  (1.34–2.71) | | | | | <0.001 | | | |  | | |
| **Diastolic BP, mmHg** | | | | | | | | | | | | | | | | | | | | | | | | | | | | | | | | | | | | | | | | | | | | | | | | | | | | | | | | | | | | | | | | | | | | | | | | | | | | |
| <60 | | 112,872 | 368 | | | 0.26 | | | (ref) | | | | | | | |  | | 43,083 | | | | | | | 29 | | | | | | 0.05 | | | | (ref) | | | | | | | | | | | |  | | | 9,772 | | | | | 80 | | | | | | | 0.16 | | | | | | (ref) | | | | | |  | |
| 60-69 | | 716,136 | 2,986 | | | 0.33 | | | 1.08  (0.97–1.20) | 0.169 | | | | | | |  | | 248,118 | | | | | | | 204 | | | | | | 0.06 | | | | 1.20  (0.82–1.78) | | | | | | | 0.353 | | | | |  | | | 10,1778 | | | | | 545 | | | | | | | 0.18 | | | | | | 1.11  (0.88–1.40) | | | | 0.384 | |  | |
| 70-79 | | 1,119,967 | 6,832 | | | 0.48 | | | 1.18  (1.06–1.31) | 0.002 | | | | | | | <0.001 | | 300,421 | | | | | | | 274 | | | | | | 0.07 | | | | 1.27  (0.87–1.87) | | | | | | | 0.219 | | | | | 0.008 | | | 292,708 | | | | | 824 | | | | | | | 0.20 | | | | | | 1.13  (0.90–1.42) | | | | 0.299 | | <0.001 | |
| 80-89 | | 811,839 | 7,245 | 0.71 | | | | | 1.28  (1.15–1.41) | <0.001 | | | | | | |  | | 129,439 | | | | | | | 159 | | | | | | 0.10 | | | | 1.54  (1.03–2.30) | | | | | | | 0.034 | | | | |  | | | 340,411 | | | | | 670 | | | | | | | | 0.25 | | | | | 1.31  (1.04–1.65) | | | | 0.024 | |  | |
| ≥90 | | 227,243 | 2,968 | 1.05 | | | | | 1.43  (1.28–1.59) | <0.001 | | | | | | |  | | 11,736 | | | | | | | 19 | | | | | | 0.13 | | | | 1.54  (0.85–2.79) | | | | | | | 0.151 | | | | |  | | | 109,651 | | | | | 224 | | | | | | | | 0.35 | | | | | 1.70  (1.31–2.20) | | | | <0.001 | |  | |
| **Non-HDL-c, mg/dL** | | | | | | | | | | | | | | | | | | | | | | | | | | | | | | | | | | | | | | | | | | | | | | | | | | | | | | | | | | | | | | | | | | | | | | | | | | | | |
| <130 | 1,351,248 | | 5,620 | 0.33 | | | | (ref) | | | | |  | | | | | | 522,449 | | | | | 448 | | | | | | | 0.07 | | | | (ref) | | | | | | | | | | | |  | | | | 430,262 | 1,023 | | | | | | 0.19 | | | | | | | | | | (ref) | | | | | | | |  |
| 130-159 | 864,776 | | 5,935 | 0.54 | | | | 1.09  (1.05–1.13) | | <0.001 | | |  | | | | | | 151,944 | | | | | 144 | | | | | | | 0.07 | | | | 0.95  (0.79–1.15) | | | | | | | 0.605 | | | | |  | | | | 266,540 | 742 | | | | | | 0.22 | | | | | | | | | | 1.08  (0.98–1.19) | | | | 0.102 | | | |  |
| 160-189 | 499,740 | | 4,999 | | 0.79 | | | 1.27  (1.22–1.32) | | <0.001 | | | <0.001 | | | | | | 44,082 | | | | 69 | | | | | | | 0.12 | | | | 1.39  (1.07–1.81) | | | | | | | 0.013 | | | | 0.045 | | | | | | 119,919 | | 355 | | | | 0.23 | | | | | | | | | 1.06  (0.94–1.20) | | | | | 0.326 | | | | <0.001 | |
| 190-219 | 195,798 | | 2,536 | | 1.03 | | | 1.45  (1.38–1.52) | | <0.001 | | |  | | | | | | 10,652 | | | | 17 | | | | | | | 0.12 | | | | 1.28  (0.78–2.10) | | | | | | | 0.333 | | | |  | | | | | | 35,573 | | 150 | | | | 0.33 | | | | | | | | | 1.40  (1.18–1.67) | | | | | <0.001 | | | |  | |
| ≥220 | 76,495 | | 1,309 | | 1.37 | | | 1.78  (1.67–1.89) | | <0.001 | | |  | | | | | | 3,670 | | | | 7 | | | | | | | 0.15 | | | | 1.49  (0.70–3.18) | | | | | | | 0.298 | | | |  | | | | | | 11,233 | | 73 | | | | 0.51 | | | | | | | | | 1.97  (1.55–2.51) | | | | | <0.001 | | | |  | |
| **LDL-c, mg/dL** | | | | | | | | | | | | | | | | | | | | | | | | | | | | | | | | | | | | | | | | | | | | | | | | | | | | | | | | | | | | | | | | | | | | | | | | | | | | |
| <70 | 197,068 | | 1,051 | | 0.42 | | | (ref) | | | | |  | | | | | 84,138 | | | | 90 | | | | | | | 0.08 | | | | (ref) | | | | | | | | | | | | | | 0.546 | | | 52,888 | | | | 164 | | | | | | | | 0.24 | | | | | (ref) | | | | | | |  | | |
| 70-99 | 820,432 | | 3,922 | | 0.38 | | | 0.97  (0.91–1.04) | | 0.385 | | |  | | | | | 308,718 | | | | 246 | | | | | | | 0.06 | | | | 0.72  (0.57–0.92) | | | | | | | 0.009 | | | | | | |  |  |  | 252,255 | | | | 619 | | | | | | | | 0.19 | | | | | 0.84  (0.71–1.00) | | | 0.044 | | | |  | | |
| 100-129 | 1,046,503 | | 6,357 | | 0.48 | | | 1.00  (0.94–1.07) | | 0.977 | | | <0.001 | | | | | 245,916 | | | | 225 | | | | | | | 0.07 | | | | 0.76  (0.60–0.98) | | | | | | | 0.031 | | | | | | |  |  |  | 333,502 | | | | 844 | | | | | | | | 0.20 | | | | | 0.84  (0.71–0.99) | | | 0.037 | | | | 0.024 | | |
| 130-159 | 622,225 | | 5,386 | | 0.68 | | | 1.13  (1.05–1.20) | | 0.001 | | |  | | | | | 75,250 | | | | 97 | | | | | | | 0.10 | | | | 0.96  (0.72–1.29) | | | | | | | 0.787 | | | | | | |  |  |  | 166,520 | | | | 475 | | | | | | | | 0.22 | | | | | 0.88  (0.74–1.06) | | | 0.169 | | | |  | | |
| ≥160 | 301,829 | | 3,683 | | 0.97 | | | 1.35  (1.26–1.45) | | <0.001 | | |  | | | | | 18,775 | | | | 27 | | | | | | | 0.11 | | | | 0.97  (0.63–1.50) | | | | | | | 0.891 | | | | | | |  |  |  | 58,362 | | | | 241 | | | | | | | | 0.32 | | | | | 1.18  (0.96–1.44) | | | 0.113 | | | |  | | |
| **Triglycerides, mg/dL** | | | | | | | | | | | | | | | | | | | | | | | | | | | | | | | | | | | | | | | | | | | | | | | | | | | | | | | | | | | | | | | | | | | | | | | | | | | | |
| <100 | 1,676,753 | | 6,849 | | 0.32 | | | (ref) | | | <0.001 | | | | | | | 566,268 | | | | 467 | | | | | | | 0.06 | | | | (ref) | | | | | | | | | | | | | 0.002 | | | | 541,019 | | | | 1,255 | | | | | | | 0.18 | | | | | | (ref) | | | | | | | <0.001 | | |
| 100-149 | 749,814 | | 6,345 | | 0.67 | | | 1.17  (1.13–1.22) | | <0.001 |  |  |  |  |  |  |  | 114,564 | | | | 137 | | | | | | | 0.09 | | | | 1.24  (1.02–1.51) | | | | | | 0.031 | | | | | | |  |  |  |  | 201,875 | | | | 580 | | | | | | | 0.22 | | | | | | 1.10  (1.00–1.22) | | | 0.060 | | | |  |  |  |
| 150-199 | 309,293 | | 3,496 | | 0.90 | | | 1.28  (1.22–1.33) | | <0.001 |  |  |  |  |  |  |  | 31,110 | | | | 41 | | | | | | | 0.10 | | | | 1.22  (0.88–1.69) | | | | | | 0.237 | | | | | | |  |  |  |  | 69,609 | | | | 262 | | | | | | | 0.29 | | | | | | 1.32  (1.15–1.51) | | | <0.001 | | | |  |  |  |
| ≥200 | 252,197 | | 3,709 | | 1.17 | | | 1.51  (1.44–1.57) | | <0.001 |  |  |  |  |  |  |  | 20,855 | | | | 40 | | | | | | | 0.15 | | | | 1.61  (1.14–2.26) | | | | | | 0.006 | | | | | | |  |  |  |  | 51,024 | | | | 246 | | | | | | | 0.38 | | | | | | 1.52  (1.32–1.76) | | | <0.001 | | | |  |  |  |
| **Abdominal obesity** | | | | | | | | | | | | | | | | | | | | | | | | | | | | | | | | | | | | | | | | | | | | | | | | | | | | | | | | | | | | | | | | | | | | | | | | | | | | |
| Yes | 485,130 | | 6,307 | | 1.04 | | | 1.09  (1.06–1.12) | | <0.001 | - | | | | | | | 43,127 | | | 73 | | | | | | | 0.13 | | | | | 1.52  (1.18–1.96) | | | | | 0.001 | | | | | | | - | | | | | 93,244 | | | | 365 | | | | | | 0.31 | | | | | | | 1.21  (1.08–1.36) | | | 0.001 | | | | | - | |
| **Regular physical activity** | | | | | | | | | | | | | | | | | | | | | | | | | | | | | | | | | | | | | | | | | | | | | | | | | | | | | | | | | | | | | | | | | | | | | | | | | | | | |
| Yes | 471,637 | | 3,159 | | 0.53 | | | 1.09  (1.05–1.14) | | <0.001 | - | | | | | | | 68,411 | | 66 | | | | | | | 0.07 | | | | | | 1.02  (0.79–1.32) | | | | 0.871 | | | | | | | | - | | | | | 141,448 | | | | 371 | | | | | 0.20 | | | | | | | | 1.06  (0.95–1.19) | | | 0.288 | | | | - | | |

|  | | | | **50-64 years old** | | | | | | | | | | | | | | | | | | | | | | | | | | | | | | **≥65 years old** | | | | | | | | | | | | | | | | | | | | | | | | | |  | | |
| --- | --- | --- | --- | --- | --- | --- | --- | --- | --- | --- | --- | --- | --- | --- | --- | --- | --- | --- | --- | --- | --- | --- | --- | --- | --- | --- | --- | --- | --- | --- | --- | --- | --- | --- | --- | --- | --- | --- | --- | --- | --- | --- | --- | --- | --- | --- | --- | --- | --- | --- | --- | --- | --- | --- | --- | --- | --- | --- | --- | --- | --- | --- |
|  | | | | **N** | | **Events** | | | | | | | **IR** | | | | | | **HR**  **(95% CI)** | | | | | | | ***p*** | ***p*_trend_** | | | | | | **N** | | | | | | **Events** | | | | | | | | **IR** | | | **HR**  **(95% CI)** | | | ***p*** | ***p*_trend_** | | | | | | ***p*_interaction_** | | |
| **BMI, kg/m2** | | | | | | | | | | | | | | | | | | | | | | | | | | | | | | | | | | | | | | | | | | | | | | | | | | | | | | | | | | | | | | |
| <18.5 | | | | 16,895 | | 103 | | | | | | | 0.48 | | | | | | 1.07  (0.87–1.30) | | | | | | | 0.534 | |  | | | | | 13,346 | | | | | | 345 | | | | | | | | | 2.51 | | | 1.06  (0.94–1.18) | | 0.347 | | | |  | <0.001 | | | | |
| 18.5-22.9 | | | | 371,542 | | 2,233 | | | | | | | 0.47 | | | | | | (ref) | | | | | | | | |  | | | | | 129,141 | | | | | | 3,355 | | | | | | | | | 2.23 | | | (ref) | |  | | | |  |  | | | | |
| 23.0-24.9 | | | | 269,396 | | 1,974 | | | | | | | 0.57 | | | | | | 1.09  (1.02–1.16) | | | | | | | 0.008 | | <0.001 | | | | | 99,937 | | | | | | 2,487 | | | | | | | | | 2.07 | | | 0.94  (0.89–0.99) | | 0.012 | | | | <0.001 |  | | | | |
| 25.0-29.9 | | | | 303,745 | | 2,713 | | | | | | | 0.70 | | | | | | 1.18  (1.11–1.25) | | | | | | | <0.001 | |  | | | | | 133,283 | | | | | | 3,337 | | | | | | | | | 2.07 | | | 0.92  (0.87–0.96) | | <0.001 | | | |  |  | | | | |
| ≥30.0 | | | | 38,253 | | 409 | | | | | | | 0.84 | | | | | | 1.23  (1.10–1.37) | | | | | | | <0.001 | |  | | | | | 16,195 | | | | | | 415 | | | | | | | | | 2.14 | | | 0.90  (0.81–1.00) | | 0.042 | | | |  |  | | | | |
| **Smoking** | | | | | | | | | | | | | | | | | | | | | | | | | | | | | | | | | | | | | | | | | | | | | | | | | | | | | | | | | | | | | | |
| Non | | | | 960,054 | 6,934 | | | | | | | | | | | 0.57 | | | (ref) | | | | | | | | |  | | | | | | 378,159 | 9,375 | | | | | | | | 2.09 | | | | | | (ref) | | | | | | | |  | 0.029 | | | | |
| Ex | | | | 11,883 | 98 | | | | | | | | | | | 0.65 | | | 1.25  (1.02–1.52) | | 0.031 | | | | | | | <0.001 | | | | | | 4,108 | 128 | | | | | | | | 2.78 | | | | | | 1.22  (1.02–1.45) | | | | 0.030 | | | | <0.001 |  | | | | |
| Current | | | | 27,894 | 400 | | | | | | | | | | | 1.14 | | | 2.21  (2.00–2.46) | | <0.001 | | | | | | |  | | | | | | 9,635 | 436 | | | | | | | | 4.21 | | | | | | 1.92  (1.74–2.11) | | | | <0.001 | | | |  |  | | | | |
| **Hypertension** | | | | | | | | | | | | | | | | | | | | | | | | | | | | | | | | | | | | | | | | | | | | | | | | | | | | | | | | | | | | | | |
| Yes | | | | 293,681 | | | 3,120 | | | | | | | | | 0.84 | | | 1.36  (1.29–1.43) | | <0.001 | | | | | | | - | | | | | | 211,853 | | | | 5,967 | | | | 2.40 | | | | | | | | | | 1.17  (1.12–1.22) | <0.001 | | | | - | <0.001 | | | | |
| **Diabetes** | | | | | | | | | | | | | | | | | | | | | | | | | | | | | | | | | | | | | | | | | | | | | | | | | | | | | | | | | | | | | | |
| Normoglycemia | | | | 680,305 | | | | 4,313 | | | | | | | | 0.50 | | (ref) | | | | | | | | | | | | |  | | | 227,009 | | | | 5,110 | | | 1.88 | | | | | | | | | | (ref) | | | | | |  | <0.001 | | | | |
| Prediabetes | | | | 232,129 | | | 1,678 | | | | | | | | | 0.57 | | 1.02  (0.97–1.08) | | | | | 0.440 | | | | | | | | <0.001 | | | 99,286 | | | | 2,356 | | | 2.00 | | | | | | | | | | 1.03  (0.98–1.08) | | 0.278 | | | | <0.001 |  | | | | |
| Diabetes | | | | 87,397 | | | 1,441 | | | | | | | | | 1.31 | | 1.99  (1.87–2.12) | | | | | <0.001 | | | | | | | |  | | | 65,607 | | | 2,473 | | | | 3.31 | | | | | | | | | | 1.67  (1.59–1.75) | | <0.001 | | | |  |  | | | | |
| **Systolic BP, mmHg** | | | | | | | | | | | | | | | | | | | | | | | | | | | | | | | | | | | | | | | | | | | | | | | | | | | | | | | | | | | | | | |
| <100 | | | | 36,711 | | | | | 174 | | | | | | | | 0.37 | | (ref) | | | | | | | | | | | |  | | | 4,745 | | 97 | | | | 1.73 | | | | | | | | | | (ref) | | | | | | |  | <0.001 | | | | |
| 100-119 | | | | 360,007 | | | | | 2,199 | | | | | | | | 0.48 | | 1.17  (1.00–1.37) | | | | | 0.045 | | | | | | |  | | | 85,408 | | 1,891 | | | | 1.86 | | | | | | | | | | 1.05  (0.85–1.28) | | | 0.660 | | | |  |  | | | | |
| 120-139 | | | | 472,610 | | | | | 3,669 | | | | | | | | 0.61 | | 1.31  (1.13–1.53) | | | | | <0.001 | | | | | | | <0.001 | | | 202,155 | | 5,102 | | | | 2.12 | | | | | | | | | | 1.16  (0.95–1.42) | | | 0.155 | | | | <0.001 |  | | | | |
| 140-159 | | | | 107,665 | | | | | 1,076 | | | | | | | | 0.79 | | 1.51  (1.29–1.78) | | | | | <0.001 | | | | | | |  | | | 76,305 | | 2,107 | | | | 2.34 | | | | | | | | | | 1.24  (1.01–1.52) | | | 0.042 | | | |  |  | | | | |
| ≥160 | | | | 22,838 | | | | | 314 | | | | | | | | 1.09 | | 1.99  (1.65–2.40) | | | | | <0.001 | | | | | |  | | | | 23,289 | | 742 | | | | 2.77 | | | | | | | | | | 1.40  (1.13–1.73) | | | 0.002 | | | |  |  | | | | |
| **Diastolic BP, mmHg** | | | | | | | | | | | | | | | | | | | | | | | | | | | | | | | | | | | | | | | | | | | | | | | | | | | | | | | | | | | | | | |
| <60 | 24,975 | | | | | | | | | 124 | | | | | | 0.39 | | | (ref) | | | | | | | | | | | | |  | | 5,642 | | | | 135 | | | | | | 2.04 | | | | | (ref) | | | | | | | |  | <0.001 | | | | |
| 60-69 | 183,820 | | | | | | | | | 1,068 | | | | | | 0.46 | | | 1.12  (0.93–1.35) | | | | | | 0.241 | | | | | | |  | | 50,678 | | | | 1,169 | | | | | | 1.94 | | | | | 0.97  (0.82–1.16) | | | | 0.770 | | | |  |  | | | | |
| 70-79 | | 361,493 | | | | | | | | | | 2,541 | | | 0.55 | | | | | 1.26  (1.05–1.51) | | | | | 0.013 | | | | | | | <0.001 | | 130,281 | | | | 3,193 | | | | | | | | 2.06 | | | 1.04  (0.88–1.24) | | | | 0.642 | | | | <0.001 |  | | | | |
| 80-89 | | 323,773 | | | | | | | | | | 2,681 | | | 0.65 | | | | | 1.40  (1.17–1.68) | | | | | <0.001 | | | | | | |  | | 145,613 | | | | 3,735 | | | | | | | | 2.17 | | | 1.08  (0.91–1.28) | | | | 0.393 | | | |  |  | | | | |
| ≥90 | | 105,770 | | | | | | | | | | 1,018 | | | 0.76 | | | | | 1.54  (1.28–1.86) | | | | | <0.001 | | | | | | |  | | 59,688 | | | | 1,707 | | | | | | | | 2.44 | | | 1.20  (1.00–1.42) | | | | 0.047 | | | |  |  | | | | |
| **Non-HDL-c, mg/dL** | | | | | | | | | | | | | | | | | | | | | | | | | | | | | | | | | | | | | | | | | | | | | | | | | | | | | | | | | | | | | | |
| <130 | | | 288,643 | | | | | | | | 1,808 | | | 0.49 | | | | (ref) | | | | | | | | | | |  | | | | | 109,894 | | | 2,341 | | | | | | | | 1.82 | | | | (ref) | | | | | |  | | | <0.001 | | | |  |
| 130-159 | | | 322,271 | | | | | | | | 2,159 | | | 0.52 | | | | 1.07  (1.00–1.14) | | | | 0.036 | | | | | | |  | | | | | 124,021 | | | 2,890 | | | | | | | | 1.96 | | | | 1.11  (1.06–1.18) | | | | <0.001 | |  | | |  | | | |  |
| 160-189 | | | 239,246 | | | | | | | | 1,921 | | | 0.63 | | | | 1.23  (1.16–1.32) | | | | <0.001 | | | | | | | <0.001 | | | | | 96,493 | | | 2,654 | | | | | | | | 2.31 | | | | 1.33  (1.25–1.40) | | | | <0.001 | | <0.001 | | |  | | | |  |
| 190-219 | | | 106,139 | | | | | | | | 1,011 | | | 0.75 | | | | 1.40  (1.30–1.52) | | | | <0.001 | | | | | | |  | | | | | 43,434 | | | 1,358 | | | | | | | | 2.63 | | | | 1.50  (1.40–1.60) | | | | <0.001 | |  | | |  | | | |  |
| ≥220 | | | 43,532 | | | | | | | | 533 | | | 0.96 | | | | 1.71  (1.55–1.89) | | | | <0.001 | | | | | | |  | | | | | 18,060 | | | 696 | | | | | | | | 3.28 | | | | 1.82  (1.67–1.98) | | | | <0.001 | |  | | |  | | | |  |
| **LDL-c, mg/dL** | | | | | | | | | | | | | | | | | | | | | | | | | | | | | | | | | | | | | | | | | | | | | | | | | | | | | | | | | | | | | | |
| <70 | | | 39,592 | | | | | | | | 320 | | | 0.64 | | | | (ref) | | | | | | | | | | |  | | | | | 20,450 | | | 477 | | | | | | | | 2.01 | | | | (ref) | | | | | |  | | | <0.001 | | | |  |
| 70-99 | | | 182,016 | | | | | | | | 1,252 | | | 0.54 | | | | 0.99  (0.88–1.12) | | | | 0.914 | | | | | | |  | | | | | 77,443 | | | 1,805 | | | | | | | | 1.98 | | | | 1.05  (0.95–1.16) | | | | 0.325 | |  | | |  | | | |  |
| 100-129 | | | 337,709 | | | | | | | | 2,271 | | | 0.53 | | | | 1.03  (0.91–1.16) | | | | 0.658 | | | | | | | <0.001 | | | | | 129,376 | | | 3,017 | | | | | | | | 1.97 | | | | 1.09  (0.99–1.20) | | | | 0.091 | | <0.001 | | |  | | | |  |
| 130-159 | | | 276,305 | | | | | | | | 2,104 | | | 0.60 | | | | 1.15  (1.02–1.29) | | | | 0.023 | | | | | | |  | | | | | 104,150 | | | 2,710 | | | | | | | | 2.18 | | | | 1.23  (1.12–1.36) | | | | <0.001 | |  | | |  | | | |  |
| ≥160 | | | 164,209 | | | | | | | | 1,485 | | | 0.71 | | | | 1.31  (1.16–1.48) | | | | <0.001 | | | | | | |  | | | | | 60,483 | | | 1,930 | | | | | | | | 2.68 | | | | 1.52  (1.37–1.68) | | | | <0.001 | |  | | |  | | | |  |
| **Triglycerides, mg/dL** | | | | | | | | | | | | | | | | | | | | | | | | | | | | | | | | | | | | | | | | | | | | | | | | | | | | | | | | | | | | | | |
| <100 | | | 443,795 | | | | | | | | 2,569 | | | 0.45 | | | | (ref) | | | | | | | | | | | <0.001 | | | | | 125,671 | | | 2,558 | | | | | | | | 1.71 | | | | (ref) | | | | | | <0.001 | | | <0.001 | | | |  |
| 100-149 | | | 300,007 | | | | | | | | 2,261 | | | 0.59 | | | | 1.14  (1.08–1.21) | | | | <0.001 | | | | | | |  |  |  |  |  | 133,368 | | | 3,367 | | | | | | | | 2.13 | | | | 1.18  (1.12–1.25) | | | | <0.001 | |  |  |  |  | | | |  |
| 150-199 | | | 137,884 | | | | | | | | 1,210 | | | 0.69 | | | | 1.22  (1.14–1.31) | | | | <0.001 | | | | | | |  |  |  |  |  | 70,690 | | | 1,983 | | | | | | | | 2.37 | | | | 1.28  (1.21–1.36) | | | | <0.001 | |  |  |  |  | | | |  |
| ≥200 | | | 118,145 | | | | | | | | 1,392 | | | 0.92 | | | | 1.52  (1.42–1.62) | | | | <0.001 | | | | | | |  |  |  |  |  | 62,173 | | | 2,031 | | | | | | | | 2.76 | | | | 1.46  (1.37–1.55) | | | | <0.001 | |  |  |  |  | | | |  |
| **Abdominal obesity** | | | | | | | | | | | | | | | | | | | | | | | | | | | | | | | | | | | | | | | | | | | | | | | | | | | | | | | | | | | | | | |
| Yes | | | 213,577 | | | | | | | | | 2,208 | | | 0.81 | | | 1.18  (1.12–1.25) | | | | <0.001 | | | | | | | - | | | | | 135,182 | | | 3,661 | | | | | | | | 2.28 | | | | 1.00  (0.96–1.05) | | | | 0.917 | | | - | | | <0.001 | |  |  |
| **Regular physical activity** | | | | | | | | | | | | | | | | | | | | | | | | | | | | | | | | | | | | | | | | | | | | | | | | | | | | | | | | | | | | | | |
| Yes | | | 201,979 | | | | | | | | | 1,428 | | | 0.55 | | | 1.07  (1.01–1.13) | | | | 0.032 | | | | | | | - | | | | | 59,799 | | | 1,294 | | | | | | | | 1.78 | | | | 1.14  (1.07–1.21) | | | | <0.001 | | - | | | 0.732 | | |  |  |

HRs were estimated with adjustment for age, sex, BMI, smoking, alcohol, exercise, hypertension, diabetes, dyslipidemia, and eGFR, excluding the primary exposure variable under analysis. MI, myocardial infarction; other abbreviations as **Supplementary Table 2** and **3**.

**Supplementary Table 6. Associations of individual risk factors with ischemic stroke risk by age and sex**

1. **Total population**

|  | | **Total** | | | | | | | | | | | | | | **<40 years old** | | | | | | | | | | | | | | | | | | | | | | | | | | | | | | **40-49 years old** | | | | | | | | | | | | | | | | | | | | | | | | | | | | | | | |  |  |
| --- | --- | --- | --- | --- | --- | --- | --- | --- | --- | --- | --- | --- | --- | --- | --- | --- | --- | --- | --- | --- | --- | --- | --- | --- | --- | --- | --- | --- | --- | --- | --- | --- | --- | --- | --- | --- | --- | --- | --- | --- | --- | --- | --- | --- | --- | --- | --- | --- | --- | --- | --- | --- | --- | --- | --- | --- | --- | --- | --- | --- | --- | --- | --- | --- | --- | --- | --- | --- | --- | --- | --- | --- | --- | --- | --- | --- | --- | --- | --- |
|  | | **N** | | **Events** | | | **IR** | | | **HR**  **(95% CI)** | | ***p*** | | | ***p*_trend_** | **N** | | | **Events** | | | | | | | | | | **IR** | | | | **HR**  **(95% CI)** | | | | | | ***p*** | | | | | ***p*_trend_** | | **N** | | | | | | | | **Events** | | | | | | **IR** | | | | **HR**  **(95% CI)** | | | | | | ***p*** | | | | | | ***p*_trend_** | | | |
| **BMI, kg/m2** | | | | | | | | | | | | | | | | | | | | | | | | | | | | | | | | | | | | | | | | | | | | | | | | | | | | | | | | | | | | | | | | | | | | | | | | | | | | | |  |  |
| <18.5 | | 238,814 | | 4,782 | | 1.63 | | | 1.01  (0.98–1.04) | | | 0.617 | | |  | 136,039 | | | 307 | | | | | | | | | | 0.18 | | | | 1.03  (0.92–1.16) | | | | | | 0.607 | | <0.001 | | | | | 41,859 | | | | | | | | 379 | | | | | | 0.71 | | | | 0.96  (0.87–1.07) | | | | | | 0.467 | | | | | | <0.001 | | | |
| 18.5-22.9 | | 2,492,083 | | 60,775 | | 1.95 | | | (ref) | | | | | |  | 878,129 | | | 2,418 | | | | | | | | | | 0.21 | | | | (ref) | | | | | | | |  |  |  |  |  | 700,605 | | | | | | | | 7,244 | | | | | | 0.81 | | | | (ref) | | | | | | | | | | | |  |  |  |  |
| 23.0-24.9 | | 1,530,969 | | 47,594 | | 2.48 | | | 1.01  (1.00–1.02) | | | 0.165 | <0.001 | | | 376,021 | | | 1,566 | | | | | | | | | | 0.32 | | | | 1.21  (1.13–1.29) | | | | | | <0.001 | |  |  |  |  |  | 431,671 | | | | | | | | 5,718 | | | | | | 1.04 | | | | 1.09  (1.05–1.13) | | | | | | <0.001 | | | | | |  |  |  |  |
| 25.0-29.9 | | 1,773,812 | | 59,795 | | 2.69 | | | 1.04  (1.02–1.05) | | | <0.001 |  | | | 435,435 | | | 2,427 | | | | | | | | | | 0.43 | | | | 1.38  (1.29–1.46) | | | | | | <0.001 | |  |  |  |  |  | 487,357 | | | | | | | | 7,748 | | | | | | 1.25 | | | | 1.16  (1.12–1.20) | | | | | | <0.001 | | | | | |  |  |  |  |
| ≥30.0 | | 214,174 | | 6,823 | | 2.54 | | | 1.16  (1.14–1.19) | | | <0.001 |  | | | 76,779 | | | 733 | | | | | | | | | | 0.74 | | | | 2.07  (1.89–2.26) | | | | | | <0.001 | |  |  |  |  |  | 56,355 | | | | | | | | 1,090 | | | | | | 1.53 | | | | 1.31  (1.23–1.40) | | | | | | <0.001 | | | | | |  |  |  |  |
| **Smoking** | | | | | | | | | | | | | | | | | | | | | | | | | | | | | | | | | | | | | | | | | | | | | | | | | | | | | | | | | | | | | | | | | | | | | | | | | | | | | |  |  |
| Non | | 3,827,796 | | 106,495 | | 2.22 | | | (ref) | | | | | | <0.001 | 1,020,523 | | | | | | | | 2,993 | | | | | 0.23 | | | | (ref) | | | | | | | | <0.001 | | | | | 1,041,334 | | | | | | 10,468 | | | | | | | 0.79 | | | | (ref) | | | | | | | | | | |  | | | |  |  |
| Ex | | 863,923 | | 26,779 | | 2.49 | | | 0.95  (0.94–0.97) | | | <0.001 | | |  | 208,465 | | | 889 | | | | | | | | | | 0.33 | | | | 1.02  (0.94–1.11) | | | | | | 0.678 | |  |  |  |  |  | 243,998 | | | | | | | 2,910 | | | | | | 0.93 | | | | 0.90  (0.86–0.95) | | | | | <0.001 | | | | | | <0.001 | | | |  |  |
| Current | | 1,558,133 | | 46,495 | | 2.39 | | | 1.55  (1.53–1.57) | | | <0.001 | | |  | 673,415 | | | 3,569 | | | | | | | | | | 0.41 | | | | 1.38  (1.30–1.47) | | | | | | <0.001 | |  |  |  |  |  | 432,515 | | | | | | | 8,801 | | | | | | 1.61 | | | | 1.63  (1.57–1.70) | | | | | <0.001 | | | | | |  | | | |  |  |
| **Hypertension** | | | | | | | | | | | | | | | | | | | | | | | | | | | | | | | | | | | | | | | | | | | | | | | | | | | | | | | | | | | | | | | | | | | | | | | | | | | | | |  |  |
| Yes | | 1,395,180 | | 84,338 | | 5.00 | | | 1.34  (1.33–1.35) | | <0.001 | | | | - | 144,432 | | 1,508 | | | | | | | | | | 0.81 | | | | | 1.98  (1.86–2.10) | | | | | | <0.001 | | - | | | | | 270,642 | | | | | 6,324 | | | | | | 1.84 | | | | | | 1.67  (1.62–1.72) | | | | | <0.001 | | | | | | | | - | | |  |
| **Diabetes** | | | | | | | | | | | | | | | | | | | | | | | | | | | | | | | | | | | | | | | | | | | | | | | | | | | | | | | | | | | | | | | | | | | | | | | | | | | | | |  |  |
| Normo-  glycemia | | 4,347,864 | | 97624 | | 1.78 | | | (ref) | | | | | | <0.001 | 1,561,963 | | | | | | | 5,442 | | | | | | | 0.27 | | | | (ref) | | | | | | | <0.001 | | | | | 1,216,685 | | | | 13,649 | | | | | | 0.88 | | | | | | | (ref) | | | | | | | | | | | <0.001 | | | |  |  |
| Prediabetes | | 1,407,599 | | 46711 | | 2.67 | | | 1.03  (1.02–1.04) | | <0.001 | | | |  | 300,804 | | 1,520 | | | | | | | | | | 0.39 | | | | 1.07  (1.01–1.13) | | | | | | 0.025 | | |  |  |  |  |  | 399,548 | | | | 5,550 | | | | | | 1.09 | | | | | | | 1.04  (1.01–1.08) | | | | 0.011 | | | | | | |  |  |  |  |  |  |
| Diabetes | | 494,389 | | 35434 | | 6.06 | | | 1.51  (1.49–1.53) | | <0.001 | | | |  | 39,636 | | 489 | | | | | | | | | | 0.96 | | | | 1.86  (1.69–2.05) | | | | | | <0.001 | | |  |  |  |  |  | 101,614 | | | | 2,980 | | | | | | 2.33 | | | | | | | 1.78  (1.70–1.85) | | | | <0.001 | | | | | | |  |  |  |  |  |  |
| **Systolic BP, mmHg** | | | | | | | | | | | | | | | | | | | | | | | | | | | | | | | | | | | | | | | | | | | | | | | | | | | | | | | | | | | | | | | | | | | | | | | | | | | | | |  |  |
| <100 | | 221,402 | | 2,490 | | 0.89 | | | (ref) | | | | | |  | 88,092 | | | | | | 165 | | | | | 0.15 | | | | | (ref) | | | | | | | |  | | | | | 74,010 | | | | 504 | | | | | | 0.53 | | | | | | | (ref) | | | | | | | | | | |  | | | | |  |  |
| 100-119 | | 2,371,006 | | 40,898 | | 1.36 | | | 1.14  (1.09–1.19) | | <0.001 | | | |  | 879,732 | | | | | | 2,393 | | | | | 0.21 | | | | | 1.23  (1.05–1.45) | | | | 0.010 | | | |  | | | | | 721,388 | | | | 6,733 | | | | | | 0.73 | | | | | | | 1.20  (1.09–1.31) | | | | <0.001 | | | | | | |  | | | | |  |  |
| 120-139 | | 2,959,527 | | 92,369 | | 2.50 | | | 1.39  (1.33–1.44) | | <0.001 | | | | <0.001 | 844,366 | | | | | | 3,820 | | | | | 0.35 | | | | | 1.65  (1.41–1.94) | | | | <0.001 | | | | <0.001 | | | | | 780,712 | | | | 11,258 | | | | | | 1.13 | | | | | | | 1.60  (1.46–1.75) | | | | <0.001 | | | | | | | <0.001 | | | | |  |  |
| 140-159 | | 568,074 | | 32,940 | | 4.79 | | | 1.67  (1.60–1.74) | | <0.001 | | | |  | 77,539 | | | | | | 790 | | | | | 0.79 | | | | | 2.87  (2.41–3.42) | | | | <0.001 | | | |  | | | | | 118,115 | | | | 2751 | | | | | | 1.84 | | | | | | | 2.33  (2.11–2.57) | | | | <0.001 | | | | | | |  | | | | |  |  |
| ≥160 | | 129,843 | | 11,072 | | 7.30 | | | 2.10  (2.01–2.20) | | <0.001 | | |  | | 12,674 | | | | | | 283 | | | | | 1.76 | | | | 5.56  (4.56–6.79) | | | | | <0.001 | | | |  | | | | | | 23,622 | | | | 933 | | | | | | 3.18 | | | | | | | 3.83  (3.43–4.28) | | | | <0.001 | | | | | | |  | | | |  |  |
| **Diastolic BP, mmHg** | | | | | | | | | | | | | | | | | | | | | | | | | | | | | | | | | | | | | | | | | | | | | | | | | | | | | | | | | | | | | | | | | | | | | | | | | | | | | |  |  |
| <60 | 150,910 | | 96 | | 0.13 | | | (ref) | | | | | | <0.001 | | | 56,909 | | | | | | 333 | | | | 0.53 | | | | | | (ref) | | | | | | | | <0.001 | | | | | | 48,944 | | 811 | | | | | | | | | | 1.87 | | | | | | (ref) | | | | | | | | | | | <0.001 | | | |
| 60-69 | 1,107,039 | | 941 | | 0.18 | | | 1.29  (1.04–1.59) | | | 0.018 | | |  |  |  | 400,546 | | | | | | 2,699 | | | | 0.63 | | | | | | 1.10  (0.98–1.23) | | | | | 0.119 | | |  |  |  |  |  |  | 335,298 | | 7,109 | | | | | | | | | | 2.03 | | | | | | 1.02  (0.95–1.10) | | | | 0.574 | | | | | | |  |  |  |  |
| 70-79 | 2,268,533 | | 2,479 | | 0.25 | | | 1.57  (1.28–1.93) | | | <0.001 | | |  |  |  | 755,821 | | | | | | 6,789 | | | | 0.86 | | | | | | 1.32  (1.18–1.48) | | | | | <0.001 | | |  |  |  |  |  |  | 620,480 | | 20,615 | | | | | | | | | | 2.53 | | | | | | 1.17  (1.09–1.26) | | | | <0.001 | | | | | | |  |  |  |  |
| 80-89 | 2,103,395 | | 2,789 | | 0.37 | | | 1.91  (1.56–2.35) | | | <0.001 | | |  |  |  | 587,863 | | | | | | 8,358 | | | | 1.18 | | | | | | 1.63  (1.46–1.82) | | | | | <0.001 | | |  |  |  |  |  |  | 553,425 | | 26,866 | | | | | | | | | | 3.14 | | | | | | 1.37  (1.27–1.47) | | | | <0.001 | | | | | | |  |  |  |  |
| ≥90 | 619,975 | | 1,146 | | 0.88 | | | 3.54  (2.86–4.38) | | | <0.001 | | |  |  |  | 101,264 | | | | | | 4,000 | | | | 1.98 | | | | | | 2.50  (2.23–2.80) | | | | | <0.001 | | |  |  |  |  |  |  | 159,700 | | 12,509 | | | | | | | | | | 4.12 | | | | | | 1.71  (1.59–1.84) | | | | <0.001 | | | | | | |  |  |  |  |
| **Non-HDL-c, mg/dL** | | | | | | | | | | | | | | | | | | | | | | | | | | | | | | | | | | | | | | | | | | | | | | | | | | | | | | | | | | | | | | | | | | | | | | | | | | | | | |  |  |
| <130 | 2,589,305 | | 57,694 | | 1.78 | | | (ref) | | | | | <0.001 | | | | 1,041,985 | | | | | 2,992 | | | | | 0.22 | | | | | | (ref) | | | | | | | | | | <0.001 | | | | 700,724 | 7,306 | | | | | | | | | | | | | 0.82 | | | | (ref) | | | | | | | | | | | | <0.001 | | |
| 130-159 | 1,913,101 | | 56,868 | | 2.37 | | | 1.08  (1.06–1.09) | | | <0.001 | |  |  |  |  | 510,962 | | | | | 2,209 | | | | | 0.33 | | | | | | 1.12  (1.06–1.18) | | | | | 0.914 | | | | |  |  |  |  | 549,841 | 6,952 | | | | | | | | | | | | | 0.99 | | | | 1.07  (1.04–1.11) | | | | | | | <0.001 | | | | |  |  |  |
| 160-189 | 1,150,763 | | 40,581 | | 2.82 | | | 1.15  (1.14–1.17) | | | <0.001 | |  |  |  |  | 242,787 | | | | | 1,450 | | | | | 0.46 | | | | | | 1.26  (1.18–1.35) | | | | | <0.001 | | | | |  |  |  |  | 314,844 | 4,904 | | | | | | | | | | | | | 1.22 | | | | 1.19  (1.15–1.24) | | | | | | | <0.001 | | | | |  |  |  |
| 190-219 | 436,771 | | 17,316 | | 3.18 | | | 1.22  (1.20–1.24) | | | <0.001 | |  |  |  |  | 79,766 | | | | | 541 | | | | | 0.53 | | | | | | 1.25  (1.13–1.38) | | | | | <0.001 | | | | |  |  |  |  | 112,950 | 2,074 | | | | | | | | | | | | | 1.44 | | | | 1.30  (1.23–1.36) | | | | | | | <0.001 | | | | |  |  |  |
| ≥220 | 159,912 | | 7,310 | | 3.70 | | | 1.36  (1.32–1.39) | | | <0.001 | |  |  |  |  | 26,903 | | | | | 259 | | | | | 0.75 | | | | | | 1.60  (1.41–1.81) | | | | | <0.001 | | | | |  |  |  |  | 39,488 | 943 | | | | | | | | | | | | | 1.89 | | | | 1.57  (1.47–1.69) | | | | | | | <0.001 | | | | |  |  |  |
| **LDL-c, mg/dL** | | | | | | | | | | | | | | | | | | | | | | | | | | | | | | | | | | | | | | | | | | | | | | | | | | | | | | | | | | | | | | | | | | | | | | | | | | | | | |  |  |
| <70 | 475,298 | | 13,564 | | 2.30 | | | (ref) | | | | | 0.059 | | | | 190,321 | | | | | 688 | | | | | 0.28 | | | | | | (ref) | | | | | | | | | | <0.001 | | | | 116,751 | | 1,722 | | | | | | | | | | | | 1.16 | | | | (ref) | | | | | | | | | | | <0.001 | | | |
| 70-99 | 1,703,244 | | 40,564 | | 1.90 | | | 0.95  (0.93–0.97) | | | <0.001 | |  |  |  |  | 674,961 | | | | | 2140 | | | | | 0.25 | | | | | | 0.91  (0.84–1.00) | | | | 0.037 | | | | | |  |  |  |  | 457,891 | | 5,178 | | | | | | | | | | | | 0.89 | | | | 0.90  (0.85–0.95) | | | | | | <0.001 | | | | |  |  |  |  |
| 100-129 | 2,212,393 | | 60,906 | | 2.19 | | | 1.00  (0.98–1.02) | | | 0.787 | |  |  |  |  | 666,476 | | | | | 2605 | | | | | 0.30 | | | | | | 0.96  (0.89–1.05) | | | | 0.390 | | | | | |  |  |  |  | 642,418 | | 7,692 | | | | | | | | | | | | 0.94 | | | | 0.93  (0.88–0.98) | | | | | | 0.006 | | | | |  |  |  |  |
| 130-159 | 1,294,595 | | 42,730 | | 2.64 | | | 1.05  (1.03–1.07) | | | <0.001 | |  |  |  |  | 281,961 | | | | | 1453 | | | | | 0.40 | | | | | | 1.06  (0.97–1.17) | | | | 0.194 | | | | | |  |  |  |  | 361,073 | | 5,183 | | | | | | | | | | | | 1.12 | | | | 1.04  (0.99–1.10) | | | | | | 0.160 | | | | |  |  |  |  |
| ≥160 | 564,322 | | 22,005 | | 3.13 | | | 1.12  (1.10–1.15) | | | <0.001 | |  |  |  |  | 88,684 | | | | | 565 | | | | | 0.49 | | | | | | 1.15  (1.02–1.28) | | | | 0.018 | | | | | |  |  |  |  | 139,714 | | 2,404 | | | | | | | | | | | | 1.35 | | | | 1.17  (1.10–1.24) | | | | | | <0.001 | | | | |  |  |  |  |
| **Triglycerides, mg/dL** | | | | | | | | | | | | | | | | | | | | | | | | | | | | | | | | | | | | | | | | | | | | | | | | | | | | | | | | | | | | | | | | | | | | | | | | | | | | | |  |  |
| <100 | 2,805,260 | | 57,715 | | 1.63 | | | (ref) | | | | | <0.001 | | | | 1,025,571 | | | | | 2,840 | | | | | 0.21 | | | | | | (ref) | | | | | | | | | | <0.001 | | | | 793,014 | | 7,633 | | | | | | | | | | | 0.75 | | | | | (ref) | | | | | | | | | | <0.001 | | | |  |
| 100-149 | 1,669,525 | | 54,601 | | 2.62 | | | 1.10  (1.09–1.11) | | | <0.001 | |  |  |  |  | 428,981 | | | | | 1,851 | | | | | 0.33 | | | | | | 1.14  (1.07–1.21) | | | | <0.001 | | | | | |  |  |  |  | 439,169 | | 5,793 | | | | | | | | | | | 1.03 | | | | | 1.12  (1.08–1.16) | | | | | <0.001 | | | | |  |  |  |  |  |
| 150-199 | 844,984 | | 31,499 | | 2.99 | | | 1.17  (1.15–1.18) | | | <0.001 | |  |  |  |  | 205,143 | | | | | 1,051 | | | | | 0.40 | | | | | | 1.12  (1.04–1.21) | | | | 0.003 | | | | | |  |  |  |  | 221,637 | | 3,550 | | | | | | | | | | | 1.26 | | | | | 1.19  (1.14–1.24) | | | | | <0.001 | | | | |  |  |  |  |  |
| ≥200 | 930,083 | | 35,954 | | 3.10 | | | 1.25  (1.23–1.27) | | | <0.001 | |  |  |  |  | 242,708 | | | | | 1,709 | | | | | 0.54 | | | | | | 1.26  (1.17–1.35) | | | | <0.001 | | | | | |  |  |  |  | 264,027 | | 5,203 | | | | | | | | | | | 1.55 | | | | | 1.28  (1.23–1.33) | | | | | <0.001 | | | | |  |  |  |  |  |
| **Abdominal obesity** | | | | | | | | | | | | | | | | | | | | | | | | | | | | | | | | | | | | | | | | | | | | | | | | | | | | | | | | | | | | | | | | | | | | | | | | | | | | | |  |  |
| Yes | 1,176,285 | | 52,380 | | 3.61 | | | 1.09  (1.07–1.10) | | | <0.001 | | - | | | | 245,998 | | | | 1,758 | | | | | 0.55 | | | | | | | 1.38  (1.30–1.46) | | | <0.001 | | | | | | - | | | | | 272,361 | | 4,930 | | | | | | | | | | 1.42 | | | | | | 1.16  (1.12–1.20) | | | <0.001 | | | | | | - | | | |  |  |
| **Regular physical activity** | | | | | | | | | | | | | | | | | | | | | | | | | | | | | | | | | | | | | | | | | | | | | | | | | | | | | | | | | | | | | | | | | | | | | | | | | | | | | |  |  |
| Yes | 1,121,280 | | 33,857 | | 2.41 | | | 1.12  (1.11–1.13) | | | <0.001 | | - | | | | 254,136 | | | 1,067 | | | | | 0.32 | | | | | | | | 1.00  (0.93–1.06) | | 0.879 | | | | | | | - | | | | | 304,750 | | 3,875 | | | | | | | | | 0.99 | | | | | | | 1.05  (1.01–1.09) | | | 0.008 | | | | | | - | | | |  |  |

|  | | | | **50-64 years old** | | | | | | | | | | | | | | | | | | | | | | | | | | | | | | **≥65 years old** | | | | | | | | | | | | | | | | | | | | | | | | | | | | | | | |  | | | |
| --- | --- | --- | --- | --- | --- | --- | --- | --- | --- | --- | --- | --- | --- | --- | --- | --- | --- | --- | --- | --- | --- | --- | --- | --- | --- | --- | --- | --- | --- | --- | --- | --- | --- | --- | --- | --- | --- | --- | --- | --- | --- | --- | --- | --- | --- | --- | --- | --- | --- | --- | --- | --- | --- | --- | --- | --- | --- | --- | --- | --- | --- | --- | --- | --- | --- | --- | --- | --- | --- |
|  | | | | **N** | | **Events** | | | | | | | **IR** | | | | | | **HR**  **(95% CI)** | | | | | | | ***p*** | ***p*_trend_** | | | | | | **N** | | | | | | **Events** | | | | | | | **IR** | | **HR**  **(95% CI)** | | | | | | | ***p*** | | | | | ***p*_trend_** | | | | | | ***p*_interaction_** | | | |
| **BMI, kg/m2** | | | | | | | | | | | | | | | | | | | | | | | | | | | | | | | | | | | | | | | | | | | | | | | | | | | | | | | | | | | | | | | | | | | | | |
| <18.5 | | | | 32,068 | | 1124 | | | | | | | 2.91 | | | | | | 1.08  (1.02–1.15) | | | | | | | 0.010 | | <0.001 | | | | | 28,848 | | | | | | 2,972 | | | | | | | | 11.22 | | 0.99  (0.95–1.03) | | | | | | 0.590 | | | | | <0.001 | | | | | | <0.001 | | | |
| 18.5-22.9 | | | | 646,731 | | 20910 | | | | | | | 2.59 | | | | | | (ref) | | | | | | | | |  |  |  |  |  | 266,618 | | | | | | 30,203 | | | | | | | | 10.57 | | (ref) | | | | | | | | | | |  |  |  |  |  |  |  | | | |
| 23.0-24.9 | | | | 530,856 | | 18936 | | | | | | | 2.85 | | | | | | 1.01  (0.99–1.03) | | | | | | | 0.254 | |  |  |  |  |  | 192,421 | | | | | | 21,374 | | | | | | | | 9.88 | | 0.96  (0.95–0.98) | | | | | | <0.001 | | | | |  |  |  |  |  |  |  | | | |
| 25.0-29.9 | | | | 624,841 | | 24346 | | | | | | | 3.12 | | | | | | 1.03  (1.01–1.05) | | | | | | | 0.001 | |  |  |  |  |  | 226,179 | | | | | | 25,274 | | | | | | | | 9.80 | | 0.96  (0.95–0.98) | | | | | | <0.001 | | | | |  |  |  |  |  |  |  | | | |
| ≥30.0 | | | | 59,804 | | 2594 | | | | | | | 3.49 | | | | | | 1.13  (1.08–1.18) | | | | | | | <0.001 | |  |  |  |  |  | 21,236 | | | | | | 2,406 | | | | | | | | 9.95 | | 0.99  (0.95–1.03) | | | | | | 0.576 | | | | |  |  |  |  |  |  |  | | | |
| **Smoking** | | | | | | | | | | | | | | | | | | | | | | | | | | | | | | | | | | | | | | | | | | | | | | | | | | | | | | | | | | | | | | | | | | | | | |
| Non | | | | 1,240,131 | 36,984 | | | | | | | | | | | 2.36 | | | (ref) | | | | | | | | | <0.001 | | | | | | 525,808 | 56,050 | | | | | | | | 9.44 | | | | | | | | | (ref) | | | | | | | | <0.001 | | | | | | <0.001 | | | |
| Ex | | | | 300,261 | 10,737 | | | | | | | | | | | 2.87 | | | 0.93  (0.90–0.95) | | <0.001 | | | | | | |  |  |  |  |  |  | 111,199 | 12,243 | | | | | | | | 10.29 | | | | | | | | 0.97  (0.95–0.99) | | | | 0.009 | | | | |  |  |  |  |  |  |  | | | |
| Current | | | | 353,908 | 20,189 | | | | | | | | | | | 4.71 | | | 1.64  (1.60–1.67) | | <0.001 | | | | | | |  |  |  |  |  |  | 98,295 | 13,936 | | | | | | | | 14.24 | | | | | | | | 1.42  (1.39–1.45) | | | | <0.001 | | | | |  |  |  |  |  |  |  | | | |
| **Hypertension** | | | | | | | | | | | | | | | | | | | | | | | | | | | | | | | | | | | | | | | | | | | | | | | | | | | | | | | | | | | | | | | | | | | | | |
| Yes | | | | 600,316 | | | 29,138 | | | | | | | | | 3.93 | | | 1.37  (1.35–1.39) | | <0.001 | | | | | | | - | | | | | | 379,790 | | | | 47,368 | | | | 11.42 | | | | | | | | 1.23  (1.21–1.24) | | | | | <0.001 | | | | | - | | | | | | <0.001 | | | |
| **Diabetes** | | | | | | | | | | | | | | | | | | | | | | | | | | | | | | | | | | | | | | | | | | | | | | | | | | | | | | | | | | | | | | | | | | | | | |
| Normoglycemia | | | | 1,161,480 | | | | 35,794 | | | | | | | | 2.45 | | (ref) | | | | | | | | | | | | | <0.001 | | | 407,736 | | | | 42,739 | | | 9.37 | | | | | | | | (ref) | | | | | | | | | |  | | | | | | | <0.001 | | | |
| Prediabetes | | | | 509,040 | | | 18,223 | | | | | | | | | 2.87 | | 1.04  (1.02–1.05) | | | | | <0.001 | | | | | | | |  |  |  | 198,207 | | | | 21,418 | | | 9.77 | | | | | | | | 1.01  (0.99–1.02) | | | | | | 0.427 | | | | | | | | <0.001 | | |  | | | |
| Diabetes | | | | 223,780 | | | 13,893 | | | | | | | | | 5.13 | | 1.60  (1.57–1.64) | | | | | <0.001 | | | | | | | |  |  |  | 129,359 | | | 18,072 | | | | 13.35 | | | | | | | | 1.37  (1.35–1.40) | | | | | | <0.001 | | | | | | | |  | | |  | | | |
| **Systolic BP, mmHg** | | | | | | | | | | | | | | | | | | | | | | | | | | | | | | | | | | | | | | | | | | | | | | | | | | | | | | | | | | | | | | | | | | | | | |
| <100 | | | | 50,473 | | | | | 1,088 | | | | | | | | 1.71 | | (ref) | | | | | | | | | | | |  | | | 8,827 | | 733 | | | | 7.69 | | | | | | | | (ref) | | | | | | | | | |  | | | | | | | | <0.001 | | | |
| 100-119 | | | | 609,874 | | | | | 16,500 | | | | | | | | 2.15 | | 1.12  (1.05–1.19) | | | | | <0.001 | | | | | | |  | | | 160,012 | | 15,272 | | | | 8.58 | | | | | | | | 1.09  (1.01–1.17) | | | | | | | 0.803 | | | | | | | | |  | |  | | | |
| 120-139 | | | | 953,152 | | | | | 35,258 | | | | | | | | 2.96 | | 1.37  (1.29–1.46) | | | | | <0.001 | | | | | | | <0.001 | | | 381,297 | | 42,033 | | | | 9.94 | | | | | | | | 1.24  (1.15–1.33) | | | | | | | 0.001 | | | | | | | | | <0.001 | |  | | | |
| 140-159 | | | | 229,942 | | | | | 11,574 | | | | | | | | 4.08 | | 1.73  (1.62–1.84) | | | | | <0.001 | | | | | | |  | | | 142,478 | | 17,825 | | | | 11.45 | | | | | | | | 1.39  (1.29–1.49) | | | | | | | <0.001 | | | | | | | | |  | |  | | | |
| ≥160 | | | | 50,859 | | | | | 3,490 | | | | | | | | 5.68 | | 2.30  (2.15–2.46) | | | | | <0.001 | | | | | |  | | | | 42,688 | | 6,366 | | | | 14.21 | | | | | | | | 1.66  (1.53–1.79) | | | | | | | <0.001 | | | | | | | | |  | |  | | | |
| **Diastolic BP, mmHg** | | | | | | | | | | | | | | | | | | | | | | | | | | | | | | | | | | | | | | | | | | | | | | | | | | | | | | | | | | | | | | | | | | | | | |
| <60 | 34,405 | | | | | | | | | 811 | | | | | | 1.87 | | | (ref) | | | | | | | | | | | | |  | | 10,652 | | | | 1,005 | | | | | | 8.77 | | | | | | | | | (ref) | | |  | | | | | | | | |  | <0.001 | | | |
| 60-69 | 278,331 | | | | | | | | | 7,109 | | | | | | 2.03 | | | 1.02  (0.95–1.10) | | | | | | 0.574 | | | | | | |  | | 92,864 | | | | 8,830 | | | | | | 8.58 | | | | | | | | | 1.01  (0.95–1.08) | | | 0.803 | | | | | | | | |  |  | | | |
| 70-79 | | 649,250 | | | | | | | | | | 20,615 | | | 2.53 | | | | | 1.17  (1.09–1.26) | | | | | <0.001 | | | | | | | <0.001 | | 242,982 | | | | 25,209 | | | | | | | 9.35 | | | | | | | | | 1.11  (1.04–1.18) | | | 0.002 | | | <0.001 | | | | | | |  | | |
| 80-89 | | 686,011 | | | | | | | | | | 26,866 | | | 3.14 | | | | | 1.37  (1.27–1.47) | | | | | <0.001 | | | | | | |  | | 276,096 | | | | 32,116 | | | | | | | 10.54 | | | | | | | | | 1.24  (1.16–1.32) | | | <0.001 | | |  | | | | | | |  | | |
| ≥90 | | 246,303 | | | | | | | | | | 12,509 | | | 4.12 | | | | | 1.71  (1.59–1.84) | | | | | <0.001 | | | | | | |  | | 112,708 | | | | 15,069 | | | | | | | 12.36 | | | | | | | | | 1.44  (1.35–1.53) | | | <0.001 | | |  | | | | | | |  | | |
| **Non-HDL-c, mg/dL** | | | | | | | | | | | | | | | | | | | | | | | | | | | | | | | | | | | | | | | | | | | | | | | | | | | | | | | | | | | | | | | | | | | | | |
| <130 | | | 591,275 | | | | | | | | 20,160 | | | 2.74 | | | | (ref) | | | | | | | | | | |  | | | | | 255,321 | | | 27,236 | | | | | | | 9.91 | | | | | | | | | (ref) | | | | | | | |  | | | | | <0.001 | | |  |
| 130-159 | | | 618,456 | | | | | | | | 21,650 | | | 2.79 | | | | 1.06  (1.04–1.08) | | | | <0.001 | | | | | | |  | | | | | 233,842 | | | 26,057 | | | | | | | 10.02 | | | | | | | | | 1.07  (1.05–1.08) | | | <0.001 | | | | |  | | | | |  | | |  |
| 160-189 | | | 435,088 | | | | | | | | 16,067 | | | 2.95 | | | | 1.13  (1.10–1.15) | | | | <0.001 | | | | | | | <0.001 | | | | | 158,044 | | | 18,160 | | | | | | | 10.25 | | | | | | | | | 1.12  (1.10–1.14) | | | <0.001 | | | | | <0.001 | | | | |  | | |  |
| 190-219 | | | 180,448 | | | | | | | | 7,092 | | | 3.14 | | | | 1.20  (1.17–1.23) | | | | <0.001 | | | | | | |  | | | | | 63,607 | | | 7,609 | | | | | | | 10.67 | | | | | | | | | 1.19  (1.16–1.22) | | | <0.001 | | | | |  | | | | |  | | |  |
| ≥220 | | | 69,033 | | | | | | | | 2,941 | | | 3.42 | | | | 1.29  (1.24–1.34) | | | | <0.001 | | | | | | |  | | | | | 24,488 | | | 3,167 | | | | | | | 11.67 | | | | | | | | | 1.30  (1.25–1.35) | | | <0.001 | | | | |  | | | | |  | | |  |
| **LDL-c, mg/dL** | | | | | | | | | | | | | | | | | | | | | | | | | | | | | | | | | | | | | | | | | | | | | | | | | | | | | | | | | | | | | | | | | | | | | |
| <70 | | | 114,139 | | | | | | | | 5,027 | | | 3.61 | | | | (ref) | | | | | | | | | | |  | | | | | 54,087 | | | 6127 | | | | | | | 10.84 | | | | | | | | | (ref) | | | | | | | |  | | | | | <0.001 | | |  |
| 70-99 | | | 398,665 | | | | | | | | 14,383 | | | 2.90 | | | | 0.95  (0.92–0.98) | | | | 0.002 | | | | | | |  | | | | | 171,727 | | | 18,863 | | | | | | | 10.13 | | | | | | | | | 0.99  (0.96–1.02) | | | 0.369 | | | | |  | | | | |  | | |  |
| 100-129 | | | 653,239 | | | | | | | | 22,767 | | | 2.78 | | | | 0.99  (0.96–1.02) | | | | 0.557 | | | | | | | <0.001 | | | | | 250,260 | | | 27,842 | | | | | | | 10.05 | | | | | | | | | 1.03  (1.00–1.06) | | | 0.076 | | | | | <0.001 | | | | |  | | |  |
| 130-159 | | | 478,788 | | | | | | | | 16,734 | | | 2.79 | | | | 1.03  (1.00–1.06) | | | | 0.087 | | | | | | |  | | | | | 172,773 | | | 19,360 | | | | | | | 9.99 | | | | | | | | | 1.06  (1.03–1.09) | | | <0.001 | | | | |  | | | | |  | | |  |
| ≥160 | | | 249,469 | | | | | | | | 8,999 | | | 2.88 | | | | 1.09  (1.05–1.13) | | | | <0.001 | | | | | | |  | | | | | 86,455 | | | 10,037 | | | | | | | 10.33 | | | | | | | | | 1.13  (1.09–1.16) | | | <0.001 | | | | |  | | | | |  | | |  |
| **Triglycerides, mg/dL** | | | | | | | | | | | | | | | | | | | | | | | | | | | | | | | | | | | | | | | | | | | | | | | | | | | | | | | | | | | | | | | | | | | | | |
| <100 | | | 727,549 | | | | | | | | 21,159 | | | 2.32 | | | | (ref) | | | | | | | | | | | <0.001 | | | | | 259,126 | | | 26,083 | | | | | | | 9.15 | | | | | | | | | (ref) | | | | | | | | <0.001 | | | | | <0.001 | | |  |
| 100-149 | | | 561,527 | | | | | | | | 20,144 | | | 2.87 | | | | 1.10  (1.08–1.12) | | | | <0.001 | | | | | | |  |  |  |  |  | 239,848 | | | 26,813 | | | | | | | 10.14 | | | | | | | | | 1.09  (1.07–1.11) | | | <0.001 | | | | |  |  |  |  |  |  | | |  |
| 150-199 | | | 294,398 | | | | | | | | 12,103 | | | 3.30 | | | | 1.17  (1.15–1.20) | | | | <0.001 | | | | | | |  |  |  |  |  | 123,806 | | | 14,795 | | | | | | | 10.80 | | | | | | | | | 1.15  (1.12–1.17) | | | <0.001 | | | | |  |  |  |  |  |  | | |  |
| ≥200 | | | 310,826 | | | | | | | | 14,504 | | | 3.76 | | | | 1.23  (1.20–1.26) | | | | <0.001 | | | | | | |  |  |  |  |  | 112,522 | | | 14,538 | | | | | | | 11.73 | | | | | | | | | 1.23  (1.20–1.25) | | | <0.001 | | | | |  |  |  |  |  |  | | |  |
| **Abdominal obesity** | | | | | | | | | | | | | | | | | | | | | | | | | | | | | | | | | | | | | | | | | | | | | | | | | | | | | | | | | | | | | | | | | | | | | |
| Yes | | | 434,187 | | | | | | | | | 19,070 | | | 3.53 | | | 1.09  (1.07–1.11) | | | | <0.001 | | | | | | | - | | | | | 223,739 | | | 26,622 | | | | | | | 10.66 | | | | | | | | | 1.04  (1.03–1.06) | | | <0.001 | | | | | | - | | | | | <0.001 |  |  |
| **Regular physical activity** | | | | | | | | | | | | | | | | | | | | | | | | | | | | | | | | | | | | | | | | | | | | | | | | | | | | | | | | | | | | | | | | | | | | | |
| Yes | | | 412,891 | | | | | | | | | 13,987 | | | 2.71 | | | 1.12  (1.10–1.14) | | | | <0.001 | | | | | | | - | | | | | 149,503 | | | 14,928 | | | | | | | 8.82 | | | | | | | | | 1.17  (1.15–1.19) | | | <0.001 | | | | | - | | | | | <0.001 | |  |  |

1. **Men**

|  | | **Total** | | | | | | | | | | | | | | | **<40 years old** | | | | | | | | | | | | | | | | | | | | | | | | | | | **40-49 years old** | | | | | | | | | | | | | | | | | | | | | | | | | | |
| --- | --- | --- | --- | --- | --- | --- | --- | --- | --- | --- | --- | --- | --- | --- | --- | --- | --- | --- | --- | --- | --- | --- | --- | --- | --- | --- | --- | --- | --- | --- | --- | --- | --- | --- | --- | --- | --- | --- | --- | --- | --- | --- | --- | --- | --- | --- | --- | --- | --- | --- | --- | --- | --- | --- | --- | --- | --- | --- | --- | --- | --- | --- | --- | --- | --- | --- | --- | --- | --- | --- |
|  | | **N** | **Events** | | | **IR** | | | **HR**  **(95% CI)** | ***p*** | | | | | ***p*_trend_** | | **N** | | | **Events** | | | | | | | | **IR** | | | | **HR**  **(95% CI)** | ***p*** | | | | | | | ***p*_trend_** | | | | **N** | | | | | **Events** | | | | | | **IR** | | | | | **HR**  **(95% CI)** | | | | | ***p*** | | | | | ***p*_trend_** |
| **BMI, kg/m2** | | | | | | | | | | | | | | | | | | | | | | | | | | | | | | | | | | | | | | | | | | | | | | | | | | | | | | | | | | | | | | | | | | | | | | |
| <18.5 | | 72,269 | 2,607 | | | 3.10 | | | 1.01  (0.97–1.05) | 0.785 | | | | |  | | 28,778 | | | 82 | | | | | | | | 0.22 | | | | 0.92  (0.74–1.15) | | 0.478 | | | | | |  | | | | 12,816 | | | | | | | 191 | | | | | 1.19 | | | | | 1.03  (0.89–1.20) | | | | 0.653 | | | | |  |
| 18.5-22.9 | | 109,2707 | 34,308 | | | 2.54 | | | (ref) | | |  | | | | | 421,828 | | | 1,402 | | | | | | | | 0.26 | | | | (ref) | | | | | | | |  | | | | 258,213 | | | | | | | 3,803 | | | | | 1.16 | | | | | (ref) | | | | | | | | |  |
| 23.0-24.9 | | 882,703 | 27,862 | | | 2.52 | | | 0.99  (0.97–1.00) | 0.066 | | | <0.001 | | | | 290,363 | | | 1,304 | | | | | | | | 0.35 | | | | 1.20  (1.11–1.29) | | <0.001 | | | | | | <0.001 | | | | 238,396 | | | | | | | 3,716 | | | | | 1.22 | | | | | 1.01  (0.97–1.06) | | | | 0.595 | | | | | <0.001 |
| 25.0-29.9 | | 109,5910 | 33,990 | | | 2.47 | | | 1.01  (0.99–1.02) | 0.385 | | |  | | | | 367,511 | | | 2,142 | | | | | | | | 0.45 | | | | 1.35  (1.26–1.45) | | <0.001 | | | | | |  | | | | 314,407 | | | | | | | 5,671 | | | | | 1.42 | | | | | 1.10  (1.05–1.14) | | | | <0.001 | | | | |  |
| ≥30.0 | | 118,206 | 3,092 | | | 2.08 | | | 1.18  (1.13–1.22) | <0.001 | | |  | | | | 61,126 | | | 613 | | | | | | | | 0.78 | | | | 2.02  (1.83–2.24) | | <0.001 | | | | | |  | | | | 30,488 | | | | | | | 710 | | | | | 1.84 | | | | | 1.26  (1.16–1.37) | | | | <0.001 | | | | |  |
| **Smoking** | | | | | | | | | | | | | | | | | | | | | | | | | | | | | | | | | | | | | | | | | | | | | | | | | | | | | | | | | | | | | | | | | | | | | | |
| Non | | 1,001,024 | 32,894 | | | 2.64 | | | (ref) | | |  | | | | | 348,753 | | | 1,266 | | | | | | | | 0.28 | | | | (ref) | | | | |  | | | | | | | 224,545 | | | | | | | 3,026 | | | | | 1.05 | | | | | (ref) | | | | | | | | |  |
| Ex | | 808,659 | 25,713 | | | 2.56 | | | 0.94  (0.92–0.94) | <0.001 | | | <0.001 | | | | 184,671 | | | 830 | | | | | | | | 0.35 | | | | 1.02  (0.93–1.11) | | 0.014 | | | | | | <0.001 | | | | 228,519 | | | | | | | 2,752 | | | | | 0.94 | | | | | 0.88  (0.83–0.93) | | | | <0.001 | | | | | <0.001 |
| Current | | 1,452,112 | 43,252 | | | 2.38 | | | 1.54  (1.51–1.56) | <0.001 | | |  | | | | 636,182 | | | 3,447 | | | | | | | | 0.42 | | | | 1.39  (1.30–1.48) | | <0.001 | | | | | |  | | | | 401,256 | | | | | | | 8,313 | | | | | 1.63 | | | | | 1.60  (1.54–1.68) | | | | <0.001 | | | | |  |
| **Hypertension** | | | | | | | | | | | | | | | | | | | | | | | | | | | | | | | | | | | | | | | | | | | | | | | | | | | | | | | | | | | | | | | | | | | | | | |
| Yes | | 780,990 | 46,575 | | | 4.97 | | | 1.35  (1.34–1.37) | <0.001 | | | | - | | | 127,945 | | | 1,351 | | | | | | | | 0.82 | | | | 1.94  (1.30–1.48) | | <0.001 | | | | | | - | | | | 178,473 | | | | | | | 4,662 | | | | | 2.07 | | | | | 1.65  (1.59–1.71) | | | | <0.001 | | | | | - |
| **Diabetes** | | | | | | | | | | | | | | | | | | | | | | | | | | | | | | | | | | | | | | | | | | | | | | | | | | | | | | | | | | | | | | | | | | | | | | |
| Normo-glycemia | | 2,110,224 | 51,572 | | | 1.94 | | | (ref) | | | | |  | | | 910,342 | | | | 3,885 | | | | | | 0.33 | | | | (ref) | | | | | | | | |  | | | | 537,980 | | | | | | | 7,733 | | | | | | 1.12 | | | | (ref) | | | | | | | | |  |
| Prediabetes | | 844,976 | 28,652 | | | 2.73 | | | 1.05  (1.03–1.06) | <0.001 | | | | <0.001 | | | 226,609 | | | 1,244 | | | | | | | | 0.42 | | | 1.03  (0.96–1.10) | | | 0.412 | | | | | | <0.001 | | | | 242,535 | | | | | | | 3,993 | | | | | | 1.29 | | | | 1.06  (1.02–1.10) | | | | 0.006 | | | | | <0.001 |
| Diabetes | | 306,595 | 21,635 | | | 6.00 | | | 1.53  (1.51–1.56) | <0.001 | | | |  | | | 32,655 | | | 414 | | | | | | | | 0.99 | | | 1.75  (1.58–1.95) | | | <0.001 | | | | | |  | | | | 73,805 | | | | | | | 2,365 | | | | | | 2.56 | | | | 1.76  (1.68–1.85) | | | | <0.001 | | | | |  |
| **Systolic BP, mmHg** | | | | | | | | | | | | | | | | | | | | | | | | | | | | | | | | | | | | | | | | | | | | | | | | | | | | | | | | | | | | | | | | | | | | | | |
| <100 | | 45,444 | 946 | | | 1.68 | | | (ref) | | | | |  | | | 13,637 | | | | | 28 | | | | | 0.16 | | | | (ref) | | | | | | | | |  | | | | 13,963 | | | | | | | 124 | | | | | | 0.70 | | | | (ref) | | | | | | | | |  |
| 100-119 | | 1,030,001 | 21,282 | | | 1.64 | | | 1.14  (1.07–1.22) | <0.001 | | | |  | | | 419,475 | | | | | 1,353 | | | | | 0.25 | | | | 1.49  (1.02–2.16) | | | 0.039 | | | | | |  | | | | 286,055 | | | | | | | 3,414 | | | | | | 0.93 | | | | 1.36  (1.14–1.63) | | | | <0.001 | | | | |  |
| 120-139 | 1,774,926 | | 53,966 | | | 2.43 | | | 1.41  (1.32–1.50) | <0.001 | | | | <0.001 | | | 654,740 | | | | | 3,197 | | | | | 0.38 | | | | 2.03  (1.40–2.95) | | | <0.001 | | | | | | <0.001 | | | | 460,502 | | | | | | | 7,754 | | | | | | 1.32 | | | | 1.87  (1.56–2.23) | | | | <0.001 | | | | | <0.001 |
| 140-159 | 337,114 | | 19,193 | | | 4.72 | | | 1.75  (1.64–1.87) | <0.001 | | | |  | | | 70,354 | | | | | 720 | | | | | 0.80 | | | | 3.47  (2.38–5.07) | | | <0.001 | | | | | |  | | | | 78,310 | | | | | | | 2,093 | | | | | | 2.12 | | | | 2.80  (2.33–3.36) | | | | <0.001 | | | | |  |
| ≥160 | 74,310 | | 6,472 | | | 7.55 | | | 2.29  (2.14–2.46) | <0.001 | | | |  | | | 11,400 | | | | | 245 | | | | | 1.69 | | 6.48  (4.37–9.61) | | | | | <0.001 | | | | | |  | | | | 15,490 | | | | | | | 706 | | | | | | 3.70 | | | | 4.60  (3.80–5.58) | | | | <0.001 | | | | |  |
| **Diastolic BP, mmHg** | | | | | | | | | | | | | | | | | | | | | | | | | | | | | | | | | | | | | | | | | | | | | | | | | | | | | | | | | | | | | | | | | | | | | | |
| <60 | 38,038 | | 991 | | 2.11 | | (ref) | | | | | | | | |  | | | 13,826 | | | | | | 36 | | | 0.20 | | | | (ref) | | | | | |  | | | | | 9,772 | | | | | | | 73 | | | | | | | 0.59 | | | | (ref) | | | | | | | | |  |
| 60-69 | 390,903 | | 9,302 | | 1.91 | | 1.01  (0.94–1.07) | | | 0.852 | | | | | |  | | | 152,428 | | | | | | 419 | | | 0.21 | | | | 1.00  (0.71–1.41) | | | | 0.982 | | | | |  | | 10,1778 | | | | | | | 1,074 | | | | | | | 0.83 | | | | 1.40  (1.10–1.77) | | | | 0.006 | | | | |  |
| 70-79 | 1,148,566 | | 29,743 | | 2.07 | | 1.13  (1.06–1.20) | | | <0.001 | | | | | | <0.001 | | | 455,400 | | | | | | 1,714 | | | 0.29 | | | | 1.29  (0.93–1.79) | | | | 0.132 | | | | | <0.001 | | 292,708 | | | | | | | 3,945 | | | | | | | 1.05 | | | | 1.74  (1.39–2.19) | | <0.001 | | | | | <0.001 | | |
| 80-89 | 1,291,556 | | 41,629 | 2.58 | | | 1.33  (1.25–1.41) | | | <0.001 | |  | | | | | | | 458,424 | | | | | 2,349 | | | 0.40 | | | | 1.58  (1.14–2.20) | | | | | 0.006 | | | | |  | | 340,411 | | | | | 5,905 | | | | | 1.36 | | | | | 2.16  (1.71–2.72) | | | | | <0.001 | | | | |  | | |
| ≥90 | 392,732 | | 20,194 | 4.22 | | | 1.72  (1.61–1.83) | | | <0.001 | |  | | | | | | | 89,528 | | | | | 1,025 | | | 0.89 | | | | 2.87  (2.05–4.00) | | | | | <0.001 | | | | |  | | 109,651 | | | | | 3,094 | | | | | 2.24 | | | | | 3.37  (2.67–4.25) | | | | | <0.001 | | | | |  | | |
| **Non-HDL-c, mg/dL** | | | | | | | | | | | | | | | | | | | | | | | | | | | | | | | | | | | | | | | | | | | | | | | | | | | | | | | | | | | | | | | | | | | | | | |
| <130 | 1,238,057 | | 34,960 | 2.27 | | | | (ref) | | |  | | | | | | | | 519,536 | | | | | 1,800 | | | 0.27 | | | (ref) | | | | | | | | |  | | | | 270,462 | | | 3,772 | | | | | | | | | | 1.10 | | | | (ref) | | | | | | | | |  | |
| 130-159 | 1,048,325 | | 32,515 | 2.48 | | | | 1.08  (1.07–1.10) | | <0.001 |  | | | | | | | | 359,018 | | | | | 1,752 | | | 0.38 | | | 1.13  (1.05–1.20) | | | | | | <0.001 | | | | |  | 283,301 | | | 4,411 | | | | | | | | | | 1.22 | | | | 1.09  (1.05–1.14) | | | | | <0.001 | | | |  | | |
| 160-189 | 651,023 | | 22,204 | 2.73 | | | | 1.20  (1.18–1.22) | | <0.001 | <0.001 | | | | | | | | 198,705 | | | | | 1,266 | | | 0.49 | | | 1.26  (1.17–1.36) | | | | | | <0.001 | | | | | <0.001 | 194,925 | | | 3,564 | | | | | | | | | | 1.43 | | | | 1.24  (1.18–1.30) | | | | | <0.001 | | | | <0.001 | | |
| 190-219 | 240,973 | | 8,762 | 2.91 | | | | 1.30  (1.26–1.33) | | <0.001 |  | | | | | | | | 69,114 | | | | | 486 | | | 0.54 | | | 1.24  (1.12–1.38) | | | | | | <0.001 | | | | |  | 77,377 | | | 1,602 | | | | | | | | | | 1.63 | | | | 1.34  (1.27–1.43) | | | | | <0.001 | | | |  | | |
| ≥220 | 83,417 | | 3,418 | 3.31 | | | | 1.48  (1.42–1.53) | | <0.001 |  | | | | | | | | 23,233 | | | | | 239 | | | 0.80 | | | 1.64  (1.42–1.88) | | | | | | <0.001 | | | | |  | 28,255 | | | 742 | | | | | | | | | | 2.08 | | | | 1.62  (1.50–1.76) | | | | | <0.001 | | | |  | | |
| **LDL-c, mg/dL** | | | | | | | | | | | | | | | | | | | | | | | | | | | | | | | | | | | | | | | | | | | | | | | | | | | | | | | | | | | | | | | | | | | | | | |
| <70 | 278,230 | | 9,397 | 2.75 | | | (ref) | | | |  | | | | | | | 106,183 | | | | | 474 | | | 0.34 | | | (ref) | | | | | | | | | |  | | | | 63,863 | | | | 1,190 | | | | | | | | 1.47 | | | | (ref) | | | | | | |  | | | | |
| 70-99 | 882,812 | | 24,916 | 2.26 | | | 0.95  (0.92–0.97) | | | <0.001 |  | | | | | | | 366,243 | | | | | 1,423 | | | 0.30 | | | 0.93  (0.83–1.03) | | | | | | 0.147 | | | | | |  | | 205,636 | | | | 3,020 | | | | | | | | 1.15 | | | | 0.89  (0.84–0.96) | | | 0.001 | | | |  | | | | |
| 100-129 | 1,165,890 | | 35,108 | 2.41 | | | 1.00  (0.98–1.02) | | | 0.992 | <0.001 | | | | | | | 420,560 | | | | | 1,954 | | | 0.36 | | | 0.99  (0.90–1.10) | | | | | | 0.866 | | | | | | <0.001 | | 308,916 | | | | 4,784 | | | | | | | | 1.21 | | | | 0.96  (0.90–1.03) | | | 0.231 | | | | <0.001 | | | | |
| 130-159 | 672,370 | | 22,472 | 2.68 | | | 1.08  (1.05–1.10) | | | <0.001 |  | | | | | | | 206,711 | | | | | 1,208 | | | 0.45 | | | 1.10  (0.99–1.22) | | | | | | 0.087 | | | | | |  | | 194,553 | | | | 3,426 | | | | | | | | 1.38 | | | | 1.08  (1.01–1.15) | | | 0.030 | | | |  | | | | |
| ≥160 | 262,493 | | 9,966 | 3.06 | | | 1.20  (1.161–1.23) | | | <0.001 |  | | | | | | | 69,909 | | | | | 484 | | | 0.54 | | | 1.17  (1.03–1.33) | | | | | | 0.017 | | | | | |  | | 81,352 | | | | 1,671 | | | | | | | | 1.62 | | | | 1.22  (1.13–1.32) | | | <0.001 | | | |  | | | | |
| **Triglycerides, mg/dL** | | | | | | | | | | | | | | | | | | | | | | | | | | | | | | | | | | | | | | | | | | | | | | | | | | | | | | | | | | | | | | | | | | | | | | |
| <100 | 1,128,507 | | 30,371 | 2.16 | | | (ref) | | | | <0.001 | | | | | | | 459,303 | | | | | 1,558 | | | 0.26 | | | (ref) | | | | | |  | | | | | | <0.001 | | 251,995 | | | | 3,275 | | | | | | | 1.02 | | | | | (ref) | | | | | | | <0.001 | | | | |
| 100-149 | 919,711 | | 29,955 | 2.62 | | | 1.11  (1.09–1.12) | | | <0.001 |  |  |  |  |  |  |  | 314,417 | | | | | 1,457 | | | 0.36 | | | 1.09  (1.01–1.17) | | | | | | 0.022 | | | | | |  |  | 237,294 | | | | 3,722 | | | | | | | 1.23 | | | | | 1.11  (1.05–1.16) | | | <0.001 | | | |  |  |  |  |  |
| 150-199 | 535,691 | | 18,370 | 2.75 | | | 1.18  (1.16–1.21) | | | <0.001 |  |  |  |  |  |  |  | 174,033 | | | | | 930 | | | 0.41 | | | 1.09  (1.00–1.18) | | | | | | 0.052 | | | | | |  |  | 152,028 | | | | 2,711 | | | | | | | 1.40 | | | | | 1.18  (1.12–1.25) | | | <0.001 | | | |  |  |  |  |  |
| ≥200 | 677,886 | | 23,163 | 2.73 | | | 1.26  (1.23–1.28) | | | <0.001 |  |  |  |  |  |  |  | 221,853 | | | | | 1,598 | | | 0.56 | | | 1.23  (1.13–1.32) | | | | | | <0.001 | | | | | |  |  | 213,003 | | | | 4,383 | | | | | | | 1.62 | | | | | 1.25  (1.19–1.31) | | | <0.001 | | | |  |  |  |  |  |
| **Abdominal obesity** | | | | | | | | | | | | | | | | | | | | | | | | | | | | | | | | | | | | | | | | | | | | | | | | | | | | | | | | | | | | | | | | | | | | | | |
| Yes | 691,155 | | 28,019 | 3.28 | | | 1.07  (1.06–1.09) | | | <0.001 | - | | | | | | | 202,871 | | | | | 1,518 | | | 0.58 | | | 1.34  (1.26–1.43) | | | | | | <0.001 | | | | | | - | | 179,117 | | | | 3,651 | | | | | | 1.61 | | | | | | 1.14  (1.09–1.18) | | | <0.001 | | | | | - | | | |
| **Regular physical activity** | | | | | | | | | | | | | | | | | | | | | | | | | | | | | | | | | | | | | | | | | | | | | | | | | | | | | | | | | | | | | | | | | | | | | | |
| Yes | 649,643 | | 22,239 | 2.75 | | | 1.11  (1.09–1.13) | | | <0.001 | - | | | | | | | 185,725 | | | | | 871 | | | 0.36 | | | 1.00  (0.93–1.08) | | | | | | 0.937 | | | | | | - | | 163,302 | | | | 2,573 | | | | | 1.23 | | | | | | | 1.05  (1.00–1.09) | | | 0.036 | | | | - | | | | |

|  | | | | **50-64 years old** | | | | | | | | | | | | | | | | | | | | | | | | | | | | | | **≥65 years old** | | | | | | | | | | | | | | | | | | | | | | | |  | | |
| --- | --- | --- | --- | --- | --- | --- | --- | --- | --- | --- | --- | --- | --- | --- | --- | --- | --- | --- | --- | --- | --- | --- | --- | --- | --- | --- | --- | --- | --- | --- | --- | --- | --- | --- | --- | --- | --- | --- | --- | --- | --- | --- | --- | --- | --- | --- | --- | --- | --- | --- | --- | --- | --- | --- | --- | --- | --- | --- | --- | --- |
|  | | | | **N** | | **Events** | | | | | | | **IR** | | | | | | **HR**  **(95% CI)** | | | | | | ***p*** | | ***p*_trend_** | | | | | | **N** | | | | | | **Events** | | | | | | | **IR** | | | **HR**  **(95% CI)** | | | ***p*** | ***p*_trend_** | | | | | ***p*_interaction_** | | |
| **BMI, kg/m2** | | | | | | | | | | | | | | | | | | | | | | | | | | | | | | | | | | | | | | | | | | | | | | | | | | | | | | | | | | | | |
| <18.5 | | | | 15,173 | | 748 | | | | | | | 4.28 | | | | | | 1.11  (1.03–1.20) | | | | | | 0.005 | | |  | | | | | 15,502 | | | | | | 1,586 | | | | | | | | 12.04 | | | 0.97  (0.92–1.02) | | 0.214 | | | |  | <0.001 | | | |
| 18.5-22.9 | | | | 275,189 | | 12,443 | | | | | | | 3.70 | | | | | | (ref) | | | | | | | | |  | | | | | 137,477 | | | | | | 16,660 | | | | | | | | 11.90 | | | (ref) | | | | | |  |  | | | |
| 23.0-24.9 | | | | 261,460 | | 11,628 | | | | | | | 3.60 | | | | | | 0.97  (0.95–1.00) | | | | | | 0.022 | | | <0.001 | | | | | 92,484 | | | | | | 11,214 | | | | | | | | 11.23 | | | 0.96  (0.94–0.98) | | 0.001 | | | | 0.012 |  | | | |
| 25.0-29.9 | | | | 321,096 | | 14,666 | | | | | | | 3.69 | | | | | | 0.97  (0.94–0.99) | | | | | | 0.006 | | |  | | | | | 92,896 | | | | | | 11,511 | | | | | | | | 11.30 | | | 0.96  (0.94–0.99) | | 0.002 | | | |  |  | | | |
| ≥30.0 | | | | 21,551 | | 1,110 | | | | | | | 4.20 | | | | | | 1.02  (0.96–1.09) | | | | | | 0.497 | | |  | | | | | 5,041 | | | | | | 659 | | | | | | | | 12.15 | | | 1.00  (0.93–1.08) | | 0.960 | | | |  |  | | | |
| **Smoking** | | | | | | | | | | | | | | | | | | | | | | | | | | | | | | | | | | | | | | | | | | | | | | | | | | | | | | | | | | | | |
| Non | | | | 280,077 | 11,215 | | | | | | | | | | | 3.22 | | | (ref) | | | | | | | | | <0.001 | | | | | | 147,649 | 17,387 | | | | | | | | 11.01 | | | | | (ref) | | | | | | | | <0.001 | <0.001 | | | |
| Ex | | | | 288,378 | 10,395 | | | | | | | | | | | 2.90 | | | 0.91  (0.89–0.94) | | <0.001 | | | | | | |  | | | | | | 107,091 | 11,736 | | | | | | | | 10.25 | | | | | 0.96  (0.93–0.98) | | | | <0.001 | | | |  |  | | | |
| Current | | | | 326,014 | 18,985 | | | | | | | | | | | 4.82 | | | 1.61  (1.57–1.65) | | <0.001 | | | | | | |  | | | | | | 88,660 | 12,507 | | | | | | | | 14.22 | | | | | 1.40  (1.37–1.43) | | | | <0.001 | | | |  |  | | | |
| **Hypertension** | | | | | | | | | | | | | | | | | | | | | | | | | | | | | | | | | | | | | | | | | | | | | | | | | | | | | | | | | | | | |
| Yes | | | | 306,635 | | | 17,765 | | | | | | | | | 4.76 | | | 1.36  (1.33–1.39) | | <0.001 | | | | | | | - | | | | | | 167,937 | | | | 22,797 | | | | 13.05 | | | | | | | | | 1.23  (1.20–1.25) | <0.001 | | | | - | <0.001 | | | |
| **Diabetes** | | | | | | | | | | | | | | | | | | | | | | | | | | | | | | | | | | | | | | | | | | | | | | | | | | | | | | | | | | | | |
| Normoglycemia | | | | 481,175 | | | | 19,234 | | | | | | | | 3.22 | | (ref) | | | | | | | | | | | | | <0.001 | | | 180,727 | | | | 20,720 | | | 10.80 | | | | | | | | | (ref) | | | | | |  | <0.001 | | | |
| Prediabetes | | | | 276,911 | | | 11,877 | | | | | | | | | 3.48 | | 1.05  (1.02–1.07) | | | | | <0.001 | | | | | | | |  | | | 98,921 | | | | 11,538 | | | 11.00 | | | | | | | | | 1.02  (0.99–1.04) | | 0.182 | | | | <0.001 |  | | | |
| Diabetes | | | | 136,383 | | | 9,484 | | | | | | | | | 5.84 | | 1.60  (1.56–1.64) | | | | | <0.001 | | | | | | | |  | | | 63,752 | | | 9,372 | | | | 14.75 | | | | | | | | | 1.36  (1.33–1.40) | | <0.001 | | | |  |  | | | |
| **Systolic BP, mmHg** | | | | | | | | | | | | | | | | | | | | | | | | | | | | | | | | | | | | | | | | | | | | | | | | | | | | | | | | | | | | |
| <100 | | | | 13,762 | | | | | 441 | | | | | | | | 2.61 | | (ref) | | | | | | | | | | | |  | | | 4,082 | | 353 | | | | 8.72 | | | | | | | | | (ref) | | | | | | |  | <0.001 | | | |
| 100-119 | | | | 249,867 | | | | | 8,736 | | | | | | | | 2.82 | | 1.11  (1.01–1.22) | | | | | 0.030 | | | | | | |  | | | 74,604 | | 7,779 | | | | 9.91 | | | | | | | | | 1.13  (1.02–1.26) | | | 0.022 | | | |  |  | | | |
| 120-139 | | | | 480,542 | | | | | 21,748 | | | | | | | | 3.67 | | 1.41  (1.28–1.55) | | | | | <0.001 | | | | | | | <0.001 | | | 179,142 | | 21,267 | | | | 11.21 | | | | | | | | | 1.27  (1.15–1.42) | | | <0.001 | | | | <0.001 |  | | | |
| 140-159 | | | | 122,277 | | | | | 7,342 | | | | | | | | 4.95 | | 1.79  (1.62–1.97) | | | | | <0.001 | | | | | | |  | | | 66,173 | | 9,038 | | | | 13.11 | | | | | | | | | 1.46  (1.31–1.62) | | | <0.001 | | | |  |  | | | |
| ≥160 | | | | 28,021 | | | | | 2,328 | | | | | | | | 7.05 | | 2.44  (2.20–2.70) | | | | | <0.001 | | | | | |  | | | | 19,399 | | 3,193 | | | | 16.66 | | | | | | | | | 1.79  (1.60–2.00) | | | <0.001 | | | |  |  | | | |
| **Diastolic BP, mmHg** | | | | | | | | | | | | | | | | | | | | | | | | | | | | | | | | | | | | | | | | | | | | | | | | | | | | | | | | | | | | |
| <60 | 9,430 | | | | | | | | | 333 | | | | | | 2.88 | | | | (ref) | | | | | | | | | | | |  | | 5,010 | | | | 549 | | | | | | 11.05 | | | | (ref) | | | | | | | |  | <0.001 | | | |
| 60-69 | 94,511 | | | | | | | | | 3,338 | | | | | | 2.86 | | | | 1.01  (0.90–1.13) | | | | | | 0.873 | | | | | |  | | 42,186 | | | | 4,471 | | | | | | 10.16 | | | | 0.96  (0.88–1.15) | | | | 0.390 | | | |  |  | | | |
| 70-79 | | 287,757 | | | | | | | | | | 11,432 | | | 3.21 | | | | | 1.14  (1.03–1.28) | | | | | | 0.002 | | | | | | <0.001 | | 112,701 | | | | 12,652 | | | | | | | 10.65 | | | 1.03  (0.95–1.23) | | | | 0.491 | | | | <0.001 |  | | | |
| 80-89 | | 362,238 | | | | | | | | | | 17,088 | | | 3.83 | | | | | 1.37  (1.23–1.53) | | | | | | <0.001 | | | | | |  | | 130,483 | | | | 16,287 | | | | | | | 11.83 | | | 1.16  (1.06–1.26) | | | | <0.001 | | | |  |  | | | |
| ≥90 | | 140,533 | | | | | | | | | | 8,404 | | | 4.93 | | | | | 1.73  (1.55–1.93) | | | | | | <0.001 | | | | | |  | | 53,020 | | | | 7,671 | | | | | | | 14.00 | | | 1.36  (1.25–1.49) | | | | <0.001 | | | |  |  | | | |
| **Non-HDL-c, mg/dL** | | | | | | | | | | | | | | | | | | | | | | | | | | | | | | | | | | | | | | | | | | | | | | | | | | | | | | | | | | | | |
| <130 | | | 302,632 | | | | | | | | 12,953 | | | 3.51 | | | | (ref) | | | | | | | | | | |  | | | | | 145,427 | | | 16,435 | | | | | | | 10.97 | | | | (ref) | | | | | |  | | | | <0.001 | |  |
| 130-159 | | | 296,185 | | | | | | | | 13,045 | | | 3.56 | | | | 1.06  (1.04–1.10) | | | | <0.001 | | | | | | |  | | | | | 6,457 | | | 13,307 | | | | | | | 11.38 | | | | 1.07  (1.05–1.09) | | | | <0.001 | |  | | | |  | |  |
| 160-189 | | | 195,842 | | | | | | | | 9,317 | | | 3.85 | | | | 1.17  (1.13–1.20) | | | | <0.001 | | | | | | | <0.001 | | | | | 3,773 | | | 8,057 | | | | | | | 12.26 | | | | 1.16  (1.13–1.19) | | | | <0.001 | | <0.001 | | | |  | |  |
| 190-219 | | | 74,309 | | | | | | | | 3,814 | | | 4.17 | | | | 1.25  (1.21–1.30) | | | | <0.001 | | | | | | |  | | | | | 1,385 | | | 2,860 | | | | | | | 13.42 | | | | 1.27  (1.22–1.32) | | | | <0.001 | |  | | | |  | |  |
| ≥220 | | | 25,501 | | | | | | | | 1,466 | | | 4.71 | | | | 1.38  (1.30–1.45) | | | | <0.001 | | | | | | |  | | | | | 481 | | | 971 | | | | | | | 14.70 | | | | 1.37  (1.29–1.47) | | | | <0.001 | |  | | | |  | |  |
| **LDL-c, mg/dL** | | | | | | | | | | | | | | | | | | | | | | | | | | | | | | | | | | | | | | | | | | | | | | | | | | | | | | | | | | | | |
| <70 | | | 74,547 | | | | | | | | 3,741 | | | 4.17 | | | | (ref) | | | | | | | | | | |  | | | | | 33,637 | | | 3,992 | | | | | | | 11.90 | | | | (ref) | | | | | |  | | | | <0.001 | |  |
| 70-99 | | | 216,649 | | | | | | | | 9,544 | | | 3.59 | | | | 0.96  (0.92–0.99) | | | | 0.017 | | | | | | |  | | | | | 94,284 | | | 10,929 | | | | | | | 11.17 | | | | 0.97  (0.94–1.01) | | | | 0.111 | |  | | | |  | |  |
| 100-129 | | | 315,530 | | | | | | | | 13,828 | | | 3.54 | | | | 1.00  (0.96–1.03) | | | | 0.866 | | | | | | | <0.001 | | | | | 120,884 | | | 14,542 | | | | | | | 11.35 | | | | 1.02  (0.98–1.06) | | | | 0.347 | | <0.001 | | | |  | |  |
| 130-159 | | | 202,483 | | | | | | | | 9,259 | | | 3.70 | | | | 1.06  (1.02–1.10) | | | | 0.003 | | | | | | |  | | | | | 68,623 | | | 8,579 | | | | | | | 11.70 | | | | 1.07  (1.03–1.11) | | | | 0.001 | |  | | | |  | |  |
| ≥160 | | | 85,260 | | | | | | | | 4,223 | | | 4.02 | | | | 1.15  (1.10–1.21) | | | | <0.001 | | | | | | |  | | | | | 25,972 | | | 3,588 | | | | | | | 13.11 | | | | 1.20  (1.14–1.25) | | | | <0.001 | |  | | | |  | |  |
| **Triglycerides, mg/dL** | | | | | | | | | | | | | | | | | | | | | | | | | | | | | | | | | | | | | | | | | | | | | | | | | | | | | | | | | | | | |
| <100 | | | 283,754 | | | | | | | | 11,027 | | | 3.16 | | | | (ref) | | | | | | | | | | | <0.001 | | | | | 133,455 | | | 14,511 | | | | | | | 10.39 | | | | (ref) | | | | | | <0.001 | | | | <0.001 | |  |
| 100-149 | | | 261,520 | | | | | | | | 11,753 | | | 3.65 | | | | 1.11  (1.08–1.14) | | | | <0.001 | | | | | | |  |  |  |  |  | 106,480 | | | 13,023 | | | | | | | 11.68 | | | | 1.10  (1.07–1.12) | | | | <0.001 | |  |  |  |  |  | |  |
| 150-199 | | | 156,514 | | | | | | | | 7,688 | | | 3.99 | | | | 1.18  (1.15–1.22) | | | | <0.001 | | | | | | |  |  |  |  |  | 53,116 | | | 7,041 | | | | | | | 12.58 | | | | 1.18  (1.14–1.21) | | | | <0.001 | |  |  |  |  |  | |  |
| ≥200 | | | 192,681 | | | | | | | | 10,127 | | | 4.28 | | | | 1.23  (1.19–1.26) | | | | <0.001 | | | | | | |  |  |  |  |  | 50,349 | | | 7,055 | | | | | | | 13.28 | | | | 1.24  (1.20–1.27) | | | | <0.001 | |  |  |  |  |  | |  |
| **Abdominal obesity** | | | | | | | | | | | | | | | | | | | | | | | | | | | | | | | | | | | | | | | | | | | | | | | | | | | | | | | | | | | | |
| Yes | | | 220,610 | | | | | | | | | 11,258 | | | 4.16 | | | 1.05  (1.03–1.07) | | | | <0.001 | | | | | | | - | | | | | 88,557 | | | 11,592 | | | | | | | 12.30 | | | | 1.04  (1.01–1.06) | | | | 0.002 | | | - | | | <0.001 |  |  |
| **Regular physical activity** | | | | | | | | | | | | | | | | | | | | | | | | | | | | | | | | | | | | | | | | | | | | | | | | | | | | | | | | | | | | |
| Yes | | | 210,912 | | | | | | | | | 8,990 | | | 3.45 | | | 1.11  (1.08–1.13) | | | | <0.001 | | | | | | | - | | | | | 89,704 | | | 9,805 | | | | | | | 9.99 | | | | 1.16  (1.13–1.18) | | | | <0.001 | | - | | | | <0.001 |  |  |

1. **Women**

|  | **Total** | | | | | | | | | | | | | | | | | | | **<40 years old** | | | | | | | | | | | | | | | | | | | | | | | | | | | | | | **40-49 years old** | | | | | | | | | | | | | | | | | | | | | | | | | | | |
| --- | --- | --- | --- | --- | --- | --- | --- | --- | --- | --- | --- | --- | --- | --- | --- | --- | --- | --- | --- | --- | --- | --- | --- | --- | --- | --- | --- | --- | --- | --- | --- | --- | --- | --- | --- | --- | --- | --- | --- | --- | --- | --- | --- | --- | --- | --- | --- | --- | --- | --- | --- | --- | --- | --- | --- | --- | --- | --- | --- | --- | --- | --- | --- | --- | --- | --- | --- | --- | --- | --- | --- | --- | --- | --- | --- | --- | --- |
|  | **N** | **Events** | | | | **IR** | | **HR**  **(95% CI)** | | | ***p*** | | | | | | | ***p*_trend_** | | **N** | | **Events** | | | | | | | | | | **IR** | | | | **HR**  **(95% CI)** | | | | | | | ***p*** | | | | | | ***p*_trend_** | **N** | | | | | **Events** | | | | **IR** | | | | | **HR**  **(95% CI)** | | | | ***p*** | | | | | ***p*_trend_** | | | | |
| **BMI, kg/m2** | | | | | | | | | | | | | | | | | | | | | | | | | | | | | | | | | | | | | | | | | | | | | | | | | | | | | | | | | | | | | | | | | | | | | | | | | | | | | |
| <18.5 | 166,545 | | 2,175 | | | 1.04 | | 1.01  (0.96–1.05) | | | 0.739 | | | | | | |  | | 107,261 | | 225 | | | | | | | | | | 0.16 | | | | 1.09  (0.94–1.26) | | | | | | | 0.239 | | | | |  | | 29,043 | | | | | 188 | | | | 0.51 | | | | | 0.90  (0.78–1.05) | | | | 0.176 | | | | |  | | | | |
| 18.5-22.9 | 1,399,376 | | 26,467 | | | 1.50 | | (ref) | | | | | |  | | | | | | 456,301 | | 1,016 | | | | | | | | | | 0.17 | | | | (ref) | | | | | | | | | | | |  | | 442,392 | | | | | 3,441 | | | | 0.61 | | | | | (ref) | | | | | | | | |  | | | | |
| 23.0-24.9 | 648,266 | | 19,732 | | | 2.42 | | 1.04  (1.03–1.06) | | | <0.001 | | | | | <0.001 | | | | 85,658 | | 262 | | | | | | | | | | 0.24 | | | | 1.17  (1.02–1.34) | | | | | | | 0.027 | | | | | <0.001 | | 193,275 | | | | | 2,002 | | | | 0.81 | | | | | 1.21  (1.14–1.28) | | | | <0.001 | | | | | <0.001 | | | | |
| 25.0-29.9 | 677,902 | | 25,805 | | | 3.04 | | 1.08  (1.06–1.10) | | | <0.001 | | | | |  | | | | 67,924 | | 285 | | | | | | | | | | 0.33 | | | | 1.43  (1.25–1.64) | | | | | | | <0.001 | | | | |  | | 172,950 | | | | | 2,077 | | | | 0.94 | | | | | 1.28  (1.20–1.35) | | | | <0.001 | | | | |  | | | | |
| ≥30.0 | 95,968 | | 3,731 | | | 3.12 | | 1.17  (1.13–1.21) | | | <0.001 | | | | |  | | | | 15,653 | | 120 | | | | | | | | | | 0.60 | | | | 2.16  (1.76–2.65) | | | | | | | <0.001 | | | | |  | | 25,867 | | | | | 380 | | | | 1.16 | | | | | 1.35  (1.21–1.50) | | | | <0.001 | | | | |  | | | | |
| **Smoking** | | | | | | | | | | | | | | | | | | | | | | | | | | | | | | | | | | | | | | | | | | | | | | | | | | | | | | | | | | | | | | | | | | | | | | | | | | | | | |
| Non | 2,826,772 | | 73,601 | | | 2.07 | | (ref) | | | | | | |  | | | | | 671,770 | | 1,727 | | | | | | | | | | 0.20 | | | | (ref) | | | | | | | | | | | |  | | 816,789 | | | | | 7,442 | | | | 0.71 | | | | | (ref) | | | | | | | | |  | | | | |
| Ex | 55,264 | | 1,066 | | | 1.53 | | 1.13  (1.07–1.20) | | | <0.001 | | | | | <0.001 | | | | 237,94 | | 59 | | | | | | | | | | 0.19 | | | | 1.00  (0.77–1.30) | | | | | | 0.999 | | | | | | <0.001 | | 15,479 | | | | | 158 | | | | 0.80 | | | | | 1.21  (1.03–1.42) | | | | 0.019 | | | | | <0.001 | | | | |
| Current | 106,021 | | 3,243 | | | 2.46 | | 1.62  (1.57–1.68) | | | <0.001 | | | | |  | | | | 372,33 | | 122 | | | | | | | | | | 0.26 | | | | 1.32  (1.09–1.59) | | | | | | 0.005 | | | | | |  | | 31,259 | | | | | 488 | | | | 1.24 | | | | | 1.72  (1.56–1.89) | | | | <0.001 | | | | |  | | | | |
| **Hypertension** | | | | | | | | | | | | | | | | | | | | | | | | | | | | | | | | | | | | | | | | | | | | | | | | | | | | | | | | | | | | | | | | | | | | | | | | | | | | | |
| Yes | 614,190 | | 37,763 | | | 5.05 | | 1.33  (1.31–1.35) | | | <0.001 | | | | | | - | | | 16,487 | | | | | | | 157 | | | | | 0.75 | | | | 2.34  (1.97–2.78) | | | | | | <0.001 | | | | | | - | | 92,169 | | | | | 1,662 | | | | 1.42 | | | | | 1.75  (1.65–1.85) | | | | <0.001 | | | | | - | | | | |
| **Diabetes** | | | | | | | | | | | | | | | | | | | | | | | | | | | | | | | | | | | | | | | | | | | | | | | | | | | | | | | | | | | | | | | | | | | | | | | | | | | | | |
| Normo-glycemia | 2,237,640 | | 46,052 | | | 1.62 | | (ref) | | | | | | | | |  | | | 651,621 | | | | | | | | 1,557 | | | | 0.19 | | | | (ref) | | | | | | | | | | | |  | | 678,705 | | | | | 5,916 | | | | 0.68 | | | | | (ref) | | | | | | | | |  | | | | |
| Prediabetes | 562,623 | | 18,059 | | | 2.56 | | 1.01  (0.99–1.03) | | | 0.186 | | | | | | <0.001 | | | 74,195 | | | | | | | 276 | | | | | 0.29 | | | | 1.25  (1.10–1.42) | | | | | | <0.001 | | | | | | <0.001 | | 157,013 | | | | | 1,557 | | | | 0.78 | | | | | 1.01  (0.95–1.07) | | | | 0.806 | | | | | <0.001 | | | | |
| Diabetes | 187,794 | | 13,799 | | | 6.15 | | 1.49  (1.46–1.52) | | | <0.001 | | | | | |  | | | 6,981 | | | | | | | 75 | | | | | 0.84 | | | | 2.47  (1.94–3.14) | | | | | | <0.001 | | | | | |  | | 27,809 | | | | | 615 | | | | 1.75 | | | | | 1.86  (1.71–2.03) | | | | <0.001 | | | | |  | | | | |
| **Systolic BP, mmHg** | | | | | | | | | | | | | | | | | | | | | | | | | | | | | | | | | | | | | | | | | | | | | | | | | | | | | | | | | | | | | | | | | | | | | | | | | | | | | |
| <100 | 175,958 | | 1,544 | | | 0.69 | | (ref) | | | | | | | | |  | | | 74,455 | | | | | | | | 137 | | | | 0.14 | | | | (ref) | | | | | | | | | | | |  | | 60,047 | | | | | 380 | | | | 0.49 | | | | | (ref) | | | | | | | | |  | | | | |
| 100-119 | 1,341,005 | | 19,616 | | | 1.15 | | 1.16  (1.10–1.22) | | | <0.001 | | | | | |  | | | 460,257 | | | | | | | | 1,040 | | | | 0.18 | | | | 1.18  (0.98–1.41) | | | | | | 0.075 | | | | | |  | | 435,333 | | | | | 3,319 | | | | 0.60 | | | | | 1.14  (1.03–1.27) | | | | 0.013 | | | | |  | | | | |
| 120-139 | 1,184,601 | | 38,403 | | | 2.59 | | 1.39  (1.32–1.47) | | | <0.001 | | | | | | <0.001 | | | 189,626 | | | | | | | | 623 | | | | 0.26 | | | | 1.47  (1.22–1.78) | | | | | | <0.001 | | | | | | <0.001 | | 320,210 | | | | | 3,504 | | | | 0.86 | | | | | 1.49  (1.34–1.66) | | | | <0.001 | | | | | <0.001 | | | | |
| 140-159 | 230,960 | | 13,747 | | | 4.88 | | 1.61  (1.53–1.70) | | | <0.001 | | | | | |  | | | 7,185 | | | | | | | | 70 | | | | 0.76 | | | | 2.96  (2.19–3.99) | | | | | | <0.001 | | | | | |  | | 39,805 | | | | | 658 | | | | 1.30 | | | | | 2.01  (1.77–2.29) | | | | <0.001 | | | | |  | | | | |
| ≥160 | 55,533 | | 4,600 | | | 6.98 | | 1.93  (1.82–2.05) | | | <0.001 | | | | | |  | | | 1,274 | | | | | | | | 38 | | | | 2.36 | | | | 7.65  (5.27–11.11) | | | | | | <0.001 | | | | | |  | | 8,132 | | | | | 227 | | | | 2.21 | | | | | 3.31  (2.80–3.91) | | | | <0.001 | | | | |  | | | | |
| **Diastolic BP, mmHg** | | | | | | | | | | | | | | | | | | | | | | | | | | | | | | | | | | | | | | | | | | | | | | | | | | | | | | | | | | | | | | | | | | | | | | | | | | | | | |
| <60 | 112,872 | | 1,254 | 0.87 | | | | (ref) | | | | | | | | | | |  | 43,083 | | | | | | | | 60 | | | | 0.11 | | | | (ref) | | | | | | | | | | | |  | | 9,772 | | | | | | 260 | | | | | | 0.52 | | | | | (ref) | | | | | | | |  | | |
| 60-69 | 716,136 | | 10,277 | 1.13 | | | | 1.07  (1.01–1.14) | | | 0.017 | | | | | | | |  | 248,118 | | | | | | | | 522 | | | | 0.16 | | | | 1.48  (1.13–1.93) | | | | | | 0.004 | | | | | |  | | 10,1778 | | | | | | 1,625 | | | | | | 0.54 | | | | | 1.01  (0.89–1.15) | | | | | 0.862 | | |  | | |
| 70-79 | 1,119,967 | | 25,349 | 1.79 | | | | 1.25  (1.18–1.32) | | | <0.001 | | | | | | | | <0.001 | 300,421 | | | | | | | | 765 | | | | 0.20 | | | | 1.69  (1.30–2.20) | | | | | | <0.001 | | | | | | 0.008 | | 292,708 | | | | | | 2,844 | | | | | | 0.68 | | | | | 1.19  (1.05–1.35) | | | | | 0.007 | | | <0.001 | | |
| 80-89 | 811,839 | | 28,500 | | 2.81 | | | 1.42  (1.35–1.51) | | | <0.001 | | | | | | | |  | 129,439 | | | | | | | | 440 | | | | 0.26 | | | | 1.99  (1.51–2.61) | | | | | | <0.001 | | | | | |  | | 340,411 | | | | | | 2,453 | | | | | | | 0.90 | | | | 1.47  (1.29–1.67) | | | | | <0.001 | | |  | | |
| ≥90 | 227,243 | | 12,530 | | 4.50 | | | 1.70  (1.60–1.80) | | | <0.001 | | | | | | | |  | 11,736 | | | | | | | | 121 | | | | 0.81 | | | | 4.34  (3.16–5.97) | | | | | | <0.001 | | | | | |  | | 109,651 | | | | | | 906 | | | | | | | 1.42 | | | | 2.12  (1.85–2.44) | | | | | <0.001 | | |  | | |
| **Non-HDL-c, mg/dL** | | | | | | | | | | | | | | | | | | | | | | | | | | | | | | | | | | | | | | | | | | | | | | | | | | | | | | | | | | | | | | | | | | | | | | | | | | | | | |
| <130 | 1,351,248 | | 22,734 | | 1.33 | | (ref) | | | | | | | |  | | | | | 522,449 | | | | | 1,192 | | | | | | 0.18 | | | (ref) | | | | | | | | | | |  | | | | | 430,262 | | 3,534 | | | | | | 0.64 | | | | | | | | (ref) | | | | | | | | | |  | |
| 130-159 | 864,776 | | 24,353 | | 2.24 | | 1.07  (1.05–1.09) | | | | <0.001 | | | |  | | | | | 151,944 | | | | | 457 | | | | | | 0.23 | | | 1.08  (0.97–1.20) | | | | | | | 0.181 | | | |  | | | | | 266,540 | | 2,541 | | | | | | 0.75 | | | | | | | | 1.05  (1.00–1.11) | | | | | 0.044 | | | | |  | |
| 160-189 | 499,740 | | 18,377 | | 2.94 | | 1.10  (1.08–1.12) | | | | <0.001 | | | | <0.001 | | | | | 44,082 | | | 184 | | | | | | | 0.33 | | | 1.26  (1.07–1.48) | | | | | | 0.005 | | | | | 0.045 | | | | | | 119,919 | | | 1,340 | | | | 0.87 | | | | | | | | 1.13  (1.06–1.21) | | | | <0.001 | | | | | <0.001 | | | |
| 190-219 | 195,798 | | 8,554 | | 3.52 | | 1.15  (1.13–1.18) | | | | <0.001 | | | |  | | | | | 10,652 | | | 55 | | | | | | | 0.40 | | | 1.33  (1.01–1.75) | | | | | | 0.045 | | | | |  | | | | | | 35,573 | | | 472 | | | | 1.04 | | | | | | | | 1.24  (1.12–1.36) | | | | <0.001 | | | | |  | | | |
| ≥220 | 76,495 | | 3,892 | | 4.12 | | 1.26  (1.22–1.31) | | | | <0.001 | | | |  | | | | | 3,670 | | | 20 | | | | | | | 0.42 | | | 1.34  (0.86–2.10) | | | | | | 0.200 | | | | |  | | | | | | 11,233 | | | 201 | | | | 1.41 | | | | | | | | 1.53  (1.33–1.77) | | | | <0.001 | | | | |  | | | |
| **LDL-c, mg/dL** | | | | | | | | | | | | | | | | | | | | | | | | | | | | | | | | | | | | | | | | | | | | | | | | | | | | | | | | | | | | | | | | | | | | | | | | | | | | | |
| <70 | 197,068 | | 4,167 | | | 1.68 | | | | (ref) | | | | | | | |  | | | 84,138 | | | | | 214 | | | | | 0.20 | | | | (ref) | | | | | | | | | | | | | 0.546 | | | 52,888 | | | 532 | | | | | | | | 0.79 | | | | | (ref) | | | | | | | |  | | |
| 70-99 | 820,432 | | 15,648 | | | 1.51 | | | | 0.96  (0.93–0.99) | | 0.020 | | | | | |  | | | 308,718 | | | | | 717 | | | | | 0.18 | | | | 0.88  (0.75–1.02) | | | | | | 0.095 | | | | | | |  |  |  | 252,255 | | | 2,158 | | | | | | | | 0.67 | | | | | 0.89  (0.81–0.98) | | | 0.019 | | | | |  | | |
| 100-129 | 1,046,503 | | 25,798 | | | 1.96 | | | | 0.99  (0.96–1.02) | | 0.478 | | | | | | <0.001 | | | 245,916 | | | | | 651 | | | | | 0.21 | | | | 0.89  (0.76–1.04) | | | | | | 0.140 | | | | | | |  |  |  | 333,502 | | | 2,908 | | | | | | | | 0.68 | | | | | 0.87  (0.79–0.96) | | | 0.003 | | | | | 0.003 | | |
| 130-159 | 622,225 | | 20,258 | | | 2.60 | | | | 1.02  (0.98–1.05) | | 0.380 | | | | | |  | | | 75,250 | | | | | 245 | | | | | 0.25 | | | | 0.94  (0.78–1.13) | | | | | | 0.484 | | | | | | |  |  |  | 166,520 | | | 1,757 | | | | | | | | 0.83 | | | | | 0.98  (0.89–1.08) | | | 0.626 | | | | |  | | |
| ≥160 | 301,829 | | 12,039 | | | 3.20 | | | | 1.06  (1.02–1.09) | | 0.003 | | | | | |  | | | 18,775 | | | | | 81 | | | | | 0.34 | | | | 1.06  (0.82–1.37) | | | | | | 0.672 | | | | | | |  |  |  | 58,362 | | | 733 | | | | | | | | 0.98 | | | | | 1.07  (0.95–1.19) | | | 0.271 | | | | |  | | |
| **Triglycerides, mg/dL** | | | | | | | | | | | | | | | | | | | | | | | | | | | | | | | | | | | | | | | | | | | | | | | | | | | | | | | | | | | | | | | | | | | | | | | | | | | | | |
| <100 | 1,676,753 | | 27,344 | | | 1.28 | | | | (ref) | | | <0.001 | | | | | | | | 566,268 | | | | | 1,282 | | | | | 0.18 | | | | (ref) | | | | | | | | | | | | 0.002 | | | | 541,019 | | | 4,358 | | | | | | | | 0.63 | | | | | (ref) | | | | | | | | <0.001 | | |
| 100-149 | 749,814 | | 24,646 | | | 2.63 | | | | 1.10  (1.08–1.12) | | <0.001 |  |  |  |  |  |  |  |  | 114,564 | | | | | 394 | | | | | 0.27 | | | | 1.22  (1.09–1.37) | | | | | <0.001 | | | | | | |  |  |  |  | 201,875 | | | 2,071 | | | | | | | | 0.80 | | | | | 1.11  (1.06–1.18) | | | <0.001 | | | | |  |  |  |
| 150-199 | 309,293 | | 13,129 | | | 3.42 | | | | 1.16  (1.13–1.18) | | <0.001 |  |  |  |  |  |  |  |  | 31,110 | | | | | 121 | | | | | 0.30 | | | | 1.17  (0.96–1.42) | | | | | 0.116 | | | | | | |  |  |  |  | 69,609 | | | 839 | | | | | | | | 0.94 | | | | | 1.19  (1.10–1.28) | | | <0.001 | | | | |  |  |  |
| ≥200 | 252,197 | | 12,791 | | | 4.11 | | | | 1.27  (1.24–1.30) | | <0.001 |  |  |  |  |  |  |  |  | 20,855 | | | | | 111 | | | | | 0.41 | | | | 1.38  (1.12–1.70) | | | | | 0.002 | | | | | | |  |  |  |  | 51,024 | | | 820 | | | | | | | | 1.26 | | | | | 1.43  (1.32–1.55) | | | <0.001 | | | | |  |  |  |
| **Abdominal obesity** | | | | | | | | | | | | | | | | | | | | | | | | | | | | | | | | | | | | | | | | | | | | | | | | | | | | | | | | | | | | | | | | | | | | | | | | | | | | | |
| Yes | 485,130 | | 24,361 | | | 4.07 | | | 1.11  (1.09–1.12) | | | <0.001 | - | | | | | | | | 43,127 | | | 240 | | | | | | 0.43 | | | | | 1.58  (1.37–1.82) | | | <0.001 | | | | | | | | - | | | | | 93,244 | | | 1,279 | | | | | | | 1.08 | | | | | | 1.22  (1.15–1.30) | | | <0.001 | | | | | | | - |
| **Regular physical activity** | | | | | | | | | | | | | | | | | | | | | | | | | | | | | | | | | | | | | | | | | | | | | | | | | | | | | | | | | | | | | | | | | | | | | | | | | | | | | |
| Yes | 471,637 | | 11,618 | | 1.95 | | | | 1.14  (1.12–1.16) | | | <0.001 | - | | | | | | | | 68,411 | | 196 | | | | | | 0.22 | | | | | | 0.96  (0.83–1.12) | | 0.609 | | | | | | | | - | | | | | | 141,448 | | | 1,302 | | | | | | 0.72 | | | | | | | 1.05  (0.99–1.11) | | | 0.140 | | | | | - | | |

|  | | | | **50-64 years old** | | | | | | | | | | | | | | | | | | | | | | | | | | | | | | **≥65 years old** | | | | | | | | | | | | | | | | | | | | | | | | | |  | | |
| --- | --- | --- | --- | --- | --- | --- | --- | --- | --- | --- | --- | --- | --- | --- | --- | --- | --- | --- | --- | --- | --- | --- | --- | --- | --- | --- | --- | --- | --- | --- | --- | --- | --- | --- | --- | --- | --- | --- | --- | --- | --- | --- | --- | --- | --- | --- | --- | --- | --- | --- | --- | --- | --- | --- | --- | --- | --- | --- | --- | --- | --- | --- |
|  | | | | **N** | | **Events** | | | | | | | **IR** | | | | | | **HR**  **(95% CI)** | | | | | | | ***p*** | ***p*_trend_** | | | | | | **N** | | | | | | **Events** | | | | | | | | **IR** | | | **HR**  **(95% CI)** | | | ***p*** | ***p*_trend_** | | | | | | ***p*_interaction_** | | |
| **BMI, kg/m2** | | | | | | | | | | | | | | | | | | | | | | | | | | | | | | | | | | | | | | | | | | | | | | | | | | | | | | | | | | | | | | |
| <18.5 | | | | 16,895 | | 376 | | | | | | | 1.77 | | | | | | 1.02  (0.92–1.13) | | | | | | | 0.769 | |  | | | | | 13,346 | | | | | | 1,386 | | | | | | | | | 10.40 | | | 1.02  (0.96–1.07) | | 0.594 | | | |  | <0.001 | | | | |
| 18.5-22.9 | | | | 371,542 | | 8,467 | | | | | | | 1.80 | | | | | | (ref) | | | | | | | | |  | | | | | 129,141 | | | | | | 13,543 | | | | | | | | | 9.29 | | | (ref) | |  | | | |  |  | | | | |
| 23.0-24.9 | | | | 269,396 | | 7,308 | | | | | | | 2.14 | | | | | | 1.07  (1.04–1.11) | | | | | | | <0.001 | | <0.001 | | | | | 99,937 | | | | | | 10,160 | | | | | | | | | 8.73 | | | 0.96  (0.94–0.99) | | 0.005 | | | | 0.004 |  | | | | |
| 25.0-29.9 | | | | 303,745 | | 9,680 | | | | | | | 2.52 | | | | | | 1.14  (1.10–1.17) | | | | | | | <0.001 | |  | | | | | 133,283 | | | | | | 13,763 | | | | | | | | | 8.82 | | | 0.96  (0.94–0.99 | | 0.003 | | | |  |  | | | | |
| ≥30.0 | | | | 38,253 | | 1,484 | | | | | | | 3.09 | | | | | | 1.24  (1.18–1.32) | | | | | | | <0.001 | |  | | | | | 16,195 | | | | | | 1,747 | | | | | | | | | 9.32 | | | 0.98  (0.83–1.03) | | 0.444 | | | |  |  | | | | |
| **Smoking** | | | | | | | | | | | | | | | | | | | | | | | | | | | | | | | | | | | | | | | | | | | | | | | | | | | | | | | | | | | | | | |
| Non | | | | 960,054 | 25,769 | | | | | | | | | | | 2.12 | | | (ref) | | | | | | | | |  | | | | | | 378,159 | 38,663 | | | | | | | | 8.87 | | | | | | (ref) | | | | | | | |  | 0.029 | | | | |
| Ex | | | | 11,883 | 342 | | | | | | | | | | | 2.28 | | | 1.13  (1.02–1.26) | | 0.021 | | | | | | | <0.001 | | | | | | 4,108 | 507 | | | | | | | | 11.41 | | | | | | 1.16  (1.06–1.26) | | | | 0.001 | | | | <0.001 |  | | | | |
| Current | | | | 27,894 | 1,204 | | | | | | | | | | | 3.48 | | | 1.73  (1.63–1.83) | | <0.001 | | | | | | |  | | | | | | 9,635 | 1,429 | | | | | | | | 14.38 | | | | | | 1.53  (1.45–1.61) | | | | <0.001 | | | |  |  | | | | |
| **Hypertension** | | | | | | | | | | | | | | | | | | | | | | | | | | | | | | | | | | | | | | | | | | | | | | | | | | | | | | | | | | | | | | |
| Yes | | | | 293,681 | | | 11,373 | | | | | | | | | 3.08 | | | 1.39  (1.35–1.42) | | <0.001 | | | | | | | - | | | | | | 211,853 | | | | 24,571 | | | | 10.23 | | | | | | | | | | 1.23  (1.20–1.25) | <0.001 | | | | - | <0.001 | | | | |
| **Diabetes** | | | | | | | | | | | | | | | | | | | | | | | | | | | | | | | | | | | | | | | | | | | | | | | | | | | | | | | | | | | | | | |
| Normoglycemia | | | | 680,305 | | | | 16,560 | | | | | | | | 1.92 | | (ref) | | | | | | | | | | | | |  | | | 227,009 | | | | 22,019 | | | 8.34 | | | | | | | | | | (ref) | | | | | |  | <0.001 | | | | |
| Prediabetes | | | | 232,129 | | | 6,346 | | | | | | | | | 2.16 | | 1.01  (0.98–1.04) | | | | | 0.493 | | | | | | | | <0.001 | | | 99,286 | | | | 9,880 | | | 8.64 | | | | | | | | | | 1.00  (0.97–1.02) | | 0.779 | | | | <0.001 |  | | | | |
| Diabetes | | | | 87,397 | | | 4,409 | | | | | | | | | 4.06 | | 1.62  (1.57–1.68) | | | | | <0.001 | | | | | | | |  | | | 65,607 | | | 8,700 | | | | 12.11 | | | | | | | | | | 1.38  (1.35–1.42) | | <0.001 | | | |  |  | | | | |
| **Systolic BP, mmHg** | | | | | | | | | | | | | | | | | | | | | | | | | | | | | | | | | | | | | | | | | | | | | | | | | | | | | | | | | | | | | | |
| <100 | | | | 36,711 | | | | | 647 | | | | | | | | 1.39 | | (ref) | | | | | | | | | | | |  | | | 4,745 | | 380 | | | | 6.93 | | | | | | | | | | (ref) | | | | | | |  | <0.001 | | | | |
| 100-119 | | | | 360,007 | | | | | 7,764 | | | | | | | | 1.70 | | 1.12  (1.04–1.22) | | | | | 0.005 | | | | | | |  | | | 85,408 | | 7,493 | | | | 7.54 | | | | | | | | | | 1.05  (0.95–1.17) | | | 0.319 | | | |  |  | | | | |
| 120-139 | | | | 472,610 | | | | | 13,510 | | | | | | | | 2.26 | | 1.33  (1.23–1.44) | | | | | <0.001 | | | | | | | <0.001 | | | 202,155 | | 20,766 | | | | 8.91 | | | | | | | | | | 1.20  (1.09–1.33) | | | <0.001 | | | | <0.001 |  | | | | |
| 140-159 | | | | 107,665 | | | | | 4,232 | | | | | | | | 3.13 | | 1.66  (1.53–1.81) | | | | | <0.001 | | | | | | |  | | | 76,305 | | 8,787 | | | | 10.13 | | | | | | | | | | 1.33  (1.20–1.47) | | | <0.001 | | | |  |  | | | | |
| ≥160 | | | | 22,838 | | | | | 1,162 | | | | | | | | 4.09 | | 2.09  (1.89–2.30) | | | | | <0.001 | | | | | |  | | | | 23,289 | | 3,173 | | | | 12.38 | | | | | | | | | | 1.55  (1.39–1.72) | | | <0.001 | | | |  |  | | | | |
| **Diastolic BP, mmHg** | | | | | | | | | | | | | | | | | | | | | | | | | | | | | | | | | | | | | | | | | | | | | | | | | | | | | | | | | | | | | | |
| <60 | 24,975 | | | | | | | | | 478 | | | | | | 1.51 | | | (ref) | | | | | | | | | | | | |  | | 5,642 | | | | 456 | | | | | | 7.02 | | | | | (ref) | | | | | | | |  | <0.001 | | | | |
| 60-69 | 183,820 | | | | | | | | | 3,771 | | | | | | 1.62 | | | 1.03  (0.93–1.13) | | | | | | 0.605 | | | | | | |  | | 50,678 | | | | 4,359 | | | | | | 7.41 | | | | | 1.07  (0.97–1.18) | | | | 0.186 | | | |  |  | | | | |
| 70-79 | | 361,493 | | | | | | | | | | 9,183 | | | 2.00 | | | | | 1.19  (1.09–1.30) | | | | | <0.001 | | | | | | | <0.001 | | 130,281 | | | | 12,557 | | | | | | | | 8.32 | | | 1.20  (1.09–1.32) | | | | <0.001 | | | | <0.001 |  | | | | |
| 80-89 | | 323,773 | | | | | | | | | | 9,778 | | | 2.39 | | | | | 1.34  (1.23–1.47) | | | | | <0.001 | | | | | | |  | | 145,613 | | | | 15,829 | | | | | | | | 9.49 | | | 1.35  (1.23–1.48) | | | | <0.001 | | | |  |  | | | | |
| ≥90 | | 105,770 | | | | | | | | | | 4,105 | | | 3.09 | | | | | 1.65  (1.50–1.82) | | | | | <0.001 | | | | | | |  | | 59,688 | | | | 7,398 | | | | | | | | 11.01 | | | 1.53  (1.40–1.69) | | | | <0.001 | | | |  |  | | | | |
| **Non-HDL-c, mg/dL** | | | | | | | | | | | | | | | | | | | | | | | | | | | | | | | | | | | | | | | | | | | | | | | | | | | | | | | | | | | | | | |
| <130 | | | 288,643 | | | | | | | | 7,207 | | | 1.97 | | | | (ref) | | | | | | | | | | |  | | | | | 109,894 | | | 10,801 | | | | | | | | 8.63 | | | | (ref) | | | | | |  | | | <0.001 | | | |  |
| 130-159 | | | 322,271 | | | | | | | | 8,605 | | | 2.11 | | | | 1.06  (1.03–1.09) | | | | <0.001 | | | | | | |  | | | | | 124,021 | | | 12,750 | | | | | | | | 8.91 | | | | 1.06  (1.03–1.08) | | | | <0.001 | |  | | |  | | | |  |
| 160-189 | | | 239,246 | | | | | | | | 6,750 | | | 2.23 | | | | 1.08  (1.04–1.12) | | | | <0.001 | | | | | | | <0.001 | | | | | 96,493 | | | 10,103 | | | | | | | | 9.07 | | | | 1.08  (1.05–1.11) | | | | <0.001 | | <0.001 | | |  | | | |  |
| 190-219 | | | 106,139 | | | | | | | | 3,278 | | | 2.44 | | | | 1.14  (1.09–1.19) | | | | <0.001 | | | | | | |  | | | | | 43,434 | | | 4,749 | | | | | | | | 9.50 | | | | 1.13  (1.09–1.17) | | | | <0.001 | |  | | |  | | | |  |
| ≥220 | | | 43,532 | | | | | | | | 1,475 | | | 2.69 | | | | 1.21  (1.14–1.27) | | | | <0.001 | | | | | | |  | | | | | 18,060 | | | 2,196 | | | | | | | | 10.69 | | | | 1.25  (1.19–1.31) | | | | <0.001 | |  | | |  | | | |  |
| **LDL-c, mg/dL** | | | | | | | | | | | | | | | | | | | | | | | | | | | | | | | | | | | | | | | | | | | | | | | | | | | | | | | | | | | | | | |
| <70 | | | 39,592 | | | | | | | | 1,286 | | | 2.58 | | | | (ref) | | | | | | | | | | |  | | | | | 20,450 | | | 2,135 | | | | | | | | 9.29 | | | | (ref) | | | | | |  | | | <0.001 | | | |  |
| 70-99 | | | 182,016 | | | | | | | | 4,839 | | | 2.10 | | | | 0.93  (0.87–0.98) | | | | 0.013 | | | | | | |  | | | | | 77,443 | | | 7,934 | | | | | | | | 8.99 | | | | 1.01  (0.96–1.06) | | | | 0.729 | |  | | |  | | | |  |
| 100-129 | | | 337,709 | | | | | | | | 8,939 | | | 2.09 | | | | 0.96  (0.91–1.02) | | | | 0.174 | | | | | | | 0.007 | | | | | 129,376 | | | 13,300 | | | | | | | | 8.94 | | | | 1.03  (0.99–1.08) | | | | 0.162 | | <0.001 | | |  | | | |  |
| 130-159 | | | 276,305 | | | | | | | | 7,475 | | | 2.13 | | | | 0.96  (0.91–1.02) | | | | 0.232 | | | | | | |  | | | | | 104,150 | | | 10,781 | | | | | | | | 8.95 | | | | 1.06  (1.00–1.10) | | | | 0.034 | |  | | |  | | | |  |
| ≥160 | | | 164,209 | | | | | | | | 4,776 | | | 2.30 | | | | 1.00  (0.94–1.07) | | | | 0.914 | | | | | | |  | | | | | 60,483 | | | 6,449 | | | | | | | | 9.24 | | | | 1.09  (1.04–1.14) | | | | <0.001 | |  | | |  | | | |  |
| **Triglycerides, mg/dL** | | | | | | | | | | | | | | | | | | | | | | | | | | | | | | | | | | | | | | | | | | | | | | | | | | | | | | | | | | | | | | |
| <100 | | | 443,795 | | | | | | | | 10,132 | | | 1.80 | | | | (ref) | | | | | | | | | | | <0.001 | | | | | 125,671 | | | 11,572 | | | | | | | | 7.95 | | | | (ref) | | | | | | <0.001 | | | <0.001 | | | |  |
| 100-149 | | | 300,007 | | | | | | | | 8,391 | | | 2.21 | | | | 1.08  (1.05–1.12) | | | | <0.001 | | | | | | |  |  |  |  |  | 133,368 | | | 13,790 | | | | | | | | 9.01 | | | | 1.08  (1.05–1.10) | | | | <0.001 | |  |  |  |  | | | |  |
| 150-199 | | | 137,884 | | | | | | | | 4,415 | | | 2.53 | | | | 1.16  (1.12–1.20) | | | | <0.001 | | | | | | |  |  |  |  |  | 70,690 | | | 7,754 | | | | | | | | 9.58 | | | | 1.12  (1.09–1.15) | | | | <0.001 | |  |  |  |  | | | |  |
| ≥200 | | | 118,145 | | | | | | | | 4,377 | | | 2.94 | | | | 1.26  (1.21–1.31) | | | | <0.001 | | | | | | |  |  |  |  |  | 62,173 | | | 7,483 | | | | | | | | 10.56 | | | | 1.22  (1.18–1.26) | | | | <0.001 | |  |  |  |  | | | |  |
| **Abdominal obesity** | | | | | | | | | | | | | | | | | | | | | | | | | | | | | | | | | | | | | | | | | | | | | | | | | | | | | | | | | | | | | | |
| Yes | | | 213,577 | | | | | | | | | 7,812 | | | 2.90 | | | 1.16  (1.12–1.19) | | | | <0.001 | | | | | | | - | | | | | 135,182 | | | 15,030 | | | | | | | | 9.67 | | | | 1.04  (1.02–1.07) | | | | <0.001 | | | - | | | <0.001 | |  |  |
| **Regular physical activity** | | | | | | | | | | | | | | | | | | | | | | | | | | | | | | | | | | | | | | | | | | | | | | | | | | | | | | | | | | | | | | |
| Yes | | | 201,979 | | | | | | | | | 4,997 | | | 1.95 | | | 1.14  (1.11–1.18) | | | | <0.001 | | | | | | | - | | | | | 59,799 | | | 5,123 | | | | | | | | 7.21 | | | | 1.19  (1.15–1.22) | | | | <0.001 | | - | | | <0.001 | | |  |  |

HRs were estimated with adjustment for age, sex, BMI, smoking, alcohol, exercise, hypertension, diabetes, dyslipidemia, and eGFR, excluding the primary exposure variable under analysis. Abbreviations as **Supplementary Table 2** and **3**.

**Supplementary Table 7. Detailed estimates of aggregated PAFs**

|  | **Total** | **Men** | | | **Women** | | |
| --- | --- | --- | --- | --- | --- | --- | --- |
|  |  | All | <50 years old | ≥50 years old | All | <50 years old | ≥50 years old |
| **Aggregated PAF, %**  **(95% CI)** | 46.2  (43.2 – 49.1) | 52.8  (48.8 – 56.6) | 71.5  (65.3 – 76.8) | 47.5  (42.7 – 51.9) | 30.4  (25.6 – 35.0) | 34.4  (25.2 – 42.7) | 34.3  (28.1 – 40.1) |
| **Residual Risk, %**  **(95% CI)** | 53.8  (50.9 – 56.8) | 47.2  (43.4 – 51.2) | 28.5  (23.2 – 34.7) | 52.5  (48.1 – 57.3) | 69.6  (65.0 – 74.4) | 65.6  (57.3 – 74.8) | 65.7  (59.9 – 71.9) |
|  | | | | | | | |
| **Systolic BP, mmHg** | 27.7  (22.6 – 28.1) | 27.4  (20.9-29.4) | 48.6 (33.2-49.8) | 26.4 (19.3-28.9) | 23.3 (17.9-25.3) | 24.5 (16.1-29.1) | 26.4 (19.4-28.7) |
| <100 |  |  |  |  |  |  |  |
| 100-119 | 5.7 (4.3-7.0) | 3.4 (1.7-5.0) | 8.9 (4.7-13.1) | 2.1 (0.7-3.6) | 7.4 (5.2-9.6) | 8.4 (3.9-12.8) | 3.7 (2.0-5.4) |
| 120-139 | 15.3 (13.8-16.8) | 16.1 (13.6-18.6) | 27.6 (22.2-32.9) | 13.1 (10.3-15.8) | 11.6 (10.0-13.2) | 13.0 (10.4-15.6) | 13.2 (10.6-15.7) |
| 140-159 | 4.8 (4.4-5.2) | 5.7 (5.0-6.3) | 9.0 (7.4-10.5) | 7.5 (6.4-8.6) | 3.2 (2.8-3.5) | 2.2 (1.8-2.6) | 6.6 (5.6-7.6) |
| ≥160 | 1.9 (1.7-2.0) | 2.2 (2.0-2.4) | 3.2 (2.7-3.7) | 3.6 (3.2-4.1) | 1.2 (1.1-1.4) | 0.9 (0.8-1.1) | 2.9 (2.5-3.2) |
| **Smoking** | 16.3 (16.0-16.7) | 23.7 (23.2-24.2) | 29.4 (28.2-30.6) | 16.4 (15.9-16.9) | 3.7 (3.3-4.0) | 4.4 (3.4-5.3) | 2.2 (2.0-2.4) |
| Non |  |  |  |  |  |  |  |
| Ex | 0.0 (0.0-0.0) | 0.0 (0.0-0.0) | 0.0 (0.0-0.0) | 0.0 (0.0-0.0) | 0.5 (0.4-0.6) | 0.7 (0.3-1.1) | 0.2 (0.1-0.3) |
| Current | 16.3 (16.0-16.7) | 23.7 (23.2-24.2) | 29.4 (28.2-30.6) | 16.4 (15.9-16.9) | 3.2 (3.0-3.4) | 3.6 (3.1-4.2) | 2.0 (1.8-2.1) |
| **Non-HDL-c, mg/dL** | 9.0 (8.1-9.3) | 12.8 (11.4-13.1) | 21.0 (17.6-21.2) | 10.5 (9.1-11.2) | 3.5 (2.6-4.2) | 3.0 (1.2-4.7) | 6.2 (4.7-7.4) |
| <130 |  |  |  |  |  |  |  |
| 130-159 | 2.4 (2.2-2.7) | 3.1 (2.7-3.5) | 5.0 (4.1-5.9) | 2.5 (2.1-3.0) | 1.1 (0.6-1.5) | 1.1 (0.1-2.0) | 1.2 (0.7-1.7) |
| 160-189 | 3.3 (3.1-3.5) | 4.7 (4.4-5.0) | 7.7 (7.1-8.4) | 3.9 (3.6-4.3) | 1.1 (0.9-1.4) | 0.9 (0.4-1.4) | 2.1 (1.7-2.5) |
| 190-219 | 2.1 (2.0-2.2) | 3.2 (3.0-3.3) | 5.0 (4.7-5.4) | 2.6 (2.4-2.8) | 0.7 (0.6-0.9) | 0.6 (0.4-0.8) | 1.7 (1.4-1.9) |
| ≥220 | 1.2 (1.2-1.3) | 1.9 (1.8-2.0) | 3.2 (3.0-3.4) | 1.4 (1.3-1.5) | 0.5 (0.5-0.6) | 0.4 (0.3-0.6) | 1.3 (1.1-1.4) |
| **Diabete**s | 3.5 (3.3-3.8) | 4.5 (4.1-4.8) | 3.1 (2.4-3.8) | 7.8 (7.1-8.4) | 2.4 (2.1-2.7) | 1.6 (0.9-2.4) | 5.7 (5.0-6.3) |
| Normoglycemia |  |  |  |  |  |  |  |
| Prediabetes | 0.6 (0.4-0.8) | 1.0 (0.7-1.2) | 0.8 (0.3-1.3) | 0.9 (0.5-1.3) | 0.2 (0-0.4) | 0.2 (-0.3-0.8) | 0.2 (-0.1-0.6) |
| Diabetes | 2.9 (2.8-3.0) | 3.5 (3.4-3.7) | 2.3 (2.1-2.5) | 6.9 (6.6-7.2) | 2.2 (2.1-2.3) | 1.4 (1.2-1.6) | 5.4 (5.1-5.7) |
| **BMI, kg/m^2^** | 2.1 (1.7-2.5) | 1.4 (0.8-1.9) | 11.2 (10.1-12.3) | 0.0 (0.0-0.0) | 2.2 (1.7-2.7) | 7.1 (5.8-8.3) | 0.1 (-0.6-0.8) |

PAF, population-attributable fraction; other abbreviation as **Supplementary Table 2** and **3**

**Supplementary Table 8. Sensitivity analysis using Fine–Gray competing risk models for ASCVD**

|  | **HR (95%CI)** | | | **PAF % (95%CI)** | **SHR (95%CI)** | | | **PAF % (95%CI)** | | |
| --- | --- | --- | --- | --- | --- | --- | --- | --- | --- | --- |
| **Aggregated PAF, %** |  | | | 46.2 (43.2 – 49.1) |  | | | 50.4 (48.5 – 53.3) | | |
| **Residual Risk, %** |  | | | 53.8 (50.9 – 56.8) |  | | | 49.6 (47.7 – 51.5) | | |
| **Systolic BP, mmHg** |  |  |  | 27.7 (22.6 – 28.1) |  |  |  | 29.9 (28.4 –33.2) | | |
| <100 | (ref) | | |  | (ref) | | |  | | |
| 100-119 | 1.15 (1.11 – 1.19) | | | 5.7 (4.3 – 7.0) | 1.16 (1.12 – 1.20) | | | 6.3 (4.8 – 7.7) | | |
| 120-139 | 1.40 (1.35 – 1.44) | | | 15.3 (13.8 – 16.8) | 1.44 (1.39 – 1.49) | | | 16.6 (16.6 – 18.4) | | |
| 140-159 | 1.67 (1.61 – 1.72) | | | 4.8 (4.4 – 5.2) | 1.72 (1.66 – 1.78) | | | 5.2 (5.2 –5.2) | | |
| ≥160 | 2.10 (2.03 – 2.18) | | | 1.9 (1.7 – 2.0) | 2.09 (2.01 – 2.17) | | | 1.8 (1.8 – 1.9) | | |
| **Smoking** |  |  |  | 16.3 (16.0 – 16.7) |  |  |  | 14.5 (14.1 – 14.9) | | |
| Non | (ref) | | |  | (ref) | | |  |  |  |
| Ex | 0.99 (0.98 – 1.00) | | | 0.0 (0.0 – 0.0) | 0.98 (0.97 – 1.00) | | | 0.0 (0.0 – 0.0) | | |
| Current | 1.72 (1.70 – 1.74) | | | 16.3 (16.0 – 16.7) | 1.62 (1.60 – 1.64) | | | 14.5 (14.1 – 14.9) | | |
| **Non-HDL-c, mg/dL** |  |  |  | 9.0 (8.1 – 9.3) |  |  |  | 12.6 (11.8 – 13.3) | | |
| <130 | (ref) | | |  | (ref) | | |  |  |  |
| 130-159 | 1.09 (1.08 – 1.10) | | | 2.4 (2.2 – 2.7) | 1.14 (1.13 – 1.15) | | | 3.9 (3.6 – 4.2) | | |
| 160-189 | 1.21 (1.20 – 1.22) | | | 3.3 (3.1 – 3.5) | 1.29 (1.28 – 1.31) | | | 4.5 (4.3 – 4.7) | | |
| 190-219 | 1.36 (1.35 – 1.38) | | | 2.1 (2.0 – 2.2) | 1.48 (1.45 – 1.50) | | | 2.7 (2.6 – 2.8) | | |
| ≥220 | 1.60 (1.57 – 1.63) | | | 1.2 (1.2 – 1.3) | 1.70 (1.66 – 1.73) | | | 1.5 (1.4 – 1.5) | | |
| **Diabete**s |  |  |  | 3.5 (3.3 – 3.8) |  |  |  | 2.6 (2.3 – 2.9) | | |
| Normoglycemia | (ref) | | |  | (ref) | | |  |  |  |
| Prediabetes | 1.03 (1.02 – 1.04) | | | 0.6 (0.4 – 0.8) | 1.03 (1.02 – 1.04) | | | 0.60 (0.39 – 0.81) | | |
| Diabetes | 1.48 (1.47 – 1.50) | | | 2.9 (2.8 – 3.0) | 1.34 (1.32 – 1.35) | | | 2.04 (1.94 – 2.13) | | |
| **BMI, kg/m^2^** |  |  |  | 2.1 (1.8 – 2.5) |  |  |  | 7.1 (6.5 – 7.8) | | |

**SHR, subdistribution hazard ratio**; other abbreviation as **Supplementary Table 2**, **3**, and **7**.

**Supplementary Table 9.** **PAFs of five major modifiable risk factors for CV death**

|  | Total | Men | Women |
| --- | --- | --- | --- |
|  | **PAF, %**  **(95% CI)** | **PAF, %**  **(95% CI)** | **PAF, %**  **(95% CI)** |
| BMI, kg/m^2^ | 0.0 (0.0 – 0.0) | 0.0 (0.0 – 0.0) | 0.0 (0.0 – 0.0) |
| Smoking | 19.4 (18.7 – 20.2) | 25.2 (24.1 – 26.3) | 5.3 (4.6 – 6.0) |
| Systolic BP, mmHg | 24.9 (17.4 – 32.6) | 17.1 (10.3 – 24.0) | 24.3 (13.3 – 35.5) |
| Diabetes | 3.6 (3.0 – 4.2) | 4.1 (3.3 – 5.0) | 2.6 (1.9 – 3.3) |
| Non-HDL-c, mg/dL | 2.0 (1.4 – 2.7) | 3.2 (2.2 – 4.2) | 0.2 (-4.1 – 1.0) |
| Overall PAF | 41.4 (35.1 – 47.4) | 41.8 (35.3 – 48.0) | 28.5 (15.0 – 37.2) |
| Residual Risk | 58.6 (52.6 – 64.9) | 58.2 (52.0 – 64.7) | 71.5 (62.8 – 85.0) |

Abbreviation as **Supplementary Table 2**, **3**, and **7**.

**Supplementary Table 10. PAFs of five major modifiable risk factors for MI**

|  | Total | Men | Women |
| --- | --- | --- | --- |
|  | **PAF, %**  **(95% CI)** | **PAF, %**  **(95% CI)** | **PAF, %**  **(95% CI)** |
| BMI, kg/m^2^ | 7.2 (6.5 – 7.9) | 9.5 (8.5 – 10.4) | 2.4 (1.2 – 3.6) |
| Smoking | 23.0 (22.0 – 24.1) | 33.0 (31.6 – 34.5) | 4.9 (4.1 – 5.8) |
| Systolic BP, mmHg | 24.4 (17.9 – 31.0) | 20.9 (12.3 – 29.9) | 20.5 (11.2 – 29.9) |
| Diabetes | 4.4 (3.9 – 5.0) | 5.0 (4.2 – 5.7) | 3.9 (3.1 – 4.8) |
| Non-HDL-c, mg/dL | 24.8 (23.3 – 26.3) | 33.0 (30.6 – 34.6) | 9.0 (7.0 – 11.1) |
| Overall PAF | 59.0 (54.1 – 63.4) | 66.8 (61.2 – 71.7) | 34.1 (23.7 – 43.7) |
| Residual Risk | 41.1 (36.6 – 45.9) | - 1. (28.3 –38.8) | 65.9 (56.3 – 76.3) |

Abbreviation as **Supplementary Table 2**, **3**, and **7**.

**Supplementary Table 11. PAFs of five major modifiable risk factors for Ischemic stroke**

|  | Total | Men | Women |
| --- | --- | --- | --- |
|  | **PAF, %**  **(95% CI)** | **PAF, %**  **(95% CI)** | **PAF, %**  **(95% CI)** |
| BMI, kg/m^2^ | 2.1 (1.8 – 2.5) | 1.4 (0.8 – 1.9) | 2.7 (2.1 – 3.3) |
| Smoking | 13.0 (12.5 – 13.4) | 19.9 (19.2 – 20.6) | 2.9 (2.5 – 3.3) |
| Systolic BP, mmHg | 27.1 (23.0 – 31.2) | 31.6 (25.0 – 38.3) | 24.0 (18.9 – 29.1) |
| Diabetes | 3.7 (3.4 – 4.0) | 5.0 (4.5 – 5.6) | 2.4 (2.0 – 2.8) |
| Non-HDL-c, mg/dL | 6.5 (5.8 – 7.3) | 8.8 (7.7 – 9.9) | 4.2 (3.3 – 5.2) |
| Overall PAF | 42.3 (37.7 – 46.7) | 50.6 (45.1 – 55.8) | 31.2 (25.6 – 36.6) |
| Residual Risk | 57.7 (53.3 – 62.3) | 49.4 (44.3 – 54.9) | 68.8 (63.4 – 74.4) |

Abbreviation as **Supplementary Table 2**, **3**, and **7**.
